# Smooth Interpolating Curves with Local Control and Monotone Alternating Curvature

**DOI:** 10.1111/cgf.14600

**Published:** 2022-10-06

**Authors:** Alexandre Binninger, Olga Sorkine‐Hornung

**Affiliations:** ^1^ ETH Zurich Switzerland

**Keywords:** CCS Concepts, • ***Computing methodologies*** → ***Parametric curve and surface models***

## Abstract

We propose a method for the construction of a planar curve based on piecewise clothoids and straight lines that intuitively interpolates a given sequence of control points. Our method has several desirable properties that are not simultaneously fulfilled by previous approaches: Our interpolating curves are C^2^ continuous, their computation does not rely on global optimization and has local support, enabling fast evaluation for interactive modeling. Further, the sign of the curvature at control points is consistent with the control polygon; the curvature attains its extrema at control points and is monotone between consecutive control points of opposite curvature signs. In addition, we can ensure that the curve has self‐intersections only when the control polygon also self‐intersects between the same control points. For more fine‐grained control, the user can specify the desired curvature and tangent values at certain control points, though it is not required by our method. Our local optimization can lead to discontinuity w.r.t. the locations of control points, although the problem is limited by its locality. We demonstrate the utility of our approach in generating various curves and provide a comparison with the state of the art.

## 1. Introduction

Curve modeling is a fundamental field of computer graphics and is at the cornerstone of computer‐aided geometric design [FF02], spanning over domains as diverse as sketching, shape representation, drawing, shape completion, curve fitting and animation. One major distinction in curve modeling lies between approximation and interpolation, the latter requiring the curve to pass through the control points, which is desirable in many applications. Our work focuses on the interpolation of a sequence of planar points (a control polygon) without parameterization constraints.

There are infinitely many curves passing through a set of points, thus the interpolation problem is by nature ill posed. In practice, various mathematical properties and guarantees are desired in applications, enabling characterization of different interpolation methods and construction of new ones. One such major property is *continuity*, which measures the smoothness of a curve. If a curve is parameterized such that its derivatives up to order *n* exist and are continuous, we say that the curve is *C^n^.* A curve is said to be *G^n^* geometrically continuous if its arc length reparameterization is *C^n^.* Second order geometric continuity is generally considered necessary for producing fair planar curves. Another important property is locality: if moving a control point modifies the curve shape within a bounded number of neighboring control points, the curve is said to have *local support.* Preventing unintended *self‐intersections* or *cusps* is another component of shape fairness. Levien [Lev09] discusses such properties in detail.

There is no conclusive evidence that accumulating mathematical properties is directly related to designing fair and good looking curves. The fairness of a curve remains a poorly mathematically defined concept [LS09], but there is a strong indication based on psychological science that curvature variation plays a major role in our perception of a shape [Att74,Zia16]. This advocates for curves where curvature extrema are located at control points, implying monotonicity of curvature between two consecutive control points.

The curves we produce are formed by stitching clothoids and straight lines in a *G* ^2^ manner. We start by locally estimating the tangent and curvature objectives at each control point and then locally modify the curvature to enable the transition between two consecutive control points. With regards to the aforementioned properties, our contribution consists in producing a curve parameterized by arc‐length with guarantees that have not previously been ensured simultaneously, to our knowledge: *G* ^2^‐continuity, local support of 6 or 8 points, monotone curvature between control points with alternate curvature sign, curvature extrema at control points and no self‐intersection if the control polygon does not present a self‐intersection between the respective control points. Moreover, our method also permits further modeling options, since the user can manually set curvature and tangent constraints at control points. Yet, solving local optimization problems and enforcing the consistency of the curvature sign with the control polygon brings one major drawback: the lack of continuity with respect to the locations of control points. We compare our approach to the state of the art and discuss its advantages and limitations. Our code is publicly available here to foster further research in this field.

## 2. Related work

A substantial body of work on interpolating curve construction exists. We only present a subset of this rich domain and refer the reader to [HL93] and [CDH∗07] for a more detailed presentation.

**Interpolation methods.** Catmull‐Rom splines [CR74] provide a *G* ^2^ interpolation method based on local Lagrange interpolations blended via Bézier curves and B‐splines. Catmull‐Rom splines with centripetal parameterization do not produce cusps or self‐intersections within the curve segments [YSK09]. Interpolating *n* points via *G* ^2^ cubic splines [FF02] can be done by solving a linear system to find *n*+2 control points of a corresponding B‐spline. Contrary to our work, these methods take as input the parameter values *t_i_* such that the resulting curve α interpolates the control points *p_i_* on these values, i.e., α(*t_i_*) = *p_i_.* Our method does not require such additional constraints. Note that the distinction between *C* ^2^ and *G* ^2^ continuity is irrelevant without parameterization constraints, since any *G* ^2^ curve has a *C* ^2^ parameterization by definition.

Several methods use blending functions without parameterization constraints. Linear functions can be used to blend circular interpolations of triplets of consecutive control points [Wen96], but the result is only *G* ^1^ continuous. Trigonometric blending functions raise the continuity to *G* ^2^ [SNV00]. Theses curves reproduce exact circles if four consecutive points are lying on a circle, but are prone to self‐intersections, cusps and unbounded distance to the control polygon. Using trigonometric blending directly on the tangent angles estimated by circle fitting at control points [SLY05] produces cusps‐free curves. A scheme based on quadratic and trigonometric functions blending local conic interpolation produces curves compatible with nonuniform rational basis splines [SZ09]. Trigonometric blending of hybrid circular‐elliptical interpolations [Yuk20] produces curves without cusps or self‐intersections between two consecutive control points, but does not prevent self‐intersections between different curve segments. In addition, these curves are *G* ^2^ continuous and have bounded distance to the control polygon. Polynomial blending functions with vanishing derivatives at the boundary produce curves of arbitrarily high order of continuity [Pob13]. All such blending methods provide continuity with local support and are easy to compute, but fail to ensure piecewise curvature monotonicity and curvature extrema at control points.

Methods producing curves with curvature extrema at control points usually rely on global optimization techniques and do not have local support. Havemann et al. [HEWF13] propose a global iterative algorithm based on piecewise clothoids. They estimate curvature based on circle interpolation of neighbouring points and use it to add a new point between each control point. The curvature information is therefore globally propagated, and this method does not provide local support. Like in our method, the control polygon should be sparse in order to offer intuitive user control and avoid artifacts. Another proposal are κ‐curves [YSW∗17]: these are piecewise‐quadratic curves based on an iterative optimization process to set Bézier control points, which produces *G* ^2^ splines except at inflection points. Miura et al. [MGS∗21] extend the concept of κ‐curves to ∊κ‐curves by using cubic instead of quadratic Bézier curves. This provides user control over the magnitude of the curvature extrema at the input points and can reduce the gap in curvature at inflection points. Yan et al. [YSS19] can additionally reproduce circles and ellipses by using rational quadratic Bézier interpolation primitives and the additional degree of freedom to minimize eccentricity of interpolating conics. A similar optimization process to κ‐curves can be used on log‐aesthetics interpolating curves [WGS∗20] to produce *G* ^2^ curves. Bézier curves can also produce splines with monotone curvature under certain conditions [Far06]. In contrast to this type of techniques, our method has both local support and curvature monotonicity between consecutive control points when the control polygon implies curvature of alternating sign.

**Clothoids.** Clothoids are curves whose curvature varies linearly with arc length. They present particularly beneficial fairness properties for transition curves, which justifies their use in a large variety of domains [Lev09], ranging from mechanical engineering of road design [MJF15] to shape completion [KFP03] or sketching [MS09,BLP10]. Fitting a *G* ^2^ curve using piecewise clothoids is possible using global optimization with nonlinear programming [Sto82]. Bertolazzi and Frego [BF18a] obtain a clothoid‐based *G* ^2^ spline by building a nonlinear system with two degrees of freedom. With these additional degrees of freedom, they globally optimize over a set of nine problems including boundary conditions, minimization of length, and integrated curvature. Similar to other global optimization methods, this approach lacks local support.

Our method is based on the evaluation of tangents and curvatures at control points and fitting a piecewise clothoid according to these conditions. Interpolating two given points with assigned tangent vectors is always possible [WM09, BF15] and is referred to as the Hermite *G* ^1^ interpolation problem. Finding a clothoid curve to join two points with tangent and curvature constraints is not necessary feasible, but the set of points that allows a Hermite *G* ^2^ transition via monotone piecewise clothoids has been explicitly determined [MW92]. Without the curvature monotonicity condition, it is always possible to find a 3‐arcs clothoid transition that solves the Hermite *G* ^2^ problem [BF18b].

Our method relies on several research tasks around clothoids. Finding the shortest transition clothoid from a point with second order constraints to a line with a target angle is explicitly derived in [VMCU16] and is at the foundation of our clothoid‐line‐clothoid transition method. We also rely on the detection of intersections between clothoids [BBF20] to prevent self‐intersections that are inconsistent with the control polygon. Clothoids formulation can be framed within Fresnel integrals, which are transcendental functions [ASR66], but extensive research has been conducted to ease their computation. Fresnel integrals can be approximated via rational functions [Hea85] or s‐power series [SRC03], which are much easier to compute. A comparison between the explicit formulas based on Taylor expansion and an alternative method based on the explicit Euler method can be found in [VMCU16].

## 3. Background

We set the notation and review the necessary concepts related to clothoids. We denote by *p_i_* ∊ ℝ^2^ the *n* input points. The curve's tangent at *p_i_* is denoted as *T_i_* and the curvature as *K_i_.* We can represent *T_i_* by the angle θ_*i*_ it makes with the *x*‐axis. The *control polygon* is the set of segments generated by the ordered control points, i.e., the union of segments [*p*_*i*_, *p*_*i* + 1_].

### 3.1. Clothoids

Clothoids are curves whose curvature variation is linear w.r.t. arc length. Perusing the notation in [BF15, BF18b, BF18a], if α(*t*) is a curve parameterized by arc length, then α is a clothoid if its curvature is of the form

(1)

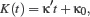




where κ′ and κ_0_ are constants. Let *T*(*t*) = (cos θ(*t*), sin θ(*t*))^T^ be the tangent vector field of the curve. Since the derivative of the tangent's slope angle is the curvature, θ′(*t*) = *K*(*t*), we can write


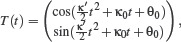




and the expression of the curve is recovered as

(2)

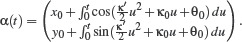




Clothoids are often studied within the frame of Fresnel integrals


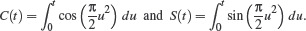




**3‐arcs clothoids.** Given boundary conditions (*p*_0_, θ_0_, *K*_0_) and (*p*_1_, θ_1_, *K*_1_) for the start and end points, it is always possible to find a transition made of three pieces of clothoids connected in a *G* ^2^ manner that matches these conditions [BF18b]. We refer to this as the *3‐arcs clothoid transition.*

### 3.2. Clothoid shell

Part of our method is based on finding a smooth clothoid‐line‐clothoid (CLC) transition. For this purpose, we need to compute the shortest clothoid that makes a *G* ^2^ transition between a starting point with boundary conditions (*p*_0_, θ_0_, *K*_0_) and a straight line, such that the tangent angle with the *x*‐axis of the clothoid at the transition point to the line is Φ. See Fig. 2 for an illustration. This problem is studied in [VMCU16], and we summarize the results here. Assume w.l.o.g. that *K*_0_ > 0. Let *L* be the length of the clothoid. A *G* ^2^ transition to a straight line implies that the end curvature vanishes: *K*(*L*) = 0, and from the linear expression of clothoid curvature (Eq. (1)) we get κ′ *L* = — κ_0_. The condition θ(*L*) = Φ implies 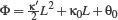, i.e., *L* = 2(Φ – θ_0_)/κ_0_.

**Figure 1:**
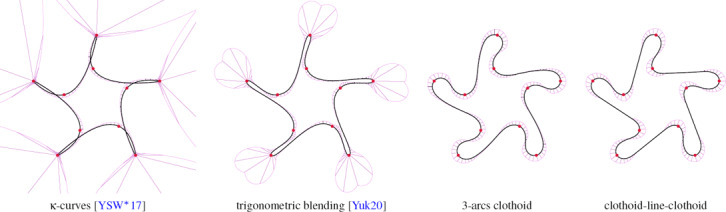
Interpolating curves generated from the same control points (shown in red) using κ‐curves [YSW∗17], trigonometric blending [Yuk20], our 3‐arcs clothoids method and our clothoid‐line‐clothoid method. Our approach guarantees G^2^ continuity, has bounded local support and provides curvature monotonicity between control points of opposite curvature sign. The curvature normals are visualized with purple lines.

**Figure 2:**
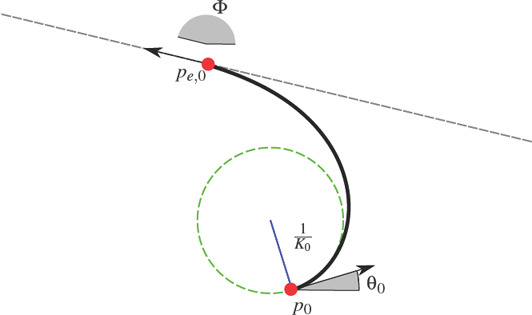
Clothoid‐line transition problem: find a clothoid starting at point p *_0_* with starting tangent angle *θ_0_* and curvature K *_0_* that makes a G *^2^‐*smooth transition to a straight line of target angle *Φ*. Note that the target is the angle *Φ*, not a precise spatial positioning of the line. The G *^2^* condition means that the curvature of the clothoid at the endpoint p_e,0_ must be zero.

The end point of the clothoid can be found by substituting the expression for *L* in Eq. (2). Given the relations between *L*, κ_0_ and κ′, and substituting the variable *u* in the integral in Eq. (2) with a parameter *s* ∊ [0, 1], such that *u* = *Ls*, one gets


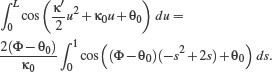




The same derivation applies for the second Fresnel integral. Therefore, the end point *p_e,i_* (Φ) found for a clothoid‐line transition with starting conditions (*p_i_*, θ_*i*_, *K*_*i*_) and target angle Φ ∊ [θ_*i*_, θ_*i*_ + 2π] is

(3)

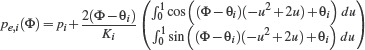




**Figure 3:**
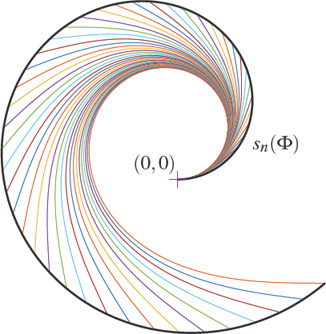
Normal clothoid shell (black curve) for *Φ ∊ [0, 2π]*. The colored curves are the shortest clothoids emanating from the starting condition at the origin, namely (p*_0_, θ_0_*, K *_0_) = ((0, 0)^T^, 0, 1)* and connecting with G *^2^* smoothness to a straight line of slope angle *Φ*.

The following function *s_n_* : [0, 2π] → ℝ^2^ is termed the *normal clothoid shell*, see Fig. 3:

(4)

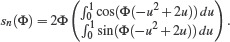




It maps the angle Φ to the end point of the shortest clothoid starting at the origin with tangent slope angle 0 and curvature 1 and *G* ^2^‐smoothly transitioning to a straight line of slope angle Φ. Note that *p_e,i_* (Φ) can be obtained for Φ ∊ [θ_*i*_, θ_*i*_ + 2π] using the normal clothoid shell *s_n_* (Φ) via a translation by *p_i_*, scaling by 1*/K_i_*, rotation 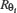 by angle θ_*i*_ and shifting by –θ_*i*_:


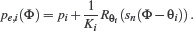




### 3.3. Clothoid‐line‐clothoid (CLC) transition

**Figure 4:**
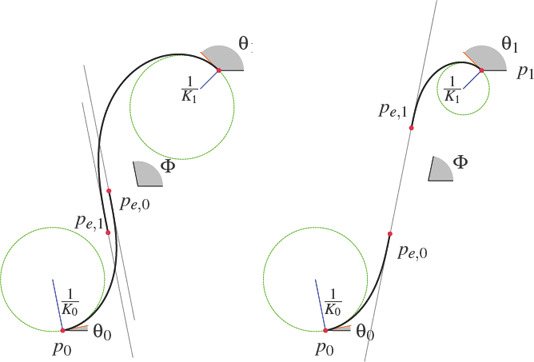
A CLC‐transition is not guaranteed to exist. On the left, we show a case where the boundary conditions at the endpoints prevent finding a single straight line to which two clothoid pieces could connect G *^2^‐*smoothly. On the right we show a feasible CLC configuration.

The clothoid shell provides us with a clothoid‐line transition, namely a *G* ^2^ interpolation of a starting point with prescribed tangent and curvature values and end point tangent and vanishing curvature conditions, but not the end point *location.* For our curve interpolation task, we are interested in smoothly connecting control points by pieces of clothoids and straight lines, hence we are seeking a *G* ^2^ clothoid‐line‐clothoid (CLC) transition, where the clothoid pieces connect smoothly to *the same* straight line. A smooth CLC transition is not guaranteed to exist, see Fig. 4. However, we can prove the following statement:

**Proposition 3.1** Let *p*_0_, *p*_1_ be two distinct points and θ_0_ and θ_1_ two angles. There exist curvature bounds *K*_0_, *K*_1_, such that for any κ_0_ ≥ *K*_0_, κ_1_ ≥ *K*_1_, there exists an angle Φ such that the line segment [*p*_*e*,0_(Φ), *p*_*e*,1_(Φ + π)] makes an angle Φ with the *x*‐axis, where the starting conditions for the two clothoids are (*p*_0_, θ_0_, κ_0_) and (*p*_1_, θ_1_, κ_1_), respectively.

*Proof* See appendix. □

Hence by prescribing sufficiently high curvature at control points, Proposition 3.1 ensures the existence of a transition consisting of a clothoid from *p*_0_ to *p*_*e*,0_(Φ), a straight line from *p*_*e*,0_(Φ) to *p*_*e*,1_(Φ + π), and a clothoid from *p*_*e*,1_ (Φ + π) to *p*_1_. Note that we prove the existence of an angle Φ that is equal to the angle of the tangent at *p*_*e*,0_ and to the angle of the tangent at *p*_*e*,1_ plus π, that is to say, the existence of a CLC transition where the end points of the clothoids are not in a reversed order on the transition line. This transition can be as close as desired to the straight segment [*p*_*i*_, *p*_*i* + 1_] by taking sufficiently high curvatures, as shown below.

**Proposition 3.2** Let *p*_0_, *p*_1_ be two distinct points and θ_0_, θ_1_ two angles. If the absolute value of κ_0_ and κ_1_ simultaneously tends to infinity, then the *G* ^2^‐smooth CLC transition between boundary conditions (*p*_0_, θ_0_, κ_0_) and (*p*_1_, θ_1_, κ_1_) uniformly converges to the segment [*p*_0_, *p*_1_].

*Proof* For all Φ ∊ [0, 2π], applying Jensen's inequality, we have


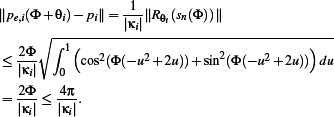




If |κ_*i*_| tends to infinity, then *p_i,e_* (Φ + θ_*i*_) converges to *p_i_* for all Φ. Hence the end points of the clothoid parts of the CLC transition, *p*_*e*,0_ and *p*_*e*,1_, converge to *p*_0_ and *p*_1_, whereby the clothoid pieces become increasingly shorter, and the line [*p*_*e*,0_, *p*_*e*,1_] converges to [*p*_0_, *p*_1_]. □

## 4. Method

**Figure 5:**
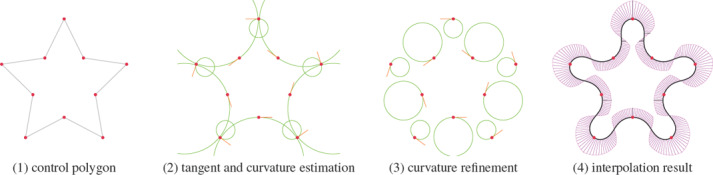
Summary of our method. (1) We start with an ordered set of points p_i_. (2) We then estimate the tangent slope angle *θ* and curvature K_i_ at each control point (Sec. 4.1). (3) We apply our refinement method to increase the curvatures at control points so that a G *^2^* transition becomes feasible (Sec. 4.2). (4) Finally, we fit a transition curve between eachpair of control points that matches the tangent and curvature conditions.

We propose two interpolating methods. The first method is based on finding a transition composed of 3‐arcs clothoids [BF18b], and the second one is based on *G* ^2^ clothoid‐line‐clothoid transition. Both rely on a local estimation of tangent and curvature values at control points (Sec. 4.1) followed by a curvature refinement procedure (Sec. 4.2) to achieve the desired curve properties. Our method is summarized in Fig. 5 and Algorithm 1. We provide the code in the supplemental material.

**Algorithm 1**: *G* ^2^ interpolating curve

**Table:** 

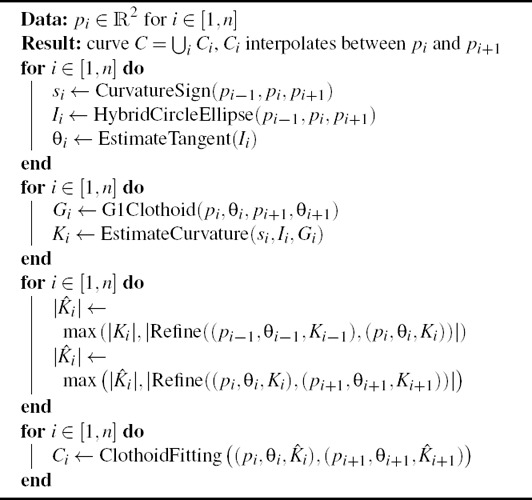

### 4.1. Tangent and curvature estimation at control point

Estimation of the tangent *t_i_* and curvature *K_i_* at a control point *p_i_* is a key step in our method. This estimation must be done using a bounded number of consecutive points to keep local support. We start by applying the approach in [Yuk20] that locally fits circles or ellipses, relying on only 3 consecutive points, *p*_*i* – 1_, *p*_*i*_, *p*_*i* + 1_. If the three points are collinear, the resulting interpolation is a line segment; the curvature at *p_i_* is then estimated to be 0 and the tangent is set to *t_i_* = (*p_i_* – *p*_*i* – 1_)/‖*p_i_* – *p*_*i* –1_‖. In a general configuration, circles are uniquely determined by 3 points. For ellipses, the method adds the constraint that *p_i_* should be on one of the two axes of the ellipse *(primary axis*), one of the neighboring control points *p*_*i* – 1_ or *p*_*i* + 1_ should be on the other axis *(secondary axis)*, and the remaining point on the other side of the primary axis. These added constraints define the ellipse uniquely. The choice of whether to fit a circle or an ellipse depends on the angles corresponding to the circular arcs 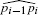 and 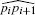. We fit an ellipse if one of the angles is greater than π/2, and a circle otherwise. We refer to section 4 of [Yuk20] for a more detailed explanation.

**Figure 6:**
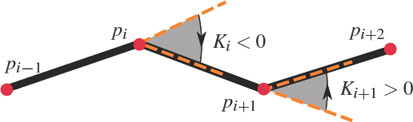
The sign of the curvature at control points is given by the control polygon.

We compute the tangent at a control point *p_i_* from the local interpolation described above. It is possible to also take the curvature of the circle or ellipse interpolant as the initial guess, but such curvature values can lead to sub‐optimal results. For example, as shown in Fig. 11, the local ellipse fitting can produce unnecessarily high curvature that hurts the curve's appearance. We propose using an alternative curvature estimation method via *G* ^1^ clothoid fitting. The curvature should be oriented in a consistent manner with the control polygon, i.e., the sign of *K_i_* should be equal to the sign of the principal angle formed by vectors *p_i_* – *p*_*i* – 1_ and *p*_*i* + 1_ – *p_i_* (see Fig. 6). Since the *G* ^1^ Hermite interpolation is a good approximation of the *G* ^2^ Hermite interpolation problem [BF18b], we perform two *G* ^1^ clothoid fittings with endpoint locations and tangents constraints (*p*_*i* – 1_, *t*_*i* – 1_, *p_i_, t_i_*) and (*p*_*i*_, *t*_*i*_, *p*_*i* + 1_, *t*_*i* + 1_) [BF15]. *Let* 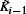 and 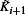 be the resulting curvature of the *G* ^1^ fitting at *p_i_.* We set the target curvature at *p_i_* as the average 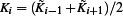. In case one of the two curvatures 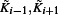 has opposite sign to the control polygon, we instead set *K_i_* to be equal to the curvature estimated from the local circle/ellipse interpolation, whose sign is always consistent with the control polygon.

### 4.2. Curvature refinement

As we discuss in the following, the curvature estimated in the previous section does not necessarily allow for a solution that has monotone curvature (3‐arcs clothoid) or even exists (CLC). Global optimization of the curvature prescribed at control points would remove the strong locality property. Fortunately, as we saw, starting from a certain threshold, increasing the prescribed curvature maintains the existence of a CLC‐transition. For each point *p_i_*, it is therefore possible to find the smallest curvatures that satisfy the desired properties for the segments (*p*_*i* – 1_, *p_i_*) and (*p*_*i*_, *p*_*i* + 1_), and choose the curvature with the largest absolute value. In practice, we can always find the threshold curvatures for points with curvature of alternate signs, but this can fail for consecutive curvatures of same sign. Solutions to this issue are studied in Sec. 5.1. We describe our approach in more detail in Sections 4.2.1 and 4.2.2.

#### 4.2.1. 3‐arcs clothoid transition

**Figure 7:**
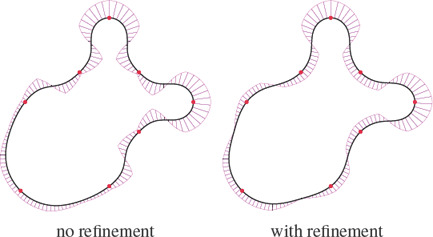
The 3‐arcs clothoid transition without curvature refinement (left) and with curvature refinement (right). With our refinement, the curvature is monotone between control points. In this example, the initial curvatures were estimated using the hybrid circle‐ellipse fitting of [Yuk20].

Given two boundary conditions (*p_i_*, θ_*i*_, *K*_*i*_) and (*p*_*i* + 1_, θ_*i* + 1_, *K*_*i* + 1_), it is always possible to find a curve composed of 3 clothoids with a *G* ^2^ connection interpolating between these conditions [BF18b]. However the resulting curve does not necessarily possess a monotone curvature profile between two control points of opposite curvature sign (see Fig. 7). Intuitively, if the curvature at control points is set sufficiently high, the curve has a monotone curvature profile.

For consecutive points with curvature of the same sign, there is no obvious way to deal with the curvature at control points without resorting to global optimization. If the curvature at a control point is too high, the curvature profile of the resulting curve can exhibit opposite sign to compensate. If the curvature is set too low, then the curvature extrema are not reached at control points. To avoid the latter problem and for consistency with the alternate sign case, we opt to increase the curvature if it does not reach its maximum at control points, even though the curvature can shift sign between two control points.

The increase of curvature is computed as follows. First, we initialize the target curvatures 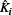 with the estimated curvature *K_i_.* For pairs of consecutive points *p*_*i*_, *p*_*i* + 1_, we solve a *G* ^2^ Hermite problem with a 3‐arc clothoid with constraints (*p_i_*, θ_*i*_, *K*_*i*_) and (*p*_*i* + 1_, θ_*i* + 1_, *K*_*i* + 1_) as described in [BF18b]. If the resulting curve does not attain curvature extrema at control points, we jointly increase the curvatures *K_i_* and *K*_*i* + 1_. The choice of the increase function is discussed in Sec. 4.2.3. Then, 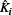 is set to be the largest (in terms of absolute value) of the curvature increases for both problems *p*_*i* – 1_, *p_i_* and *p*_*i*_, *p*_*i* + 1_. Since 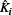 only depends on *K*_*i* – 1_, *K_i_* and *K*_*i* + 1_, curvature refinement somewhat increases the local support but maintains bounded locality. We analyze the local support in detail in Sec. 5. The algorithm is summarized in Algorithm 2.

**Algorithm 2**: Curvature refinement method

**Table:** 

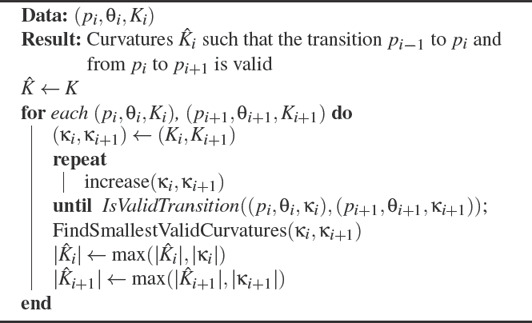

#### 4.2.2. Clothoid‐line‐clothoid transition

Given two boundary conditions (*p_i_*, θ_*i*_, *K*_*i*_) and (*p*_*i* + 1_, θ_*i* + 1_, *K*_*i* + 1_), a *G* ^2^ CLC is not guaranteed to exist. If such a transition does exist, its curvature varies linearly from *K_i_* to 0 (first clothoid), continues as constant 0 (straight line), and finally goes linearly from 0 to *K*_*i* + 1_ (second clothoid). With such a curvature profile, a CLC transition attains curvature extrema at control points and curvature monotonicity between control points of opposite curvature sign. If the straight line portion has vanishing length, the result is a 2‐arcs clothoid.

The angle Φ entirely determines the parameters of both clothoids of the transition curve (Sec. 3.2). Computing the CLC transition amounts to finding Φ such that the vector *v*(Φ) = *p*_*e,i* + 1_ (Φ + π) – *p_e,i_* (Φ) makes an angle Φ with the *x*‐axis. We use a binary search method for this purpose and find the value of Φ ∊ [θ_*i*_, θ_*i*_ + π] that gives the best alignment of *v*(Φ) with *T*(Φ) = (cos(Φ), sin(Φ))^T^. Alignment is measured as ‖*v*(Φ) × *T*(Φ)‖. We reject values of Φ for which *v*(Φ) · *T*(Φ) is negative, as it means that the points are aligned in the wrong order.

The increase of curvature is analogous to Sec. 4.2.1. The target curvatures 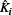 are initialized with the estimated curvatures *K_i_.* Then, for both conditions (*p_i_*, θ_*i*_, *K*_*i*_), (*p*_*i* + 1_, θ_*i* + 1_, *K*_*i* + 1_), we find Φ that achieves the best alignment. If the alignment error is above a certain threshold or if *v*(Φ) · *T*(Φ) < 0, we jointly increase *K_i_* and *K*_*i* + 1_ and set 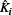 to be the maximum (in terms of absolute value) found for both problems. The locality is affected in the same way as for the 3‐arcs curves.

**Intersections.** Self‐intersections are often seen as undesirable yet unavoidable in the general case. We claim that our method can produce curves with intersections consistent with those of the control polygon, i.e., the piece of curve *C_i_* does not intersect with *C_j_* if the straight segment (*p*_*i*_, *p*_*i* + 1_) does not intersect with (*p*_*j*_, *p*_*j* + 1_). According to Proposition 3.2, we can approximate the control polygon by taking arbitrarily high curvature. Therefore, we can use the same principle as the previous sections to increase the curvature if necessary. First, we check whether the control polygon self‐intersects. If not, we apply Algorithm 2 where the function *InvalidTransition* returns *true* if the two curves intersect. These two curves are composed of straight lines and clothoids, for which the intersection detection can be performed via curve segmentation [BBF20].

**Figure 8:**
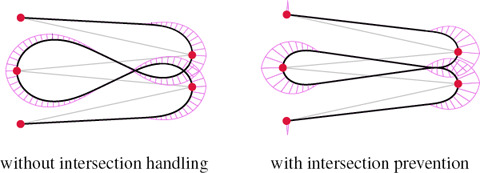
CLC transition with intersection, and with further refinement of the curvature to prevent intersections that do not occur within the control polygon (shown in grey).

Intersection detection and locality are opposite targets if we apply intersection detection on the whole curve. It is however possible to restrict the intersection detection and the refinement to adjacent curves to keep the local support property. We show in Fig. 8 the result of our curvature refinement via intersection detection.

#### 4.2.3. Increase function for the curvature

We consider two strategies when increasing the curvature at control points: linear and a max‐linear increase. Both rely on an increase parameter τ > 1. The linear increase function scales both curvatures *K_i_* and *K*_*i* + 1_ by the parameter τ. This leads to an undifferentiated treatment of the two curvatures. In contrast, the max‐linear increase prioritizes increasing the smaller curvature. Assume |*K_i_* | < |*K*_*i* + 1_|, and let τ_*l*_ = |*K*_*i* + 1_|/|*K_i_* |. The max‐linear increase is defined as


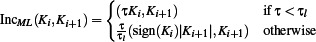




Since we want the increase to be only as high as necessary, the modification of τ has to undergo two phases. First, τ is initialized to 1, which is equivalent to no increase of curvature. If the resulting curve is not valid, in the sense defined for each respective method in Sections 4.2.1 and 4.2.2, we add to τ a positive step ∊_*t.*_ We double the step ∊_*t* + 1_ = 2∊_*t*_ until we reach a coefficient τ_max_ high enough for a valid transition. Once (*K*_*i*_, *K*_*i* + 1_) is high enough for a valid transition, we know that τ must be in the range [τ_max_ – ∊_*t*_, τ_max_]. We can then operate a binary search on this interval.

**Figure 9:**
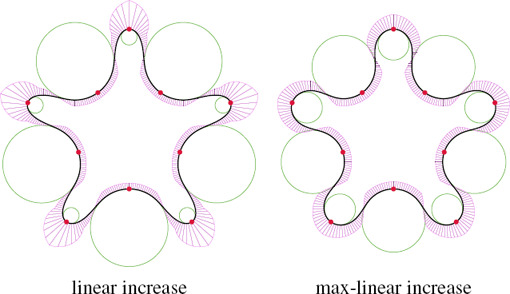
The linear and the max‐linear increase functions give different results. We show their effect in this example using 3‐arcs clothoid transition, where the control point curvatures are initialized by circle‐ellipse fitting. The osculating circles are shown in green, and the scaled curvature normals in purple.

The different results given by the increase functions are showcased in Fig. 9. While linear increase maintains proportionality between curvature profiles, the max‐linear strategy produces more homogeneous curvatures among the control points.

### 4.3. Interactive input of tangents and curvatures

**Figure 10:**
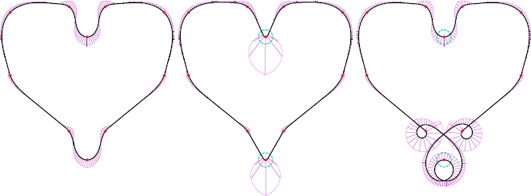
Allowing the user to input curvature and tangent constraints offers additional modeling possibilities. We show the reference shape (left), the shape with user‐modified higher curvature at top and bottom (middle), and a result where input curvature at the bottom tip is set inconsistently with the sign of the control polygon (right). The results are produced with CLC transition, G *^1^* curvature estimate and max‐linear increase function.

Our method is based on an estimation of tangents and curvatures at the control points. We can offer further modeling options by allowing the user to input a constrained tangent and curvature at certain control points. Constrained curvatures are not modified during the refinement and therefore improve the locality of the method. However, the user choices, in extreme cases, could prevent us from fulfilling the desired set of mathematical properties of the curve. We show examples in Fig. 10.

## 5. Results

We implement our technique in C++ using libigl [JP∗18] for interacting with the curve and libhedra [V∗17] for visualization. Clothoid computations for solving the *G* ^1^ problem [BF15], the 3‐arcs problem [BF18b], and intersections detection [BBF20] employ a dedicated library [BF21]. Our method has a few variations, and the user can tweak the result as follows:

1. Choose either 3‐arcs clothoids or CLC‐transitions for the transition curves between consecutive control points.
2. Initial curvature estimation via circle‐ellipse local interpolation [Yuk20] or *G* ^1^‐clothoid fitting.
3. Linear or max‐linear increase function for curvature refinement.

**Figure 11:**
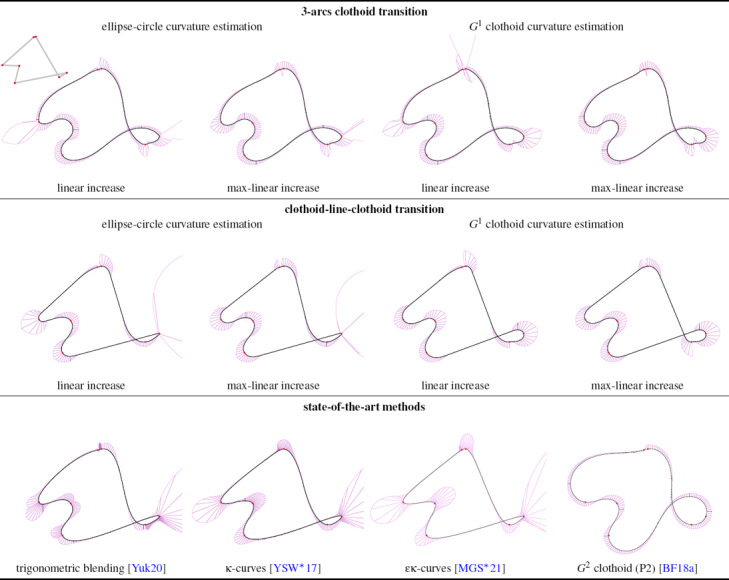
We show all the variants of our method and compare it with the state of the art. All curves are generated from the same set of control points, taken from [Yuk20]. The control polygon is shown in the inset (top left). The top row demonstrates our formulation for the 3‐arcs clothoid transition, and the middle row shows CLC transition, each with the different options in curvature estimation and increase functions for curvature refinement. The ∊κ‐curves [MGS∗21] are produced with the cubic method, α = 0.85. The purple lines represent the scaled curvature normals.

We present our results with all the possible combinations and compare with other works in Fig. 11. In the following, we discuss and compare our method with the state of the art; Table 1 summarizes the comparison using the criteria employed in [Yuk20].

**Table 1:** Comparison of curvature‐continuous splines formulations; the criteria are taken from [Yuk20]. Our method is listed in the last two columns, distinguishing between the two options for transition curves: 3‐arcs clothoids and the CLC transition. *Collinearity* refers to reproducing a straight line when three consecutive control points are collinear. *Roundness* refers to reproducing a circle or part of it.

Property	Trigonometric blending [Yuk20]	κ‐curves [YSW∗17]	∊κ‐curves [MGS∗21]	Clothoid spline [BF18a]	3‐arcs clothoid transition	CLC transition
Smoothness	*G* ^2^	mostly *G* ^2^	bounded *G* ^1^ gaps	*G* ^2^	*G* ^2^	*G* ^2^
Continuity w.r.t. *p_i_*	yes	yes	yes	no	no	curvature estimation
Local support	4 points	no	no	no	6 or 8 points	6 or 8 points
Global optimization	no	yes	yes	yes	no	no
Self‐intersections	no intersections within a curve segment					consistent w/control polygon
Monotone curvature	no	yes	yes	yes	yes	yes
Curvature extrema at *p_i_*	no	yes	yes	yes	alternate curvature	yes
Collinearity	yes	hard	hard	hard	yes	hard
Roundness	locally	no, but [YSS19] can globally		globally	locally	no
User control	no	no	curvature profile	9 objectives	directly on tangents and curvatures	

### 5.1. Discussion, comparisons and limitations

**Smoothness.** Our method produces curves with *G* ^2^ smoothness, an important property for fairness. The method does not require parameter values as input and produces arc length parameterized curves, hence no reparameterization is required to ensure *C* ^2^. The trigonometric blending functions in [Yuk20] and the clothoid splines [BF18a] achieve *G* ^2^ continuity, while κ‐curves [YSW∗17] may show *G* ^1^ discontinuity at inflection points. The ∊κ‐curves [MGS∗21] have an additional degree of freedom that can be tuned to reduce the discontinuity at inflection points. Our method produces *G* ^2^ curves, but we do not control the curvature slope, which can in certain cases give an impression of curvature discontinuity in practice (see Fig. 11). The user can manually increase the curvature at a neighboring control point to prevent it, but we do not propose an automatic correction method.

**Local support.** Locality characterizes what part of the curve is influenced by moving a control point. With trigonometric blending [Yuk20], each curve is obtained through the blending of two arcs interpolating between three consecutive points. Therefore, each curve segment between two control points has a support of 4 consecutive points. Catmull‐Rom splines [CR74] provide local support with 6 points. All previous techniques that enforce curvature extrema at control points rely on global optimization and do not have local support: κ‐curves [YSW∗17] and ∊κ‐curves [MGS∗21] optimize the whole shape to place Bézier control points, and Bertolazzi and Frego [BF18a] propose clothoid splines with curvature continuity, which only leaves two degrees of freedom, exploited to optimize a selection of optimization objectives.

The locality of our curves depends on the curvature estimation method. The curve segment *Ci* interpolating between *p_i_* and *p*_*i* + 1_ is determined by the values of (θ_*i*_, 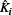) and (θ_*i* + 1_, 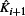). The curvature 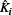 depends on *K*_*i* – 1_. If we estimate curvature solely via the hybrid ellipse‐circle method [Yuk20], then *K*_*i* – 1_ depends at most on *p*_*i* – 2_. If we use the more advanced *G* ^1^ Hermite fitting [BF15] to perform the estimation, then *K*_*i* – 1_ depends on *t*_*i* – 2_ which depends on *p*_*i* – 3_. Likewise, 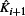 either depends on *p*_*i* + 3_ or also on *p*_*i* + 4_. Therefore our local support relies on either 6 or 8 consecutive control points, depending on the choice of the curvature estimation method. Our optional curvature refinement based on intersection detection (see Sec. 4.2.2) is performed up to a certain number of consecutive curve segments and can therefore impact locality. The supplemental video demonstrates the effects of moving control points and the local support in practice.

**Curvature profile.** Curvature is a core object of our work. We separate two cases: the monotonicity of curvature between two control points whose curvature signs are opposite, and the local extremum of curvature at control points. Blending methods [Yuk20] cannot achieve these goals. Their local interpolations pass through the control points but with different tangents and curvatures. Blending these interpolations results in a necessary curvature increase near the control points, which can be observed as two bumps in the curvature comb (see e.g. Fig. 1). In contrast, κ‐curves [YSW∗17] and ∊κ‐curves [MGS∗21] achieve the curvature goals precisely, at the cost of finite support. By elevating the degree of quadratic Bézier segments to cubic, ∊κ‐curves propose an additional parameter to tweak the curvature profile and reduce the discontinuity by increasing the curvature at control points. *G* ^2^ clothoid splines [BF18b] achieve the best curvature profile, since monotonicity is ensured even when some consecutive control points have curvature of the same sign.

Both our methods provide monotonicity of curvature between two control points of opposite curvature sign, see e.g. Fig. 1. However, the 3‐arcs clothoid method does not ensure that the maximum of curvature is reached at control points because the curvature sign can flip between two control points of the same sign. This problem does not show up with the CLC transition because its curvature profile always follows a specific pattern: linear ramp from 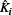 to 0, constant 0, and another ramp from 0 to 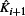. Even though our method can work solely based on the automatic estimation of the geometric features at control points, we also offer to manually set the curvature and tangents at certain control points, which variegates the possibilities in curvature profiles (see the accompanying videos). Other methods with parameters [MGS∗21, BF18b] can also vary their results, but such a variation must apply to the whole shape.

**Intersections and distance to control polygon.** All methods shown in Fig. 11 avoid self‐intersections within the individual curve segments between two successive control points. However, our CLC transition method can use curvature refinement so that the resulting curve is as close as possible to the control polygon (Proposition 3.2). As shown in Sec. 4.2.2, we can therefore produce curves that do not self‐intersect at all if the self‐intersection is not present in the control polygon, which is a stronger property.

Another point developed in [Yuk20] concerns the distance to the control polygon. Trigonometric blending based on circular interpolation [SNV00, SLY05] produces curves than can be arbitrarily far from the control polygon, while Bézier or elliptic interpolations [SZ09,Yuk20] form curves with bounded distance. We do not provide such bounds for our methods. However, we note that for the CLC method, increasing the curvature at both control points shortens the distance from the control polygon segment to the CLC curve. It is therefore possible to bound the distance from the control polygon as much as desired. Contrary to intersection detection, such a curvature refinement does not affect our local support size.

**Figure 12:**
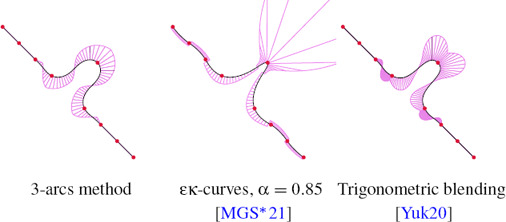
Linearity and curvature extrema at control points are conflicting objectives. The 3‐arcs clothoid transitions have local collinearity property, but the curvature extrema do not necessarily occur at control points (left). The *∊κ‐*curves [MGS∗21] attain curvature extrema at control points, but the curve shape is wavy and does not reproduce a straight line (middle). Trigonometric blending [Yuk20] ensures linearity but fails to provide curvature extrema at control points (right).

**Figure 13:**
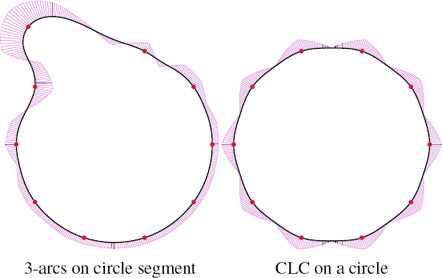
Circles can be challenging for our method. The 3‐arcs clothoid transitions have local collinearity and roundness properties, but the curvature extrema do not necessarily occur at control points (left). CLC transitions do not reproduce circles (right).

**Lines and circles.** Having curvature extrema at control points appears to be in contradiction with collinearity and roundness properties. Locally, these properties imply that if several consecutive control points are on a line or a circle, the curve reproduces this line or circle segment. Blending methods excel at this property when blending the appropriate interpolants [Yuk20,SNV00], but the constant curvature along a line or a circle conflicts with attaining a curvature extrema at control points. For κ‐curves [YSW∗17], ∊κ‐curves [MGS∗21] and CLC transitions, even global roundness fails: when all the control points are set on a circle, the resulting curve is not a circle (Fig. 13). Yan et al. [YSS19] fix this issue on κ‐curves by using a rational quadratic Bézier interpolation base. Clothoid splines [BF18b] provide global roundness, and our 3‐arcs clothoid method has local roundness, since 3‐arcs clothoids do not enforce curvature maximum at control points. This method requires 6 consecutive points set on a circular to produce a circle arc, while [Yuk20] only requires 4. Our CLC method fails if three or more consecutive control points are collinear, since the curvature refinement cannot operate on vanishing curvature. In this case, we can either break *G* ^2^ continuity or use a 3‐arcs clothoid transition, losing the placement of curvature extrema at control points. We show in Fig. 12 how our method compares to ∊κ‐curves [MGS∗21] and trigonometric blending [Yuk20]. All methods fail to simultaneously provide maximum curvature at control points and linearity, because these two objectives are contradictory.

**Figure 14:**
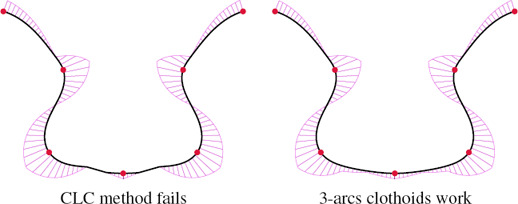
Curvature refinement for consecutive points of the same curvature sign can lead to failure of G^2^ CLC transition (left). Detecting such cases and using 3‐arcs clothoid transition solves the problem (right).

**Invalid CLC transitions.** We prove in Proposition 3.1 that if two clothoid shells have curvatures greater than a certain threshold, then there exists a CLC transition. This is a weaker statement than the affirmation that if a curvature pair *(K*_0_, *K*_1_) allows a valid CLC transition, then any pair 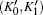 such that 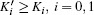, also allows it. We believe it to be true for points of alternating curvature sign (see appendix). Unfortunately, in rare cases, this does not hold for control points with curvature of same sign, as show in Fig. 14. It is possible to fix the problem in several ways: the user can always try to manually set the curvature; we can apply a second round of curvature refinement at the cost of locality; finally, we can use the 3‐arcs clothoid transition at the cost of losing curvature extrema at control points. In practice, the latter solution seems to keep the curvature extremum property because the failure of the CLC transition means that the curvature conditions are too low, which is opposite to the change of sign of the curvature in the 3‐arcs transition, as explained in Sec. 4.2.1.

**Number of control points.** Our method places curvature extrema at control points, implying that control points are positioned at salient features of the shape and should generally be sparse. Increasing the number of control points produces either wavier curves (3‐arcs method) or high localized curvature and straight lines (CLC method).

**Figure 15:**
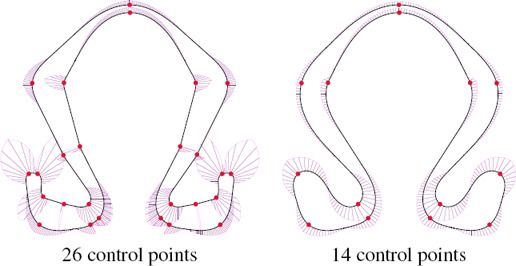
Clothoid‐line‐clothoid transition on the Ω shape using varying number of control points (taken from [BF21]).

Our method works best with points whose curvature is of alternate sign, in contrast to κ‐curves, which have discontinuities at inflection points. As shown in Fig. 15, using more control points does not necessarily produce a fairer shape.

**Continuity with respect to control points.** Continuity w.r.t. the control point locations is desirable, as it makes the modeling results more predictable. For methods with local support, any discontinuity w.r.t. moving a point *p_i_* is restricted to the neighborhood of *p_i_.* Trigonometric blending [Yuk20] and κ‐curves [YSW∗17, MGS∗21] have general continuity w.r.t. control points, while clothoid splines [BBF20] and our 3‐arcs clothoid methods do not, as our experiments reveal. The case of CLC transitions is dependent on the curvature estimation technique. With *G* ^1^ clothoid estimation, there is no general continuity for two reasons: we use hybrid interpolation if the curvature estimation via *G* ^1^ fitting is not consistent with the sign of the control polygon, leading to an abrupt change in some cases; additionally, the curvature estimation abruptly changes when the sign of the angles of the control polygon changes, which happens when a control point crosses a segment of the control polygon. However, CLC transitions with circle‐ellipse based curvature estimation are continuous w.r.t. control points, except when they are collinear. We stress that discontinuity w.r.t. control points is mitigated by the local support. Moreover, any modeling method that involves nonlinear optimization does not guarantee such continuity, since there can be multiple optima, but the collection of desirable properties generally leads to intuitive user experience overall.

### 5.2. Computation

According to our experiments on a *2.6 GHz 6‐Core Intel Core i7*, our method takes 2.3 seconds on 1000 control points and 100 evaluations per control. Thanks to the local support, the runtime is linear in the number of control points. Changing a control point modifies up to 8 piecewise clothoid transitions, hence there is no need for complete recomputation, and modeling by moving one control point can be performed in constant time. Since clothoids are based on Fresnel integrals, which are transcendental functions, they are expensive to compute. Contrary to trigonometric functions, they are not periodic and therefore prone to numerical errors for evaluation of large quantities. However, Fresnel integrals are popular and their evaluation is well studied [SRC03,VMCU16]. The accompanying videos show the realtime performance of our method, and our interactive demo code is attached to the submission as well.

## 6. Conclusion

We presented a method for planar curve interpolation that unites several properties that previously could not be achieved within one framework: *G* ^2^ smoothness, strong locality, curvature sign consistent with the control polygon, curvature extrema at control points and curvature monotonicity for alternating curvature sign, and self‐intersections entirely consistent with the control polygon. Previously, guarantees on curvature monotonicity and extrema could only be achieved using global optimization in a *G* ^2^ framework. The local support offered by our method leads to more efficient computation and intuitive control. Our key insight is that the curvature estimations at control points can be minimally increased in a coordinated, but localized manner to achieve the desired curve properties.

Several improvements on performance and quality could be attempted in future work. We would like to find a formula for the clothoid shell intersection to obtain a better understanding of self‐intersection scenarios and more efficient computation. It would be interesting to explore additional curvature refinement approaches and derive lower bounds on the feasible curvatures at control points. We did not modify the tangent estimations in this work, but tangents do have a significant influence on the final curve shape, and their optimization could be explored.

Perhaps surprisingly, there are still many unanswered questions to explore on the topic of smooth curve interpolation despite the maturity of this research field. The quantification of *aesthetics* remains elusive, although subjectively clothoids seem appealing to the eye. Clothoids have linear curvature profiles, and it would be interesting to explore other profiles, such as polynomial curvature. Another avenue for future work is the generalization to curves in 3D and tensor product surfaces.

## Acknowledgements

We thank the reviewers for their remarks. We also thank Ilya Baran for the insightful discussions and inspiration. This work was supported in party by the European Research Council (ERC) under the European Union's Horizon 2020 research and innovation programme (grant agreement No. 101003104, ERC CoG MYCLOTH).

## Appendix A: Proof of Proposition 3.1

The equation of the normal clothoid shell function is given by

(5)

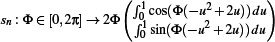




We defined the clothoid shell of the point *p_i_* with tangent angle condition θ_*i*_ and curvature κ_*i*_ through the normal shell clothoid *s_n_* via the formula


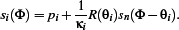




This formula is valid for Φ ∊ [θ_*i*_, 2π + θ_*i*_], but the function can be extended to Φ ∊ ℝ by taking the angle corresponding to the angle in [θ_*i*_, 2π + θ_*i*_]. Therefore *s_i_* is discontinuous on ℝ at θ_*i*_ + 2*k*π with *k* ∊ ℤ.

**Proposition 3.1.** Let *p*_0_, *p*_1_ be two distinct points and θ_0_ and θ_1_ two angles. There exist curvature bounds *K*_0_, *K*_1_, such that for any κ_0_ ≥ *K*_0_, κ_1_ ≥ *K*_1_, there exists an angle Φ such that the line segment [*s*_0_(Φ), *s*_1_(Φ + π)] makes an angle Φ with the *x*‐axis, where the starting conditions for the two clothoids are (*p*_0_, θ_0_, κ_0_) and *(p*_1_, θ_1_, κ_1_), respectively.

**Proof.** Let *s*_0_ and *s*_1_ be the clothoid shells related to points *(p*_0_, θ_0_, κ_0_) and (*p*_1_, θ_1_, κ_1_), respectively. Let Σ_0_ and Σ_1_ be the traces of curves *s*_0_ and *s*_1_ and 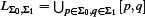 be the set of segments whose two end points are in Σ_0_ and Σ_1_, respectively. Let [Φ_*a*_, Φ_*b*_] be the set of angles between the *x*‐axis and the lines 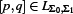. This set is simply connected and can be noted as an interval because Σ_0_ and Σ_1_ are simply connected.

We know that for all Φ in 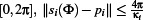. Therefore, when both curvatures κ_0_ and κ_1_ converge towards +∞, *s*_0_ and *s*1 converge uniformly towards *p*_0_ and *p*_1_ and the set [Φ_*a*_, Φ_*b*_] is actually converging to the singleton {Φ_0,1_}, which is the angle formed by the *x*‐axis and the segment [*p*_0_, *p*_1_]. We define *v*(Φ) := *s*_1_(Φ – π) – *s*_0_(Φ). Proving Proposition 3.1 is equivalent to proving that there exists Φ such that *v*(Φ) makes an angle Φ with the *x*‐axis. We distinguish between several cases:

- If Φ_0,1_ = θ_0_ = θ_1_ – π, then *v*(θ_0_) = *s*_1_(θ_1_) – *s*_0_(θ_0_) = *p*_1_ – *p*_0_ makes an angle θ_0_ with the *x*‐axis.
- Otherwise, we recall that *s_i_* is continuous except on the set of points {θ_*i*_ + 2*k*π| *k* ∊ ℤ}. We have again two cases to consider:
  – If Φ_0,1_ is not equal to θ_1_ – π or θ_0_, then there exists an ∊ > 0 such that there are *K*_0_ and *K*_1_, such that for κ_0_ > *K*_0_, κ_1_ > *K*_1_, Φ_*a*_, Φ_*b*_] ⊂ [Φ_0,1_ – ∊, Φ_0,1_ + ∊] and both θ_1_ – π and θ_0_ are not in [Φ_*a*_, Φ_*b*_]. Therefore, both *s*_0_ and *s*_1_ ○ τ_–π_ are continuous on such an interval [Φ_*a*_, Φ_*b*_] (τ_–π_ is a shift by –π). By continuity, there is Φ ∊ [Φ_*a*_, Φ_*b*_] such that *v*(Φ) makes an angle Φ with the *x*‐axis.
  – We suppose that Φ_0,1_ = θ_0_ but Φ_0,1_ ≠ θ_1_ – π (by symmetry of the problem, the case in which Φ_0,1_ = θ_1_ – π and Φ_0,1_ ≠ θ_0_ is similar). Without loss of generality, since the situation is the same up to a rotation and translation, we can consider θ_0_ = 0 = Φ_0,1_ and *p*_0_ = (0, 0). In this setting, *p*_1_ has necessarily a positive *x*‐coordinate and vanishing *y*‐coordinate.

We consider ∊ > 0 such that ∊ < θ_1_ ± π, where θ_1_ ± π is the value of the angle θ_1_ + π in [0, 2π]. Let *K*_0_, *K*_1_ be such that ∀κ_0_ > *K*_0_, κ_1_ > *K*_1_, [Φ_*a*_, Φ_*b*_] ⊂ [–∊, ∊]. We cannot conclude as before because we do not have continuity of *s*_0_ at 0. We must prove that the angle with the *x*‐axis of *v*(Φ) = *s*_1_ (Φ ± π) – *s*_0_(Φ) is positive for Φ ∊ [0, ∊]. To do so, we prove that *s*_1_(π) has a *y*‐coordinate (noted *s*_1_(π)_*y*_) that is always positive. Indeed, we know that if θ_1_ ∊ [0, π), then


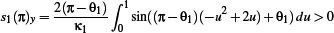




and if θ_1_ ∊ (π, 2π),


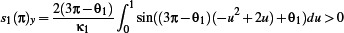

○ If θ_1_ ∊ [0, π): since *u* ∊ [0, 1] → ‐ *u* ^2^ + 2*u* is an increasing function with values in [0, 1], we know that ∀θ_1_ ∊ 0, π), ∀*u* ∊ [0, 1], 0 < θ_1_ < (π – θ_1_)(–*u* ^2^ + 2*u*) + θ_1_ ≤ π. This means that, ∀θ_1_ ∊ [0, π), ∀*u* ∊ [0, 1], sin((π – θ_1_)(–*u* ^2^ + 2*u*) + θ_1_) ≥ 0. Since the sine function is not constantly equal to 0 on [θ1, π], this shows that 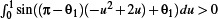 sin((π – θ_1_)(–*u* ^2^ + 2*u*) + θ_1_) *du* > 0.
○ If θ_1_ ∊ (π, 2π), let us take Θ_1_ = θ_1_ – π ∊ (0, π). We want to prove that 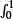 sin((3π – θ_1_)(–*u* ^2^ + 2*u*) + θ_1_) *du* > 0, i.e., 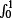 sin((2π – Θ_1_)(– *u* ^2^ + 2*u*) + Θ_1_ + π) *du* > 0, i.e., that

(6)

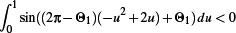

  We therefore show that:


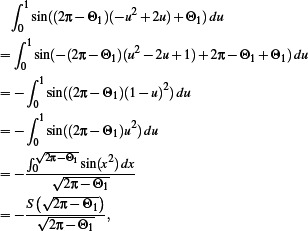

  where *S* is the Fresnel integral 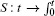 sin(*x* ^2^) *dx.* It is known that *S* is positive on 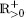, therefore we have proved the statement in (6).

Hence the angle ψ between the *x*‐axis and *v*(0) = *s*_1_ (π) – *s*_0_(0) is positive. This angle ψ is smaller than ∊ since [Φ_*a*_, Φ_*b*_] ⊂ [–∊, ∊] and *s*_1_ ○ τ_–π_ is continuous on [0, ∊**].** By continuity, there exists Φ ∊ [0, ψ] ⊂]0, ∊] such that *v*(Φ) = *s*_1_(Φ – π) – *s*_0_(Φ) makes an angle Φ with the *x*‐axis.

**Figure 16:**
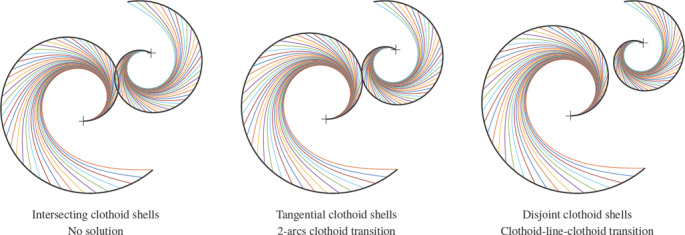
Clothoid shells (black curves) for clothoid‐line‐clothoid (CLC) transition between points with given curvatures of opposite signs. p*_0_ = (0, 0), θ_0_ = 0, κ_0_ = 1*, p*_1_ = (3, 3)*, 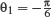 and *κ_1_* is equal to *1.5* (left), *1.655* (middle) and *2.0* (right). The function s1 has been produced with positive curvature: we are looking for the point p_e,1_ = s_1_ *(Φ)* that makes v(*Φ*) have an angle *Φ* with the x‐axis. But the second part of the CLC transition goes from p_e,1_ to p*_1_* and therefore has curvature *–κ_1_* at p*_1_*. Colored curves represent the shortest clothoids joining (p, *θ*, K) and a line of slope angle *Φ*.

**Interpretations of Proposition 3.1.** In our method, the tangent is computed based on a hybrid circular‐elliptical interpolation of three successive points *p*_*i* – 1_, *p_i_* and *p*_*i* + 1_ [Yuk20]. Therefore, the angles θ_0_ and θ_1_ are always such that the vector *p*_1_ – *p*_0_ is making a positive angle smaller than π/2 with the line (*p*_0_, θ_0_) (respectively, the vector *p*_0_ – *p*_1_ and the line (*p*_1_, π + θ_1_)). This situation is illustrated in Figure 16. We can see in this figure that three cases can happen:

1. The clothoid shells intersect twice. The angle with the *x*‐axis of *v*(Φ) therefore makes a whole round and is never equal to Φ.
2. The clothoid shells are tangent. The unique intersection point *p* is such that there exists Φ such that *s*_0_(Φ) = *s*_1_(Φ + π) = *p.* Therefore, the line part of the CLC transition is vanishes, and we have a 2‐arcs clothoid transition.
3. The clothoid shells are disjoint. Let ψ be the angle that *v*(Φ) makes with the *x*‐axis. In this situation, the difference ψ – Φ is first positive around Φ = 0 and then negative around Φ = π. Therefore there is a Φ such that ψ – Φ = 0.

Note that in the case of same‐sign curvature, the situation can be different. It is possible to find *K*_0_, *K*_1_ such that there exists a CLC transition from (*p*_0_, θ_0_, *K*_0_) to (*p*_1_, θ_1_, *K*_1_), but not every κ_0_ > *K*_0_ and κ_1_ > *K*_1_ makes the existence of a CLC transition persist (Sec. 5.1).

**Figure 17:**
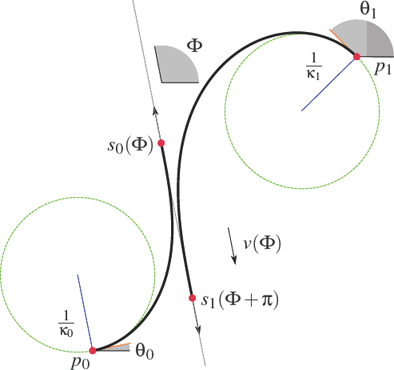
In this example, the order of the point is reversed on the transition line. This case is not considered as valid for Proposition 3.1 because v*(Φ)* makes an angle *Φ + π* with the x‐axis.

It is possible that the only Φ such that *s*_0_ (Φ) and *s*_1_(Φ + π) end on the same tangent line is not suitable for our CLC transition. In Fig. 17, the order of the points is reversed. However, *v*(Φ) makes an angle Φ + π with the *x*‐axis. This case is not considered as valid in our proof, and hence further increase of the curvature is required. Therefore, Proposition 3.1 states that there exists a *valid* CLC transition, without eluding the case of reverse order of the points on the transition line.

## Supporting information

Supplement Material
