# Supplementary material for: Smooth Interpolating Curves with Local Control and Monotone Alternating Curvature
Source: Comput Graph Forum. 2022 Oct 6;41(5):25–38. doi: 10.1111/cgf.14600 (PMC9827861; doi:10.1111/cgf.14600)
Supplement: Supplementary file 1 — Supplement Material [file CGF-41-25-s001.zip › Local-Smooth-Interpolating-MonoCurvature/extern/clothoids/docs/api-cpp/class_a00123.html]

Class BBox — Clothoids v2.0.9

### Navigation

- index
- toc
- next
- previous
- Clothoids »
- C++ API »
- Class BBox

# Class BBox¶

- Defined in File AABBtree.hxx

## Class Documentation¶

class G2lib::BBox¶
:   Class to manipulate bounding box

    Public Types

    typedef BBox const \*PtrBBox¶

    Public Functions

    inline BBox(real\_type xmin, real\_type ymin, real\_type xmax, real\_type ymax, int\_type id, int\_type ipos)¶
    :   Construct a bounding box with additional information

        Parameters
        :   - **xmin** – **[in]** x-minimimum box coordinate
            - **ymin** – **[in]** y-minimimum box coordinate
            - **xmax** – **[in]** x-maximum box coordinate
            - **ymax** – **[in]** y-maximum box coordinate
            - **id** – **[in]** identifier of the box
            - **ipos** – **[in]** ranking position of the box

    inline BBox(vector<PtrBBox> const &bboxes, int\_type id, int\_type ipos)¶
    :   Build a buonding box that cover a list of bounding box

        Parameters
        :   - **bboxes** – **[in]** list of bounding box
            - **id** – **[in]** identifier of the box
            - **ipos** – **[in]** ranking position of the box

    inline real\_type Xmin() const¶
    :   x-minimum coordinate of the bbox

    inline real\_type Ymin() const¶
    :   y-minimum coordinate of the bbox

    inline real\_type Xmax() const¶
    :   x-maximum coordinate of the bbox

    inline real\_type Ymax() const¶
    :   y-maximum coordinate of the bbox

    inline int\_type const &Id() const¶
    :   return BBOX id

    inline int\_type const &Ipos() const¶
    :   return BBOX position

    inline BBox const &operator=(BBox const &rhs)¶
    :   copy a bbox

    inline bool collision(BBox const &box) const¶
    :   detect if two bbox collide

    void join(vector<PtrBBox> const &bboxes)¶
    :   Build bbox for a list of bbox

    real\_type distance(real\_type x, real\_type y) const¶
    :   distance of the point `(x,y)` to the bbox

    real\_type maxDistance(real\_type x, real\_type y) const¶
    :   Maximum distance of the point `(x,y)` to the point of bbox

    inline void print(ostream\_type &stream) const¶
    :   Pretty print a bbox

    Friends

    *friend class* AABBtree

### Quick search

### Table of Contents

- Matlab Interface Manual
- C++ API
- MATLAB API

«
hide menu

menu
sidebar
»

### Navigation

- index
- toc
- next
- previous
- Clothoids »
- C++ API »
- Class BBox

© Copyright 2021, Enrico Bertolazzi and Marco Frego.
Created using Sphinx 4.2.0.
