# Supplementary material for: Smooth Interpolating Curves with Local Control and Monotone Alternating Curvature
Source: Comput Graph Forum. 2022 Oct 6;41(5):25–38. doi: 10.1111/cgf.14600 (PMC9827861; doi:10.1111/cgf.14600)
Supplement: Supplementary file 1 — Supplement Material [file CGF-41-25-s001.zip › Local-Smooth-Interpolating-MonoCurvature/extern/clothoids/docs/api-cpp/class_a00127.html]

Class AABBtree — Clothoids v2.0.9

### Navigation

- index
- toc
- next
- previous
- Clothoids »
- C++ API »
- Class AABBtree

# Class AABBtree¶

- Defined in File AABBtree.hxx

## Class Documentation¶

class G2lib::AABBtree¶
:   Class to build and manage an AABB tree (Axis-Aligned Bounding Box Trees)

    The class provides 2-dimensional aabb-tree construction and search for arbitrary collections of spatial objects. These tree-based indexing structures are useful when seeking to implement efficient spatial queries, reducing the complexity of intersection tests between collections of objects.

    Public Types

    typedef BBox const \*PtrBBox¶

    typedef AABBtree \*PtrAABB¶

    typedef pair<PtrBBox, PtrBBox> PairPtrBBox¶

    typedef vector<PtrBBox> VecPtrBBox¶

    typedef vector<PairPtrBBox> VecPairPtrBBox¶

    Public Functions

    AABBtree()¶
    :   Create an empty AABB tree.

    ~AABBtree()¶
    :   destroy the stored AABB tree.

    void clear()¶
    :   Initialized AABB tree.

    bool empty() const¶
    :   Check if AABB tree is empty.

    inline void bbox(real\_type &xmin, real\_type &ymin, real\_type &xmax, real\_type &ymax) const¶
    :   Get the Bounding Box of the whole AABB tree

        Parameters
        :   - **xmin** – **[in]** x-minimimum box coordinate
            - **ymin** – **[in]** y-minimimum box coordinate
            - **xmax** – **[in]** x-maximum box coordinate
            - **ymax** – **[in]** y-maximum box coordinate

    void build(vector<PtrBBox> const &bboxes)¶
    :   Build AABB tree given a list of bbox

    void print(ostream\_type &stream, int level = 0) const¶
    :   Pretty print the AABB tree

    template<typename COLLISION\_fun> inline bool collision(AABBtree const &tree, COLLISION\_fun ifun, bool swap\_tree = false) const¶
    :   Check if two AABB tree collide

        Parameters
        :   - **tree** – **[in]** an AABB tree that is used to check collision
            - **ifun** – **[in]** function the check if the contents of two bbox (curve) collide
            - **swap\_tree** – **[in]** if true exchange the tree in computation

        Returns
        :   true if the two tree collides

    void intersect(AABBtree const &tree, VecPairPtrBBox &intersectionList, bool swap\_tree = false) const¶
    :   Compute all the intersection of AABB trees.

        Parameters
        :   - **tree** – **[in]** an AABB tree that is used to check collision
            - **intersectionList** – **[out]** list of pair bbox that overlaps
            - **swap\_tree** – **[in]** if true exchange the tree in computation

    void min\_distance(real\_type x, real\_type y, VecPtrBBox &candidateList) const¶
    :   Select all the bboxes candidate to be at minimum distance.

        Parameters
        :   - **x** – **[in]** x-coordinate of the point
            - **y** – **[in]** y-coordinate of the point
            - **candidateList** – **[out]** candidate list

### Quick search

### Table of Contents

- Matlab Interface Manual
- C++ API
- MATLAB API

«
hide menu

menu
sidebar
»

### Navigation

- index
- toc
- next
- previous
- Clothoids »
- C++ API »
- Class AABBtree

© Copyright 2021, Enrico Bertolazzi and Marco Frego.
Created using Sphinx 4.2.0.
