# Supplementary material for: Smooth Interpolating Curves with Local Control and Monotone Alternating Curvature
Source: Comput Graph Forum. 2022 Oct 6;41(5):25–38. doi: 10.1111/cgf.14600 (PMC9827861; doi:10.1111/cgf.14600)
Supplement: Supplementary file 1 — Supplement Material [file CGF-41-25-s001.zip › Local-Smooth-Interpolating-MonoCurvature/extern/clothoids/docs/api-cpp/class_a00143.html]

Class CircleArc — Clothoids v2.0.9

### Navigation

- index
- toc
- next
- previous
- Clothoids »
- C++ API »
- Class CircleArc

# Class CircleArc¶

- Defined in File Circle.hxx

## Inheritance Relationships¶

### Base Type¶

- `public G2lib::BaseCurve` (Class BaseCurve)

## Class Documentation¶

class G2lib::CircleArc : public G2lib::BaseCurve¶
:   Class to manage a circle arc

    Public Functions

    inline CircleArc()¶
    :   Build an empty circle

    inline CircleArc(CircleArc const &s)¶
    :   Build a copy of an existing circle arc.

    inline explicit CircleArc(real\_type x0, real\_type y0, real\_type theta0, real\_type k, real\_type L)¶
    :   Construct a circle arc with the standard parameters.

        Parameters
        :   - **x0** – **[in]** starting position x-coordinate
            - **y0** – **[in]** starting position y-coordinate
            - **theta0** – **[in]** initial angle
            - **k** – **[in]** curvature
            - **L** – **[in]** length

    inline explicit CircleArc(LineSegment const &LS)¶
    :   Construct a circle arc from a line segment (degenerate circle).

    inline void copy(CircleArc const &c)¶
    :   Make a copy of an existing circle arc.

    explicit CircleArc(BaseCurve const &C)¶
    :   Build a circle arc from a generic curve (if possibile).

    inline CircleArc const &operator=(CircleArc const &s)¶
    :   Make a copy of an existing circle arc.

    inline void build(real\_type x0, real\_type y0, real\_type theta0, real\_type k, real\_type L)¶
    :   Construct a circle arc with the standard parameters.

        Parameters
        :   - **x0** – **[in]** starting position x-coordinate
            - **y0** – **[in]** starting position y-coordinate
            - **theta0** – **[in]** initial angle
            - **k** – **[in]** curvature
            - **L** – **[in]** length

    bool build\_G1(real\_type x0, real\_type y0, real\_type theta0, real\_type x1, real\_type y1)¶
    :   Build a circle by solving the hermite G1 problem.

        Parameters
        :   - **x0** – **[in]** starting position x-coordinate
            - **y0** – **[in]** starting position y-coordinate
            - **theta0** – **[in]** initial angle
            - **x1** – **[in]** final position x-coordinate
            - **y1** – **[in]** final position y-coordinate

        Returns
        :   true if success

    bool build\_3P(real\_type x0, real\_type y0, real\_type x1, real\_type y1, real\_type x2, real\_type y2)¶
    :   Build a circle passing by 3 points.

        Parameters
        :   - **x0** – **[in]** starting point x-coordinate
            - **y0** – **[in]** starting point y-coordinate
            - **x1** – **[in]** intermediate point x-coordinate
            - **y1** – **[in]** intermediate point y-coordinate
            - **x2** – **[in]** final point x-coordinate
            - **y2** – **[in]** final point y-coordinate

        Returns
        :   true if success

    bool bbTriangle(real\_type &x0, real\_type &y0, real\_type &x1, real\_type &y1, real\_type &x2, real\_type &y2) const¶
    :   Get the bounding box triangle (if angle variation less that pi/3).

        Parameters
        :   - **x0** – **[out]** first triangle point x-coordinate
            - **y0** – **[out]** first triangle point y-coordinate
            - **x1** – **[out]** second triangle point x-coordinate
            - **y1** – **[out]** second triangle point y-coordinate
            - **x2** – **[out]** third triangle point x-coordinate
            - **y2** – **[out]** third triangle point y-coordinate

        Returns
        :   true if success

    bool bbTriangle\_ISO(real\_type offs, real\_type &x0, real\_type &y0, real\_type &x1, real\_type &y1, real\_type &x2, real\_type &y2) const¶
    :   Get the bounding box triangle of the circle arc with offset (if angle variation less that \( \pi/3 \)).

        Parameters
        :   - **offs** – **[in]** offset
            - **x0** – **[out]** first triangle point x-coordinate
            - **y0** – **[out]** first triangle point y-coordinate
            - **x1** – **[out]** second triangle point x-coordinate
            - **y1** – **[out]** second triangle point y-coordinate
            - **x2** – **[out]** third triangle point x-coordinate
            - **y2** – **[out]** third triangle point y-coordinate

        Returns
        :   true if success

    inline bool bbTriangle\_SAE(real\_type offs, real\_type &x0, real\_type &y0, real\_type &x1, real\_type &y1, real\_type &x2, real\_type &y2) const¶
    :   Get the bounding box triangle of the circle arc with offset if angle variation less that \( \pi/3 \)).

        Parameters
        :   - **offs** – **[in]** offset
            - **x0** – **[out]** first triangle point x-coordinate
            - **y0** – **[out]** first triangle point y-coordinate
            - **x1** – **[out]** second triangle point x-coordinate
            - **y1** – **[out]** second triangle point y-coordinate
            - **x2** – **[out]** third triangle point x-coordinate
            - **y2** – **[out]** third triangle point y-coordinate

        Returns
        :   true if success

    inline bool bbTriangle(real\_type \*p0, real\_type \*p1, real\_type \*p2) const¶
    :   Get the bounding box triangle of the circle (if angle variation less that \( \pi/3 \)).

        Parameters
        :   - **p0** – **[out]** first triangle point
            - **p1** – **[out]** second triangle point
            - **p2** – **[out]** third triangle point

        Returns
        :   true if success

    inline bool bbTriangle\_ISO(real\_type offs, real\_type \*p0, real\_type \*p1, real\_type \*p2) const¶
    :   Get the bounding box triangle of the circle arc with offset (if angle variation less that \( \pi/3 \)).

        Parameters
        :   - **offs** – **[in]** offset
            - **p0** – **[out]** first triangle point
            - **p1** – **[out]** second triangle point
            - **p2** – **[out]** third triangle point

        Returns
        :   true if success

    inline bool bbTriangle\_SAE(real\_type offs, real\_type \*p0, real\_type \*p1, real\_type \*p2) const¶
    :   Get the bounding box triangle of the circle arc with offset (if angle variation less that \( \pi/3 \)).

        Parameters
        :   - **offs** – **[in]** offset
            - **p0** – **[out]** first triangle point
            - **p1** – **[out]** second triangle point
            - **p2** – **[out]** third triangle point

        Returns
        :   true if success

    inline bool bbTriangle(Triangle2D &t, real\_type ss0 = 0, real\_type ss1 = 0, int\_type icurve = 0) const¶
    :   Get the bounding box triangle list of the circle arc.

        Parameters
        :   - **t** – **[out]** the bounding triangle
            - **ss0** – **[in]** `s0` stored in the triangle class
            - **ss1** – **[in]** `s1` stored in the triangle class
            - **icurve** – **[in]** `id` stored in the triangle class

        Returns
        :   true if success

    inline bool bbTriangle\_ISO(real\_type offs, Triangle2D &t, real\_type ss0 = 0, real\_type ss1 = 0, int\_type icurve = 0) const¶
    :   Get the bounding box triangle list of the circle arc with offset.

        Parameters
        :   - **offs** – **[in]** offset
            - **t** – **[out]** the bounding triangle
            - **ss0** – **[in]** `s0` stored in the triangle class
            - **ss1** – **[in]** `s1` stored in the triangle class
            - **icurve** – **[in]** `id` stored in the triangle class

        Returns
        :   true if success

    inline bool bbTriangle\_SAE(real\_type offs, Triangle2D &t, real\_type ss0 = 0, real\_type ss1 = 0, int\_type icurve = 0) const¶
    :   Get the bounding box triangle list of the circle arc with offset.

        Parameters
        :   - **offs** – **[in]** offset
            - **t** – **[out]** the bounding triangle
            - **ss0** – **[in]** `s0` stored in the triangle class
            - **ss1** – **[in]** `s1` stored in the triangle class
            - **icurve** – **[in]** `id` stored in the triangle class

        Returns
        :   true if success

    virtual void bbTriangles(std::vector<Triangle2D> &tvec, real\_type max\_angle = Utils::m\_pi / 18, real\_type max\_size = 1e100, int\_type icurve = 0) const override¶
    :   Get the bounding box triangle list of the circle arc with offset.

        Parameters
        :   - **tvec** – **[out]** the bounding triangle list
            - **max\_angle** – **[in]** maximum angle variation admitted for all splitted segment
            - **max\_size** – **[in]** maximum size admitted for all splitted segment
            - **icurve** – **[in]** `id` stored in the triangles

    virtual void bbTriangles\_ISO(real\_type offs, std::vector<Triangle2D> &tvec, real\_type max\_angle = Utils::m\_pi / 18, real\_type max\_size = 1e100, int\_type icurve = 0) const override¶
    :   Get the bounding box triangle list of the circle arc with offset.

        Parameters
        :   - **offs** – **[in]** offset
            - **tvec** – **[out]** the bounding triangle list
            - **max\_angle** – **[in]** maximum angle variation admitted for all splitted segment
            - **max\_size** – **[in]** maximum size admitted for all splitted segment
            - **icurve** – **[in]** `id` stored in the triangles

    inline virtual void bbTriangles\_SAE(real\_type offs, std::vector<Triangle2D> &tvec, real\_type max\_angle = Utils::m\_pi / 18, real\_type max\_size = 1e100, int\_type icurve = 0) const override¶
    :   Get the bounding box triangle list of the circle arc with offset.

        Parameters
        :   - **offs** – **[in]** offset
            - **tvec** – **[out]** the bounding triangle list
            - **max\_angle** – **[in]** maximum angle variation admitted for all splitted segment
            - **max\_size** – **[in]** maximum size admitted for all splitted segment
            - **icurve** – **[in]** `id` stored in the triangles

    virtual void bbox(real\_type &xmin, real\_type &ymin, real\_type &xmax, real\_type &ymax) const override¶
    :   Compute the bounding box of the curve.

        Parameters
        :   - **xmin** – **[out]** left bottom
            - **ymin** – **[out]** left bottom
            - **xmax** – **[out]** right top
            - **ymax** – **[out]** right top

    virtual void bbox\_ISO(real\_type offs, real\_type &xmin, real\_type &ymin, real\_type &xmax, real\_type &ymax) const override¶
    :   Compute the bounding box of the curve with offset (ISO).

        Parameters
        :   - **offs** – **[in]** curve offset
            - **xmin** – **[out]** left bottom
            - **ymin** – **[out]** left bottom
            - **xmax** – **[out]** right top
            - **ymax** – **[out]** right top

    inline virtual real\_type length() const override¶
    :   The length of the curve

    inline virtual real\_type length\_ISO(real\_type offs) const override¶
    :   The length of the curve with offset (ISO)

    inline virtual real\_type thetaBegin() const override¶
    :   Initial angle of the curve.

    inline virtual real\_type kappaBegin() const override¶
    :   Initial curvature.

    inline virtual real\_type kappaEnd() const override¶
    :   Final curvature.

    inline virtual real\_type xBegin() const override¶
    :   Initial x-coordinate.

    inline virtual real\_type yBegin() const override¶
    :   Initial y-coordinate.

    inline virtual real\_type tx\_Begin() const override¶
    :   Initial tangent x-coordinate.

    inline virtual real\_type ty\_Begin() const override¶
    :   Initial tangent y-coordinate.

    inline virtual real\_type nx\_Begin\_ISO() const override¶
    :   Intial normal x-coordinate (ISO).

    inline virtual real\_type ny\_Begin\_ISO() const override¶
    :   Intial normal y-coordinate (ISO).

    inline virtual real\_type theta(real\_type s) const override¶
    :   Angle at curvilinear coodinate `s`.

    inline virtual real\_type theta\_D(real\_type) const override¶
    :   Angle derivative (curvature) at curvilinear coodinate `s`.

    inline virtual real\_type theta\_DD(real\_type) const override¶
    :   Angle second derivative (devitive of curvature) at curvilinear coodinate `s`.

    inline virtual real\_type theta\_DDD(real\_type) const override¶
    :   Angle third derivative at curvilinear coodinate `s`.

    inline virtual void evaluate(real\_type s, real\_type &th, real\_type &kappa, real\_type &x, real\_type &y) const override¶
    :   Evaluate curve at curvilinear coordinate `s`.

        Parameters
        :   - **s** – **[in]** curvilinear coordinate
            - **th** – **[out]** angle
            - **k** – **[out]** curvature
            - **x** – **[out]** x-coordinate
            - **y** – **[out]** y-coordinate

    virtual real\_type X(real\_type s) const override¶
    :   x-coordinate at curvilinear coordinate `s`.

    virtual real\_type Y(real\_type s) const override¶
    :   y-coordinate at curvilinear coordinate `s`.

    virtual real\_type X\_D(real\_type) const override¶
    :   x-coordinate derivative at curvilinear coordinate `s`.

    virtual real\_type Y\_D(real\_type) const override¶
    :   y-coordinate derivative at curvilinear coordinate `s`.

    virtual real\_type X\_DD(real\_type) const override¶
    :   x-coordinate second derivative at curvilinear coordinate `s`.

    virtual real\_type Y\_DD(real\_type) const override¶
    :   y-coordinate second derivative at curvilinear coordinate `s`.

    virtual real\_type X\_DDD(real\_type) const override¶
    :   x-coordinate third derivative at curvilinear coordinate `s`.

    virtual real\_type Y\_DDD(real\_type) const override¶
    :   y-coordinate third derivative at curvilinear coordinate `s`.

    virtual void eval(real\_type s, real\_type &x, real\_type &y) const override¶
    :   x and y-coordinate at curvilinear coordinate `s`.

    virtual void eval\_D(real\_type, real\_type &x\_D, real\_type &y\_D) const override¶
    :   x and y-coordinate derivative at curvilinear coordinate `s`.

    virtual void eval\_DD(real\_type, real\_type &x\_DD, real\_type &y\_DD) const override¶
    :   x and y-coordinate second derivative at curvilinear coordinate `s`.

    virtual void eval\_DDD(real\_type, real\_type &x\_DDD, real\_type &y\_DDD) const override¶
    :   x and y-coordinate third derivative at curvilinear coordinate `s`.

    inline virtual real\_type tx(real\_type s) const override¶
    :   Tangent x-coordinate at curvilinear coodinate `s`.

    inline virtual real\_type tx\_D(real\_type s) const override¶
    :   Tangent derivative x-coordinate at curvilinear coodinate `s`.

    inline virtual real\_type tx\_DD(real\_type s) const override¶
    :   Tangent second derivative x-coordinate at curvilinear coodinate `s`.

    inline virtual real\_type tx\_DDD(real\_type s) const override¶
    :   Tangent third derivative x-coordinate at curvilinear coodinate `s`.

    inline virtual real\_type ty(real\_type s) const override¶
    :   Tangent y-coordinate at curvilinear coodinate `s`.

    inline virtual real\_type ty\_D(real\_type s) const override¶
    :   Tangent derivative y-coordinate at curvilinear coodinate `s`.

    inline virtual real\_type ty\_DD(real\_type s) const override¶
    :   Tangent second derivative y-coordinate at curvilinear coodinate `s`.

    inline virtual real\_type ty\_DDD(real\_type s) const override¶
    :   Tangent third derivative y-coordinate at curvilinear coodinate `s`.

    virtual void tg(real\_type s, real\_type &tx, real\_type &ty) const override¶
    :   Tangent at curvilinear coodinate `s`.

    virtual void tg\_D(real\_type s, real\_type &tx\_D, real\_type &ty\_D) const override¶
    :   Tangent derivative at curvilinear coodinate `s`.

    virtual void tg\_DD(real\_type s, real\_type &tx\_DD, real\_type &ty\_DD) const override¶
    :   Tangent second derivative at curvilinear coodinate `s`.

    virtual void tg\_DDD(real\_type s, real\_type &tx\_DDD, real\_type &ty\_DDD) const override¶
    :   Tangent third derivative at curvilinear coodinate `s`.

    inline virtual void translate(real\_type tx, real\_type ty) override¶
    :   translate curve by \( (t\_x,t\_y) \)

    virtual void rotate(real\_type angle, real\_type cx, real\_type cy) override¶
    :   Rotate curve by angle \( theta \) centered at point \( (c\_x,c\_y)\).

        Parameters
        :   - **angle** – **[in]** angle \( theta \)
            - **cx** – **[in]** \( c\_x\)
            - **cy** – **[in]** \( c\_y\)

    virtual void reverse() override¶
    :   Reverse curve parameterization.

    inline virtual void changeOrigin(real\_type newx0, real\_type newy0) override¶
    :   Translate curve so that origin will be (`newx0`, `newy0`).

    virtual void scale(real\_type s) override¶
    :   Scale curve by factor `sc`.

    virtual void trim(real\_type s\_begin, real\_type s\_end) override¶
    :   Cut curve at parametrix coordinate `s_begin` and `s_end`.

    virtual int\_type closestPoint\_ISO(real\_type qx, real\_type qy, real\_type &x, real\_type &y, real\_type &s, real\_type &t, real\_type &dst) const override¶
    :   Given a point find closest point on the curve.

        Parameters
        :   - **qx** – x-coordinate of the point
            - **qy** – y-coordinate of the point
            - **x** – x-coordinate of the projected point on the curve
            - **y** – y-coordinate of the projected point on the curve
            - **s** – parameter on the curve of the projection
            - **t** – curvilinear coordinate of the point x,y (if orthogonal projection)
            - **dst** – distance point projected point

        Returns
        :   1 = point is projected orthogonal 0 = more than one projection (first returned) -1 = minimum point is not othogonal projection to curve

    virtual int\_type closestPoint\_ISO(real\_type qx, real\_type qy, real\_type offs, real\_type &x, real\_type &y, real\_type &s, real\_type &t, real\_type &dst) const override¶
    :   Given a point find closest point on the curve.

        Parameters
        :   - **qx** – x-coordinate of the point
            - **qy** – y-coordinate of the point
            - **offs** – offset of the curve
            - **x** – x-coordinate of the projected point on the curve
            - **y** – y-coordinate of the projected point on the curve
            - **s** – parameter on the curve of the projection
            - **t** – curvilinear coordinate of the point x,y (if orthogonal projection)
            - **dst** – distance point projected point

        Returns
        :   1 = point is projected orthogonal 0 = more than one projection (first returned) -1 = minimum point is not othogonal projection to curve

    inline virtual void info(ostream\_type &stream) const override¶
    :   Pretty print of the curve data.

    bool collision(CircleArc const&) const¶
    :   Detect a collision with another circle arc.

    bool collision\_ISO(real\_type offs, CircleArc const &C, real\_type offs\_obj) const¶
    :   Detect a collision with another circle arc with offset.

        Parameters
        :   - **offs** – **[in]** offset of first circle arc
            - **C** – **[in]** second circle arc
            - **offs\_obj** – **[in]** offset of second circle arc

    void intersect(CircleArc const &obj, IntersectList &ilist, bool swap\_s\_vals) const¶
    :   Intersect a circle arc with another circle arc.

        Parameters
        :   - **obj** – **[in]** second biarc
            - **ilist** – **[out]** list of the intersection (as parameter on the curves)
            - **swap\_s\_vals** – **[in]** if true store `(s2,s1)` instead of `(s1,s2)` for each intersection

    void intersect\_ISO(real\_type offs, CircleArc const &C, real\_type offs\_obj, IntersectList &ilist, bool swap\_s\_vals) const¶
    :   Intersect a circle arc with another circle arc with offset (ISO).

        Parameters
        :   - **offs** – **[in]** offset of first circle arc
            - **C** – **[in]** second circle arc
            - **offs\_obj** – **[in]** offset of second circle arc
            - **ilist** – **[out]** list of the intersection (as parameter on the curves)
            - **swap\_s\_vals** – **[in]** if true store `(s2,s1)` instead of `(s1,s2)` for each intersection

    inline real\_type sinTheta0() const¶
    :   Return \( \sin \theta\_0 \) where \( \theta\_0 \) is the initial tangent angle.

    inline real\_type cosTheta0() const¶
    :   Return \( \cos \theta\_0 \) where \( \theta\_0 \) is the initial tangent angle.

    inline real\_type curvature() const¶
    :   Return curvature of the circle arc.

    real\_type lenTolerance(real\_type tol) const¶
    :   Return the length of the arc that can approximated by a line segment.

    inline real\_type delta\_theta() const¶
    :   Return the tangent angle variation in the circle arc.

    inline real\_type thetaTotalVariation() const¶
    :   Return the absolute value of the tangent angle variation in the circle arc.

    real\_type thetaMinMax(real\_type &thMin, real\_type &thMax) const¶
    :   Minimum and maximum tangent angle.

        Parameters
        :   - **thMin** – **[out]** mimimum tangent angle
            - **thMax** – **[out]** maximum tangent angle

        Returns
        :   `thMax`-`thMin`

    inline real\_type deltaTheta() const¶
    :   Minimum and maximum tangent angle.

        Parameters
        :   - **thMin** – **[out]** mimimum tangent angle
            - **thMax** – **[out]** maximum tangent angle

        Returns
        :   `thMax`-`thMin`

    void changeCurvilinearOrigin(real\_type s0, real\_type newL)¶
    :   Change the origin of the circle arc at \( s\_0 \) and the length of the arc to \( L \).

        Parameters
        :   - **s0** – **[in]** \( s\_0 \)
            - **newL** – **[in]** \( L \)

    void center(real\_type &cx, real\_type &cy) const¶
    :   Get the center of the circle arc \( (c\_x,c\_y) \).

        Parameters
        :   - **cx** – **[in]** \( c\_x \)
            - **cy** – **[in]** \( c\_y \)

    inline real\_type ray() const¶
    :   Get the ray of the circle arc.

    void paramNURBS(int\_type &n\_knots, int\_type &n\_pnts) const¶
    :   Get the parameters to build a NURBS for the circle ars.

        Parameters
        :   - **n\_knots** – **[out]** number of knots for the NURBS
            - **n\_pnts** – **[out]** number of point of the polygon of the NURBS

    void toNURBS(real\_type \*knots, real\_type Poly[][3]) const¶
    :   Get the parameters to build a NURBS for the circle ars.

        Parameters
        :   - **knots** – **[out]** vector of the knots
            - **Poly** – **[out]** points of the polygon of the NURBS

    inline CurveType type() const¶
    :   The name of the curve type

    inline real\_type length\_SAE(real\_type offs) const¶
    :   The length of the curve with offset (SAE)

    inline void bbox\_SAE(real\_type offs, real\_type &xmin, real\_type &ymin, real\_type &xmax, real\_type &ymax) const¶
    :   Compute the bounding box of the curve (SAE).

        Parameters
        :   - **offs** – **[in]** curve offset
            - **xmin** – **[out]** left bottom
            - **ymin** – **[out]** left bottom
            - **xmax** – **[out]** right top
            - **ymax** – **[out]** right top

    inline virtual real\_type thetaEnd() const¶
    :   Final angle of the curve.

    inline virtual real\_type xEnd() const¶
    :   Final x-coordinate.

    inline virtual real\_type yEnd() const¶
    :   Final y-coordinate.

    inline virtual real\_type xBegin\_ISO(real\_type offs) const¶
    :   Initial x-coordinate with offset (ISO standard).

    inline virtual real\_type yBegin\_ISO(real\_type offs) const¶
    :   Initial y-coordinate with offset (ISO standard).

    inline virtual real\_type xEnd\_ISO(real\_type offs) const¶
    :   Final x-coordinate with offset (ISO standard).

    inline virtual real\_type yEnd\_ISO(real\_type offs) const¶
    :   Final y-coordinate with offset (ISO standard).

    inline real\_type xBegin\_SAE(real\_type offs) const¶
    :   Initial x-coordinate with offset (SAE standard).

    inline real\_type yBegin\_SAE(real\_type offs) const¶
    :   Initial y-coordinate with offset (SAE standard).

    inline real\_type xEnd\_SAE(real\_type offs) const¶
    :   Final y-coordinate with offset (SAE standard).

    inline real\_type yEnd\_SAE(real\_type offs) const¶
    :   Final y-coordinate with offset (ISO standard).

    inline virtual real\_type tx\_End() const¶
    :   Final tangent x-coordinate.

    inline virtual real\_type ty\_End() const¶
    :   Final tangent y-coordinate.

    inline virtual real\_type nx\_End\_ISO() const¶
    :   Final normal x-coordinate (ISO).

    inline virtual real\_type ny\_End\_ISO() const¶
    :   Final normal y-coordinate (ISO).

    inline real\_type nx\_Begin\_SAE() const¶
    :   Intial normal x-coordinate (SAE).

    inline real\_type ny\_Begin\_SAE() const¶
    :   Intial normal y-coordinate (SAE).

    inline real\_type nx\_End\_SAE() const¶
    :   Final normal x-coordinate (SAE).

    inline real\_type ny\_End\_SAE() const¶
    :   Intial normal y-coordinate (SAE).

    inline real\_type kappa(real\_type s) const¶
    :   Ccurvature at curvilinear coodinate `s`.

    inline real\_type kappa\_D(real\_type s) const¶
    :   Curvature derivative at curvilinear coodinate `s`.

    inline real\_type kappa\_DD(real\_type s) const¶
    :   Curvature second derivative at curvilinear coodinate `s`.

    inline real\_type nx\_ISO(real\_type s) const¶
    :   Normal x-coordinate at curvilinear coodinate `s` (ISO).

    inline real\_type nx\_ISO\_D(real\_type s) const¶
    :   Normal derivative x-coordinate at curvilinear coodinate `s` (ISO).

    inline real\_type nx\_ISO\_DD(real\_type s) const¶
    :   Normal second derivative x-coordinate at curvilinear coodinate `s` (ISO).

    inline real\_type nx\_ISO\_DDD(real\_type s) const¶
    :   Normal third derivative x-coordinate at curvilinear coodinate `s` (ISO).

    inline real\_type ny\_ISO(real\_type s) const¶
    :   Normal y-coordinate at curvilinear coodinate `s` (ISO).

    inline real\_type ny\_ISO\_D(real\_type s) const¶
    :   Normal derivative y-coordinate at curvilinear coodinate `s` (ISO).

    inline real\_type ny\_ISO\_DD(real\_type s) const¶
    :   Normal second derivative y-coordinate at curvilinear coodinate `s` (ISO).

    inline real\_type ny\_ISO\_DDD(real\_type s) const¶
    :   Normal third derivative y-coordinate at curvilinear coodinate `s` (ISO).

    inline real\_type nx\_SAE(real\_type s) const¶
    :   Normal x-coordinate at curvilinear coodinate `s` (SAE).

    inline real\_type nx\_SAE\_D(real\_type s) const¶
    :   Normal derivative x-coordinate at curvilinear coodinate `s` (SAE).

    inline real\_type nx\_SAE\_DD(real\_type s) const¶
    :   Normal second derivative x-coordinate at curvilinear coodinate `s` (SAE).

    inline real\_type nx\_SAE\_DDD(real\_type s) const¶
    :   Normal third derivative x-coordinate at curvilinear coodinate `s` (SAE).

    inline real\_type ny\_SAE(real\_type s) const¶
    :   Normal y-coordinate at curvilinear coodinate `s` (ISO)

    inline real\_type ny\_SAE\_D(real\_type s) const¶
    :   Normal derivative y-coordinate at curvilinear coodinate `s` (SAE).

    inline real\_type ny\_SAE\_DD(real\_type s) const¶
    :   Normal second derivative x-coordinate at curvilinear coodinate `s` (SAE).

    inline real\_type ny\_SAE\_DDD(real\_type s) const¶
    :   Normal third derivative y-coordinate at curvilinear coodinate `s` (SAE).

    inline void nor\_ISO(real\_type s, real\_type &nx, real\_type &ny) const¶
    :   Normal at curvilinear coodinate `s` (ISO).

    inline void nor\_ISO\_D(real\_type s, real\_type &nx\_D, real\_type &ny\_D) const¶
    :   Normal derivative at curvilinear coodinate `s` (ISO).

    inline void nor\_ISO\_DD(real\_type s, real\_type &nx\_DD, real\_type &ny\_DD) const¶
    :   Normal second derivative at curvilinear coodinate `s` (ISO).

    inline void nor\_ISO\_DDD(real\_type s, real\_type &nx\_DDD, real\_type &ny\_DDD) const¶
    :   Normal third derivative at curvilinear coodinate `s` (ISO).

    inline void nor\_SAE(real\_type s, real\_type &nx, real\_type &ny) const¶
    :   Normal at curvilinear coodinate `s` (SAE).

    inline void nor\_SAE\_D(real\_type s, real\_type &nx\_D, real\_type &ny\_D) const¶
    :   Normal derivative at curvilinear coodinate `s` (SAE).

    inline void nor\_SAE\_DD(real\_type s, real\_type &nx\_DD, real\_type &ny\_DD) const¶
    :   Normal second derivative at curvilinear coodinate `s` (SAE).

    inline void nor\_SAE\_DDD(real\_type s, real\_type &nx\_DDD, real\_type &ny\_DDD) const¶
    :   Normal third at curvilinear coodinate `s` (SAE).

    inline virtual void evaluate\_ISO(real\_type s, real\_type offs, real\_type &th, real\_type &k, real\_type &x, real\_type &y) const¶
    :   Evaluate curve with offset at curvilinear coordinate `s` (ISO).

        Parameters
        :   - **s** – **[in]** curvilinear coordinate
            - **offs** – **[in]** offset
            - **th** – **[out]** angle
            - **k** – **[out]** curvature
            - **x** – **[out]** x-coordinate
            - **y** – **[out]** y-coordinate

    inline virtual void evaluate\_SAE(real\_type s, real\_type offs, real\_type &th, real\_type &k, real\_type &x, real\_type &y) const¶
    :   Evaluate curve with offset at curvilinear coordinate `s` (SAE).

        Parameters
        :   - **s** – **[in]** curvilinear coordinate
            - **offs** – **[in]** offset
            - **th** – **[out]** angle
            - **k** – **[out]** curvature
            - **x** – **[out]** x-coordinate
            - **y** – **[out]** y-coordinate

    virtual real\_type X\_ISO(real\_type s, real\_type offs) const¶
    :   x-coordinate at curvilinear coordinate `s` with offset `offs` (ISO).

    virtual real\_type Y\_ISO(real\_type s, real\_type offs) const¶
    :   y-coordinate at curvilinear coordinate `s` with offset `offs` (ISO).

    virtual real\_type X\_ISO\_D(real\_type s, real\_type offs) const¶
    :   x-coordinate derivative at curvilinear coordinate `s` with offset `offs` (ISO).

    virtual real\_type Y\_ISO\_D(real\_type s, real\_type offs) const¶
    :   y-coordinate derivative at curvilinear coordinate `s` with offset `offs` (ISO).

    virtual real\_type X\_ISO\_DD(real\_type s, real\_type offs) const¶
    :   x-coordinate second derivative at curvilinear coordinate `s` with offset `offs` (ISO).

    virtual real\_type Y\_ISO\_DD(real\_type s, real\_type offs) const¶
    :   y-coordinate second derivative at curvilinear coordinate `s` with offset `offs` (ISO).

    virtual real\_type X\_ISO\_DDD(real\_type s, real\_type offs) const¶
    :   x-coordinate third derivative at curvilinear coordinate `s` with offset `offs` (ISO).

    virtual real\_type Y\_ISO\_DDD(real\_type s, real\_type offs) const¶
    :   y-coordinate third derivative at curvilinear coordinate `s` with offset `offs` (ISO).

    inline real\_type X\_SAE(real\_type s, real\_type offs) const¶
    :   x-coordinate at curvilinear coordinate `s` with offset `offs` (SAE).

    inline real\_type Y\_SAE(real\_type s, real\_type offs) const¶
    :   y-coordinate at curvilinear coordinate `s` with offset `offs` (SAE).

    inline real\_type X\_SAE\_D(real\_type s, real\_type offs) const¶
    :   x-coordinate derivative at curvilinear coordinate `s` with offset `offs` (SAE).

    inline real\_type Y\_SAE\_D(real\_type s, real\_type offs) const¶
    :   y-coordinate derivative at curvilinear coordinate `s` with offset `offs` (SAE).

    inline real\_type X\_SAE\_DD(real\_type s, real\_type offs) const¶
    :   x-coordinate second derivative at curvilinear coordinate `s` with offset `offs` (SAE).

    inline real\_type Y\_SAE\_DD(real\_type s, real\_type offs) const¶
    :   y-coordinate second derivative at curvilinear coordinate `s` with offset `offs` (SAE).

    inline real\_type X\_SAE\_DDD(real\_type s, real\_type offs) const¶
    :   x-coordinate third derivative at curvilinear coordinate `s` with offset `offs` (SAE).

    inline real\_type Y\_SAE\_DDD(real\_type s, real\_type offs) const¶
    :   y-coordinate third derivative at curvilinear coordinate `s` with offset `offs` (SAE).

    virtual void eval\_ISO(real\_type s, real\_type offs, real\_type &x, real\_type &y) const¶
    :   Compute curve at position `s` with offset `offs` (ISO).

        Parameters
        :   - **s** – **[in]** parameter on the curve
            - **offs** – **[in]** offset of the curve
            - **x** – **[out]** coordinate
            - **y** – **[out]** coordinate

    inline void eval\_SAE(real\_type s, real\_type offs, real\_type &x, real\_type &y) const¶
    :   Compute curve at position `s` with offset `offs` (SAE).

        Parameters
        :   - **s** – **[in]** parameter on the curve
            - **offs** – **[in]** offset of the curve
            - **x** – **[out]** coordinate
            - **y** – **[out]** coordinate

    virtual void eval\_ISO\_D(real\_type s, real\_type offs, real\_type &x\_D, real\_type &y\_D) const¶
    :   Compute derivative curve at position `s` with offset `offs` (ISO).

        Parameters
        :   - **s** – **[in]** parameter on the curve
            - **offs** – **[in]** offset of the curve
            - **x\_D** – **[out]** x-coordinate
            - **y\_D** – **[out]** y-coordinate

    inline void eval\_SAE\_D(real\_type s, real\_type offs, real\_type &x\_D, real\_type &y\_D) const¶
    :   Compute derivative curve at position `s` with offset `offs` (SAE).

        Parameters
        :   - **s** – **[in]** parameter on the curve
            - **offs** – **[in]** offset of the curve
            - **x\_D** – **[out]** x-coordinate first derivative
            - **y\_D** – **[out]** y-coordinate first derivative

    virtual void eval\_ISO\_DD(real\_type s, real\_type offs, real\_type &x\_DD, real\_type &y\_DD) const¶
    :   Compute second derivative curve at position `s` with offset `offs` (ISO).

        Parameters
        :   - **s** – **[in]** parameter on the curve
            - **offs** – **[in]** offset of the curve
            - **x\_DD** – **[out]** x-coordinate second derivative
            - **y\_DD** – **[out]** y-coordinate second derivative

    inline void eval\_SAE\_DD(real\_type s, real\_type offs, real\_type &x\_DD, real\_type &y\_DD) const¶
    :   Compute second derivative curve at position `s` with offset `offs` (SAE).

        Parameters
        :   - **s** – **[in]** parameter on the curve
            - **offs** – **[in]** offset of the curve
            - **x\_DD** – **[out]** x-coordinate second derivative
            - **y\_DD** – **[out]** y-coordinate second derivative

    virtual void eval\_ISO\_DDD(real\_type s, real\_type offs, real\_type &x\_DDD, real\_type &y\_DDD) const¶
    :   Compute third derivative curve at position `s` with offset `offs` (ISO).

        Parameters
        :   - **s** – **[in]** parameter on the curve
            - **offs** – **[in]** offset of the curve
            - **x\_DDD** – **[out]** x-coordinate third derivative
            - **y\_DDD** – **[out]** y-coordinate third derivative

    inline void eval\_SAE\_DDD(real\_type s, real\_type offs, real\_type &x\_DDD, real\_type &y\_DDD) const¶
    :   Compute third derivative curve at position `s` with offset `offs` (SAE).

        Parameters
        :   - **s** – **[in]** parameter on the curve
            - **offs** – **[in]** offset of the curve
            - **x\_DDD** – **[out]** x-coordinate third derivative
            - **y\_DDD** – **[out]** y-coordinate third derivative

    inline bool collision(BaseCurve const &C) const¶
    :   Check collision with another curve.

    inline bool collision\_ISO(real\_type offs, BaseCurve const &C, real\_type offs\_C) const¶
    :   Check collision with another curve with offset (ISO).

        Parameters
        :   - **offs** – **[in]** curve offset
            - **C** – **[in]** second curve to check collision
            - **offs\_C** – **[in]** curve offset of the second curve

        Returns
        :   true if collision is detected

    inline bool collision\_SAE(real\_type offs, BaseCurve const &C, real\_type offs\_C) const¶
    :   Check collision with another curve with offset (SAE).

        Parameters
        :   - **offs** – **[in]** curve offset
            - **C** – **[in]** second curve to check collision
            - **offs\_C** – **[in]** curve offset of the second curve

        Returns
        :   true if collision is detected

    inline void intersect(BaseCurve const &C, IntersectList &ilist, bool swap\_s\_vals) const¶
    :   Intersect the curve with another curve.

        Parameters
        :   - **C** – **[in]** second curve intersect
            - **ilist** – **[out]** list of the intersection (as parameter on the curves)
            - **swap\_s\_vals** – **[in]** if true store `(s2,s1)` instead of `(s1,s2)` for each intersection

    inline void intersect\_ISO(real\_type offs, BaseCurve const &C, real\_type offs\_C, IntersectList &ilist, bool swap\_s\_vals) const¶
    :   Intersect the curve with another curve with offset (ISO)

        Parameters
        :   - **offs** – **[in]** offset first curve
            - **C** – **[in]** second curve intersect
            - **offs\_C** – **[in]** offset second curve
            - **ilist** – **[out]** list of the intersection (as parameter on the curves)
            - **swap\_s\_vals** – **[in]** if true store `(s2,s1)` instead of `(s1,s2)` for each intersection

    inline void intersect\_SAE(real\_type offs, BaseCurve const &C, real\_type offs\_C, IntersectList &ilist, bool swap\_s\_vals) const¶
    :   Intersect the curve with another curve with offset (SAE).

        Parameters
        :   - **offs** – **[in]** offset first curve
            - **C** – **[in]** second curve intersect
            - **offs\_C** – **[in]** offset second curve
            - **ilist** – **[out]** list of the intersection (as parameter on the curves)
            - **swap\_s\_vals** – **[in]** if true store `(s2,s1)` instead of `(s1,s2)` for each intersection

    inline int\_type closestPoint\_SAE(real\_type qx, real\_type qy, real\_type &x, real\_type &y, real\_type &s, real\_type &t, real\_type &dst) const¶
    :   Given a point find closest point on the curve.

        Parameters
        :   - **qx** – x-coordinate of the point
            - **qy** – y-coordinate of the point
            - **x** – x-coordinate of the projected point on the curve
            - **y** – y-coordinate of the projected point on the curve
            - **s** – parameter on the curve of the projection
            - **t** – curvilinear coordinate of the point x,y (if orthogonal projection)
            - **dst** – distance point projected point

        Returns
        :   1 = point is projected orthogonal 0 = more than one projection (first returned) -1 = minimum point is not othogonal projection to curve

    inline int\_type closestPoint\_SAE(real\_type qx, real\_type qy, real\_type offs, real\_type &x, real\_type &y, real\_type &s, real\_type &t, real\_type &dst) const¶
    :   Given a point find closest point on the curve.

        Parameters
        :   - **qx** – x-coordinate of the point
            - **qy** – y-coordinate of the point
            - **offs** – offset of the curve
            - **x** – x-coordinate of the projected point on the curve
            - **y** – y-coordinate of the projected point on the curve
            - **s** – parameter on the curve of the projection
            - **t** – curvilinear coordinate of the point x,y (if orthogonal projection)
            - **dst** – distance point projected point

        Returns
        :   1 = point is projected orthogonal 0 = more than one projection (first returned) -1 = minimum point is not othogonal projection to curve

    inline virtual real\_type distance(real\_type qx, real\_type qy) const¶
    :   Compute the distance between a point \( q=(q\_x,q\_y) \) and the curve.

        Parameters
        :   - **qx** – **[in]** component \( q\_x \)
            - **qy** – **[in]** component \( q\_y \)

        Returns
        :   the computed distance

    inline real\_type distance\_ISO(real\_type qx, real\_type qy, real\_type offs) const¶
    :   Compute the distance between a point \( q=(q\_x,q\_y) \) and the curve with offset (ISO).

        Parameters
        :   - **qx** – **[in]** component \( q\_x \)
            - **qy** – **[in]** component \( q\_y \)
            - **offs** – **[in]** offset of the curve

        Returns
        :   the computed distance

    inline real\_type distance\_SAE(real\_type qx, real\_type qy, real\_type offs) const¶
    :   Compute the distance between a point \( q=(q\_x,q\_y) \) and the curve with offset (SAE).

        Parameters
        :   - **qx** – **[in]** component \( q\_x \)
            - **qy** – **[in]** component \( q\_y \)
            - **offs** – **[in]** offset of the curve

        Returns
        :   the computed distance

    inline bool findST\_ISO(real\_type x, real\_type y, real\_type &s, real\_type &t) const¶
    :   Find the curvilinear coordinate of point \( P=(x,y) \) respect to the curve (ISO), i.e.

        \[ P = C(s)+N(s)t \]

        where \( C(s) \) is the curve position respect to the curvilinear coordinates and \( C(s) \) is the normal at the point \( C(s) \).

        Parameters
        :   - **x** – **[in]** component \( x \)
            - **y** – **[in]** component \( y \)
            - **s** – **[out]** curvilinear coordinate
            - **t** – **[out]** offset respect to the curve of \( (x,y) \)

        Returns
        :   true if the coordinate are found

    inline bool findST\_SAE(real\_type x, real\_type y, real\_type &s, real\_type &t) const¶
    :   Find the curvilinear coordinate of point \( (x,y) \) respect to the curve (SAE), i.e.

        \[ P = C(s)+N(s)t \]

        where \( C(s) \) is the curve position respect to the curvilinear coordinates and \( C(s) \) is the normal at the point \( C(s) \).

        Parameters
        :   - **x** – **[in]** component \( x \)
            - **y** – **[in]** component \( y \)
            - **s** – **[out]** curvilinear coordinate
            - **t** – **[out]** offset respect to the curve of \( (x,y) \)

        Returns
        :   true if the coordinate are found

    Friends

    *friend class* ClothoidCurve

    friend ostream\_type &operator<<(ostream\_type &stream, CircleArc const &c)¶
    :   Pretty print circle arc.

### Quick search

### Table of Contents

- Matlab Interface Manual
- C++ API
- MATLAB API

«
hide menu

menu
sidebar
»

### Navigation

- index
- toc
- next
- previous
- Clothoids »
- C++ API »
- Class CircleArc

© Copyright 2021, Enrico Bertolazzi and Marco Frego.
Created using Sphinx 4.2.0.
