# Supplementary material for: Smooth Interpolating Curves with Local Control and Monotone Alternating Curvature
Source: Comput Graph Forum. 2022 Oct 6;41(5):25–38. doi: 10.1111/cgf.14600 (PMC9827861; doi:10.1111/cgf.14600)
Supplement: Supplementary file 1 — Supplement Material [file CGF-41-25-s001.zip › Local-Smooth-Interpolating-MonoCurvature/extern/clothoids/docs/api-cpp/class_a00151.html]

Class AsyPlot — Clothoids v2.0.9

### Navigation

- index
- toc
- next
- previous
- Clothoids »
- C++ API »
- Class AsyPlot

# Class AsyPlot¶

- Defined in File ClothoidAsyPlot.hxx

## Class Documentation¶

class G2lib::AsyPlot¶
:   Public Functions

    AsyPlot(string filename, bool showAxes)¶

    ~AsyPlot()¶

    void drawClothoid(ClothoidCurve const &c, std::string const &penna = ~~"black"~~, real\_type offset = 0) const¶

    void dot(real\_type x, real\_type y, string const &penna = ~~"black"~~) const¶

    void triangle(Triangle2D const &t, string const &penna = ~~"black"~~) const¶

    void drawRect(real\_type x0, real\_type y0, real\_type x1, real\_type y1, real\_type x2, real\_type y2, real\_type x3, real\_type y3, string const &penna = ~~"black"~~) const¶

    void drawLine(real\_type x0, real\_type y0, real\_type x1, real\_type y1, std::string const &penna = ~~"black"~~) const¶

    void label(string const &text, real\_type x, real\_type y, string const &placement = ~~""~~, string const &penna = ~~"black"~~) const¶

    void displayAxes(string const &labX, string const &labY, real\_type xmin, real\_type xmax, real\_type ymin, real\_type ymax) const¶

### Quick search

### Table of Contents

- Matlab Interface Manual
- C++ API
- MATLAB API

«
hide menu

menu
sidebar
»

### Navigation

- index
- toc
- next
- previous
- Clothoids »
- C++ API »
- Class AsyPlot

© Copyright 2021, Enrico Bertolazzi and Marco Frego.
Created using Sphinx 4.2.0.
