# Supplementary material for: Smooth Interpolating Curves with Local Control and Monotone Alternating Curvature
Source: Comput Graph Forum. 2022 Oct 6;41(5):25–38. doi: 10.1111/cgf.14600 (PMC9827861; doi:10.1111/cgf.14600)
Supplement: Supplementary file 1 — Supplement Material [file CGF-41-25-s001.zip › Local-Smooth-Interpolating-MonoCurvature/extern/clothoids/docs/api-cpp/class_a00159.html]

Class G2solveCLC — Clothoids v2.0.9

### Navigation

- index
- toc
- next
- previous
- Clothoids »
- C++ API »
- Class G2solveCLC

# Class G2solveCLC¶

- Defined in File ClothoidList.hxx

## Class Documentation¶

class G2lib::G2solveCLC¶
:   Construct a piecewise clothoids \( G(s) \) composed by 2 clothoid and one line segment that solve the G2 problem

    \[\begin{split} \begin{array}{ll} \textrm{endpoints:}\quad & \begin{cases} G(0) = \mathbf{p}\_0 & \\[0.5em] G(L) = \mathbf{p}\_1 & \end{cases} \\[1em] \textrm{angles:}\quad & \begin{cases} \theta(0) = \theta\_0 & \\[0.5em] \theta(L) = \theta\_1 & \end{cases} \\[1em] \textrm{curvature:}\quad & \begin{cases} \kappa(0) = \kappa\_0 & \\[0.5em] \kappa(L) = \kappa\_1 & \end{cases} \end{array} \end{split}\]

    **note**

    The solution do not exist for all the combination of points/angle/curvature

    Public Functions

    inline G2solveCLC()¶
    :   Build an empty clothoid list

    inline ~G2solveCLC()¶

    int build(real\_type x0, real\_type y0, real\_type theta0, real\_type kappa0, real\_type x1, real\_type y1, real\_type theta1, real\_type kappa1)¶
    :   Construct a piecewise clothoids \( G(s) \) composed by two clothoids and one line segment that solve the G2 problem, with data

        \[ \mathbf{p}\_0 = (x\_0,y\_0)^T, \qquad \mathbf{p}\_1 = (x\_1,y\_1)^T \]

        \[ \theta\_0, \qquad \theta\_1, \qquad \kappa\_0, \qquad \kappa\_1 \]

        Parameters
        :   - **x0** – **[in]** \( x\_0 \)
            - **y0** – **[in]** \( y\_0 \)
            - **theta0** – **[in]** \( \theta\_0 \)
            - **kappa0** – **[in]** \( \kappa\_0 \)
            - **x1** – **[in]** \( x\_1 \)
            - **y1** – **[in]** \( y\_1 \)
            - **theta1** – **[in]** \( \theta\_1 \)
            - **kappa1** – **[in]** \( \kappa\_1 \)

        Returns
        :   number of iterations of -1 if failed

    void setTolerance(real\_type tol)¶
    :   Fix tolerance for the G2 problem

    void setMaxIter(int tol)¶
    :   Fix maximum number of iteration for the G2 problem

    int solve()¶
    :   Solve the G2 problem

        Returns
        :   number of iterations of -1 if failed

    inline ClothoidCurve const &getS0() const¶
    :   Return the first clothoid of the G2 clothoid list

    inline ClothoidCurve const &getSM() const¶
    :   Return the second segment (the line) as a clothoid

    inline ClothoidCurve const &getS1() const¶
    :   Return the third clothoid of the G2 clothoid list

    void save(ostream\_type &stream) const¶

### Quick search

### Table of Contents

- Matlab Interface Manual
- C++ API
- MATLAB API

«
hide menu

menu
sidebar
»

### Navigation

- index
- toc
- next
- previous
- Clothoids »
- C++ API »
- Class G2solveCLC

© Copyright 2021, Enrico Bertolazzi and Marco Frego.
Created using Sphinx 4.2.0.
