# Supplementary material for: Smooth Interpolating Curves with Local Control and Monotone Alternating Curvature
Source: Comput Graph Forum. 2022 Oct 6;41(5):25–38. doi: 10.1111/cgf.14600 (PMC9827861; doi:10.1111/cgf.14600)
Supplement: Supplementary file 1 — Supplement Material [file CGF-41-25-s001.zip › Local-Smooth-Interpolating-MonoCurvature/extern/clothoids/docs/api-cpp/class_a00163.html]

Class G2solve3arc — Clothoids v2.0.9

### Navigation

- index
- toc
- next
- previous
- Clothoids »
- C++ API »
- Class G2solve3arc

# Class G2solve3arc¶

- Defined in File ClothoidList.hxx

## Class Documentation¶

class G2lib::G2solve3arc¶
:   Construct a piecewise clothoids \( G(s) \) composed by 3 clothoid and one line segment that solve the G2 problem

    **match**

    \[\begin{split} \begin{array}{ll} \textrm{endpoints:}\quad& \begin{cases} G(0) = \mathbf{p}\_0 & \\[0.5em] G(L) = \mathbf{p}\_1 & \end{cases} \\[1em] \textrm{angles:}\quad& \begin{cases} \theta(0) = \theta\_0 & \\[0.5em] \theta(L) = \theta\_1 & \end{cases} \\[1em] \textrm{curvature:}\quad& \begin{cases} \kappa(0) = \kappa\_0 & \\[0.5em] \kappa(L) = \kappa\_1 & \end{cases} \end{array} \end{split}\]

    **Reference**

    The solution algorithm is described in

    - **E.Bertolazzi, M.Frego**, On the G2 Hermite Interpolation Problem with clothoids Journal of Computational and Applied Mathematics, vol 341, pp. 99-116, 2018

    Public Functions

    inline G2solve3arc()¶

    inline ~G2solve3arc()¶

    void setTolerance(real\_type tol)¶
    :   Fix tolerance for the G2 problem

    void setMaxIter(int miter)¶
    :   Fix maximum number of iteration for the G2 problem

    int build(real\_type x0, real\_type y0, real\_type theta0, real\_type kappa0, real\_type x1, real\_type y1, real\_type theta1, real\_type kappa1, real\_type Dmax = 0, real\_type dmax = 0)¶
    :   Compute the 3 arc clothoid spline that fit the data

        Parameters
        :   - **x0** – **[in]** initial `x` position
            - **y0** – **[in]** initial `y` position
            - **theta0** – **[in]** initial angle
            - **kappa0** – **[in]** initial curvature
            - **x1** – **[in]** final `x` position
            - **y1** – **[in]** final `y` position
            - **theta1** – **[in]** final angle
            - **kappa1** – **[in]** final curvature
            - **Dmax** – **[in]** rough desidered maximum angle variation, if 0 computed automatically
            - **dmax** – **[in]** rough desidered maximum angle divergence from guess, if 0 computed automatically

        Returns
        :   number of iteration, -1 if fails

    int build\_fixed\_length(real\_type s0, real\_type x0, real\_type y0, real\_type theta0, real\_type kappa0, real\_type s1, real\_type x1, real\_type y1, real\_type theta1, real\_type kappa1)¶
    :   Compute the 3 arc clothoid spline that fit the data

        Parameters
        :   - **s0** – **[in]** length of the first segment
            - **x0** – **[in]** initial `x` position
            - **y0** – **[in]** initial `y` position
            - **theta0** – **[in]** initial angle
            - **kappa0** – **[in]** initial curvature
            - **s1** – **[in]** length of the last segment
            - **x1** – **[in]** final `x` position
            - **y1** – **[in]** final `y` position
            - **theta1** – **[in]** final angle
            - **kappa1** – **[in]** final curvature

        Returns
        :   number of iteration, -1 if fails

    inline ClothoidCurve const &getS0() const¶
    :   Returns
        :   get the first clothoid for the 3 arc G2 fitting

    inline ClothoidCurve const &getS1() const¶
    :   Returns
        :   get the last clothoid for the 3 arc G2 fitting

    inline ClothoidCurve const &getSM() const¶
    :   Returns
        :   get the middle clothoid for the 3 arc G2 fitting

    inline real\_type totalLength() const¶
    :   Returns
        :   get the length of the 3 arc G2 fitting

    inline real\_type thetaTotalVariation() const¶
    :   Returns
        :   get the total angle variation of the 3 arc G2 fitting

    inline real\_type curvatureTotalVariation() const¶
    :   Returns
        :   get the total curvature variation of the 3 arc G2 fitting

    inline real\_type integralCurvature2() const¶
    :   Returns
        :   get the integral of the curvature squared of the 3 arc G2 fitting

    inline real\_type integralJerk2() const¶
    :   Returns
        :   get the integral of the jerk squared of the 3 arc G2 fitting

    inline real\_type integralSnap2() const¶
    :   Returns
        :   get the integral of the snap squared of the 3 arc G2 fitting

    real\_type thetaMinMax(real\_type &thMin, real\_type &thMax) const¶
    :   Parameters
        :   - **thMin** – **[out]** minimum angle in the 3 arc G2 fitting curve
            - **thMax** – **[out]** maximum angle in the 3 arc G2 fitting curve

        Returns
        :   the difference of `thMax` and `thMin`

    inline real\_type deltaTheta() const¶
    :   Return the difference of maximum-minimum angle in the 3 arc G2 fitting curve

    real\_type curvatureMinMax(real\_type &kMin, real\_type &kMax) const¶
    :   Parameters
        :   - **kMin** – **[out]** minimum curvature in the 3 arc G2 fitting curve
            - **kMax** – **[out]** maximum curvature in the 3 arc G2 fitting curve

        Returns
        :   the difference of `kMax` and `kMin`

    real\_type theta(real\_type s) const¶
    :   Return angle as a function of curvilinear coordinate

    real\_type theta\_D(real\_type s) const¶
    :   Return angle derivative (curvature) as a function of curvilinear coordinate

    real\_type theta\_DD(real\_type s) const¶
    :   Return angle second derivative (curvature derivative) as a function of curvilinear coordinate

    real\_type theta\_DDD(real\_type s) const¶
    :   Return angle third derivative as a function of curvilinear coordinate

    real\_type X(real\_type s) const¶
    :   Return x coordinate of the3 arc clothoid as a function of curvilinear coordinate

    real\_type Y(real\_type s) const¶
    :   Return y coordinate of the3 arc clothoid as a function of curvilinear coordinate

    inline real\_type xBegin() const¶
    :   Return initial x coordinate of the 3 arc clothoid

    inline real\_type yBegin() const¶
    :   Return initial y coordinate of the 3 arc clothoid

    inline real\_type kappaBegin() const¶
    :   Return initial curvature of the 3 arc clothoid

    inline real\_type thetaBegin() const¶
    :   Return initial angle of the 3 arc clothoid

    inline real\_type xEnd() const¶
    :   Return final x coordinate of the 3 arc clothoid

    inline real\_type yEnd() const¶
    :   Return final y coordinate of the 3 arc clothoid

    inline real\_type kappaEnd() const¶
    :   Return final curvature of the 3 arc clothoid

    inline real\_type thetaEnd() const¶
    :   Return final angle of the 3 arc clothoid

    void eval(real\_type s, real\_type &theta, real\_type &kappa, real\_type &x, real\_type &y) const¶
    :   Compute parameters of 3 arc clothoid at curvilinear coordinate `s`

        Parameters
        :   - **s** – **[in]** curvilinear coordinate of where curve is computed
            - **theta** – **[out]** the curve angle
            - **kappa** – **[out]** the curve curvature
            - **x** – **[out]** the curve x-coordinate
            - **y** – **[out]** the curve y-coordinate

    void eval(real\_type s, real\_type &x, real\_type &y) const¶
    :   x and y-coordinate at curvilinear coordinate `s`

    void eval\_D(real\_type s, real\_type &x\_D, real\_type &y\_D) const¶
    :   x and y-coordinate derivative at curvilinear coordinate `s`

    void eval\_DD(real\_type s, real\_type &x\_DD, real\_type &y\_DD) const¶
    :   x and y-coordinate second derivative at curvilinear coordinate `s`

    void eval\_DDD(real\_type s, real\_type &x\_DDD, real\_type &y\_DDD) const¶
    :   x and y-coordinate third derivative at curvilinear coordinate `s`

    void eval\_ISO(real\_type s, real\_type offs, real\_type &x, real\_type &y) const¶
    :   x and y-coordinate at curvilinear coordinate `s` with offset

    void eval\_ISO\_D(real\_type s, real\_type offs, real\_type &x\_D, real\_type &y\_D) const¶
    :   x and y-coordinate derivative at curvilinear coordinate `s` with offset

    void eval\_ISO\_DD(real\_type s, real\_type offs, real\_type &x\_DD, real\_type &y\_DD) const¶
    :   x and y-coordinate second derivative at curvilinear coordinate `s` with offset

    void eval\_ISO\_DDD(real\_type s, real\_type offs, real\_type &x\_DDD, real\_type &y\_DDD) const¶
    :   x and y-coordinate third derivative at curvilinear coordinate `s` with offset

    inline void rotate(real\_type angle, real\_type cx, real\_type cy)¶
    :   Rotate curve by angle \( theta \) centered at point \( (c\_x,c\_y)\)

        Parameters
        :   - **angle** – **[in]** angle \( theta \)
            - **cx** – **[in]** \( c\_x\)
            - **cy** – **[in]** \( c\_y\)

    inline void translate(real\_type tx, real\_type ty)¶
    :   Translate curve by \( (t\_x,t\_y) \)

    inline void reverse()¶
    :   Reverse curve parameterization

    void save(ostream\_type &stream) const¶
    :   save clothoid list of a file stream

    Friends

    friend ostream\_type &operator<<(ostream\_type &stream, ClothoidCurve const &c)¶

### Quick search

### Table of Contents

- Matlab Interface Manual
- C++ API
- MATLAB API

«
hide menu

menu
sidebar
»

### Navigation

- index
- toc
- next
- previous
- Clothoids »
- C++ API »
- Class G2solve3arc

© Copyright 2021, Enrico Bertolazzi and Marco Frego.
Created using Sphinx 4.2.0.
