# Supplementary material for: Smooth Interpolating Curves with Local Control and Monotone Alternating Curvature
Source: Comput Graph Forum. 2022 Oct 6;41(5):25–38. doi: 10.1111/cgf.14600 (PMC9827861; doi:10.1111/cgf.14600)
Supplement: Supplementary file 1 — Supplement Material [file CGF-41-25-s001.zip › Local-Smooth-Interpolating-MonoCurvature/extern/clothoids/docs/api-cpp/class_a00167.html]

Class ClothoidList — Clothoids v2.0.9

### Navigation

- index
- toc
- next
- previous
- Clothoids »
- C++ API »
- Class ClothoidList

# Class ClothoidList¶

- Defined in File ClothoidList.hxx

## Inheritance Relationships¶

### Base Type¶

- `public G2lib::BaseCurve` (Class BaseCurve)

## Class Documentation¶

class G2lib::ClothoidList : public G2lib::BaseCurve¶
:   Manage a piecewise clothoids \( G(s) \) composed by n clothoids (not necessarily G2 or G1 connected)

    Public Functions

    inline ClothoidList()¶
    :   Build an empty clothoid list

    inline ~ClothoidList() override¶

    inline ClothoidList(ClothoidList const &s)¶
    :   Build a copy of an existing clothoid list

    void init()¶
    :   Initialize the clothoid list

    void reserve(int\_type n)¶
    :   Reserve memory for `n` clothoid

    void copy(ClothoidList const &L)¶
    :   Build a clothoid list copying an existing one

    inline ClothoidList const &operator=(ClothoidList const &s)¶
    :   Copy an existing clothoid list

    explicit ClothoidList(LineSegment const &LS)¶
    :   Build a clothoid from a line segment

    explicit ClothoidList(CircleArc const &C)¶
    :   Build a clothoid from a circle arc

    explicit ClothoidList(Biarc const &B)¶
    :   Build a clothoid from a biarc

    explicit ClothoidList(BiarcList const &BL)¶
    :   Build a clothoid from a list of biarc

    explicit ClothoidList(ClothoidCurve const &CL)¶
    :   Build a clothoid from a clothoid curve

    explicit ClothoidList(PolyLine const &PL)¶
    :   Build a clothoid from a list line segment

    explicit ClothoidList(BaseCurve const &C)¶
    :   Build a clothoid from a curve

    void push\_back(LineSegment const &c)¶
    :   Add a line segment to the tail of clothoid list

    void push\_back(CircleArc const &c)¶
    :   Add a circle arc to the tail of clothoid list

    void push\_back(Biarc const &c)¶
    :   Add a biarc to the tail of clothoid list

    void push\_back(BiarcList const &c)¶
    :   Add a biarc list to the tail of clothoid list

    void push\_back(ClothoidCurve const &c)¶
    :   Add a clothoid curve to the tail of clothoid list

    void push\_back(ClothoidList const &c)¶
    :   Add a clothoid list to the tail of clothoid list

    void push\_back(PolyLine const &c)¶
    :   Add a list of line segment to the tail of clothoid list

    void push\_back(real\_type kappa0, real\_type dkappa, real\_type L)¶
    :   Add a clothoid to the tail of the clothoid list.

        Parameters
        :   - **kappa0** – initial curvature
            - **dkappa** – derivative of the curvature
            - **L** – length of the segment

    void push\_back(real\_type x0, real\_type y0, real\_type theta0, real\_type kappa0, real\_type dkappa, real\_type L)¶
    :   Add a clothoid to the tail of the clothoid list. The builded clothoid is translated to the tail of the clothioid list.

        Parameters
        :   - **x0** – initial x
            - **y0** – initial y
            - **theta0** – initial angle
            - **kappa0** – initial curvature
            - **dkappa** – derivative of the curvature
            - **L** – length of the segment

    void push\_back\_G1(real\_type x1, real\_type y1, real\_type theta1)¶
    :   Add a clothoid to the tail of the clothoid list solving the G1 problem. The initial point and angle are taken from the tail of the clothoid list.

        Parameters
        :   - **x1** – final x
            - **y1** – final y
            - **theta1** – final angle

    void push\_back\_G1(real\_type x0, real\_type y0, real\_type theta0, real\_type x1, real\_type y1, real\_type theta1)¶
    :   Add a clothoid to the tail of the clothoid list solving the G1 problem. The initial point and angle are taken from the tail of the clothoid list. The builded clothoid is translated to the tail of the clothioid list.

        Parameters
        :   - **x0** – initial x
            - **y0** – initial y
            - **theta0** – initial angle
            - **x1** – final x
            - **y1** – final y
            - **theta1** – final angle

    inline bool is\_closed() const¶
    :   True if curve is closed

    inline void make\_closed()¶
    :   Set clousure flag to true

    inline void make\_open()¶
    :   Set clousure flag to false

    inline real\_type closure\_gap\_x() const¶
    :   Difference initial final point x component

    inline real\_type closure\_gap\_y() const¶
    :   Difference initial final point y component

    inline real\_type closure\_gap\_tx() const¶
    :   Difference initial final tangent x component

    inline real\_type closure\_gap\_ty() const¶
    :   Difference initial final tangent y component

    inline bool closure\_check(real\_type tol\_xy = 1e-6, real\_type tol\_tg = 1e-6) const¶
    :   check if clothoid list is closed

        Parameters
        :   - **tol\_xy** – **[in]** position tolerance
            - **tol\_tg** – **[in]** angle (tangent) tolerance

        Returns
        :   true if curve is closed

    bool build\_G1(int\_type n, real\_type const \*x, real\_type const \*y)¶
    :   Build clothoid list passing to a list of points solving a series of G1 fitting problems. The angle at points are estimated using the routine `xy_to_guess_angle`

        Parameters
        :   - **n** – **[in]** number of points
            - **x** – **[in]** x-coordinates
            - **y** – **[in]** y-coordinates

        Returns
        :   false if routine fails

    bool build\_G1(int\_type n, real\_type const \*x, real\_type const \*y, real\_type const \*theta)¶
    :   Build clothoid list passing to a list of points solving a series of G1 fitting problems.

        Parameters
        :   - **n** – **[in]** number of points
            - **x** – **[in]** x-coordinates
            - **y** – **[in]** y-coordinates
            - **theta** – **[in]** angles at the points

        Returns
        :   false if routine fails

    bool build(real\_type x0, real\_type y0, real\_type theta0, int\_type n, real\_type const \*s, real\_type const \*kappa)¶
    :   Build clothoid list with G2 continuity. The vector `s` contains the breakpoints of the curve. Between two breakpoint the curvature change linearly (is a clothoid)

        Parameters
        :   - **x0** – **[in]** initial x
            - **y0** – **[in]** initial y
            - **theta0** – **[in]** initial angle
            - **n** – **[in]** number of segments
            - **s** – **[in]** break point of the piecewise curve
            - **kappa** – **[in]** curvature at the break point

        Returns
        :   true if curve is closed

    inline bool build(real\_type x0, real\_type y0, real\_type theta0, vector<real\_type> const &s, vector<real\_type> const &kappa)¶
    :   Build clothoid list with G2 continuity. The vector `s` contains the breakpoints of the curve. Between two breakpoint the curvature change linearly (is a clothoid)

        Parameters
        :   - **x0** – **[in]** initial x
            - **y0** – **[in]** initial y
            - **theta0** – **[in]** initial angle
            - **s** – **[in]** break point of the piecewise curve
            - **kappa** – **[in]** curvature at the break point

        Returns
        :   true if curve is closed

    bool build\_raw(int\_type n, real\_type const \*x, real\_type const \*y, real\_type const \*abscissa, real\_type const \*theta, real\_type const \*kappa)¶
    :   Build clothoid listy using raw data.

        Parameters
        :   - **n** – **[in]** number of points
            - **x** – **[in]** x-coordinates
            - **y** – **[in]** y-coordinates
            - **abscissa** – **[in]** break point of the piecewise curve
            - **theta** – **[in]** angles at breakpoints
            - **kappa** – **[in]** curvature at the break point

        Returns
        :   false if fails

    inline bool build\_raw(vector<real\_type> const &x, vector<real\_type> const &y, vector<real\_type> const &abscissa, vector<real\_type> const &theta, vector<real\_type> const &kappa)¶
    :   Build clothoid listy using raw data.

        Parameters
        :   - **x** – **[in]** x-coordinates
            - **y** – **[in]** y-coordinates
            - **abscissa** – **[in]** break point of the piecewise curve
            - **theta** – **[in]** angles at breakpoints
            - **kappa** – **[in]** curvature at the break point

        Returns
        :   false if fails

    ClothoidCurve const &get(int\_type idx) const¶
    :   Get the `idx`-th clothoid of the list

    ClothoidCurve const &getAtS(real\_type s) const¶
    :   Get the `idx`-th clothoid of the list where `idx` is the clothoid at parameter `s`

    inline int\_type numSegments() const¶
    :   Return the numbber of clothoid of the list

    void wrap\_in\_range(real\_type &s) const¶
    :   The list of clothoid has total length \( L \) the parameter \( s \) us recomputed as \( s+kL\) in such a way \( s+kL\in[0,L)\) with \( k\in\mathbb{Z} \).

    int\_type findAtS(real\_type &s) const¶
    :   Find the clothoid segment whose definiton range contains `s`

    virtual real\_type length() const override¶
    :   The length of the curve

    virtual real\_type length\_ISO(real\_type offs) const override¶
    :   The length of the curve with offset (ISO)

    real\_type segment\_length(int\_type nseg) const¶
    :   Return the length of the `nseg`-th clothoid of the list

    real\_type segment\_length\_ISO(int\_type nseg, real\_type offs) const¶
    :   Return the length of the `nseg`-th clothoid of the list with offset

    inline real\_type segment\_length\_SAE(int\_type nseg, real\_type offs) const¶
    :   Return the length of the `nseg`-th clothoid of the list with offset

    virtual void bbTriangles(std::vector<Triangle2D> &tvec, real\_type max\_angle = Utils::m\_pi / 6, real\_type max\_size = 1e100, int\_type icurve = 0) const override¶
    :   Build a cover with triangles of the curve.

        Parameters
        :   - **tvec** – **[out]** list of covering triangles
            - **max\_angle** – **[out]** maximum angle variation of the curve covered by a triangle
            - **max\_size** – **[out]** maximum admissible size of the covering tirnagles
            - **icurve** – **[out]** index of the covering triangles

    virtual void bbTriangles\_ISO(real\_type offs, std::vector<Triangle2D> &tvec, real\_type max\_angle = Utils::m\_pi / 6, real\_type max\_size = 1e100, int\_type icurve = 0) const override¶
    :   Build a cover with triangles of the curve with offset (ISO).

        Parameters
        :   - **offs** – **[out]** curve offset
            - **tvec** – **[out]** list of covering triangles
            - **max\_angle** – **[out]** maximum angle variation of the curve covered by a triangle
            - **max\_size** – **[out]** maximum admissible size of the covering tirnagles
            - **icurve** – **[out]** index of the covering triangles

    inline virtual void bbTriangles\_SAE(real\_type offs, std::vector<Triangle2D> &tvec, real\_type max\_angle = Utils::m\_pi / 6, real\_type max\_size = 1e100, int\_type icurve = 0) const override¶
    :   Build a cover with triangles of the curve with offset (SAE).

        Parameters
        :   - **offs** – **[out]** curve offset
            - **tvec** – **[out]** list of covering triangles
            - **max\_angle** – **[out]** maximum angle variation of the arc covered by a triangle
            - **max\_size** – **[out]** maximum admissible size of the covering tirnagles
            - **icurve** – **[out]** index of the covering triangles

    inline virtual void bbox(real\_type &xmin, real\_type &ymin, real\_type &xmax, real\_type &ymax) const override¶
    :   Compute the bounding box of the curve.

        Parameters
        :   - **xmin** – **[out]** left bottom
            - **ymin** – **[out]** left bottom
            - **xmax** – **[out]** right top
            - **ymax** – **[out]** right top

    virtual void bbox\_ISO(real\_type offs, real\_type &xmin, real\_type &ymin, real\_type &xmax, real\_type &ymax) const override¶
    :   Compute the bounding box of the curve with offset (ISO).

        Parameters
        :   - **offs** – **[in]** curve offset
            - **xmin** – **[out]** left bottom
            - **ymin** – **[out]** left bottom
            - **xmax** – **[out]** right top
            - **ymax** – **[out]** right top

    inline virtual real\_type thetaBegin() const override¶
    :   Initial angle of the curve.

    inline virtual real\_type thetaEnd() const override¶
    :   Final angle of the curve.

    inline virtual real\_type xBegin() const override¶
    :   Initial x-coordinate.

    inline virtual real\_type yBegin() const override¶
    :   Initial y-coordinate.

    inline virtual real\_type xEnd() const override¶
    :   Final x-coordinate.

    inline virtual real\_type yEnd() const override¶
    :   Final y-coordinate.

    inline virtual real\_type xBegin\_ISO(real\_type offs) const override¶
    :   Initial x-coordinate with offset (ISO standard).

    inline virtual real\_type yBegin\_ISO(real\_type offs) const override¶
    :   Initial y-coordinate with offset (ISO standard).

    inline virtual real\_type xEnd\_ISO(real\_type offs) const override¶
    :   Final x-coordinate with offset (ISO standard).

    inline virtual real\_type yEnd\_ISO(real\_type offs) const override¶
    :   Final y-coordinate with offset (ISO standard).

    inline virtual real\_type tx\_Begin() const override¶
    :   Initial tangent x-coordinate.

    inline virtual real\_type ty\_Begin() const override¶
    :   Initial tangent y-coordinate.

    inline virtual real\_type tx\_End() const override¶
    :   Final tangent x-coordinate.

    inline virtual real\_type ty\_End() const override¶
    :   Final tangent y-coordinate.

    inline virtual real\_type nx\_Begin\_ISO() const override¶
    :   Intial normal x-coordinate (ISO).

    inline virtual real\_type ny\_Begin\_ISO() const override¶
    :   Intial normal y-coordinate (ISO).

    inline virtual real\_type nx\_End\_ISO() const override¶
    :   Final normal x-coordinate (ISO).

    inline virtual real\_type ny\_End\_ISO() const override¶
    :   Final normal y-coordinate (ISO).

    virtual real\_type theta(real\_type s) const override¶
    :   Angle at curvilinear coodinate `s`.

    virtual real\_type theta\_D(real\_type s) const override¶
    :   Angle derivative (curvature) at curvilinear coodinate `s`.

    virtual real\_type theta\_DD(real\_type s) const override¶
    :   Angle second derivative (devitive of curvature) at curvilinear coodinate `s`.

    virtual real\_type theta\_DDD(real\_type s) const override¶
    :   Angle third derivative at curvilinear coodinate `s`.

    virtual real\_type tx(real\_type s) const override¶
    :   Tangent x-coordinate at curvilinear coodinate `s`.

    virtual real\_type ty(real\_type s) const override¶
    :   Tangent y-coordinate at curvilinear coodinate `s`.

    virtual real\_type tx\_D(real\_type s) const override¶
    :   Tangent derivative x-coordinate at curvilinear coodinate `s`.

    virtual real\_type ty\_D(real\_type s) const override¶
    :   Tangent derivative y-coordinate at curvilinear coodinate `s`.

    virtual real\_type tx\_DD(real\_type s) const override¶
    :   Tangent second derivative x-coordinate at curvilinear coodinate `s`.

    virtual real\_type ty\_DD(real\_type s) const override¶
    :   Tangent second derivative y-coordinate at curvilinear coodinate `s`.

    virtual real\_type tx\_DDD(real\_type s) const override¶
    :   Tangent third derivative x-coordinate at curvilinear coodinate `s`.

    virtual real\_type ty\_DDD(real\_type s) const override¶
    :   Tangent third derivative y-coordinate at curvilinear coodinate `s`.

    virtual void tg(real\_type s, real\_type &tg\_x, real\_type &tg\_y) const override¶
    :   Tangent at curvilinear coodinate `s`.

    virtual void tg\_D(real\_type s, real\_type &tg\_x\_D, real\_type &tg\_y\_D) const override¶
    :   Tangent derivative at curvilinear coodinate `s`.

    virtual void tg\_DD(real\_type s, real\_type &tg\_x\_DD, real\_type &tg\_y\_DD) const override¶
    :   Tangent second derivative at curvilinear coodinate `s`.

    virtual void tg\_DDD(real\_type s, real\_type &tg\_x\_DDD, real\_type &tg\_y\_DDD) const override¶
    :   Tangent third derivative at curvilinear coodinate `s`.

    virtual void evaluate(real\_type s, real\_type &th, real\_type &k, real\_type &x, real\_type &y) const override¶
    :   Evaluate curve at curvilinear coordinate `s`.

        Parameters
        :   - **s** – **[in]** curvilinear coordinate
            - **th** – **[out]** angle
            - **k** – **[out]** curvature
            - **x** – **[out]** x-coordinate
            - **y** – **[out]** y-coordinate

    virtual void evaluate\_ISO(real\_type s, real\_type offs, real\_type &th, real\_type &k, real\_type &x, real\_type &y) const override¶
    :   Evaluate curve with offset at curvilinear coordinate `s` (ISO).

        Parameters
        :   - **s** – **[in]** curvilinear coordinate
            - **offs** – **[in]** offset
            - **th** – **[out]** angle
            - **k** – **[out]** curvature
            - **x** – **[out]** x-coordinate
            - **y** – **[out]** y-coordinate

    virtual real\_type X(real\_type s) const override¶
    :   x-coordinate at curvilinear coordinate `s`.

    virtual real\_type Y(real\_type s) const override¶
    :   y-coordinate at curvilinear coordinate `s`.

    virtual real\_type X\_D(real\_type s) const override¶
    :   x-coordinate derivative at curvilinear coordinate `s`.

    virtual real\_type Y\_D(real\_type s) const override¶
    :   y-coordinate derivative at curvilinear coordinate `s`.

    virtual real\_type X\_DD(real\_type s) const override¶
    :   x-coordinate second derivative at curvilinear coordinate `s`.

    virtual real\_type Y\_DD(real\_type s) const override¶
    :   y-coordinate second derivative at curvilinear coordinate `s`.

    virtual real\_type X\_DDD(real\_type s) const override¶
    :   x-coordinate third derivative at curvilinear coordinate `s`.

    virtual real\_type Y\_DDD(real\_type s) const override¶
    :   y-coordinate third derivative at curvilinear coordinate `s`.

    virtual void eval(real\_type s, real\_type &x, real\_type &y) const override¶
    :   x and y-coordinate at curvilinear coordinate `s`.

    virtual void eval\_D(real\_type s, real\_type &x\_D, real\_type &y\_D) const override¶
    :   x and y-coordinate derivative at curvilinear coordinate `s`.

    virtual void eval\_DD(real\_type s, real\_type &x\_DD, real\_type &y\_DD) const override¶
    :   x and y-coordinate second derivative at curvilinear coordinate `s`.

    virtual void eval\_DDD(real\_type s, real\_type &x\_DDD, real\_type &y\_DDD) const override¶
    :   x and y-coordinate third derivative at curvilinear coordinate `s`.

    virtual real\_type X\_ISO(real\_type s, real\_type offs) const override¶
    :   x-coordinate at curvilinear coordinate `s` with offset `offs` (ISO).

    virtual real\_type Y\_ISO(real\_type s, real\_type offs) const override¶
    :   y-coordinate at curvilinear coordinate `s` with offset `offs` (ISO).

    virtual real\_type X\_ISO\_D(real\_type s, real\_type offs) const override¶
    :   x-coordinate derivative at curvilinear coordinate `s` with offset `offs` (ISO).

    virtual real\_type Y\_ISO\_D(real\_type s, real\_type offs) const override¶
    :   y-coordinate derivative at curvilinear coordinate `s` with offset `offs` (ISO).

    virtual real\_type X\_ISO\_DD(real\_type s, real\_type offs) const override¶
    :   x-coordinate second derivative at curvilinear coordinate `s` with offset `offs` (ISO).

    virtual real\_type Y\_ISO\_DD(real\_type s, real\_type offs) const override¶
    :   y-coordinate second derivative at curvilinear coordinate `s` with offset `offs` (ISO).

    virtual real\_type X\_ISO\_DDD(real\_type s, real\_type offs) const override¶
    :   x-coordinate third derivative at curvilinear coordinate `s` with offset `offs` (ISO).

    virtual real\_type Y\_ISO\_DDD(real\_type s, real\_type offs) const override¶
    :   y-coordinate third derivative at curvilinear coordinate `s` with offset `offs` (ISO).

    virtual void eval\_ISO(real\_type s, real\_type offs, real\_type &x, real\_type &y) const override¶
    :   Compute curve at position `s` with offset `offs` (ISO).

        Parameters
        :   - **s** – **[in]** parameter on the curve
            - **offs** – **[in]** offset of the curve
            - **x** – **[out]** coordinate
            - **y** – **[out]** coordinate

    virtual void eval\_ISO\_D(real\_type s, real\_type offs, real\_type &x\_D, real\_type &y\_D) const override¶
    :   Compute derivative curve at position `s` with offset `offs` (ISO).

        Parameters
        :   - **s** – **[in]** parameter on the curve
            - **offs** – **[in]** offset of the curve
            - **x\_D** – **[out]** x-coordinate
            - **y\_D** – **[out]** y-coordinate

    virtual void eval\_ISO\_DD(real\_type s, real\_type offs, real\_type &x\_DD, real\_type &y\_DD) const override¶
    :   Compute second derivative curve at position `s` with offset `offs` (ISO).

        Parameters
        :   - **s** – **[in]** parameter on the curve
            - **offs** – **[in]** offset of the curve
            - **x\_DD** – **[out]** x-coordinate second derivative
            - **y\_DD** – **[out]** y-coordinate second derivative

    virtual void eval\_ISO\_DDD(real\_type s, real\_type offs, real\_type &x\_DDD, real\_type &y\_DDD) const override¶
    :   Compute third derivative curve at position `s` with offset `offs` (ISO).

        Parameters
        :   - **s** – **[in]** parameter on the curve
            - **offs** – **[in]** offset of the curve
            - **x\_DDD** – **[out]** x-coordinate third derivative
            - **y\_DDD** – **[out]** y-coordinate third derivative

    virtual void translate(real\_type tx, real\_type ty) override¶
    :   translate curve by \( (t\_x,t\_y) \)

    virtual void rotate(real\_type angle, real\_type cx, real\_type cy) override¶
    :   Rotate curve by angle \( theta \) centered at point \( (c\_x,c\_y)\).

        Parameters
        :   - **angle** – **[in]** angle \( theta \)
            - **cx** – **[in]** \( c\_x\)
            - **cy** – **[in]** \( c\_y\)

    virtual void scale(real\_type sc) override¶
    :   Scale curve by factor `sc`.

    virtual void reverse() override¶
    :   Reverse curve parameterization.

    virtual void changeOrigin(real\_type newx0, real\_type newy0) override¶
    :   Translate curve so that origin will be (`newx0`, `newy0`).

    virtual void trim(real\_type s\_begin, real\_type s\_end) override¶
    :   Cut curve at parametrix coordinate `s_begin` and `s_end`.

    void trim(real\_type s\_begin, real\_type s\_end, ClothoidList &newCL) const¶

    virtual int\_type closestPoint\_ISO(real\_type qx, real\_type qy, real\_type &x, real\_type &y, real\_type &s, real\_type &t, real\_type &dst) const override¶
    :   Parameters
        :   - **qx** – x-coordinate of the point
            - **qy** – y-coordinate of the point
            - **x** – x-coordinate of the projected point on the curve
            - **y** – y-coordinate of the projected point on the curve
            - **s** – parameter on the curve of the projection
            - **t** – curvilinear coordinate of the point x,y (if orthogonal projection)
            - **dst** – distance point projected point

        Returns
        :   n >= 0 point is projected orthogonal, n is the number of the segment at minimum distance

            -(n+1) minimum point is not othogonal projection to curve

    virtual int\_type closestPoint\_ISO(real\_type qx, real\_type qy, real\_type offs, real\_type &x, real\_type &y, real\_type &s, real\_type &t, real\_type &dst) const override¶
    :   Parameters
        :   - **qx** – x-coordinate of the point
            - **qy** – y-coordinate of the point
            - **offs** – offset of the curve
            - **x** – x-coordinate of the projected point on the curve
            - **y** – y-coordinate of the projected point on the curve
            - **s** – parameter on the curve of the projection
            - **t** – curvilinear coordinate of the point x,y (if orthogonal projection)
            - **dst** – distance point projected point

        Returns
        :   n > 0 point is projected orthogonal, n-1 is the number of the segment at minimum distance

            -(n+1) minimum point is not othogonal projection to curve

    int\_type closestSegment(real\_type qx, real\_type qy) const¶
    :   Parameters
        :   - **qx** – x-coordinate of the point
            - **qy** – y-coordinate of the point

        Returns
        :   the segment at minimal distance from point (qx,qy)

    int\_type closestPointInRange\_ISO(real\_type qx, real\_type qy, int\_type icurve\_begin, int\_type icurve\_end, real\_type &x, real\_type &y, real\_type &s, real\_type &t, real\_type &dst, int\_type &icurve) const¶
    :   Parameters
        :   - **qx** – x-coordinate of the point
            - **qy** – y-coordinate of the point
            - **icurve\_begin** – index of the initial segment
            - **icurve\_end** – index of the past to the last segment
            - **x** – x-coordinate of the projected point on the curve
            - **y** – y-coordinate of the projected point on the curve
            - **s** – parameter on the curve of the projection
            - **t** – curvilinear coordinate of the point x,y (if orthogonal projection)
            - **dst** – distance point projected point
            - **icurve** – number of the segment with the projected point

        Returns
        :   1 point is projected orthogonal

            0 = more than one projection (first returned)

            -1 = minimum point is not othogonal projection to curve

    inline int\_type closestPointInRange\_SAE(real\_type qx, real\_type qy, int\_type icurve\_begin, int\_type icurve\_end, real\_type &x, real\_type &y, real\_type &s, real\_type &t, real\_type &dst, int\_type &icurve) const¶
    :   Parameters
        :   - **qx** – x-coordinate of the point
            - **qy** – y-coordinate of the point
            - **icurve\_begin** – index of the initial segment
            - **icurve\_end** – index of the past to the last segment
            - **x** – x-coordinate of the projected point on the curve
            - **y** – y-coordinate of the projected point on the curve
            - **s** – parameter on the curve of the projection
            - **t** – curvilinear coordinate of the point x,y (if orthogonal projection)
            - **dst** – distance point projected point
            - **icurve** – number of the segment with the projected point

        Returns
        :   1 point is projected orthogonal

            0 = more than one projection (first returned)

            -1 = minimum point is not othogonal projection to curve

    int\_type closestPointInSRange\_ISO(real\_type qx, real\_type qy, real\_type s\_begin, real\_type s\_end, real\_type &x, real\_type &y, real\_type &s, real\_type &t, real\_type &dst, int\_type &icurve) const¶
    :   Parameters
        :   - **qx** – x-coordinate of the point
            - **qy** – y-coordinate of the point
            - **s\_begin** – initial curvilinear coordinate of the search range
            - **s\_end** – final curvilinear coordinate of the search range
            - **x** – x-coordinate of the projected point on the curve
            - **y** – y-coordinate of the projected point on the curve
            - **s** – parameter on the curve of the projection
            - **t** – curvilinear coordinate of the point x,y (if orthogonal projection)
            - **dst** – distance point projected point
            - **icurve** – number of the segment with the projected point

        Returns
        :   1 ok -1 projection failed

    inline int\_type closestPointInSRange\_SAE(real\_type qx, real\_type qy, int\_type s\_begin, int\_type s\_end, real\_type &x, real\_type &y, real\_type &s, real\_type &t, real\_type &dst, int\_type &icurve) const¶
    :   Parameters
        :   - **qx** – x-coordinate of the point
            - **qy** – y-coordinate of the point
            - **s\_begin** – initial curvilinear coordinate of the search range
            - **s\_end** – final curvilinear coordinate of the search range
            - **x** – x-coordinate of the projected point on the curve
            - **y** – y-coordinate of the projected point on the curve
            - **s** – parameter on the curve of the projection
            - **t** – curvilinear coordinate of the point x,y (if orthogonal projection)
            - **dst** – distance point projected point
            - **icurve** – number of the segment with the projected point

        Returns
        :   1 ok -1 projection failed

    inline virtual void info(ostream\_type &stream) const override¶
    :   Pretty print of the curve data.

    void getSK(real\_type \*s, real\_type \*kappa) const¶
    :   Return the clothoid list as a list of nodes and curvatures

        Parameters
        :   - **s** – **[out]** nodes
            - **kappa** – **[out]** curvature

    inline void getSK(std::vector<real\_type> &s, std::vector<real\_type> &kappa) const¶
    :   Return the clothoid list as a list of nodes and curvatures

        Parameters
        :   - **s** – **[out]** nodes
            - **kappa** – **[out]** curvature

    void getSTK(real\_type \*s, real\_type \*theta, real\_type \*kappa) const¶
    :   Return the clothoid list as a list of nodes angles and curvatures

        Parameters
        :   - **s** – **[out]** nodes
            - **theta** – **[out]** angles
            - **kappa** – **[out]** curvature

    inline void getSTK(std::vector<real\_type> &s, std::vector<real\_type> &theta, std::vector<real\_type> &kappa) const¶
    :   Return the clothoid list as a list of nodes angles and curvatures

        Parameters
        :   - **s** – **[out]** nodes
            - **theta** – **[out]** angles
            - **kappa** – **[out]** curvature

    void getXY(real\_type \*x, real\_type \*y) const¶
    :   Return the points of the clothoid list at breakpoints

        Parameters
        :   - **x** – **[out]** x-coordinates
            - **y** – **[out]** y-coordinates

    void getDeltaTheta(real\_type \*deltaTheta) const¶

    void getDeltaKappa(real\_type \*deltaKappa) const¶

    int\_type findST1(real\_type x, real\_type y, real\_type &s, real\_type &t) const¶
    :   Find parametric coordinate.

        Parameters
        :   - **x** – x-coordinate point
            - **y** – y-coordinate point
            - **s** – value \( s \)
            - **t** – value \( t \)

        Returns
        :   idx the segment with point at minimal distance, otherwise -(idx+1) if (x,y) cannot be projected orthogonally on the segment

    int\_type findST1(int\_type ibegin, int\_type iend, real\_type x, real\_type y, real\_type &s, real\_type &t) const¶
    :   Find parametric coordinate.

        Parameters
        :   - **ibegin** – initial segment to compute the distance
            - **iend** – final segment to compute the distance
            - **x** – x-coordinate point
            - **y** – y-coordinate point
            - **s** – value \( s \)
            - **t** – value \( t \)

        Returns
        :   idx the segment with point at minimal distance, otherwise -(idx+1) if (x,y) cannot be projected orthogonally on the segment

    bool collision(ClothoidList const &C) const¶
    :   Detect a collision with another clothoid list

    bool collision\_ISO(real\_type offs, ClothoidList const &CL, real\_type offs\_C) const¶
    :   Detect a collision with another clothoid list with offset

        Parameters
        :   - **offs** – **[in]** offset of first clothoid list
            - **CL** – **[in]** second clothoid list
            - **offs\_C** – **[in]** offset of second clothoid list

    inline void intersect(ClothoidList const &CL, IntersectList &ilist, bool swap\_s\_vals) const¶
    :   Intersect a clothoid list with another clothoid list

        Parameters
        :   - **CL** – **[in]** second clothoid list
            - **ilist** – **[out]** list of the intersection (as parameter on the curves)
            - **swap\_s\_vals** – **[in]** if true store `(s2,s1)` instead of `(s1,s2)` for each intersection

    void intersect\_ISO(real\_type offs, ClothoidList const &CL, real\_type offs\_obj, IntersectList &ilist, bool swap\_s\_vals) const¶
    :   Intersect a clothoid list with another clothoid list with offset (ISO)

        Parameters
        :   - **offs** – **[in]** offset of first clothoid list
            - **CL** – **[in]** second clothoid list
            - **offs\_obj** – **[in]** offset of second clothoid list
            - **ilist** – **[out]** list of the intersection (as parameter on the curves)
            - **swap\_s\_vals** – **[in]** if true store `(s2,s1)` instead of `(s1,s2)` for each intersection

    void export\_table(ostream\_type &stream) const¶
    :   Save Clothoid list to a stream

        Parameters
        :   **stream** – stream to save

    void export\_ruby(ostream\_type &stream) const¶
    :   Save Clothoid list to a stream

        Parameters
        :   **stream** – streamstream to save

    void save(ostream\_type &stream) const¶
    :   Save the clothoid list on a stream. The data is saved as follows

        ```
          # x y theta kappa
          x0 y0 theta0 kappa0
          x1 y1 theta1 kappa1
          ...
          xn yn thetan kappan
        ```

    void load(istream\_type &stream, real\_type epsi = 1e-8)¶
    :   Read the clothoid list from a stream. The data is assumed to be saved as follows

        ```
          # x y theta kappa
          x0 y0 theta0 kappa0
          x1 y1 theta1 kappa1
          ...
          xn yn thetan kappan
        ```

    inline CurveType type() const¶
    :   The name of the curve type

    inline real\_type length\_SAE(real\_type offs) const¶
    :   The length of the curve with offset (SAE)

    inline void bbox\_SAE(real\_type offs, real\_type &xmin, real\_type &ymin, real\_type &xmax, real\_type &ymax) const¶
    :   Compute the bounding box of the curve (SAE).

        Parameters
        :   - **offs** – **[in]** curve offset
            - **xmin** – **[out]** left bottom
            - **ymin** – **[out]** left bottom
            - **xmax** – **[out]** right top
            - **ymax** – **[out]** right top

    inline virtual real\_type kappaBegin() const¶
    :   Initial curvature.

    inline virtual real\_type kappaEnd() const¶
    :   Final curvature.

    inline real\_type xBegin\_SAE(real\_type offs) const¶
    :   Initial x-coordinate with offset (SAE standard).

    inline real\_type yBegin\_SAE(real\_type offs) const¶
    :   Initial y-coordinate with offset (SAE standard).

    inline real\_type xEnd\_SAE(real\_type offs) const¶
    :   Final y-coordinate with offset (SAE standard).

    inline real\_type yEnd\_SAE(real\_type offs) const¶
    :   Final y-coordinate with offset (ISO standard).

    inline real\_type nx\_Begin\_SAE() const¶
    :   Intial normal x-coordinate (SAE).

    inline real\_type ny\_Begin\_SAE() const¶
    :   Intial normal y-coordinate (SAE).

    inline real\_type nx\_End\_SAE() const¶
    :   Final normal x-coordinate (SAE).

    inline real\_type ny\_End\_SAE() const¶
    :   Intial normal y-coordinate (SAE).

    inline real\_type kappa(real\_type s) const¶
    :   Ccurvature at curvilinear coodinate `s`.

    inline real\_type kappa\_D(real\_type s) const¶
    :   Curvature derivative at curvilinear coodinate `s`.

    inline real\_type kappa\_DD(real\_type s) const¶
    :   Curvature second derivative at curvilinear coodinate `s`.

    inline real\_type nx\_ISO(real\_type s) const¶
    :   Normal x-coordinate at curvilinear coodinate `s` (ISO).

    inline real\_type nx\_ISO\_D(real\_type s) const¶
    :   Normal derivative x-coordinate at curvilinear coodinate `s` (ISO).

    inline real\_type nx\_ISO\_DD(real\_type s) const¶
    :   Normal second derivative x-coordinate at curvilinear coodinate `s` (ISO).

    inline real\_type nx\_ISO\_DDD(real\_type s) const¶
    :   Normal third derivative x-coordinate at curvilinear coodinate `s` (ISO).

    inline real\_type ny\_ISO(real\_type s) const¶
    :   Normal y-coordinate at curvilinear coodinate `s` (ISO).

    inline real\_type ny\_ISO\_D(real\_type s) const¶
    :   Normal derivative y-coordinate at curvilinear coodinate `s` (ISO).

    inline real\_type ny\_ISO\_DD(real\_type s) const¶
    :   Normal second derivative y-coordinate at curvilinear coodinate `s` (ISO).

    inline real\_type ny\_ISO\_DDD(real\_type s) const¶
    :   Normal third derivative y-coordinate at curvilinear coodinate `s` (ISO).

    inline real\_type nx\_SAE(real\_type s) const¶
    :   Normal x-coordinate at curvilinear coodinate `s` (SAE).

    inline real\_type nx\_SAE\_D(real\_type s) const¶
    :   Normal derivative x-coordinate at curvilinear coodinate `s` (SAE).

    inline real\_type nx\_SAE\_DD(real\_type s) const¶
    :   Normal second derivative x-coordinate at curvilinear coodinate `s` (SAE).

    inline real\_type nx\_SAE\_DDD(real\_type s) const¶
    :   Normal third derivative x-coordinate at curvilinear coodinate `s` (SAE).

    inline real\_type ny\_SAE(real\_type s) const¶
    :   Normal y-coordinate at curvilinear coodinate `s` (ISO)

    inline real\_type ny\_SAE\_D(real\_type s) const¶
    :   Normal derivative y-coordinate at curvilinear coodinate `s` (SAE).

    inline real\_type ny\_SAE\_DD(real\_type s) const¶
    :   Normal second derivative x-coordinate at curvilinear coodinate `s` (SAE).

    inline real\_type ny\_SAE\_DDD(real\_type s) const¶
    :   Normal third derivative y-coordinate at curvilinear coodinate `s` (SAE).

    inline void nor\_ISO(real\_type s, real\_type &nx, real\_type &ny) const¶
    :   Normal at curvilinear coodinate `s` (ISO).

    inline void nor\_ISO\_D(real\_type s, real\_type &nx\_D, real\_type &ny\_D) const¶
    :   Normal derivative at curvilinear coodinate `s` (ISO).

    inline void nor\_ISO\_DD(real\_type s, real\_type &nx\_DD, real\_type &ny\_DD) const¶
    :   Normal second derivative at curvilinear coodinate `s` (ISO).

    inline void nor\_ISO\_DDD(real\_type s, real\_type &nx\_DDD, real\_type &ny\_DDD) const¶
    :   Normal third derivative at curvilinear coodinate `s` (ISO).

    inline void nor\_SAE(real\_type s, real\_type &nx, real\_type &ny) const¶
    :   Normal at curvilinear coodinate `s` (SAE).

    inline void nor\_SAE\_D(real\_type s, real\_type &nx\_D, real\_type &ny\_D) const¶
    :   Normal derivative at curvilinear coodinate `s` (SAE).

    inline void nor\_SAE\_DD(real\_type s, real\_type &nx\_DD, real\_type &ny\_DD) const¶
    :   Normal second derivative at curvilinear coodinate `s` (SAE).

    inline void nor\_SAE\_DDD(real\_type s, real\_type &nx\_DDD, real\_type &ny\_DDD) const¶
    :   Normal third at curvilinear coodinate `s` (SAE).

    inline virtual void evaluate\_SAE(real\_type s, real\_type offs, real\_type &th, real\_type &k, real\_type &x, real\_type &y) const¶
    :   Evaluate curve with offset at curvilinear coordinate `s` (SAE).

        Parameters
        :   - **s** – **[in]** curvilinear coordinate
            - **offs** – **[in]** offset
            - **th** – **[out]** angle
            - **k** – **[out]** curvature
            - **x** – **[out]** x-coordinate
            - **y** – **[out]** y-coordinate

    inline real\_type X\_SAE(real\_type s, real\_type offs) const¶
    :   x-coordinate at curvilinear coordinate `s` with offset `offs` (SAE).

    inline real\_type Y\_SAE(real\_type s, real\_type offs) const¶
    :   y-coordinate at curvilinear coordinate `s` with offset `offs` (SAE).

    inline real\_type X\_SAE\_D(real\_type s, real\_type offs) const¶
    :   x-coordinate derivative at curvilinear coordinate `s` with offset `offs` (SAE).

    inline real\_type Y\_SAE\_D(real\_type s, real\_type offs) const¶
    :   y-coordinate derivative at curvilinear coordinate `s` with offset `offs` (SAE).

    inline real\_type X\_SAE\_DD(real\_type s, real\_type offs) const¶
    :   x-coordinate second derivative at curvilinear coordinate `s` with offset `offs` (SAE).

    inline real\_type Y\_SAE\_DD(real\_type s, real\_type offs) const¶
    :   y-coordinate second derivative at curvilinear coordinate `s` with offset `offs` (SAE).

    inline real\_type X\_SAE\_DDD(real\_type s, real\_type offs) const¶
    :   x-coordinate third derivative at curvilinear coordinate `s` with offset `offs` (SAE).

    inline real\_type Y\_SAE\_DDD(real\_type s, real\_type offs) const¶
    :   y-coordinate third derivative at curvilinear coordinate `s` with offset `offs` (SAE).

    inline void eval\_SAE(real\_type s, real\_type offs, real\_type &x, real\_type &y) const¶
    :   Compute curve at position `s` with offset `offs` (SAE).

        Parameters
        :   - **s** – **[in]** parameter on the curve
            - **offs** – **[in]** offset of the curve
            - **x** – **[out]** coordinate
            - **y** – **[out]** coordinate

    inline void eval\_SAE\_D(real\_type s, real\_type offs, real\_type &x\_D, real\_type &y\_D) const¶
    :   Compute derivative curve at position `s` with offset `offs` (SAE).

        Parameters
        :   - **s** – **[in]** parameter on the curve
            - **offs** – **[in]** offset of the curve
            - **x\_D** – **[out]** x-coordinate first derivative
            - **y\_D** – **[out]** y-coordinate first derivative

    inline void eval\_SAE\_DD(real\_type s, real\_type offs, real\_type &x\_DD, real\_type &y\_DD) const¶
    :   Compute second derivative curve at position `s` with offset `offs` (SAE).

        Parameters
        :   - **s** – **[in]** parameter on the curve
            - **offs** – **[in]** offset of the curve
            - **x\_DD** – **[out]** x-coordinate second derivative
            - **y\_DD** – **[out]** y-coordinate second derivative

    inline void eval\_SAE\_DDD(real\_type s, real\_type offs, real\_type &x\_DDD, real\_type &y\_DDD) const¶
    :   Compute third derivative curve at position `s` with offset `offs` (SAE).

        Parameters
        :   - **s** – **[in]** parameter on the curve
            - **offs** – **[in]** offset of the curve
            - **x\_DDD** – **[out]** x-coordinate third derivative
            - **y\_DDD** – **[out]** y-coordinate third derivative

    inline bool collision(BaseCurve const &C) const¶
    :   Check collision with another curve.

    inline bool collision\_ISO(real\_type offs, BaseCurve const &C, real\_type offs\_C) const¶
    :   Check collision with another curve with offset (ISO).

        Parameters
        :   - **offs** – **[in]** curve offset
            - **C** – **[in]** second curve to check collision
            - **offs\_C** – **[in]** curve offset of the second curve

        Returns
        :   true if collision is detected

    inline bool collision\_SAE(real\_type offs, BaseCurve const &C, real\_type offs\_C) const¶
    :   Check collision with another curve with offset (SAE).

        Parameters
        :   - **offs** – **[in]** curve offset
            - **C** – **[in]** second curve to check collision
            - **offs\_C** – **[in]** curve offset of the second curve

        Returns
        :   true if collision is detected

    inline void intersect(BaseCurve const &C, IntersectList &ilist, bool swap\_s\_vals) const¶
    :   Intersect the curve with another curve.

        Parameters
        :   - **C** – **[in]** second curve intersect
            - **ilist** – **[out]** list of the intersection (as parameter on the curves)
            - **swap\_s\_vals** – **[in]** if true store `(s2,s1)` instead of `(s1,s2)` for each intersection

    inline void intersect\_ISO(real\_type offs, BaseCurve const &C, real\_type offs\_C, IntersectList &ilist, bool swap\_s\_vals) const¶
    :   Intersect the curve with another curve with offset (ISO)

        Parameters
        :   - **offs** – **[in]** offset first curve
            - **C** – **[in]** second curve intersect
            - **offs\_C** – **[in]** offset second curve
            - **ilist** – **[out]** list of the intersection (as parameter on the curves)
            - **swap\_s\_vals** – **[in]** if true store `(s2,s1)` instead of `(s1,s2)` for each intersection

    inline void intersect\_SAE(real\_type offs, BaseCurve const &C, real\_type offs\_C, IntersectList &ilist, bool swap\_s\_vals) const¶
    :   Intersect the curve with another curve with offset (SAE).

        Parameters
        :   - **offs** – **[in]** offset first curve
            - **C** – **[in]** second curve intersect
            - **offs\_C** – **[in]** offset second curve
            - **ilist** – **[out]** list of the intersection (as parameter on the curves)
            - **swap\_s\_vals** – **[in]** if true store `(s2,s1)` instead of `(s1,s2)` for each intersection

    inline int\_type closestPoint\_SAE(real\_type qx, real\_type qy, real\_type &x, real\_type &y, real\_type &s, real\_type &t, real\_type &dst) const¶
    :   Given a point find closest point on the curve.

        Parameters
        :   - **qx** – x-coordinate of the point
            - **qy** – y-coordinate of the point
            - **x** – x-coordinate of the projected point on the curve
            - **y** – y-coordinate of the projected point on the curve
            - **s** – parameter on the curve of the projection
            - **t** – curvilinear coordinate of the point x,y (if orthogonal projection)
            - **dst** – distance point projected point

        Returns
        :   1 = point is projected orthogonal 0 = more than one projection (first returned) -1 = minimum point is not othogonal projection to curve

    inline int\_type closestPoint\_SAE(real\_type qx, real\_type qy, real\_type offs, real\_type &x, real\_type &y, real\_type &s, real\_type &t, real\_type &dst) const¶
    :   Given a point find closest point on the curve.

        Parameters
        :   - **qx** – x-coordinate of the point
            - **qy** – y-coordinate of the point
            - **offs** – offset of the curve
            - **x** – x-coordinate of the projected point on the curve
            - **y** – y-coordinate of the projected point on the curve
            - **s** – parameter on the curve of the projection
            - **t** – curvilinear coordinate of the point x,y (if orthogonal projection)
            - **dst** – distance point projected point

        Returns
        :   1 = point is projected orthogonal 0 = more than one projection (first returned) -1 = minimum point is not othogonal projection to curve

    inline virtual real\_type distance(real\_type qx, real\_type qy) const¶
    :   Compute the distance between a point \( q=(q\_x,q\_y) \) and the curve.

        Parameters
        :   - **qx** – **[in]** component \( q\_x \)
            - **qy** – **[in]** component \( q\_y \)

        Returns
        :   the computed distance

    inline real\_type distance\_ISO(real\_type qx, real\_type qy, real\_type offs) const¶
    :   Compute the distance between a point \( q=(q\_x,q\_y) \) and the curve with offset (ISO).

        Parameters
        :   - **qx** – **[in]** component \( q\_x \)
            - **qy** – **[in]** component \( q\_y \)
            - **offs** – **[in]** offset of the curve

        Returns
        :   the computed distance

    inline real\_type distance\_SAE(real\_type qx, real\_type qy, real\_type offs) const¶
    :   Compute the distance between a point \( q=(q\_x,q\_y) \) and the curve with offset (SAE).

        Parameters
        :   - **qx** – **[in]** component \( q\_x \)
            - **qy** – **[in]** component \( q\_y \)
            - **offs** – **[in]** offset of the curve

        Returns
        :   the computed distance

    inline bool findST\_ISO(real\_type x, real\_type y, real\_type &s, real\_type &t) const¶
    :   Find the curvilinear coordinate of point \( P=(x,y) \) respect to the curve (ISO), i.e.

        \[ P = C(s)+N(s)t \]

        where \( C(s) \) is the curve position respect to the curvilinear coordinates and \( C(s) \) is the normal at the point \( C(s) \).

        Parameters
        :   - **x** – **[in]** component \( x \)
            - **y** – **[in]** component \( y \)
            - **s** – **[out]** curvilinear coordinate
            - **t** – **[out]** offset respect to the curve of \( (x,y) \)

        Returns
        :   true if the coordinate are found

    inline bool findST\_SAE(real\_type x, real\_type y, real\_type &s, real\_type &t) const¶
    :   Find the curvilinear coordinate of point \( (x,y) \) respect to the curve (SAE), i.e.

        \[ P = C(s)+N(s)t \]

        where \( C(s) \) is the curve position respect to the curvilinear coordinates and \( C(s) \) is the normal at the point \( C(s) \).

        Parameters
        :   - **x** – **[in]** component \( x \)
            - **y** – **[in]** component \( y \)
            - **s** – **[out]** curvilinear coordinate
            - **t** – **[out]** offset respect to the curve of \( (x,y) \)

        Returns
        :   true if the coordinate are found

    Friends

    friend ostream\_type &operator<<(ostream\_type &stream, ClothoidList const &CL)¶

### Quick search

### Table of Contents

- Matlab Interface Manual
- C++ API
- MATLAB API

«
hide menu

menu
sidebar
»

### Navigation

- index
- toc
- next
- previous
- Clothoids »
- C++ API »
- Class ClothoidList

© Copyright 2021, Enrico Bertolazzi and Marco Frego.
Created using Sphinx 4.2.0.
