# Supplementary material for: Smooth Interpolating Curves with Local Control and Monotone Alternating Curvature
Source: Comput Graph Forum. 2022 Oct 6;41(5):25–38. doi: 10.1111/cgf.14600 (PMC9827861; doi:10.1111/cgf.14600)
Supplement: Supplementary file 1 — Supplement Material [file CGF-41-25-s001.zip › Local-Smooth-Interpolating-MonoCurvature/extern/clothoids/docs/api-cpp/class_a00171.html]

Class ClothoidSplineG2 — Clothoids v2.0.9

### Navigation

- index
- toc
- next
- previous
- Clothoids »
- C++ API »
- Class ClothoidSplineG2

# Class ClothoidSplineG2¶

- Defined in File ClothoidList.hxx

## Class Documentation¶

class G2lib::ClothoidSplineG2¶
:   Class for the computation of G2 spljne of clothoids

    Public Types

    enum TargetType¶
    :   *Values:*

        enumerator P1¶

        enumerator P2¶

        enumerator P3¶

        enumerator P4¶

        enumerator P5¶

        enumerator P6¶

        enumerator P7¶

        enumerator P8¶

        enumerator P9¶

    Public Functions

    inline ClothoidSplineG2()¶

    inline ~ClothoidSplineG2()¶

    inline void setP1(real\_type theta0, real\_type thetaN)¶

    inline void setP2()¶

    inline void setP3()¶

    inline void setP4()¶

    inline void setP5()¶

    inline void setP6()¶

    inline void setP7()¶

    inline void setP8()¶

    inline void setP9()¶

    void build(real\_type const \*xvec, real\_type const \*yvec, int\_type npts)¶

    inline int\_type numPnts() const¶

    int\_type numTheta() const¶

    int\_type numConstraints() const¶

    void guess(real\_type \*theta\_guess, real\_type \*theta\_min, real\_type \*theta\_max) const¶

    bool objective(real\_type const \*theta, real\_type &f) const¶

    bool gradient(real\_type const \*theta, real\_type \*g) const¶

    bool constraints(real\_type const \*theta, real\_type \*c) const¶

    int\_type jacobian\_nnz() const¶

    bool jacobian\_pattern(int\_type \*i, int\_type \*j) const¶

    bool jacobian\_pattern\_matlab(real\_type \*i, real\_type \*j) const¶

    bool jacobian(real\_type const \*theta, real\_type \*vals) const¶

    inline void info(ostream\_type &stream) const¶

    Friends

    friend ostream\_type &operator<<(ostream\_type &stream, ClothoidSplineG2 const &c)¶

### Quick search

### Table of Contents

- Matlab Interface Manual
- C++ API
- MATLAB API

«
hide menu

menu
sidebar
»

### Navigation

- index
- toc
- next
- previous
- Clothoids »
- C++ API »
- Class ClothoidSplineG2

© Copyright 2021, Enrico Bertolazzi and Marco Frego.
Created using Sphinx 4.2.0.
