# Supplementary material for: Smooth Interpolating Curves with Local Control and Monotone Alternating Curvature
Source: Comput Graph Forum. 2022 Oct 6;41(5):25–38. doi: 10.1111/cgf.14600 (PMC9827861; doi:10.1111/cgf.14600)
Supplement: Supplementary file 1 — Supplement Material [file CGF-41-25-s001.zip › Local-Smooth-Interpolating-MonoCurvature/extern/clothoids/docs/api-cpp/class_a00175.html]

Class Solve2x2 — Clothoids v2.0.9

### Navigation

- index
- toc
- next
- previous
- Clothoids »
- C++ API »
- Class Solve2x2

# Class Solve2x2¶

- Defined in File G2lib.hxx

## Class Documentation¶

class G2lib::Solve2x2¶
:   Class that solve a 2x2 linear system using Pseudo inverse to manage singular and near singular cases

    Public Functions

    inline Solve2x2()¶

    bool factorize(real\_type A[2][2])¶
    :   factorize matrix \( A \) , return false if factorization fails

    bool solve(real\_type const b[2], real\_type x[2]) const¶
    :   Solve the linear system \( Ax=b \) with \( A \) stored amd facted with a previous call of method `factorize`.

        Parameters
        :   - **b** – **[in]** the rhs of \( Ax=b \)
            - **x** – **[out]** the solution of \( Ax=b \)

        Returns
        :   true if solution found

### Quick search

### Table of Contents

- Matlab Interface Manual
- C++ API
- MATLAB API

«
hide menu

menu
sidebar
»

### Navigation

- index
- toc
- next
- previous
- Clothoids »
- C++ API »
- Class Solve2x2

© Copyright 2021, Enrico Bertolazzi and Marco Frego.
Created using Sphinx 4.2.0.
