# Supplementary material for: Smooth Interpolating Curves with Local Control and Monotone Alternating Curvature
Source: Comput Graph Forum. 2022 Oct 6;41(5):25–38. doi: 10.1111/cgf.14600 (PMC9827861; doi:10.1111/cgf.14600)
Supplement: Supplementary file 1 — Supplement Material [file CGF-41-25-s001.zip › Local-Smooth-Interpolating-MonoCurvature/extern/clothoids/docs/api-cpp/class_a00187.html]

Class Triangle2D — Clothoids v2.0.9

### Navigation

- index
- toc
- next
- previous
- Clothoids »
- C++ API »
- Class Triangle2D

# Class Triangle2D¶

- Defined in File Triangle2D.hxx

## Class Documentation¶

class G2lib::Triangle2D¶
:   Class to manage Triangle for BB of clothoid curve

    Public Functions

    inline Triangle2D(Triangle2D const &t)¶

    inline Triangle2D()¶

    inline Triangle2D(real\_type x1, real\_type y1, real\_type x2, real\_type y2, real\_type x3, real\_type y3, real\_type s0, real\_type s1, int\_type icurve)¶

    inline Triangle2D(real\_type const p1[2], real\_type const p2[2], real\_type const p3[2], real\_type s0, real\_type s1, int\_type icurve)¶

    inline ~Triangle2D()¶

    inline Triangle2D const &operator=(Triangle2D const &t)¶

    inline void build(real\_type const p1[2], real\_type const p2[2], real\_type const p3[2], real\_type s0, real\_type s1, int\_type icurve)¶

    inline void build(real\_type x1, real\_type y1, real\_type x2, real\_type y2, real\_type x3, real\_type y3, real\_type s0, real\_type s1, int\_type icurve)¶

    inline int\_type Icurve() const¶

    inline real\_type x1() const¶

    inline real\_type y1() const¶

    inline real\_type x2() const¶

    inline real\_type y2() const¶

    inline real\_type x3() const¶

    inline real\_type y3() const¶

    inline real\_type S0() const¶

    inline real\_type S1() const¶

    inline void translate(real\_type tx, real\_type ty)¶

    void rotate(real\_type angle, real\_type cx, real\_type cy)¶

    inline void scale(real\_type sc)¶

    inline void bbox(real\_type &xmin, real\_type &ymin, real\_type &xmax, real\_type &ymax) const¶

    inline real\_type baricenterX() const¶

    inline real\_type baricenterY() const¶

    inline real\_type const \*P1() const¶

    inline real\_type const \*P2() const¶

    inline real\_type const \*P3() const¶

    bool overlap(Triangle2D const&) const¶

    inline int\_type isCounterClockwise() const¶
    :   return +1 = CounterClockwise return -1 = Clockwise return 0 = degenerate triangle

    inline int\_type isInside(real\_type x, real\_type y) const¶
    :   return +1 = inside return -1 = outside return 0 = on the border

    inline int\_type isInside(real\_type const pt[2]) const¶

    real\_type distMin(real\_type x, real\_type y) const¶

    real\_type distMax(real\_type x, real\_type y) const¶

    inline void info(ostream\_type &stream) const¶

    Friends

    friend ostream\_type &operator<<(ostream\_type &stream, Triangle2D const &c)¶

### Quick search

### Table of Contents

- Matlab Interface Manual
- C++ API
- MATLAB API

«
hide menu

menu
sidebar
»

### Navigation

- index
- toc
- next
- previous
- Clothoids »
- C++ API »
- Class Triangle2D

© Copyright 2021, Enrico Bertolazzi and Marco Frego.
Created using Sphinx 4.2.0.
