# Supplementary material for: Smooth Interpolating Curves with Local Control and Monotone Alternating Curvature
Source: Comput Graph Forum. 2022 Oct 6;41(5):25–38. doi: 10.1111/cgf.14600 (PMC9827861; doi:10.1111/cgf.14600)
Supplement: Supplementary file 1 — Supplement Material [file CGF-41-25-s001.zip › Local-Smooth-Interpolating-MonoCurvature/extern/clothoids/docs/api-cpp/class_view_hierarchy.html]

Class Hierarchy — Clothoids v2.0.9

### Navigation

- index
- toc
- Clothoids »
- Class Hierarchy

# Class Hierarchy¶

- - Namespace G2lib
    - Class AABBtree
    - Class AsyPlot
    - Class BaseCurve
    - Class BBox
    - Class Biarc
    - Class BiarcList
    - Class CircleArc
    - Class ClothoidCurve
    - Class ClothoidList
    - Class ClothoidSplineG2
    - Class G2solve2arc
    - Class G2solve3arc
    - Class G2solveCLC
    - Class LineSegment
    - Class PolyLine
    - Class Solve2x2
    - Class Triangle2D
    - Enum CurveType

### Quick search

### Table of Contents

- Matlab Interface Manual
- C++ API
- MATLAB API

«
hide menu

menu
sidebar
»

### Navigation

- index
- toc
- Clothoids »
- Class Hierarchy

© Copyright 2021, Enrico Bertolazzi and Marco Frego.
Created using Sphinx 4.2.0.
