# Supplementary material for: Smooth Interpolating Curves with Local Control and Monotone Alternating Curvature
Source: Comput Graph Forum. 2022 Oct 6;41(5):25–38. doi: 10.1111/cgf.14600 (PMC9827861; doi:10.1111/cgf.14600)
Supplement: Supplementary file 1 — Supplement Material [file CGF-41-25-s001.zip › Local-Smooth-Interpolating-MonoCurvature/extern/clothoids/docs/api-cpp/dir_Clothoids.html]

Directory Clothoids — Clothoids v2.0.9

### Navigation

- index
- toc
- Clothoids »
- Directory Clothoids

# Directory Clothoids¶

*Directory path:* `Clothoids`

## Files¶

- File AABBtree.hxx
- File BaseCurve.hxx
- File BaseCurve\_using.hxx
- File Biarc.hxx
- File BiarcList.hxx
- File Circle.hxx
- File Clothoid.hxx
- File ClothoidAsyPlot.hxx
- File ClothoidList.hxx
- File Fresnel.hxx
- File G2lib.hxx
- File Line.hxx
- File PolyLine.hxx
- File Triangle2D.hxx

### Quick search

### Table of Contents

- Matlab Interface Manual
- C++ API
- MATLAB API

«
hide menu

menu
sidebar
»

### Navigation

- index
- toc
- Clothoids »
- Directory Clothoids

© Copyright 2021, Enrico Bertolazzi and Marco Frego.
Created using Sphinx 4.2.0.
