# Supplementary material for: Smooth Interpolating Curves with Local Control and Monotone Alternating Curvature
Source: Comput Graph Forum. 2022 Oct 6;41(5):25–38. doi: 10.1111/cgf.14600 (PMC9827861; doi:10.1111/cgf.14600)
Supplement: Supplementary file 1 — Supplement Material [file CGF-41-25-s001.zip › Local-Smooth-Interpolating-MonoCurvature/extern/clothoids/docs/api-cpp/enum_a00119_1a1974f42820495e201e07e508799543c3.html]

Enum CurveType — Clothoids v2.0.9

### Navigation

- index
- toc
- next
- previous
- Clothoids »
- C++ API »
- Enum CurveType

# Enum CurveType¶

- Defined in File BaseCurve.hxx

## Enum Documentation¶

enum G2lib::CurveType¶
:   *Values:*

    enumerator G2LIB\_LINE¶

    enumerator G2LIB\_POLYLINE¶

    enumerator G2LIB\_CIRCLE¶

    enumerator G2LIB\_BIARC¶

    enumerator G2LIB\_BIARC\_LIST¶

    enumerator G2LIB\_CLOTHOID¶

    enumerator G2LIB\_CLOTHOID\_LIST¶

### Quick search

### Table of Contents

- Matlab Interface Manual
- C++ API
- MATLAB API

«
hide menu

menu
sidebar
»

### Navigation

- index
- toc
- next
- previous
- Clothoids »
- C++ API »
- Enum CurveType

© Copyright 2021, Enrico Bertolazzi and Marco Frego.
Created using Sphinx 4.2.0.
