# Supplementary material for: Smooth Interpolating Curves with Local Control and Monotone Alternating Curvature
Source: Comput Graph Forum. 2022 Oct 6;41(5):25–38. doi: 10.1111/cgf.14600 (PMC9827861; doi:10.1111/cgf.14600)
Supplement: Supplementary file 1 — Supplement Material [file CGF-41-25-s001.zip › Local-Smooth-Interpolating-MonoCurvature/extern/clothoids/docs/api-cpp/file_Clothoid.cc.html]

File Clothoid.cc — Clothoids v2.0.9

### Navigation

- index
- toc
- Clothoids »
- File Clothoid.cc

# File Clothoid.cc¶

Contents

- Definition (`Clothoid.cc`)
- Includes
- Namespaces
- Functions

## Definition (`Clothoid.cc`)¶

- Program Listing for File Clothoid.cc

## Includes¶

- `Clothoids.hh` (File Clothoids.hh)
- `algorithm`
- `cfloat`
- `cmath`

## Namespaces¶

- Namespace G2lib

## Functions¶

- Function G2lib::operator<<(ostream\_type&, ClothoidCurve const&)

### Quick search

### Table of Contents

- Matlab Interface Manual
- C++ API
- MATLAB API

«
hide menu

menu
sidebar
»

### Navigation

- index
- toc
- Clothoids »
- File Clothoid.cc

© Copyright 2021, Enrico Bertolazzi and Marco Frego.
Created using Sphinx 4.2.0.
