# Supplementary material for: Smooth Interpolating Curves with Local Control and Monotone Alternating Curvature
Source: Comput Graph Forum. 2022 Oct 6;41(5):25–38. doi: 10.1111/cgf.14600 (PMC9827861; doi:10.1111/cgf.14600)
Supplement: Supplementary file 1 — Supplement Material [file CGF-41-25-s001.zip › Local-Smooth-Interpolating-MonoCurvature/extern/clothoids/docs/api-cpp/file_Clothoids.hh.html]

File Clothoids.hh — Clothoids v2.0.9

### Navigation

- index
- toc
- Clothoids »
- File Clothoids.hh

# File Clothoids.hh¶

Contents

- Definition (`Clothoids.hh`)
- Includes
- Included By
- Namespaces
- Defines
- Typedefs

## Definition (`Clothoids.hh`)¶

- Program Listing for File Clothoids.hh

## Includes¶

- `Clothoids/AABBtree.hxx` (File AABBtree.hxx)
- `Clothoids/BaseCurve.hxx` (File BaseCurve.hxx)
- `Clothoids/Biarc.hxx` (File Biarc.hxx)
- `Clothoids/BiarcList.hxx` (File BiarcList.hxx)
- `Clothoids/Circle.hxx` (File Circle.hxx)
- `Clothoids/Clothoid.hxx` (File Clothoid.hxx)
- `Clothoids/ClothoidAsyPlot.hxx` (File ClothoidAsyPlot.hxx)
- `Clothoids/ClothoidList.hxx` (File ClothoidList.hxx)
- `Clothoids/Fresnel.hxx` (File Fresnel.hxx)
- `Clothoids/G2lib.hxx` (File G2lib.hxx)
- `Clothoids/Line.hxx` (File Line.hxx)
- `Clothoids/PolyLine.hxx` (File PolyLine.hxx)
- `Clothoids/Triangle2D.hxx` (File Triangle2D.hxx)
- `Utils.hh`
- `algorithm`
- `cmath`
- `fstream`
- `iomanip`
- `iostream`
- `iterator`
- `map`
- `memory`
- `string`
- `utility`
- `vector`

## Included By¶

- File AABBtree.cc
- File Biarc.cc
- File BiarcList.cc
- File Circle.cc
- File Clothoid.cc
- File ClothoidAsyPlot.cc
- File ClothoidDistance.cc
- File ClothoidG2.cc
- File ClothoidList.cc
- File Fresnel.cc
- File G2lib.cc
- File G2lib\_intersect.cc
- File Line.cc
- File PolyLine.cc
- File Triangle2D.cc

## Namespaces¶

- Namespace G2lib

## Defines¶

- Define CLOTHOIDS\_dot\_HH
- Define G2LIB\_DEBUG\_MESSAGE
- Define G2LIB\_USE\_CXX11
- Define GLIB2\_TOL\_ANGLE

## Typedefs¶

- Typedef G2lib::int\_type
- Typedef G2lib::istream\_type
- Typedef G2lib::ostream\_type
- Typedef G2lib::real\_type

### Quick search

### Table of Contents

- Matlab Interface Manual
- C++ API
- MATLAB API

«
hide menu

menu
sidebar
»

### Navigation

- index
- toc
- Clothoids »
- File Clothoids.hh

© Copyright 2021, Enrico Bertolazzi and Marco Frego.
Created using Sphinx 4.2.0.
