# Supplementary material for: Smooth Interpolating Curves with Local Control and Monotone Alternating Curvature
Source: Comput Graph Forum. 2022 Oct 6;41(5):25–38. doi: 10.1111/cgf.14600 (PMC9827861; doi:10.1111/cgf.14600)
Supplement: Supplementary file 1 — Supplement Material [file CGF-41-25-s001.zip › Local-Smooth-Interpolating-MonoCurvature/extern/clothoids/docs/api-cpp/file_Clothoids_Triangle2D.hxx.html]

File Triangle2D.hxx — Clothoids v2.0.9

### Navigation

- index
- toc
- Clothoids »
- File Triangle2D.hxx

# File Triangle2D.hxx¶

↰ Parent directory (`Clothoids`)

Contents

- Definition (`Clothoids/Triangle2D.hxx`)
- Included By
- Namespaces
- Classes

## Definition (`Clothoids/Triangle2D.hxx`)¶

- Program Listing for File Triangle2D.hxx

## Included By¶

- File Clothoids.hh

## Namespaces¶

- Namespace G2lib

## Classes¶

- Class Triangle2D

### Quick search

### Table of Contents

- Matlab Interface Manual
- C++ API
- MATLAB API

«
hide menu

menu
sidebar
»

### Navigation

- index
- toc
- Clothoids »
- File Triangle2D.hxx

© Copyright 2021, Enrico Bertolazzi and Marco Frego.
Created using Sphinx 4.2.0.
