# Supplementary material for: Smooth Interpolating Curves with Local Control and Monotone Alternating Curvature
Source: Comput Graph Forum. 2022 Oct 6;41(5):25–38. doi: 10.1111/cgf.14600 (PMC9827861; doi:10.1111/cgf.14600)
Supplement: Supplementary file 1 — Supplement Material [file CGF-41-25-s001.zip › Local-Smooth-Interpolating-MonoCurvature/extern/clothoids/docs/api-cpp/file_G2lib.cc.html]

File G2lib.cc — Clothoids v2.0.9

### Navigation

- index
- toc
- Clothoids »
- File G2lib.cc

# File G2lib.cc¶

Contents

- Definition (`G2lib.cc`)
- Includes
- Namespaces
- Functions
- Variables

## Definition (`G2lib.cc`)¶

- Program Listing for File G2lib.cc

## Includes¶

- `Clothoids.hh` (File Clothoids.hh)
- `PolynomialRoots.hh`
- `algorithm`

## Namespaces¶

- Namespace G2lib

## Functions¶

- Function G2lib::Atanc
- Function G2lib::Atanc\_D
- Function G2lib::Atanc\_DD
- Function G2lib::Atanc\_DDD
- Function G2lib::Cosc
- Function G2lib::Cosc\_D
- Function G2lib::Cosc\_DD
- Function G2lib::Cosc\_DDD
- Function G2lib::intersectCircleCircle
- Function G2lib::isCounterClockwise
- Function G2lib::isPointInTriangle
- Function G2lib::projectPointOnCircle
- Function G2lib::projectPointOnCircleArc
- Function G2lib::rangeSymm
- Function G2lib::Sinc
- Function G2lib::Sinc\_D
- Function G2lib::Sinc\_DD
- Function G2lib::Sinc\_DDD
- Function G2lib::solveLinearQuadratic
- Function G2lib::solveLinearQuadratic2
- Function G2lib::xy\_to\_guess\_angle

## Variables¶

- Variable G2lib::CurveType\_name
- Variable G2lib::intersect\_with\_AABBtree
- Variable G2lib::m\_1\_sqrt\_pi
- Variable G2lib::machepsi
- Variable G2lib::machepsi10
- Variable G2lib::machepsi100
- Variable G2lib::machepsi1000
- Variable G2lib::sqrtMachepsi

### Quick search

### Table of Contents

- Matlab Interface Manual
- C++ API
- MATLAB API

«
hide menu

menu
sidebar
»

### Navigation

- index
- toc
- Clothoids »
- File G2lib.cc

© Copyright 2021, Enrico Bertolazzi and Marco Frego.
Created using Sphinx 4.2.0.
