# Supplementary material for: Smooth Interpolating Curves with Local Control and Monotone Alternating Curvature
Source: Comput Graph Forum. 2022 Oct 6;41(5):25–38. doi: 10.1111/cgf.14600 (PMC9827861; doi:10.1111/cgf.14600)
Supplement: Supplementary file 1 — Supplement Material [file CGF-41-25-s001.zip › Local-Smooth-Interpolating-MonoCurvature/extern/clothoids/docs/api-cpp/file_view_hierarchy.html]

File Hierarchy — Clothoids v2.0.9

### Navigation

- index
- toc
- Clothoids »
- File Hierarchy

# File Hierarchy¶

- - Directory Clothoids
    - File AABBtree.hxx
    - File BaseCurve.hxx
    - File BaseCurve\_using.hxx
    - File Biarc.hxx
    - File BiarcList.hxx
    - File Circle.hxx
    - File Clothoid.hxx
    - File ClothoidAsyPlot.hxx
    - File ClothoidList.hxx
    - File Fresnel.hxx
    - File G2lib.hxx
    - File Line.hxx
    - File PolyLine.hxx
    - File Triangle2D.hxx
  - File AABBtree.cc
  - File Biarc.cc
  - File BiarcList.cc
  - File Circle.cc
  - File Clothoid.cc
  - File ClothoidAsyPlot.cc
  - File ClothoidDistance.cc
  - File ClothoidG2.cc
  - File ClothoidList.cc
  - File Clothoids.hh
  - File Fresnel.cc
  - File G2lib.cc
  - File G2lib\_intersect.cc
  - File Line.cc
  - File PolyLine.cc
  - File Triangle2D.cc

### Quick search

### Table of Contents

- Matlab Interface Manual
- C++ API
- MATLAB API

«
hide menu

menu
sidebar
»

### Navigation

- index
- toc
- Clothoids »
- File Hierarchy

© Copyright 2021, Enrico Bertolazzi and Marco Frego.
Created using Sphinx 4.2.0.
