# Supplementary material for: Smooth Interpolating Curves with Local Control and Monotone Alternating Curvature
Source: Comput Graph Forum. 2022 Oct 6;41(5):25–38. doi: 10.1111/cgf.14600 (PMC9827861; doi:10.1111/cgf.14600)
Supplement: Supplementary file 1 — Supplement Material [file CGF-41-25-s001.zip › Local-Smooth-Interpolating-MonoCurvature/extern/clothoids/docs/api-cpp/function_a00119_1a4d0a558dc1e34cf736febb3c4de4c662.html]

Function G2lib::FresnelCS(int\_type, real\_type, real\_type \*, real\_type \*) — Clothoids v2.0.9

### Navigation

- index
- toc
- next
- previous
- Clothoids »
- C++ API »
- Function G2lib::FresnelCS(int\_type, real\_type, real\_type \*, real\_type \*)

# Function G2lib::FresnelCS(int\_type, real\_type, real\_type \*, real\_type \*)¶

- Defined in File Fresnel.cc

## Function Documentation¶

void G2lib::FresnelCS(int\_type nk, real\_type x, real\_type \*C, real\_type \*S)¶
:   Compute Fresnel integrals and its derivatives

    \[ C(x) = \int\_0^x \cos\left(\frac{\pi}{2}t^2\right) dt, \qquad S(x) = \int\_0^x \sin\left(\frac{\pi}{2}t^2\right) dt \]

    Parameters
    :   - **nk** – maximum order of the derivative
        - **x** – the input abscissa
        - **S** – S[0]= \( S(x) \), S[1]= \( S'(x) \), S[2]= \( S''(x) \)
        - **C** – C[0]= \( C(x) \), C[1]= \( C'(x) \), C[2]= \( C''(x) \)

### Quick search

### Table of Contents

- Matlab Interface Manual
- C++ API
- MATLAB API

«
hide menu

menu
sidebar
»

### Navigation

- index
- toc
- next
- previous
- Clothoids »
- C++ API »
- Function G2lib::FresnelCS(int\_type, real\_type, real\_type \*, real\_type \*)

© Copyright 2021, Enrico Bertolazzi and Marco Frego.
Created using Sphinx 4.2.0.
