# Supplementary material for: Smooth Interpolating Curves with Local Control and Monotone Alternating Curvature
Source: Comput Graph Forum. 2022 Oct 6;41(5):25–38. doi: 10.1111/cgf.14600 (PMC9827861; doi:10.1111/cgf.14600)
Supplement: Supplementary file 1 — Supplement Material [file CGF-41-25-s001.zip › Local-Smooth-Interpolating-MonoCurvature/extern/clothoids/docs/api-cpp/function_a00119_1a67f7c38f4ad0d8256c2f4d0f92501bf2.html]

Function G2lib::GeneralizedFresnelCS(real\_type, real\_type, real\_type, real\_type&, real\_type&) — Clothoids v2.0.9

### Navigation

- index
- toc
- next
- previous
- Clothoids »
- C++ API »
- Function G2lib::GeneralizedFresnelCS(real\_type, real\_type, real\_type, real\_type&, real\_type&)

# Function G2lib::GeneralizedFresnelCS(real\_type, real\_type, real\_type, real\_type&, real\_type&)¶

- Defined in File Fresnel.cc

## Function Documentation¶

void G2lib::GeneralizedFresnelCS(real\_type a, real\_type b, real\_type c, real\_type &intC, real\_type &intS)¶
:   Compute the Fresnel integrals

    \[ \int\_0^1 t^k \cos\left(a\frac{t^2}{2} + b t + c\right) dt,\qquad \int\_0^1 t^k \sin\left(a\frac{t^2}{2} + b t + c\right) dt \]

    Parameters
    :   - **a** – parameter \( a \)
        - **b** – parameter \( b \)
        - **c** – parameter \( c \)
        - **intC** – cosine integrals,
        - **intS** – sine integrals

### Quick search

### Table of Contents

- Matlab Interface Manual
- C++ API
- MATLAB API

«
hide menu

menu
sidebar
»

### Navigation

- index
- toc
- next
- previous
- Clothoids »
- C++ API »
- Function G2lib::GeneralizedFresnelCS(real\_type, real\_type, real\_type, real\_type&, real\_type&)

© Copyright 2021, Enrico Bertolazzi and Marco Frego.
Created using Sphinx 4.2.0.
