# Supplementary material for: Smooth Interpolating Curves with Local Control and Monotone Alternating Curvature
Source: Comput Graph Forum. 2022 Oct 6;41(5):25–38. doi: 10.1111/cgf.14600 (PMC9827861; doi:10.1111/cgf.14600)
Supplement: Supplementary file 1 — Supplement Material [file CGF-41-25-s001.zip › Local-Smooth-Interpolating-MonoCurvature/extern/clothoids/docs/api-cpp/function_a00119_1a753ce69800a98e59ce94300c441c2ed7.html]

Function G2lib::solveLinearQuadratic2 — Clothoids v2.0.9

### Navigation

- index
- toc
- next
- previous
- Clothoids »
- C++ API »
- Function G2lib::solveLinearQuadratic2

# Function G2lib::solveLinearQuadratic2¶

- Defined in File G2lib.cc

## Function Documentation¶

int\_type G2lib::solveLinearQuadratic2(real\_type A, real\_type B, real\_type C, real\_type \*x, real\_type \*y)¶
:   Solve the nonlinear system

    \[ A x + B y = C \]

    \[ x^2 + y^2 = 1 \]

    Parameters
    :   - **A** – **[in]** first parameter of the linear equation
        - **B** – **[in]** second parameter of the linear equation
        - **C** – **[in]** third parameter of the linear equation
        - **x** – **[out]** x-coordinates of the solutions
        - **y** – **[out]** y-coordinates of the solutions

    Returns
    :   the number of solution 0, 1 or 2

### Quick search

### Table of Contents

- Matlab Interface Manual
- C++ API
- MATLAB API

«
hide menu

menu
sidebar
»

### Navigation

- index
- toc
- next
- previous
- Clothoids »
- C++ API »
- Function G2lib::solveLinearQuadratic2

© Copyright 2021, Enrico Bertolazzi and Marco Frego.
Created using Sphinx 4.2.0.
