# Supplementary material for: Smooth Interpolating Curves with Local Control and Monotone Alternating Curvature
Source: Comput Graph Forum. 2022 Oct 6;41(5):25–38. doi: 10.1111/cgf.14600 (PMC9827861; doi:10.1111/cgf.14600)
Supplement: Supplementary file 1 — Supplement Material [file CGF-41-25-s001.zip › Local-Smooth-Interpolating-MonoCurvature/extern/clothoids/docs/api-cpp/function_a00119_1a8255221ca042d54056cb046fa8162f8e.html]

Function G2lib::isPointInTriangle — Clothoids v2.0.9

### Navigation

- index
- toc
- next
- previous
- Clothoids »
- C++ API »
- Function G2lib::isPointInTriangle

# Function G2lib::isPointInTriangle¶

- Defined in File G2lib.cc

## Function Documentation¶

int\_type G2lib::isPointInTriangle(real\_type const \*pt, real\_type const \*P1, real\_type const \*P2, real\_type const \*P3)¶
:   Check if a point is inside a triangle

    Parameters
    :   - **pt** – **[in]** point to check if is inside the triangle
        - **P1** – **[in]** first point of the triangle
        - **P2** – **[in]** second point of the triangle
        - **P3** – **[in]** third point of the triangle

    Returns
    :   {0,+1,-1} return +1 = Inside return -1 = Outsize return 0 = on border

### Quick search

### Table of Contents

- Matlab Interface Manual
- C++ API
- MATLAB API

«
hide menu

menu
sidebar
»

### Navigation

- index
- toc
- next
- previous
- Clothoids »
- C++ API »
- Function G2lib::isPointInTriangle

© Copyright 2021, Enrico Bertolazzi and Marco Frego.
Created using Sphinx 4.2.0.
