# Supplementary material for: Smooth Interpolating Curves with Local Control and Monotone Alternating Curvature
Source: Comput Graph Forum. 2022 Oct 6;41(5):25–38. doi: 10.1111/cgf.14600 (PMC9827861; doi:10.1111/cgf.14600)
Supplement: Supplementary file 1 — Supplement Material [file CGF-41-25-s001.zip › Local-Smooth-Interpolating-MonoCurvature/extern/clothoids/docs/api-cpp/function_a00119_1a8829b54b63a6e1880f15ccdfe57c2ecb.html]

Function G2lib::projectPointOnCircle — Clothoids v2.0.9

### Navigation

- index
- toc
- next
- previous
- Clothoids »
- C++ API »
- Function G2lib::projectPointOnCircle

# Function G2lib::projectPointOnCircle¶

- Defined in File G2lib.cc

## Function Documentation¶

real\_type G2lib::projectPointOnCircle(real\_type x0, real\_type y0, real\_type theta0, real\_type k, real\_type qx, real\_type qy)¶
:   Project point `(qx,qy)` to the circle passing from `(x0,y0)` with tangent direction `(c0,s0)` and curvature `k`

    Parameters
    :   - **x0** – **[in]** x-starting point of circle arc
        - **y0** – **[in]** y-starting point of circle arc
        - **theta0** – **[in]** initial angle
        - **k** – **[in]** curvature
        - **qx** – **[in]** x-point to be projected
        - **qy** – **[in]** y-point to be projected

    Returns
    :   distance point circle

### Quick search

### Table of Contents

- Matlab Interface Manual
- C++ API
- MATLAB API

«
hide menu

menu
sidebar
»

### Navigation

- index
- toc
- next
- previous
- Clothoids »
- C++ API »
- Function G2lib::projectPointOnCircle

© Copyright 2021, Enrico Bertolazzi and Marco Frego.
Created using Sphinx 4.2.0.
