# Supplementary material for: Smooth Interpolating Curves with Local Control and Monotone Alternating Curvature
Source: Comput Graph Forum. 2022 Oct 6;41(5):25–38. doi: 10.1111/cgf.14600 (PMC9827861; doi:10.1111/cgf.14600)
Supplement: Supplementary file 1 — Supplement Material [file CGF-41-25-s001.zip › Local-Smooth-Interpolating-MonoCurvature/extern/clothoids/docs/api-cpp/function_a00119_1aa5b1fe23ac7694a14290b292fe657cd2.html]

Function G2lib::collision\_SAE — Clothoids v2.0.9

### Navigation

- index
- toc
- next
- previous
- Clothoids »
- C++ API »
- Function G2lib::collision\_SAE

# Function G2lib::collision\_SAE¶

- Defined in File BaseCurve.hxx

## Function Documentation¶

inline bool G2lib::collision\_SAE(BaseCurve const &C1, real\_type offs\_C1, BaseCurve const &C2, real\_type offs\_C2)¶
:   Return `true` the the two curves intersect.

    Parameters
    :   - **C1** – **[in]** first curve
        - **offs\_C1** – **[in]** offset of the first curve
        - **C2** – **[in]** second curve
        - **offs\_C2** – **[in]** offset of the second curve

### Quick search

### Table of Contents

- Matlab Interface Manual
- C++ API
- MATLAB API

«
hide menu

menu
sidebar
»

### Navigation

- index
- toc
- next
- previous
- Clothoids »
- C++ API »
- Function G2lib::collision\_SAE

© Copyright 2021, Enrico Bertolazzi and Marco Frego.
Created using Sphinx 4.2.0.
