# Supplementary material for: Smooth Interpolating Curves with Local Control and Monotone Alternating Curvature
Source: Comput Graph Forum. 2022 Oct 6;41(5):25–38. doi: 10.1111/cgf.14600 (PMC9827861; doi:10.1111/cgf.14600)
Supplement: Supplementary file 1 — Supplement Material [file CGF-41-25-s001.zip › Local-Smooth-Interpolating-MonoCurvature/extern/clothoids/docs/api-cpp/function_a00119_1aa745733d178f053f0092b5ecc892bd44.html]

Function G2lib::build\_guess\_theta — Clothoids v2.0.9

### Navigation

- index
- toc
- next
- previous
- Clothoids »
- C++ API »
- Function G2lib::build\_guess\_theta

# Function G2lib::build\_guess\_theta¶

- Defined in File Biarc.cc

## Function Documentation¶

bool G2lib::build\_guess\_theta(int\_type n, real\_type const \*x, real\_type const \*y, real\_type \*theta)¶
:   Given a list of points \( (x\_i,y\_i) \) build a guess of angles for a spline of biarc.

    Parameters
    :   - **n** – **[in]** number of points
        - **x** – **[in]** x-coordinates
        - **y** – **[in]** y-coordinates
        - **theta** – **[out]** guessed angles

### Quick search

### Table of Contents

- Matlab Interface Manual
- C++ API
- MATLAB API

«
hide menu

menu
sidebar
»

### Navigation

- index
- toc
- next
- previous
- Clothoids »
- C++ API »
- Function G2lib::build\_guess\_theta

© Copyright 2021, Enrico Bertolazzi and Marco Frego.
Created using Sphinx 4.2.0.
