# Supplementary material for: Smooth Interpolating Curves with Local Control and Monotone Alternating Curvature
Source: Comput Graph Forum. 2022 Oct 6;41(5):25–38. doi: 10.1111/cgf.14600 (PMC9827861; doi:10.1111/cgf.14600)
Supplement: Supplementary file 1 — Supplement Material [file CGF-41-25-s001.zip › Local-Smooth-Interpolating-MonoCurvature/extern/clothoids/docs/api-cpp/function_a00119_1aaf57a0b7127fcdb2ee4aaef288c4e527.html]

Function G2lib::isCounterClockwise — Clothoids v2.0.9

### Navigation

- index
- toc
- next
- previous
- Clothoids »
- C++ API »
- Function G2lib::isCounterClockwise

# Function G2lib::isCounterClockwise¶

- Defined in File G2lib.cc

## Function Documentation¶

int\_type G2lib::isCounterClockwise(real\_type const \*P1, real\_type const \*P2, real\_type const \*P3)¶
:   Return the orientation of a triangle

    return +1 = CounterClockwise return -1 = Clockwise return 0 = flat

    CounterClockwise: the path P1->P2->P3 turns Counter-Clockwise, i.e., the point P3 is located “on the left” of the line P1-P2. Clockwise: the path turns Clockwise, i.e., the point P3 lies “on the right” of the line P1-P2. flat: the point P3 is located on the line segment [P1 P2].

    Algorithm from FileExchage geom2d adapated from Sedgewick’s book.

    Parameters
    :   - **P1** – **[in]** first point of the triangle
        - **P2** – **[in]** second point of the triangle
        - **P3** – **[in]** third point of the triangle

    Returns
    :   sign of rotation

### Quick search

### Table of Contents

- Matlab Interface Manual
- C++ API
- MATLAB API

«
hide menu

menu
sidebar
»

### Navigation

- index
- toc
- next
- previous
- Clothoids »
- C++ API »
- Function G2lib::isCounterClockwise

© Copyright 2021, Enrico Bertolazzi and Marco Frego.
Created using Sphinx 4.2.0.
