# Supplementary material for: Smooth Interpolating Curves with Local Control and Monotone Alternating Curvature
Source: Comput Graph Forum. 2022 Oct 6;41(5):25–38. doi: 10.1111/cgf.14600 (PMC9827861; doi:10.1111/cgf.14600)
Supplement: Supplementary file 1 — Supplement Material [file CGF-41-25-s001.zip › Local-Smooth-Interpolating-MonoCurvature/extern/clothoids/docs/api-cpp/function_a00119_1ad2fbbd505c9e9e83b15a7b74aedd32a3.html]

Function G2lib::intersectCircleCircle — Clothoids v2.0.9

### Navigation

- index
- toc
- next
- previous
- Clothoids »
- C++ API »
- Function G2lib::intersectCircleCircle

# Function G2lib::intersectCircleCircle¶

- Defined in File G2lib.cc

## Function Documentation¶

int\_type G2lib::intersectCircleCircle(real\_type x1, real\_type y1, real\_type theta1, real\_type kappa1, real\_type x2, real\_type y2, real\_type theta2, real\_type kappa2, real\_type \*s1, real\_type \*s2)¶
:   Intersect the parametric arc

    \[ x = x\_1+\frac{\sin(\kappa\_1 s+\theta\_1)-sin(\theta\_1)}{\kappa\_1} \]

    \[ y = y\_1+\frac{\cos(\theta\_1)-\cos(\kappa\_1 s+\theta\_1)}{\kappa\_1} \]

    with the parametric arc

    \[ x = x\_2+\frac{\sin(\kappa\_2 s+\theta\_2)-sin(\theta\_2)}{\kappa\_2} \]

    \[ y = y\_2+\frac{\cos(\theta\_2)-\cos(\kappa\_2 s+\theta\_2)}{\kappa\_2} \]

    Parameters
    :   - **x1** – **[in]** x-origin of the first arc
        - **y1** – **[in]** y-origin of the first arc
        - **theta1** – **[in]** initial angle of the first arc
        - **kappa1** – **[in]** curvature of the first arc
        - **x2** – **[in]** x-origin of the second arc
        - **y2** – **[in]** y-origin of the second arc
        - **theta2** – **[in]** initial angle of the second arc
        - **kappa2** – **[in]** curvature of the second arc
        - **s1** – **[out]** parameter2 of intersection for the first circle arc
        - **s2** – **[out]** parameter2 of intersection for the second circle arc

    Returns
    :   the number of solution 0, 1 or 2

### Quick search

### Table of Contents

- Matlab Interface Manual
- C++ API
- MATLAB API

«
hide menu

menu
sidebar
»

### Navigation

- index
- toc
- next
- previous
- Clothoids »
- C++ API »
- Function G2lib::intersectCircleCircle

© Copyright 2021, Enrico Bertolazzi and Marco Frego.
Created using Sphinx 4.2.0.
