# Supplementary material for: Smooth Interpolating Curves with Local Control and Monotone Alternating Curvature
Source: Comput Graph Forum. 2022 Oct 6;41(5):25–38. doi: 10.1111/cgf.14600 (PMC9827861; doi:10.1111/cgf.14600)
Supplement: Supplementary file 1 — Supplement Material [file CGF-41-25-s001.zip › Local-Smooth-Interpolating-MonoCurvature/extern/clothoids/docs/api-cpp/function_a00119_1aea24641ba4514e4557cd7e9a4ada946c.html]

Function G2lib::xy\_to\_guess\_angle — Clothoids v2.0.9

### Navigation

- index
- toc
- next
- previous
- Clothoids »
- C++ API »
- Function G2lib::xy\_to\_guess\_angle

# Function G2lib::xy\_to\_guess\_angle¶

- Defined in File G2lib.cc

## Function Documentation¶

void G2lib::xy\_to\_guess\_angle(int\_type npts, real\_type const \*x, real\_type const \*y, real\_type \*theta, real\_type \*theta\_min, real\_type \*theta\_max, real\_type \*omega, real\_type \*len)¶
:   Given a list of \( n \) points \( (x\_i,y\_i) \) compute the guess angles for the \( G^2 \) curve construction.

    Parameters
    :   - **npts** – **[in]** \( n \)
        - **x** – **[in]** x-coordinates of the points
        - **y** – **[in]** y-coordinates of the points
        - **theta** – **[out]** guess angles
        - **theta\_min** – **[out]** minimum angles at each nodes
        - **theta\_max** – **[out]** maximum angles at each nodes
        - **omega** – **[out]** angles of two consecutive points, with accumulated \( 2\pi \) angle rotation
        - **len** – **[out]** distance between two consecutive poijts

### Quick search

### Table of Contents

- Matlab Interface Manual
- C++ API
- MATLAB API

«
hide menu

menu
sidebar
»

### Navigation

- index
- toc
- next
- previous
- Clothoids »
- C++ API »
- Function G2lib::xy\_to\_guess\_angle

© Copyright 2021, Enrico Bertolazzi and Marco Frego.
Created using Sphinx 4.2.0.
