# Supplementary material for: Smooth Interpolating Curves with Local Control and Monotone Alternating Curvature
Source: Comput Graph Forum. 2022 Oct 6;41(5):25–38. doi: 10.1111/cgf.14600 (PMC9827861; doi:10.1111/cgf.14600)
Supplement: Supplementary file 1 — Supplement Material [file CGF-41-25-s001.zip › Local-Smooth-Interpolating-MonoCurvature/extern/clothoids/docs/api-cpp/function_a00119_1aee78c857e298d1605987e48534793843.html]

Function G2lib::FresnelCS(real\_type, real\_type&, real\_type&) — Clothoids v2.0.9

### Navigation

- index
- toc
- next
- previous
- Clothoids »
- C++ API »
- Function G2lib::FresnelCS(real\_type, real\_type&, real\_type&)

# Function G2lib::FresnelCS(real\_type, real\_type&, real\_type&)¶

- Defined in File Fresnel.cc

## Function Documentation¶

void G2lib::FresnelCS(real\_type x, real\_type &C, real\_type &S)¶
:   **Purpose:**

    Compute Fresnel integrals C(x) and S(x)

    \[ S(x) = \int\_0^x \sin t^2 \,\mathrm{d} t, \qquad C(x) = \int\_0^x \cos t^2 \,\mathrm{d} t \]

    **Example:**

    | \( x \) | \( C(x) \) | \( S(x) \) |
    | --- | --- | --- |
    | 0.0 | 0.00000000 | 0.00000000 |
    | 0.5 | 0.49234423 | 0.06473243 |
    | 1.0 | 0.77989340 | 0.43825915 |
    | 1.5 | 0.44526118 | 0.69750496 |
    | 2.0 | 0.48825341 | 0.34341568 |
    | 2.5 | 0.45741301 | 0.61918176 |

    **Adapted from:**

    - *William J. Thompson*, Atlas for computing mathematical functions : an illustrated guide for practitioners, with programs in C and Mathematica, Wiley, 1997.

    **Author:**

    - *Venkata Sivakanth Telasula*, email: , date: August 11, 2005

    Parameters
    :   - **y** – **[in]** Argument of \( C(y) \) and \( S(y) \)
        - **C** – **[out]** \( C(x) \)
        - **S** – **[out]** \( S(x) \)

### Quick search

### Table of Contents

- Matlab Interface Manual
- C++ API
- MATLAB API

«
hide menu

menu
sidebar
»

### Navigation

- index
- toc
- next
- previous
- Clothoids »
- C++ API »
- Function G2lib::FresnelCS(real\_type, real\_type&, real\_type&)

© Copyright 2021, Enrico Bertolazzi and Marco Frego.
Created using Sphinx 4.2.0.
