# Supplementary material for: Smooth Interpolating Curves with Local Control and Monotone Alternating Curvature
Source: Comput Graph Forum. 2022 Oct 6;41(5):25–38. doi: 10.1111/cgf.14600 (PMC9827861; doi:10.1111/cgf.14600)
Supplement: Supplementary file 1 — Supplement Material [file CGF-41-25-s001.zip › Local-Smooth-Interpolating-MonoCurvature/extern/clothoids/docs/api-cpp/namespace_G2lib.html]

Namespace G2lib — Clothoids v2.0.9

### Navigation

- index
- toc
- next
- previous
- Clothoids »
- C++ API »
- Namespace G2lib

# Namespace G2lib¶

Contents

- Detailed Description
- Classes
- Enums
- Functions
- Typedefs
- Variables

## Detailed Description¶

file: AABBtree.hh

file: File Biarc.cc

file: File AABBtree.hxx

file: BaseCurve.hh

file: File Biarc.hxx

file: BiarcList.hh

file: File Circle.hxx

file: Clothoid.hh

file: ClothoidList.hh

file: File Fresnel.hxx

file: G2lib.hh Clothoid computations routine

file: File Line.hxx

file: File PolyLine.hxx

file: File Triangle2D.hxx

## Classes¶

- Class AABBtree
- Class AsyPlot
- Class BaseCurve
- Class BBox
- Class Biarc
- Class BiarcList
- Class CircleArc
- Class ClothoidCurve
- Class ClothoidList
- Class ClothoidSplineG2
- Class G2solve2arc
- Class G2solve3arc
- Class G2solveCLC
- Class LineSegment
- Class PolyLine
- Class Solve2x2
- Class Triangle2D

## Enums¶

- Enum CurveType

## Functions¶

- Function G2lib::Atanc
- Function G2lib::Atanc\_D
- Function G2lib::Atanc\_DD
- Function G2lib::Atanc\_DDD
- Function G2lib::build\_guess\_theta
- Function G2lib::collision
- Function G2lib::collision\_ISO
- Function G2lib::collision\_SAE
- Function G2lib::Cosc
- Function G2lib::Cosc\_D
- Function G2lib::Cosc\_DD
- Function G2lib::Cosc\_DDD
- Function G2lib::FresnelCS(real\_type, real\_type&, real\_type&)
- Function G2lib::FresnelCS(int\_type, real\_type, real\_type \*, real\_type \*)
- Function G2lib::GeneralizedFresnelCS(int\_type, real\_type, real\_type, real\_type, real\_type \*, real\_type \*)
- Function G2lib::GeneralizedFresnelCS(real\_type, real\_type, real\_type, real\_type&, real\_type&)
- Function G2lib::intersect
- Function G2lib::intersect\_ISO
- Function G2lib::intersect\_SAE
- Function G2lib::intersectCircleCircle
- Function G2lib::isCounterClockwise
- Function G2lib::isPointInTriangle
- Function G2lib::minmax3
- Function G2lib::noAABBtree
- Function G2lib::operator<<(ostream\_type&, Biarc const&)
- Function G2lib::operator<<(ostream\_type&, BiarcList const&)
- Function G2lib::operator<<(ostream\_type&, CircleArc const&)
- Function G2lib::operator<<(ostream\_type&, ClothoidCurve const&)
- Function G2lib::operator<<(ostream\_type&, ClothoidSplineG2 const&)
- Function G2lib::operator<<(ostream\_type&, ClothoidList const&)
- Function G2lib::operator<<(ostream\_type&, BBox const&)
- Function G2lib::operator<<(ostream\_type&, LineSegment const&)
- Function G2lib::operator<<(ostream\_type&, PolyLine const&)
- Function G2lib::operator<<(ostream\_type&, Triangle2D const&)
- Function G2lib::pointInsideCircle
- Function G2lib::projectPointOnCircle
- Function G2lib::projectPointOnCircleArc
- Function G2lib::rangeSymm
- Function G2lib::Sinc
- Function G2lib::Sinc\_D
- Function G2lib::Sinc\_DD
- Function G2lib::Sinc\_DDD
- Function G2lib::solveLinearQuadratic
- Function G2lib::solveLinearQuadratic2
- Function G2lib::xy\_to\_guess\_angle
- Function G2lib::yesAABBtree

## Typedefs¶

- Typedef G2lib::int\_type
- Typedef G2lib::IntersectList
- Typedef G2lib::Ipair
- Typedef G2lib::istream\_type
- Typedef G2lib::ostream\_type
- Typedef G2lib::real\_type

## Variables¶

- Variable G2lib::CurveType\_name
- Variable G2lib::intersect\_with\_AABBtree
- Variable G2lib::m\_1\_sqrt\_pi
- Variable G2lib::machepsi
- Variable G2lib::machepsi10
- Variable G2lib::machepsi100
- Variable G2lib::machepsi1000
- Variable G2lib::sqrtMachepsi

### Quick search

### Table of Contents

- Matlab Interface Manual
- C++ API
- MATLAB API

«
hide menu

menu
sidebar
»

### Navigation

- index
- toc
- next
- previous
- Clothoids »
- C++ API »
- Namespace G2lib

© Copyright 2021, Enrico Bertolazzi and Marco Frego.
Created using Sphinx 4.2.0.
