# Supplementary material for: Smooth Interpolating Curves with Local Control and Monotone Alternating Curvature
Source: Comput Graph Forum. 2022 Oct 6;41(5):25–38. doi: 10.1111/cgf.14600 (PMC9827861; doi:10.1111/cgf.14600)
Supplement: Supplementary file 1 — Supplement Material [file CGF-41-25-s001.zip › Local-Smooth-Interpolating-MonoCurvature/extern/clothoids/docs/api-cpp/program_listing_file_AABBtree.cc.html]

Program Listing for File AABBtree.cc — Clothoids v2.0.9

### Navigation

- index
- toc
- Clothoids »
- Program Listing for File AABBtree.cc

# Program Listing for File AABBtree.cc¶

↰ Return to documentation for file (`AABBtree.cc`)

```
/*--------------------------------------------------------------------------*\
 |                                                                          |
 |  Copyright (C) 2018                                                      |
 |                                                                          |
 |         , __                 , __                                        |
 |        /|/  \               /|/  \                                       |
 |         | __/ _   ,_         | __/ _   ,_                                |
 |         |   \|/  /  |  |   | |   \|/  /  |  |   |                        |
 |         |(__/|__/   |_/ \_/|/|(__/|__/   |_/ \_/|/                       |
 |                           /|                   /|                        |
 |                           \|                   \|                        |
 |                                                                          |
 |      Paolo Bevilacqua and Enrico Bertolazzi                              |
 |                                                                          |
 |      (1) Dipartimento di Ingegneria e Scienza dell'Informazione          |
 |      (2) Dipartimento di Ingegneria Industriale                          |
 |                                                                          |
 |      Universita` degli Studi di Trento                                   |
 |      email: paolo.bevilacqua@unitn.it                                    |
 |      email: enrico.bertolazzi@unitn.it                                   |
 |                                                                          |
\*--------------------------------------------------------------------------*/


#include "Clothoids.hh"

// Workaround for Visual Studio
#ifdef min
  #undef min
#endif

#ifdef max
  #undef max
#endif

#include <algorithm>

namespace G2lib {

  using std::abs;
  using std::min;
  using std::max;
  using std::numeric_limits;

  /*\
   |   ____  ____
   |  | __ )| __ )  _____  __
   |  |  _ \|  _ \ / _ \ \/ /
   |  | |_) | |_) | (_) >  <
   |  |____/|____/ \___/_/\_\
  \*/

  void
  BBox::join( vector<PtrBBox> const & bboxes ) {
    if ( bboxes.empty() ) {
      m_xmin = m_ymin = m_xmax = m_ymax = 0;
    } else {
      vector<PtrBBox>::const_iterator it = bboxes.begin();

      m_xmin = (*it)->m_xmin;
      m_ymin = (*it)->m_ymin;
      m_xmax = (*it)->m_xmax;
      m_ymax = (*it)->m_ymax;

      for ( ++it; it != bboxes.end(); ++it ) {
        BBox const & currBox = **it;
        if ( currBox.m_xmin < m_xmin ) m_xmin = currBox.m_xmin;
        if ( currBox.m_ymin < m_ymin ) m_ymin = currBox.m_ymin;
        if ( currBox.m_xmax > m_xmax ) m_xmax = currBox.m_xmax;
        if ( currBox.m_ymax > m_ymax ) m_ymax = currBox.m_ymax;
      }
    }
  }

  // . . . . . . . . . . . . . . . . . . . . . . . . . . . . . . . . . . . . . .

  real_type
  BBox::distance( real_type x, real_type y ) const {
    /*\
     |
     |   6          7          8
     |       +-------------+
     |       |             |
     |   3   |      4      |   5
     |       |             |
     |       +-------------+
     |   0          1          2
     |
    \*/
    int_type icase = 4;
    if      ( x < m_xmin ) icase = 3;
    else if ( x > m_xmax ) icase = 5;
    if      ( y < m_ymin ) icase -= 3;
    else if ( y > m_ymax ) icase += 3;
    real_type dst = 0;
    switch ( icase ) {
      case 0: dst = hypot( x-m_xmin, y-m_ymin); break;
      case 1: dst = m_ymin-y;                   break;
      case 2: dst = hypot( x-m_xmax, y-m_ymin); break;
      case 3: dst = m_xmin-x;                   break;
      case 4:                                   break;
      case 5: dst = x-m_xmax;                   break;
      case 6: dst = hypot( x-m_xmin, y-m_ymax); break;
      case 7: dst = y-m_ymax;                   break;
      case 8: dst = hypot( x-m_xmax, y-m_ymax); break;
    }
    return dst;
  }

  // . . . . . . . . . . . . . . . . . . . . . . . . . . . . . . . . . . . . . .

  real_type
  BBox::maxDistance( real_type x, real_type y ) const {
    real_type dx = max( abs(x-m_xmin), abs(x-m_xmax) );
    real_type dy = max( abs(y-m_ymin), abs(y-m_ymax) );
    return hypot(dx,dy);
  }

  /*\
   |      _        _    ____  ____  _
   |     / \      / \  | __ )| __ )| |_ _ __ ___  ___
   |    / _ \    / _ \ |  _ \|  _ \| __| '__/ _ \/ _ \
   |   / ___ \  / ___ \| |_) | |_) | |_| | |  __/  __/
   |  /_/   \_\/_/   \_\____/|____/ \__|_|  \___|\___|
  \*/

  #ifdef G2LIB_USE_CXX11

  AABBtree::AABBtree() {
    pBBox.reset();
    children.clear();
  }

  AABBtree::~AABBtree() {
    pBBox.reset();
    children.clear();
  }

  void
  AABBtree::clear() {
    pBBox.reset();
    children.clear();
  }

  bool
  AABBtree::empty() const {
    return children.empty() && !pBBox;
  }

  #else

  AABBtree::AABBtree()
  : pBBox(nullptr)
  {
    children.clear();
  }

  AABBtree::~AABBtree() {
    if ( pBBox != nullptr ) {
      delete pBBox;
      pBBox = nullptr;
    }
    children.clear();
  }

  void
  AABBtree::clear() {
    if ( pBBox != nullptr ) {
      delete pBBox;
      pBBox = nullptr;
    }
    children.clear();
  }

  bool
  AABBtree::empty() const {
    return children.empty() && pBBox == nullptr;
  }

  #endif

  // . . . . . . . . . . . . . . . . . . . . . . . . . . . . . . . . . . . . . .

  void
  AABBtree::build( vector<PtrBBox> const & bboxes ) {
    clear();

    if ( bboxes.empty() ) return;

    size_t size = bboxes.size();

    if ( size == 1 ) {
      this -> pBBox = bboxes.front();
      return;
    }

    #ifdef G2LIB_USE_CXX11
    pBBox = shared_ptr<BBox>( new BBox(bboxes, 0, 0) );
    #else
    if ( pBBox != nullptr ) { delete pBBox; pBBox = nullptr; }
    pBBox = new BBox( bboxes, 0, 0 );
    #endif

    real_type xmin = pBBox -> Xmin();
    real_type ymin = pBBox -> Ymin();
    real_type xmax = pBBox -> Xmax();
    real_type ymax = pBBox -> Ymax();

    vector<PtrBBox> posBoxes;
    vector<PtrBBox> negBoxes;

    if ( (ymax - ymin) > (xmax - xmin) ) {
      real_type cutPos = (ymax + ymin)/2;
      vector<PtrBBox>::const_iterator it;
      for ( it = bboxes.begin(); it != bboxes.end(); ++it ) {
        real_type ymid = ( (*it) -> Ymin() + (*it) -> Ymax() ) / 2;
        if ( ymid > cutPos ) posBoxes.push_back(*it);
        else                 negBoxes.push_back(*it);
      }
    } else {
      real_type cutPos = (xmax + xmin)/2;
      vector<PtrBBox>::const_iterator it;
      for ( it = bboxes.begin(); it != bboxes.end(); ++it ) {
        real_type xmid = ( (*it) -> Xmin() + (*it) -> Xmax() ) / 2;
        if ( xmid > cutPos ) posBoxes.push_back(*it);
        else                 negBoxes.push_back(*it);
      }
    }

    if ( negBoxes.empty() ) {
      vector<PtrBBox>::iterator midIdx;
      midIdx = posBoxes.begin() + posBoxes.size()/2;
      negBoxes.insert( negBoxes.end(), midIdx, posBoxes.end() );
      posBoxes.erase( midIdx, posBoxes.end() );
    } else if ( posBoxes.empty() ) {
      vector<PtrBBox>::iterator midIdx;
      midIdx = negBoxes.begin() + negBoxes.size()/2;
      posBoxes.insert( posBoxes.end(), midIdx, negBoxes.end() );
      negBoxes.erase( midIdx, negBoxes.end() );
    }

    #ifdef G2LIB_USE_CXX11
    PtrAABB neg = make_shared<AABBtree>();
    PtrAABB pos = make_shared<AABBtree>();
    #else
    PtrAABB neg = new AABBtree();
    PtrAABB pos = new AABBtree();
    #endif

    neg->build(negBoxes);
    if (!neg->empty()) children.push_back(neg);

    pos->build(posBoxes);
    if (!pos->empty()) children.push_back(pos);
  }

  // . . . . . . . . . . . . . . . . . . . . . . . . . . . . . . . . . . . . . .

  void
  AABBtree::print( ostream_type & stream, int level ) const {
    if ( empty() ) {
      stream << "[EMPTY AABB tree]\n";
    } else {
      fmt::print( stream,
        "BBOX xmin={:<12.4)} ymin={:<12.4)} xmax={:<12.4)} ymax={:<12.4)} level={}\n",
        pBBox->m_xmin, pBBox->m_ymin, pBBox->m_xmax, pBBox->m_ymax
      );
      vector<PtrAABB>::const_iterator it;
      for ( it = children.begin(); it != children.end(); ++it )
        (*it)->print( stream, level+1 );
    }
  }

  // . . . . . . . . . . . . . . . . . . . . . . . . . . . . . . . . . . . . . .

  void
  AABBtree::intersect(
    AABBtree const & tree,
    VecPairPtrBBox & intersectionList,
    bool             swap_tree
  ) const {

    // check bbox with
    if ( !tree.pBBox->collision(*pBBox) ) return;

    int icase = (children.empty() ? 0 : 1) +
                (tree.children.empty()? 0 : 2);

    switch ( icase ) {
    case 0: // both leaf
      if ( swap_tree )
        intersectionList.push_back( PairPtrBBox(tree.pBBox,pBBox ) );
      else
        intersectionList.push_back( PairPtrBBox(pBBox,tree.pBBox ) );
      break;
    case 1: // first is a tree, second is a leaf
      { vector<PtrAABB>::const_iterator it;
        for ( it = children.begin(); it != children.end(); ++it )
          tree.intersect( **it, intersectionList, !swap_tree );
      }
      break;
    case 2: // first leaf, second is a tree
      { vector<PtrAABB>::const_iterator it;
        for ( it = tree.children.begin(); it != tree.children.end(); ++it )
          this->intersect( **it, intersectionList, swap_tree );
      }
      break;
    case 3: // first is a tree, second is a tree
      { vector<PtrAABB>::const_iterator c1;
        vector<PtrAABB>::const_iterator c2;
        for ( c1 = children.begin(); c1 != children.end(); ++c1 )
          for ( c2 = tree.children.begin(); c2 != tree.children.end(); ++c2 )
            (*c1)->intersect( **c2, intersectionList, swap_tree );
      }
      break;
    }
  }

  // . . . . . . . . . . . . . . . . . . . . . . . . . . . . . . . . . . . . . .

  real_type
  AABBtree::min_maxdist(
    real_type        x,
    real_type        y,
    AABBtree const & tree,
    real_type        mmDist
  ) {

    vector<PtrAABB> const & children = tree.children;

    if ( children.empty() ) {
      real_type dst = tree.pBBox->maxDistance( x, y );
      return min( dst, mmDist );
    }

    real_type dmin = tree.pBBox->distance( x, y );
    if ( dmin > mmDist ) return mmDist;

    // check bbox with
    vector<PtrAABB>::const_iterator it;
    for ( it = children.begin(); it != children.end(); ++it )
      mmDist = min_maxdist( x, y, **it, mmDist );

    return mmDist;
  }

  // . . . . . . . . . . . . . . . . . . . . . . . . . . . . . . . . . . . . . .

  void
  AABBtree::min_maxdist_select(
    real_type        x,
    real_type        y,
    real_type        mmDist,
    AABBtree const & tree,
    VecPtrBBox     & candidateList
  ) {
    vector<PtrAABB> const & children = tree.children;
    real_type dst = tree.pBBox->distance( x, y );
    if ( dst <= mmDist ) {
      if ( children.empty() ) {
        candidateList.push_back( tree.pBBox );
      } else {
        // check bbox with
        vector<PtrAABB>::const_iterator it;
        for ( it = children.begin(); it != children.end(); ++it )
          min_maxdist_select( x, y, mmDist, **it, candidateList );
      }
    }
  }

  // . . . . . . . . . . . . . . . . . . . . . . . . . . . . . . . . . . . . . .

  void
  AABBtree::min_distance(
    real_type    x,
    real_type    y,
    VecPtrBBox & candidateList
  ) const {
    real_type mmDist = min_maxdist(
      x, y, *this, numeric_limits<real_type>::infinity()
    );
    min_maxdist_select( x, y, mmDist, *this, candidateList );
  }

}
```

### Quick search

### Table of Contents

- Matlab Interface Manual
- C++ API
- MATLAB API

«
hide menu

menu
sidebar
»

### Navigation

- index
- toc
- Clothoids »
- Program Listing for File AABBtree.cc

© Copyright 2021, Enrico Bertolazzi and Marco Frego.
Created using Sphinx 4.2.0.
