# Supplementary material for: Smooth Interpolating Curves with Local Control and Monotone Alternating Curvature
Source: Comput Graph Forum. 2022 Oct 6;41(5):25–38. doi: 10.1111/cgf.14600 (PMC9827861; doi:10.1111/cgf.14600)
Supplement: Supplementary file 1 — Supplement Material [file CGF-41-25-s001.zip › Local-Smooth-Interpolating-MonoCurvature/extern/clothoids/docs/api-cpp/program_listing_file_Biarc.cc.html]

Program Listing for File Biarc.cc — Clothoids v2.0.9

### Navigation

- index
- toc
- Clothoids »
- Program Listing for File Biarc.cc

# Program Listing for File Biarc.cc¶

↰ Return to documentation for file (`Biarc.cc`)

```
/*--------------------------------------------------------------------------*\
 |                                                                          |
 |  Copyright (C) 2017                                                      |
 |                                                                          |
 |         , __                 , __                                        |
 |        /|/  \               /|/  \                                       |
 |         | __/ _   ,_         | __/ _   ,_                                |
 |         |   \|/  /  |  |   | |   \|/  /  |  |   |                        |
 |         |(__/|__/   |_/ \_/|/|(__/|__/   |_/ \_/|/                       |
 |                           /|                   /|                        |
 |                           \|                   \|                        |
 |                                                                          |
 |      Enrico Bertolazzi                                                   |
 |      Dipartimento di Ingegneria Industriale                              |
 |      Universita` degli Studi di Trento                                   |
 |      email: enrico.bertolazzi@unitn.it                                   |
 |                                                                          |
\*--------------------------------------------------------------------------*/


#include "Clothoids.hh"

namespace G2lib {

  using std::numeric_limits;
  using std::abs;

  /*\
   |   ____  _
   |  | __ )(_) __ _ _ __ ___
   |  |  _ \| |/ _` | '__/ __|
   |  | |_) | | (_| | | | (__
   |  |____/|_|\__,_|_|  \___|
  \*/

  Biarc::Biarc( BaseCurve const & C )
  : BaseCurve(G2LIB_BIARC)
  {
    switch ( C.type() ) {
    case G2LIB_LINE:
      {
        LineSegment const & LS = *static_cast<LineSegment const *>(&C);
        bool ok = this->build(
          LS.xBegin(), LS.yBegin(), LS.thetaBegin(),
          LS.xEnd(),   LS.yEnd(),   LS.thetaEnd()
        );
        UTILS_ASSERT(
          ok,
          "Biarc constructor failed convert from: {}\n",
          CurveType_name[C.type()]
        );
      }
      break;
    case G2LIB_CIRCLE:
      {
        CircleArc const & LS = *static_cast<CircleArc const *>(&C);
        bool ok = this->build(
          LS.xBegin(), LS.yBegin(), LS.thetaBegin(),
          LS.xEnd(),   LS.yEnd(),   LS.thetaEnd()
        );
        UTILS_ASSERT(
          ok,
          "Biarc constructor failed convert from: {}\n",
          CurveType_name[C.type()]
        );
      }
      break;
    case G2LIB_BIARC:
      *this = *static_cast<Biarc const *>(&C);
      break;
    case G2LIB_CLOTHOID:
    case G2LIB_BIARC_LIST:
    case G2LIB_CLOTHOID_LIST:
    case G2LIB_POLYLINE:
      UTILS_ERROR(
        "Biarc constructor cannot convert from: {}\n",
        CurveType_name[C.type()]
      );
    }
  }

  bool
  Biarc::build(
    real_type x0,
    real_type y0,
    real_type theta0,
    real_type x1,
    real_type y1,
    real_type theta1
  ) {

    real_type dx = x1-x0;
    real_type dy = y1-y0;
    real_type d  = hypot(dy,dx);

    real_type omega = atan2(dy,dx);

    // put in range
    real_type th0 = theta0 - omega;
    real_type th1 = theta1 - omega;

    rangeSymm(th0);
    rangeSymm(th1);

    real_type thstar = - (th0+th1)/2;

    real_type dth  = (th1 - th0)/4;
    real_type dth0 = (thstar - th0)/2;
    real_type dth1 = (thstar - th1)/2;

    real_type t  = 2 * cos( dth ) / d;
    real_type l0 = 1/(t*Sinc( dth0 ));
    real_type l1 = 1/(t*Sinc( dth1 ));
    real_type k0 = 2*t*sin( dth0 );
    real_type k1 = -2*t*sin( dth1 );

    real_type epsi = 100*d*numeric_limits<real_type>::epsilon();
    if ( l0 > epsi && l1 > epsi ) {

      m_C0.build( x0, y0, theta0, k0, l0 );

      real_type an = omega+(thstar+th0)/2;
      real_type xs = x0 + cos(an)/t;
      real_type ys = y0 + sin(an)/t;

      m_C1.build( xs, ys, omega+thstar, k1, l1 );
      return true;
    }
    return false;
  }

  // - - - - - - - - - - - - - - - - - - - - - - - - - - - - - - - - - - - - - -

  bool
  Biarc::build_3P(
    real_type x0,
    real_type y0,
    real_type x1,
    real_type y1,
    real_type x2,
    real_type y2
  ) {

    real_type dxa   = x1-x0;
    real_type dya   = y1-y0;
    real_type dxb   = x2-x1;
    real_type dyb   = y2-y1;
    real_type La    = hypot(dya,dxa);
    real_type Lb    = hypot(dyb,dxb);
    real_type arg   = (dxa*dxb + dya * dyb)/(La*Lb);
    if      ( arg >  1 ) arg = 1;
    else if ( arg < -1 ) arg = -1;
    real_type om = acos(arg);

    real_type at = (La/(La+Lb))*om;
    real_type bt = (Lb/(La+Lb))*om;
    // find solution using Halley
    real_type Delta = 0;
    bool found = false;
    for ( int_type iter = 0; iter < 10 && !found; ++iter ) {
      real_type ga[3], gb[3];
      gfun( at+Delta, ga );
      gfun( bt-Delta, gb );
      real_type f   = ga[0]/La - gb[0]/Lb;
      real_type df  = ga[1]/La + gb[1]/Lb;
      real_type ddf = ga[2]/La - gb[2]/Lb;
      real_type h   = (df*f)/(df*df-0.5*f*ddf);
      Delta -= h;
      found = abs(h) < 1e-10 && abs(f) < 1e-10;
    }

    if ( found ) {
      at += Delta; bt -= Delta;
      real_type tha = atan2(dya,dxa);
      real_type thb = atan2(dyb,dxb);
      if ( dxa*dyb < dya*dxb ) {
        tha += at;
        thb += bt;
      } else {
        tha -= at;
        thb -= bt;
      }
      m_C0.build_G1( x0, y0, tha, x1, y1 );
      m_C1.build_G1( x1, y1, thb, x2, y2 );
    }

    return found;
  }

  // - - - - - - - - - - - - - - - - - - - - - - - - - - - - - - - - - - - - - -

  void
  Biarc::bbox(
    real_type & xmin,
    real_type & ymin,
    real_type & xmax,
    real_type & ymax
  ) const {
    m_C0.bbox( xmin, ymin, xmax, ymax );
    real_type xmi1, ymi1, xma1, yma1;
    m_C1.bbox( xmi1, ymi1, xma1, yma1 );
    if ( xmi1 < xmin ) xmin = xmi1;
    if ( xma1 > xmax ) xmax = xma1;
    if ( ymi1 < ymin ) ymin = ymi1;
    if ( yma1 > ymax ) ymax = yma1;
  }

  // - - - - - - - - - - - - - - - - - - - - - - - - - - - - - - - - - - - - - -

  void
  Biarc::bbox_ISO(
    real_type   offs,
    real_type & xmin,
    real_type & ymin,
    real_type & xmax,
    real_type & ymax
  ) const {
    m_C0.bbox_ISO( offs, xmin, ymin, xmax, ymax );
    real_type xmi1, ymi1, xma1, yma1;
    m_C1.bbox_ISO( offs, xmi1, ymi1, xma1, yma1 );
    if ( xmi1 < xmin ) xmin = xmi1;
    if ( xma1 > xmax ) xmax = xma1;
    if ( ymi1 < ymin ) ymin = ymi1;
    if ( yma1 > ymax ) ymax = yma1;
  }

  /*\
   |  _                        __
   | | |_ _ __ __ _ _ __  ___ / _| ___  _ __ _ __ ___
   | | __| '__/ _` | '_ \/ __| |_ / _ \| '__| '_ ` _ \
   | | |_| | | (_| | | | \__ \  _| (_) | |  | | | | | |
   |  \__|_|  \__,_|_| |_|___/_|  \___/|_|  |_| |_| |_|
  \*/

  void
  Biarc::reverse() {
    CircleArc TMP(m_C0);
    m_C0 = m_C1; m_C0.reverse();
    m_C1 = TMP;  m_C1.reverse();
  }

  // - - - - - - - - - - - - - - - - - - - - - - - - - - - - - - - - - - - - - -

  void
  Biarc::scale( real_type scl ) {
    real_type newx0 = m_C0.xBegin() + scl*(m_C1.xBegin()-m_C0.xBegin());
    real_type newy0 = m_C0.yBegin() + scl*(m_C1.yBegin()-m_C0.yBegin());
    m_C1.changeOrigin( newx0, newy0 );
    m_C1.scale( scl );
    m_C0.scale( scl );
  }

  void
  Biarc::changeOrigin( real_type newx0, real_type newy0 ) {
    m_C0.changeOrigin(newx0,newy0);
    m_C1.changeOrigin(m_C0.xEnd(),m_C0.yEnd());
  }

  void
  Biarc::trim( real_type s_begin, real_type s_end ) {
    UTILS_ASSERT(
      s_end > s_begin,
      "Biarc::trim( begin={}, s_end={} ) s_end must be > s_begin\n",
      s_begin, s_end
    );
    real_type L0 = m_C0.length();
    if ( s_end <= L0 ) {
      m_C0.trim( s_begin, s_end );
      m_C1 = m_C0;
      real_type ss = m_C0.length();
      m_C0.trim( 0, ss/2 );
      m_C1.trim( ss/2, ss );
    } else if ( s_begin >= L0 ) {
      m_C1.trim( s_begin-L0, s_end-L0 );
      m_C0 = m_C1;
      real_type ss = m_C0.length();
      m_C0.trim( 0, ss/2 );
      m_C1.trim( ss/2, ss );
    } else {
      m_C0.trim( s_begin, L0 );
      m_C1.trim( 0, s_end-L0 );
    }
  }

  // - - - - - - - - - - - - - - - - - - - - - - - - - - - - - - - - - - - - - -

  real_type
  Biarc::theta( real_type s ) const {
    real_type L0 = m_C0.length();
    if ( s < L0 ) return m_C0.theta(s);
    else          return m_C1.theta(s-L0);
  }

  // - - - - - - - - - - - - - - - - - - - - - - - - - - - - - - - - - - - - - -

  real_type
  Biarc::theta_D( real_type s ) const {
    real_type L0 = m_C0.length();
    if ( s < L0 ) return m_C0.m_k;
    else          return m_C1.m_k;
  }

  // - - - - - - - - - - - - - - - - - - - - - - - - - - - - - - - - - - - - - -

  real_type
  Biarc::tx( real_type s ) const {
    real_type L0 = m_C0.length();
    if ( s < L0 ) return m_C0.tx(s);
    else          return m_C1.tx(s-L0);
  }

  // - - - - - - - - - - - - - - - - - - - - - - - - - - - - - - - - - - - - - -

  real_type
  Biarc::tx_D( real_type s ) const {
    real_type L0 = m_C0.length();
    if ( s < L0 ) return m_C0.tx_D(s);
    else          return m_C1.tx_D(s-L0);
  }

  // - - - - - - - - - - - - - - - - - - - - - - - - - - - - - - - - - - - - - -

  real_type
  Biarc::tx_DD( real_type s ) const {
    real_type L0 = m_C0.length();
    if ( s < L0 ) return m_C0.tx_DD(s);
    else          return m_C1.tx_DD(s-L0);
  }

  // - - - - - - - - - - - - - - - - - - - - - - - - - - - - - - - - - - - - - -

  real_type
  Biarc::tx_DDD( real_type s ) const {
    real_type L0 = m_C0.length();
    if ( s < L0 ) return m_C0.tx_DDD(s);
    else          return m_C1.tx_DDD(s-L0);
  }

  // - - - - - - - - - - - - - - - - - - - - - - - - - - - - - - - - - - - - - -

  real_type
  Biarc::ty( real_type s ) const {
    real_type L0 = m_C0.length();
    if ( s < L0 ) return m_C0.ty(s);
    else          return m_C1.ty(s-L0);
  }

  // - - - - - - - - - - - - - - - - - - - - - - - - - - - - - - - - - - - - - -

  real_type
  Biarc::ty_D( real_type s ) const {
    real_type L0 = m_C0.length();
    if ( s < L0 ) return m_C0.ty_D(s);
    else          return m_C1.ty_D(s-L0);
  }

  // - - - - - - - - - - - - - - - - - - - - - - - - - - - - - - - - - - - - - -

  real_type
  Biarc::ty_DD( real_type s ) const {
    real_type L0 = m_C0.length();
    if ( s < L0 ) return m_C0.ty_DD(s);
    else          return m_C1.ty_DD(s-L0);
  }

  // - - - - - - - - - - - - - - - - - - - - - - - - - - - - - - - - - - - - - -

  real_type
  Biarc::ty_DDD( real_type s ) const {
    real_type L0 = m_C0.length();
    if ( s < L0 ) return m_C0.ty_DDD(s);
    else          return m_C1.ty_DDD(s-L0);
  }

  // - - - - - - - - - - - - - - - - - - - - - - - - - - - - - - - - - - - - - -

  real_type
  Biarc::X( real_type s ) const {
    real_type L0 = m_C0.length();
    if ( s < L0 ) return m_C0.X(s);
    else          return m_C1.X(s-L0);
  }

  // - - - - - - - - - - - - - - - - - - - - - - - - - - - - - - - - - - - - - -

  real_type
  Biarc::X_D( real_type s ) const {
    real_type L0 = m_C0.length();
    if ( s < L0 ) return m_C0.X_D(s);
    else          return m_C1.X_D(s-L0);
  }

  // - - - - - - - - - - - - - - - - - - - - - - - - - - - - - - - - - - - - - -

  real_type
  Biarc::X_DD( real_type s ) const {
    real_type L0 = m_C0.length();
    if ( s < L0 ) return m_C0.X_DD(s);
    else          return m_C1.X_DD(s-L0);
  }

  // - - - - - - - - - - - - - - - - - - - - - - - - - - - - - - - - - - - - - -

  real_type
  Biarc::X_DDD( real_type s ) const {
    real_type L0 = m_C0.length();
    if ( s < L0 ) return m_C0.X_DDD(s);
    else          return m_C1.X_DDD(s-L0);
  }

  // - - - - - - - - - - - - - - - - - - - - - - - - - - - - - - - - - - - - - -

  real_type
  Biarc::Y( real_type s ) const {
    real_type L0 = m_C0.length();
    if ( s < L0 ) return m_C0.Y(s);
    else          return m_C1.Y(s-L0);
  }

  // - - - - - - - - - - - - - - - - - - - - - - - - - - - - - - - - - - - - - -

  real_type
  Biarc::Y_D( real_type s ) const {
    real_type L0 = m_C0.length();
    if ( s < L0 ) return m_C0.Y_D(s);
    else          return m_C1.Y_D(s-L0);
  }

  // - - - - - - - - - - - - - - - - - - - - - - - - - - - - - - - - - - - - - -

  real_type
  Biarc::Y_DD( real_type s ) const {
    real_type L0 = m_C0.length();
    if ( s < L0 ) return m_C0.Y_DD(s);
    else          return m_C1.Y_DD(s-L0);
  }

  // - - - - - - - - - - - - - - - - - - - - - - - - - - - - - - - - - - - - - -

  real_type
  Biarc::Y_DDD( real_type s ) const {
    real_type L0 = m_C0.length();
    if ( s < L0 ) return m_C0.Y_DDD(s);
    else          return m_C1.Y_DDD(s-L0);
  }

  // - - - - - - - - - - - - - - - - - - - - - - - - - - - - - - - - - - - - - -

  real_type
  Biarc::X_ISO( real_type s, real_type offs ) const {
    real_type L0 = m_C0.length();
    if ( s < L0 ) return m_C0.X_ISO(s,offs);
    else          return m_C1.X_ISO(s-L0,offs);
  }

  // - - - - - - - - - - - - - - - - - - - - - - - - - - - - - - - - - - - - - -

  real_type
  Biarc::X_ISO_D( real_type s, real_type offs ) const {
    real_type L0 = m_C0.length();
    if ( s < L0 ) return m_C0.X_ISO_D(s,offs);
    else          return m_C1.X_ISO_D(s-L0,offs);
  }

  // - - - - - - - - - - - - - - - - - - - - - - - - - - - - - - - - - - - - - -

  real_type
  Biarc::X_ISO_DD( real_type s, real_type offs ) const {
    real_type L0 = m_C0.length();
    if ( s < L0 ) return m_C0.X_ISO_DD(s,offs);
    else          return m_C1.X_ISO_DD(s-L0,offs);
  }

  // - - - - - - - - - - - - - - - - - - - - - - - - - - - - - - - - - - - - - -

  real_type
  Biarc::X_ISO_DDD( real_type s, real_type offs ) const {
    real_type L0 = m_C0.length();
    if ( s < L0 ) return m_C0.X_ISO_DDD(s,offs);
    else          return m_C1.X_ISO_DDD(s-L0,offs);
  }

  // - - - - - - - - - - - - - - - - - - - - - - - - - - - - - - - - - - - - - -

  real_type
  Biarc::Y_ISO( real_type s, real_type offs ) const {
    real_type L0 = m_C0.length();
    if ( s < L0 ) return m_C0.Y_ISO(s,offs);
    else          return m_C1.Y_ISO(s-L0,offs);
  }

  // - - - - - - - - - - - - - - - - - - - - - - - - - - - - - - - - - - - - - -

  real_type
  Biarc::Y_ISO_D( real_type s, real_type offs ) const {
    real_type L0 = m_C0.length();
    if ( s < L0 ) return m_C0.Y_ISO_D(s,offs);
    else          return m_C1.Y_ISO_D(s-L0,offs);
  }

  // - - - - - - - - - - - - - - - - - - - - - - - - - - - - - - - - - - - - - -

  real_type
  Biarc::Y_ISO_DD( real_type s, real_type offs ) const {
    real_type L0 = m_C0.length();
    if ( s < L0 ) return m_C0.Y_ISO_DD(s,offs);
    else          return m_C1.Y_ISO_DD(s-L0,offs);
  }

  // - - - - - - - - - - - - - - - - - - - - - - - - - - - - - - - - - - - - - -

  real_type
  Biarc::Y_ISO_DDD( real_type s, real_type offs ) const {
    real_type L0 = m_C0.length();
    if ( s < L0 ) return m_C0.Y_ISO_DDD(s,offs);
    else          return m_C1.Y_ISO_DDD(s-L0,offs);
  }

  // - - - - - - - - - - - - - - - - - - - - - - - - - - - - - - - - - - - - - -

  void
  Biarc::tg( real_type s, real_type & tx, real_type & ty ) const {
    real_type L0 = m_C0.length();
    if ( s < L0 ) return m_C0.tg(s,tx,ty);
    else          return m_C1.tg(s-L0,tx,ty);
  }

  // - - - - - - - - - - - - - - - - - - - - - - - - - - - - - - - - - - - - - -

  void
  Biarc::tg_D( real_type s, real_type & tx_D, real_type & ty_D ) const {
    real_type L0 = m_C0.length();
    if ( s < L0 ) return m_C0.tg_D(s,tx_D,ty_D);
    else          return m_C1.tg_D(s-L0,tx_D,ty_D);
  }

  // - - - - - - - - - - - - - - - - - - - - - - - - - - - - - - - - - - - - - -

  void
  Biarc::tg_DD( real_type s, real_type & tx_DD, real_type & ty_DD ) const {
    real_type L0 = m_C0.length();
    if ( s < L0 ) return m_C0.tg_DD(s,tx_DD,ty_DD);
    else          return m_C1.tg_DD(s-L0,tx_DD,ty_DD);
  }

  // - - - - - - - - - - - - - - - - - - - - - - - - - - - - - - - - - - - - - -

  void
  Biarc::tg_DDD( real_type s, real_type & tx_DDD, real_type & ty_DDD ) const {
    real_type L0 = m_C0.length();
    if ( s < L0 ) return m_C0.tg_DDD(s,tx_DDD,ty_DDD);
    else          return m_C1.tg_DDD(s-L0,tx_DDD,ty_DDD);
  }

  // - - - - - - - - - - - - - - - - - - - - - - - - - - - - - - - - - - - - - -

  void
  Biarc::evaluate(
    real_type   s,
    real_type & th,
    real_type & k,
    real_type & x,
    real_type & y
  ) const {
    if ( s < m_C0.length() ) {
      th = m_C0.theta(s);
      k  = m_C0.curvature();
      m_C0.eval(s,x,y);
    } else {
      s -= m_C0.length();
      th = m_C1.theta(s);
      k  = m_C1.curvature();
      m_C1.eval(s,x,y);
    }
  }

  // - - - - - - - - - - - - - - - - - - - - - - - - - - - - - - - - - - - - - -

  void
  Biarc::eval(
    real_type   s,
    real_type & x,
    real_type & y
  ) const {
    if ( s < m_C0.length() ) {
      m_C0.eval(s,x,y);
    } else {
      s -= m_C0.length();
      m_C1.eval(s,x,y);
    }
  }

  // - - - - - - - - - - - - - - - - - - - - - - - - - - - - - - - - - - - - - -

  void
  Biarc::eval_D(
    real_type   s,
    real_type & x_D,
    real_type & y_D
  ) const {
    if ( s < m_C0.length() ) {
      m_C0.eval_D(s,x_D,y_D);
    } else {
      s -= m_C0.length();
      m_C1.eval_D(s,x_D,y_D);
    }
  }

  // - - - - - - - - - - - - - - - - - - - - - - - - - - - - - - - - - - - - - -

  void
  Biarc::eval_DD(
    real_type   s,
    real_type & x_DD,
    real_type & y_DD
  ) const {
    if ( s < m_C0.length() ) {
      m_C0.eval_DD(s,x_DD,y_DD);
    } else {
      s -= m_C0.length();
      m_C1.eval_DD(s,x_DD,y_DD);
    }
  }

  // - - - - - - - - - - - - - - - - - - - - - - - - - - - - - - - - - - - - - -

  void
  Biarc::eval_DDD(
    real_type   s,
    real_type & x_DDD,
    real_type & y_DDD
  ) const {
    if ( s < m_C0.length() ) {
      m_C0.eval_DDD(s,x_DDD,y_DDD);
    } else {
      s -= m_C0.length();
      m_C1.eval_DDD(s,x_DDD,y_DDD);
    }
  }

  // - - - - - - - - - - - - - - - - - - - - - - - - - - - - - - - - - - - - - -

  void
  Biarc::eval_ISO(
    real_type   s,
    real_type   offs,
    real_type & x,
    real_type & y
  ) const {
    if ( s < m_C0.length() ) {
      m_C0.eval_ISO(s,offs,x,y);
    } else {
      s -= m_C0.length();
      m_C1.eval_ISO(s,offs,x,y);
    }
  }

  // - - - - - - - - - - - - - - - - - - - - - - - - - - - - - - - - - - - - - -

  void
  Biarc::eval_ISO_D(
    real_type   s,
    real_type   offs,
    real_type & x_D,
    real_type & y_D
  ) const {
    if ( s < m_C0.length() ) {
      m_C0.eval_ISO_D(s,offs,x_D,y_D);
    } else {
      s -= m_C0.length();
      m_C1.eval_ISO_D(s,offs,x_D,y_D);
    }
  }

  // - - - - - - - - - - - - - - - - - - - - - - - - - - - - - - - - - - - - - -

  void
  Biarc::eval_ISO_DD(
    real_type   s,
    real_type   offs,
    real_type & x_DD,
    real_type & y_DD
  ) const {
    if ( s < m_C0.length() ) {
      m_C0.eval_ISO_DD(s,offs,x_DD,y_DD);
    } else {
      s -= m_C0.length();
      m_C1.eval_ISO_DD(s,offs,x_DD,y_DD);
    }
  }

  // - - - - - - - - - - - - - - - - - - - - - - - - - - - - - - - - - - - - - -

  void
  Biarc::eval_ISO_DDD(
    real_type   s,
    real_type   offs,
    real_type & x_DDD,
    real_type & y_DDD
  ) const {
    if ( s < m_C0.length() ) {
      m_C0.eval_ISO_DDD(s,offs,x_DDD,y_DDD);
    } else {
      s -= m_C0.length();
      m_C1.eval_ISO_DDD(s,offs,x_DDD,y_DDD);
    }
  }

  /*\
   |   _       _                          _
   |  (_)_ __ | |_ ___ _ __ ___  ___  ___| |_
   |  | | '_ \| __/ _ \ '__/ __|/ _ \/ __| __|
   |  | | | | | ||  __/ |  \__ \  __/ (__| |_
   |  |_|_| |_|\__\___|_|  |___/\___|\___|\__|
  \*/

  // - - - - - - - - - - - - - - - - - - - - - - - - - - - - - - - - - - - - - -

  void
  Biarc::intersect(
    Biarc const   & B,
    IntersectList & ilist,
    bool            swap_s_vals
  ) const {
    IntersectList ilist00, ilist01, ilist10, ilist11;
    m_C0.intersect( B.m_C0, ilist00, false );
    m_C0.intersect( B.m_C1, ilist01, false );
    m_C1.intersect( B.m_C0, ilist10, false );
    m_C1.intersect( B.m_C1, ilist11, false );
    real_type L  = m_C0.length();
    real_type LB = B.m_C0.length();
    IntersectList::iterator it;
    ilist.reserve( ilist.size() +
                   ilist00.size() +
                   ilist01.size() +
                   ilist10.size() +
                   ilist11.size() );
    for ( it = ilist01.begin(); it != ilist01.end(); ++it ) it->second += LB;
    for ( it = ilist10.begin(); it != ilist10.end(); ++it ) it->first  += L;
    for ( it = ilist11.begin(); it != ilist11.end(); ++it )
      { it->first += L; it->second += LB; }

    if ( swap_s_vals ) {
      for ( it = ilist00.begin(); it != ilist00.end(); ++it )
        ilist.push_back( Ipair(it->second,it->first) );
      for ( it = ilist01.begin(); it != ilist01.end(); ++it )
        ilist.push_back( Ipair(it->second,it->first) );
      for ( it = ilist10.begin(); it != ilist10.end(); ++it )
        ilist.push_back( Ipair(it->second,it->first) );
      for ( it = ilist11.begin(); it != ilist11.end(); ++it )
        ilist.push_back( Ipair(it->second,it->first) );
    } else {
      for ( it = ilist00.begin(); it != ilist00.end(); ++it ) ilist.push_back( *it );
      for ( it = ilist01.begin(); it != ilist01.end(); ++it ) ilist.push_back( *it );
      for ( it = ilist10.begin(); it != ilist10.end(); ++it ) ilist.push_back( *it );
      for ( it = ilist11.begin(); it != ilist11.end(); ++it ) ilist.push_back( *it );
    }
  }

  // - - - - - - - - - - - - - - - - - - - - - - - - - - - - - - - - - - - - - -

  void
  Biarc::intersect_ISO(
    real_type       offs,
    Biarc const   & B,
    real_type       offs_B,
    IntersectList & ilist,
    bool            swap_s_vals
  ) const {
    IntersectList ilist00, ilist01, ilist10, ilist11;
    m_C0.intersect_ISO( offs, B.m_C0, offs_B, ilist00, false );
    m_C0.intersect_ISO( offs, B.m_C1, offs_B, ilist01, false );
    m_C1.intersect_ISO( offs, B.m_C0, offs_B, ilist10, false );
    m_C1.intersect_ISO( offs, B.m_C1, offs_B, ilist11, false );
    real_type L  = m_C0.length();
    real_type LB = B.m_C0.length();
    IntersectList::iterator it;
    ilist.reserve( ilist.size() +
                   ilist00.size() +
                   ilist01.size() +
                   ilist10.size() +
                   ilist11.size() );
    for ( it = ilist01.begin(); it != ilist01.end(); ++it ) it->second += LB;
    for ( it = ilist10.begin(); it != ilist10.end(); ++it ) it->first  += L;
    for ( it = ilist11.begin(); it != ilist11.end(); ++it )
      { it->first += L; it->second += LB; }

    if ( swap_s_vals ) {
      for ( it = ilist00.begin(); it != ilist00.end(); ++it )
        ilist.push_back( Ipair(it->second,it->first) );
      for ( it = ilist01.begin(); it != ilist01.end(); ++it )
        ilist.push_back( Ipair(it->second,it->first) );
      for ( it = ilist10.begin(); it != ilist10.end(); ++it )
        ilist.push_back( Ipair(it->second,it->first) );
      for ( it = ilist11.begin(); it != ilist11.end(); ++it )
        ilist.push_back( Ipair(it->second,it->first) );
    } else {
      for ( it = ilist00.begin(); it != ilist00.end(); ++it ) ilist.push_back( *it );
      for ( it = ilist01.begin(); it != ilist01.end(); ++it ) ilist.push_back( *it );
      for ( it = ilist10.begin(); it != ilist10.end(); ++it ) ilist.push_back( *it );
      for ( it = ilist11.begin(); it != ilist11.end(); ++it ) ilist.push_back( *it );
    }
  }

  // - - - - - - - - - - - - - - - - - - - - - - - - - - - - - - - - - - - - - -

  int_type
  Biarc::closestPoint_ISO(
    real_type   qx,
    real_type   qy,
    real_type & x,
    real_type & y,
    real_type & s,
    real_type & t,
    real_type & dst
  ) const {
    real_type x1, y1, s1, t1, dst1;
    int_type res  = m_C0.closestPoint_ISO( qx, qy, x,  y,  s,  t,  dst  );
    int_type res1 = m_C1.closestPoint_ISO( qx, qy, x1, y1, s1, t1, dst1 );
    if ( dst1 < dst ) {
      x   = x1;
      y   = y1;
      s   = s1;
      t   = t1;
      dst = dst1;
      res = res1;
    }
    return res;
  }

  // - - - - - - - - - - - - - - - - - - - - - - - - - - - - - - - - - - - - - -

  int_type
  Biarc::closestPoint_ISO(
    real_type   qx,
    real_type   qy,
    real_type   offs,
    real_type & x,
    real_type & y,
    real_type & s,
    real_type & t,
    real_type & dst
  ) const {
    real_type x1, y1, s1, t1, dst1;
    int_type res  = m_C0.closestPoint_ISO( qx, qy, offs, x,  y,  s,  t,  dst  );
    int_type res1 = m_C1.closestPoint_ISO( qx, qy, offs, x1, y1, s1, t1, dst1 );
    if ( dst1 < dst ) {
      x   = x1;
      y   = y1;
      s   = s1;
      t   = t1;
      dst = dst1;
      res = res1;
    }
    return res;
  }

  // - - - - - - - - - - - - - - - - - - - - - - - - - - - - - - - - - - - - - -

  bool
  build_guess_theta(
    int_type          n,
    real_type const * x,
    real_type const * y,
    real_type       * theta
  ) {
    UTILS_ASSERT0(
      n > 1, "build_guess_theta, at least 2 points are necessary\n"
    );
    Biarc b;
    if ( n == 2 ) {
      theta[0] = theta[1] = atan2( y[1] - y[0], x[1] - x[0] );
    } else {
      bool ok, ciclic = hypot( x[0]-x[n-1], y[0]-y[n-1] ) < 1e-10;
      if ( ciclic ) {
        ok = b.build_3P( x[n-2], y[n-2], x[0], y[0], x[1], y[1] );
        UTILS_ASSERT0( ok, "build_guess_theta, failed\n" );
        theta[0] = theta[n-1] = b.thetaMiddle();
      }
      for ( int_type k = 1; k < n-1; ++k ) {
        ok = b.build_3P( x[k-1], y[k-1], x[k], y[k], x[k+1], y[k+1] );
        UTILS_ASSERT0( ok, "build_guess_theta, failed\n" );
        theta[k] = b.thetaMiddle();
        if ( k == 1   && !ciclic ) theta[0]   = b.thetaBegin();
        if ( k == n-2 && !ciclic ) theta[n-1] = b.thetaEnd();
      }
    }
    return true;
  }

  // - - - - - - - - - - - - - - - - - - - - - - - - - - - - - - - - - - - - - -

  ostream_type &
  operator << ( ostream_type & stream, Biarc const & bi ) {
    stream
      << "C0\n" << bi.m_C0
      << "C1\n" << bi.m_C1
      << "\n";
    return stream;
  }

}
```

### Quick search

### Table of Contents

- Matlab Interface Manual
- C++ API
- MATLAB API

«
hide menu

menu
sidebar
»

### Navigation

- index
- toc
- Clothoids »
- Program Listing for File Biarc.cc

© Copyright 2021, Enrico Bertolazzi and Marco Frego.
Created using Sphinx 4.2.0.
