# Supplementary material for: Smooth Interpolating Curves with Local Control and Monotone Alternating Curvature
Source: Comput Graph Forum. 2022 Oct 6;41(5):25–38. doi: 10.1111/cgf.14600 (PMC9827861; doi:10.1111/cgf.14600)
Supplement: Supplementary file 1 — Supplement Material [file CGF-41-25-s001.zip › Local-Smooth-Interpolating-MonoCurvature/extern/clothoids/docs/api-cpp/program_listing_file_BiarcList.cc.html]

Program Listing for File BiarcList.cc — Clothoids v2.0.9

### Navigation

- index
- toc
- Clothoids »
- Program Listing for File BiarcList.cc

# Program Listing for File BiarcList.cc¶

↰ Return to documentation for file (`BiarcList.cc`)

```
/*--------------------------------------------------------------------------*\
 |                                                                          |
 |  Copyright (C) 2017                                                      |
 |                                                                          |
 |         , __                 , __                                        |
 |        /|/  \               /|/  \                                       |
 |         | __/ _   ,_         | __/ _   ,_                                |
 |         |   \|/  /  |  |   | |   \|/  /  |  |   |                        |
 |         |(__/|__/   |_/ \_/|/|(__/|__/   |_/ \_/|/                       |
 |                           /|                   /|                        |
 |                           \|                   \|                        |
 |                                                                          |
 |      Enrico Bertolazzi                                                   |
 |      Dipartimento di Ingegneria Industriale                              |
 |      Universita` degli Studi di Trento                                   |
 |      email: enrico.bertolazzi@unitn.it                                   |
 |                                                                          |
\*--------------------------------------------------------------------------*/

#include "Clothoids.hh"

#include <cfloat>
#include <limits>

#ifdef __GNUC__
#pragma GCC diagnostic push
#pragma GCC diagnostic ignored "-Wsign-conversion"
#endif
#ifdef __clang__
#pragma clang diagnostic push
#pragma clang diagnostic ignored "-Wsign-conversion"
#endif

namespace G2lib {

  using std::numeric_limits;
  using std::lower_bound;
  using std::vector;
  using std::swap;
  using std::abs;

  /*\
   |   ____ _       _   _           _     _ _     _     _
   |  / ___| | ___ | |_| |__   ___ (_) __| | |   (_)___| |_
   | | |   | |/ _ \| __| '_ \ / _ \| |/ _` | |   | / __| __|
   | | |___| | (_) | |_| | | | (_) | | (_| | |___| \__ \ |_
   |  \____|_|\___/ \__|_| |_|\___/|_|\__,_|_____|_|___/\__|
   |
  \*/

  BiarcList::BiarcList( LineSegment const & LS )
  : BaseCurve(G2LIB_BIARC_LIST)
  , m_aabb_done(false)
  {
    this->resetLastInterval();
    this->init();
    this->push_back( LS );
  }

  BiarcList::BiarcList( CircleArc const & C )
  : BaseCurve(G2LIB_BIARC_LIST)
  , m_aabb_done(false)
  {
    this->resetLastInterval();
    this->init();
    this->push_back( C );
  }

  BiarcList::BiarcList( Biarc const & C )
  : BaseCurve(G2LIB_BIARC_LIST)
  , m_aabb_done(false)
  {
    this->resetLastInterval();
    this->init();
    this->push_back( C );
  }

  BiarcList::BiarcList( PolyLine const & pl )
  : BaseCurve(G2LIB_BIARC_LIST)
  , m_aabb_done(false)
  {
    this->resetLastInterval();
    this->init();
    this->push_back( pl );
  }

  BiarcList::BiarcList( BaseCurve const & C )
  : BaseCurve(G2LIB_BIARC_LIST)
  , m_aabb_done(false)
  {
    this->resetLastInterval();
    this->init();
    switch ( C.type() ) {
    case G2LIB_LINE:
      push_back( *static_cast<LineSegment const *>(&C) );
      break;
    case G2LIB_CIRCLE:
      push_back( *static_cast<CircleArc const *>(&C) );
      break;
    case G2LIB_BIARC:
      push_back( *static_cast<Biarc const *>(&C) );
      break;
    case G2LIB_BIARC_LIST:
      copy( *static_cast<BiarcList const *>(&C) );
      break;
    case G2LIB_POLYLINE:
      push_back( *static_cast<PolyLine const *>(&C) );
      break;
    case G2LIB_CLOTHOID:
    case G2LIB_CLOTHOID_LIST:
      UTILS_ERROR(
        "BiarcList constructor cannot convert from: {}\n",
        CurveType_name[C.type()]
      );
    }
  }

  // - - - - - - - - - - - - - - - - - - - - - - - - - - - - - - - - - - - - - -

  void
  BiarcList::init() {
    m_s0.clear();
    m_biarcList.clear();
    this->resetLastInterval();
    m_aabb_done = false;
  }

  // - - - - - - - - - - - - - - - - - - - - - - - - - - - - - - - - - - - - - -

  void
  BiarcList::copy( BiarcList const & L ) {
    this->init();
    m_biarcList.reserve(L.m_biarcList.size());
    std::copy( L.m_biarcList.begin(),
               L.m_biarcList.end(),
               back_inserter(m_biarcList) );
    m_s0.reserve(L.m_s0.size());
    std::copy( L.m_s0.begin(), L.m_s0.end(), back_inserter(m_s0) );
  }

  // - - - - - - - - - - - - - - - - - - - - - - - - - - - - - - - - - - - - - -

  int_type
  BiarcList::findAtS( real_type & s ) const {
    bool ok;
    int_type & lastInterval = *m_lastInterval.search( std::this_thread::get_id(), ok );
    Utils::searchInterval<int_type,real_type>(
      static_cast<int_type>(m_s0.size()),
      &m_s0.front(), s, lastInterval, false, true
    );
    return lastInterval;
  }

  // - - - - - - - - - - - - - - - - - - - - - - - - - - - - - - - - - - - - - -

  void
  BiarcList::reserve( int_type n ) {
    m_s0.reserve(size_t(n+1));
    m_biarcList.reserve(size_t(n));
  }

  // - - - - - - - - - - - - - - - - - - - - - - - - - - - - - - - - - - - - - -

  void
  BiarcList::push_back( LineSegment const & LS ) {
    if ( m_biarcList.empty() ) {
      m_s0.push_back(0);
      m_s0.push_back(LS.length());
    } else {
      m_s0.push_back(m_s0.back()+LS.length());
    }
    m_biarcList.push_back(Biarc(LS));
  }

  // - - - - - - - - - - - - - - - - - - - - - - - - - - - - - - - - - - - - - -

  void
  BiarcList::push_back( CircleArc const & C ) {
    if ( m_biarcList.empty() ) {
      m_s0.push_back(0);
      m_s0.push_back(C.length());
    } else {
      m_s0.push_back(m_s0.back()+C.length());
    }
    m_biarcList.push_back(Biarc(C));
  }

  // - - - - - - - - - - - - - - - - - - - - - - - - - - - - - - - - - - - - - -

  void
  BiarcList::push_back( Biarc const & c ) {
    if ( m_biarcList.empty() ) {
      m_s0.push_back(0);
      m_s0.push_back(c.length());
    } else {
      m_s0.push_back(m_s0.back()+c.length());
    }
    m_biarcList.push_back(c);
  }

  // - - - - - - - - - - - - - - - - - - - - - - - - - - - - - - - - - - - - - -

  void
  BiarcList::push_back( PolyLine const & c ) {
    m_s0.reserve( m_s0.size() + c.m_polylineList.size() + 1 );
    m_biarcList.reserve( m_biarcList.size() + c.m_polylineList.size() );

    if ( m_s0.empty() ) m_s0.push_back(0);

    vector<LineSegment>::const_iterator ip = c.m_polylineList.begin();
    for (; ip != c.m_polylineList.end(); ++ip ) {
      m_s0.push_back(m_s0.back()+ip->length());
      m_biarcList.push_back(Biarc(*ip));
    }
  }

  // - - - - - - - - - - - - - - - - - - - - - - - - - - - - - - - - - - - - - -

  void
  BiarcList::push_back_G1(
    real_type x1,
    real_type y1,
    real_type theta1
  ) {
    UTILS_ASSERT0(
      !m_biarcList.empty(),
      "BiarcList::push_back_G1(...) empty list!\n"
    );
    Biarc c;
    real_type x0     = m_biarcList.back().xEnd();
    real_type y0     = m_biarcList.back().yEnd();
    real_type theta0 = m_biarcList.back().thetaEnd();
    c.build( x0, y0, theta0, x1, y1, theta1 );
    push_back( c );
  }

  // - - - - - - - - - - - - - - - - - - - - - - - - - - - - - - - - - - - - - -

  void
  BiarcList::push_back_G1(
    real_type x0,
    real_type y0,
    real_type theta0,
    real_type x1,
    real_type y1,
    real_type theta1
  ) {
    Biarc c;
    c.build( x0, y0, theta0, x1, y1, theta1 );
    push_back( c );
  }

  // - - - - - - - - - - - - - - - - - - - - - - - - - - - - - - - - - - - - - -

  bool
  BiarcList::build_G1(
    int_type          n,
    real_type const * x,
    real_type const * y,
    real_type const * theta
  ) {
    UTILS_ASSERT0(
      n > 1,
      "BiarcList::build_G1, at least 2 points are necessary\n"
    );
    init();
    reserve( n-1 );
    Biarc c;
    for ( int_type k = 1; k < n; ++k ) {
      c.build( x[k-1], y[k-1], theta[k-1], x[k], y[k], theta[k] );
      this->push_back(c);
    }
    return true;
  }

  // - - - - - - - - - - - - - - - - - - - - - - - - - - - - - - - - - - - - - -

  bool
  BiarcList::build_G1(
    int_type          n,
    real_type const * x,
    real_type const * y
  ) {
    size_t nn = size_t(n);
    Utils::Malloc<real_type> mem( "BiarcList::build_G1" );
    mem.allocate( 5 * nn );
    real_type * theta     = mem( nn );
    real_type * theta_min = mem( nn );
    real_type * theta_max = mem( nn );
    real_type * omega     = mem( nn );
    real_type * len       = mem( nn );
    G2lib::xy_to_guess_angle(
      n, x, y, theta, theta_min, theta_max, omega, len
    );
    return this->build_G1( n, x, y, theta );
  }

  // - - - - - - - - - - - - - - - - - - - - - - - - - - - - - - - - - - - - - -

  Biarc const &
  BiarcList::get( int_type idx ) const {
    UTILS_ASSERT(
      !m_biarcList.empty(),
      "BiarcList::get( {} ) empty list\n", idx
    );
    UTILS_ASSERT(
      idx >= 0 && idx < int_type(m_biarcList.size()),
      "BiarcList::get( {} ) bad index, must be in [0,{}]\n",
      idx, m_biarcList.size()-1
    );
    return m_biarcList[idx];
  }

  // - - - - - - - - - - - - - - - - - - - - - - - - - - - - - - - - - - - - - -

  Biarc const &
  BiarcList::getAtS( real_type s ) const
  { return this->get(findAtS(s)); }

  /*\
   |   _                  _   _
   |  | | ___ _ __   __ _| |_| |__
   |  | |/ _ \ '_ \ / _` | __| '_ \
   |  | |  __/ | | | (_| | |_| | | |
   |  |_|\___|_| |_|\__, |\__|_| |_|
   |                |___/
  \*/

  real_type
  BiarcList::length() const {
    return m_s0.back() - m_s0.front();
  }

  real_type
  BiarcList::length_ISO( real_type offs ) const {
    real_type L = 0;
    vector<Biarc>::const_iterator is = m_biarcList.begin();
    for (; is != m_biarcList.end(); ++is ) L += is->length_ISO( offs );
    return L;
  }

  real_type
  BiarcList::segment_length( int_type nseg ) const {
    Biarc const & c = this->get( nseg );
    return c.length();
  }

  real_type
  BiarcList::segment_length_ISO( int_type nseg, real_type offs ) const {
    Biarc const & c = this->get( nseg );
    return c.length_ISO( offs );
  }

  /*\
   |   _    _   _____    _                _
   |  | |__| |_|_   _| _(_)__ _ _ _  __ _| |___
   |  | '_ \ '_ \| || '_| / _` | ' \/ _` | / -_)
   |  |_.__/_.__/|_||_| |_\__,_|_||_\__, |_\___|
   |                                |___/
  \*/

  void
  BiarcList::bbTriangles(
    vector<Triangle2D> & tvec,
    real_type            max_angle,
    real_type            max_size,
    int_type             icurve
  ) const {
    vector<Biarc>::const_iterator ic = m_biarcList.begin();
    for ( int_type ipos = icurve; ic != m_biarcList.end(); ++ic, ++ipos )
      ic->bbTriangles( tvec, max_angle, max_size, ipos );
  }

  void
  BiarcList::bbTriangles_ISO(
    real_type            offs,
    vector<Triangle2D> & tvec,
    real_type            max_angle,
    real_type            max_size,
    int_type             icurve
  ) const {
    vector<Biarc>::const_iterator ic = m_biarcList.begin();
    for ( int_type ipos = icurve; ic != m_biarcList.end(); ++ic, ++ipos )
      ic->bbTriangles_ISO( offs, tvec, max_angle, max_size, ipos );
  }

  void
  BiarcList::bbTriangles_SAE(
    real_type            offs,
    vector<Triangle2D> & tvec,
    real_type            max_angle,
    real_type            max_size,
    int_type             icurve
  ) const {
    vector<Biarc>::const_iterator ic = m_biarcList.begin();
    for ( int_type ipos = icurve; ic != m_biarcList.end(); ++ic, ++ipos )
      ic->bbTriangles_SAE( offs, tvec, max_angle, max_size, ipos );
  }

  /*\
   |   _     _
   |  | |__ | |__   _____  __
   |  | '_ \| '_ \ / _ \ \/ /
   |  | |_) | |_) | (_) >  <
   |  |_.__/|_.__/ \___/_/\_\
  \*/

  void
  BiarcList::bbox_ISO(
    real_type   offs,
    real_type & xmin,
    real_type & ymin,
    real_type & xmax,
    real_type & ymax
  ) const {
    vector<Triangle2D> tvec;
    bbTriangles_ISO( offs, tvec, Utils::m_pi/18, 1e100 );
    xmin = ymin = numeric_limits<real_type>::infinity();
    xmax = ymax = -xmin;
    vector<Triangle2D>::const_iterator it;
    for ( it = tvec.begin(); it != tvec.end(); ++it ) {
      // - - - - - - - - - - - - - - - - - - - -
      if      ( it->x1() < xmin ) xmin = it->x1();
      else if ( it->x1() > xmax ) xmax = it->x1();
      if      ( it->x2() < xmin ) xmin = it->x2();
      else if ( it->x2() > xmax ) xmax = it->x2();
      if      ( it->x3() < xmin ) xmin = it->x3();
      else if ( it->x3() > xmax ) xmax = it->x3();
      // - - - - - - - - - - - - - - - - - - - -
      if      ( it->y1() < ymin ) ymin = it->y1();
      else if ( it->y1() > ymax ) ymax = it->y1();
      if      ( it->y2() < ymin ) ymin = it->y2();
      else if ( it->y2() > ymax ) ymax = it->y2();
      if      ( it->y3() < ymin ) ymin = it->y3();
      else if ( it->y3() > ymax ) ymax = it->y3();
    }
  }

  /*\
   |  _   _          _
   | | |_| |__   ___| |_ __ _
   | | __| '_ \ / _ \ __/ _` |
   | | |_| | | |  __/ || (_| |
   |  \__|_| |_|\___|\__\__,_|
  \*/

  real_type
  BiarcList::theta( real_type s ) const {
    int_type idx = this->findAtS( s );
    Biarc const & c = this->get( idx );
    return c.theta( s - m_s0[idx] );
  }

  // - - - - - - - - - - - - - - - - - - - - - - - - - - - - - - - - - - - - - -

  real_type
  BiarcList::theta_D( real_type s ) const {
    int_type idx = this->findAtS( s );
    Biarc const & c = this->get( idx );
    return c.theta_D( s - m_s0[idx] );
  }

  // - - - - - - - - - - - - - - - - - - - - - - - - - - - - - - - - - - - - - -

  real_type
  BiarcList::theta_DD( real_type s ) const  {
    int_type idx = this->findAtS( s );
    Biarc const & c = this->get( idx );
    return c.theta_DD( s - m_s0[idx] );
  }

  // - - - - - - - - - - - - - - - - - - - - - - - - - - - - - - - - - - - - - -

  real_type
  BiarcList::theta_DDD( real_type s ) const {
    int_type idx = this->findAtS( s );
    Biarc const & c = this->get( idx );
    return c.theta_DDD( s - m_s0[idx] );
  }

  /*\
   |  _____                   _   _   _
   | |_   _|   __ _ _ __   __| | | \ | |
   |   | |    / _` | '_ \ / _` | |  \| |
   |   | |   | (_| | | | | (_| | | |\  |
   |   |_|    \__,_|_| |_|\__,_| |_| \_|
  \*/

  real_type
  BiarcList::tx( real_type s ) const {
    int_type idx = this->findAtS( s );
    Biarc const & c = this->get( idx );
    return c.tx( s - m_s0[idx] );
  }

  // - - - - - - - - - - - - - - - - - - - - - - - - - - - - - - - - - - - - - -

  real_type
  BiarcList::ty( real_type s ) const {
    int_type idx = this->findAtS( s );
    Biarc const & c = this->get( idx );
    return c.ty( s - m_s0[idx] );
  }

  // - - - - - - - - - - - - - - - - - - - - - - - - - - - - - - - - - - - - - -

  real_type
  BiarcList::tx_D( real_type s ) const {
    int_type idx = this->findAtS( s );
    Biarc const & c = this->get( idx );
    return c.tx_D( s - m_s0[idx] );
  }

  // - - - - - - - - - - - - - - - - - - - - - - - - - - - - - - - - - - - - - -

  real_type
  BiarcList::ty_D( real_type s ) const {
    int_type idx = this->findAtS( s );
    Biarc const & c = this->get( idx );
    return c.ty_D( s - m_s0[idx] );
  }

  // - - - - - - - - - - - - - - - - - - - - - - - - - - - - - - - - - - - - - -

  real_type
  BiarcList::tx_DD( real_type s ) const {
    int_type idx = this->findAtS( s );
    Biarc const & c = this->get( idx );
    return c.tx_DD( s - m_s0[idx] );
  }

  // - - - - - - - - - - - - - - - - - - - - - - - - - - - - - - - - - - - - - -

  real_type
  BiarcList::ty_DD( real_type s ) const {
    int_type idx = this->findAtS( s );
    Biarc const & c = this->get( idx );
    return c.ty_DD( s - m_s0[idx] );
  }

  // - - - - - - - - - - - - - - - - - - - - - - - - - - - - - - - - - - - - - -

  real_type
  BiarcList::tx_DDD( real_type s ) const {
    int_type idx = this->findAtS( s );
    Biarc const & c = this->get( idx );
    return c.tx_DDD( s - m_s0[idx] );
  }

  // - - - - - - - - - - - - - - - - - - - - - - - - - - - - - - - - - - - - - -

  real_type
  BiarcList::ty_DDD( real_type s ) const {
    int_type idx = this->findAtS( s );
    Biarc const & c = this->get( idx );
    return c.ty_DDD( s - m_s0[idx] );
  }

  // - - - - - - - - - - - - - - - - - - - - - - - - - - - - - - - - - - - - - -

  void
  BiarcList::tg(
    real_type   s,
    real_type & tg_x,
    real_type & tg_y
  ) const {
    int_type idx = this->findAtS( s );
    Biarc const & c = this->get( idx );
    return c.tg( s - m_s0[idx], tg_x, tg_y );
  }

  // - - - - - - - - - - - - - - - - - - - - - - - - - - - - - - - - - - - - - -

  void
  BiarcList::tg_D(
    real_type   s,
    real_type & tg_x_D,
    real_type & tg_y_D
  ) const {
    int_type idx = this->findAtS( s );
    Biarc const & c = this->get( idx );
    return c.tg_D( s - m_s0[idx], tg_x_D, tg_y_D );
  }

  // - - - - - - - - - - - - - - - - - - - - - - - - - - - - - - - - - - - - - -

  void
  BiarcList::tg_DD(
    real_type   s,
    real_type & tg_x_DD,
    real_type & tg_y_DD
  ) const {
    int_type idx = this->findAtS( s );
    Biarc const & c = this->get( idx );
    return c.tg_DD( s - m_s0[idx], tg_x_DD, tg_y_DD );
  }

  // - - - - - - - - - - - - - - - - - - - - - - - - - - - - - - - - - - - - - -

  void
  BiarcList::tg_DDD(
    real_type   s,
    real_type & tg_x_DDD,
    real_type & tg_y_DDD
  ) const {
    int_type idx = this->findAtS( s );
    Biarc const & c = this->get( idx );
    return c.tg_DDD( s - m_s0[idx], tg_x_DDD, tg_y_DDD );
  }

  // - - - - - - - - - - - - - - - - - - - - - - - - - - - - - - - - - - - - - -

  void
  BiarcList::evaluate(
    real_type   s,
    real_type & th,
    real_type & k,
    real_type & x,
    real_type & y
  ) const {
    int_type idx = this->findAtS( s );
    Biarc const & c = this->get( idx );
    c.evaluate( s - m_s0[idx], th, k, x, y );
  }

  // - - - - - - - - - - - - - - - - - - - - - - - - - - - - - - - - - - - - - -

  void
  BiarcList::evaluate_ISO(
    real_type   s,
    real_type   offs,
    real_type & th,
    real_type & k,
    real_type & x,
    real_type & y
  ) const {
    int_type idx = this->findAtS( s );
    Biarc const & c = this->get( idx );
    c.evaluate_ISO( s - m_s0[idx], offs, th, k, x, y );
  }

  // - - - - - - - - - - - - - - - - - - - - - - - - - - - - - - - - - - - - - -

  real_type
  BiarcList::X( real_type s ) const {
    int_type idx = this->findAtS( s );
    Biarc const & c = this->get( idx );
    return c.X( s - m_s0[idx] );
  }

  // - - - - - - - - - - - - - - - - - - - - - - - - - - - - - - - - - - - - - -

  real_type
  BiarcList::Y( real_type s ) const  {
    int_type idx = this->findAtS( s );
    Biarc const & c = this->get( idx );
    return c.Y( s - m_s0[idx] );
  }

  // - - - - - - - - - - - - - - - - - - - - - - - - - - - - - - - - - - - - - -

  real_type
  BiarcList::X_D( real_type s ) const {
    int_type idx = this->findAtS( s );
    Biarc const & c = this->get( idx );
    return c.X_D( s - m_s0[idx] );
  }

  // - - - - - - - - - - - - - - - - - - - - - - - - - - - - - - - - - - - - - -

  real_type
  BiarcList::Y_D( real_type s ) const {
    int_type idx = this->findAtS( s );
    Biarc const & c = this->get( idx );
    return c.Y_D( s - m_s0[idx] );
  }

  // - - - - - - - - - - - - - - - - - - - - - - - - - - - - - - - - - - - - - -

  real_type
  BiarcList::X_DD( real_type s ) const {
    int_type idx = this->findAtS( s );
    Biarc const & c = this->get( idx );
    return c.X_DD( s - m_s0[idx] );
  }

  // - - - - - - - - - - - - - - - - - - - - - - - - - - - - - - - - - - - - - -

  real_type
  BiarcList::Y_DD( real_type s ) const {
    int_type idx = this->findAtS( s );
    Biarc const & c = this->get( idx );
    return c.Y_DD( s - m_s0[idx] );
  }

  // - - - - - - - - - - - - - - - - - - - - - - - - - - - - - - - - - - - - - -

  real_type
  BiarcList::X_DDD( real_type s ) const {
    int_type idx = this->findAtS( s );
    Biarc const & c = this->get( idx );
    return c.X_DDD( s - m_s0[idx] );
  }

  // - - - - - - - - - - - - - - - - - - - - - - - - - - - - - - - - - - - - - -

  real_type
  BiarcList::Y_DDD( real_type s ) const {
    int_type idx = this->findAtS( s );
    Biarc const & c = this->get( idx );
    return c.Y_DDD( s - m_s0[idx] );
  }

  // - - - - - - - - - - - - - - - - - - - - - - - - - - - - - - - - - - - - - -

  void
  BiarcList::eval(
    real_type   s,
    real_type & x,
    real_type & y
  ) const {
    int_type idx = this->findAtS( s );
    Biarc const & c = this->get( idx );
    return c.eval( s - m_s0[idx], x, y );
  }

  // - - - - - - - - - - - - - - - - - - - - - - - - - - - - - - - - - - - - - -

  void
  BiarcList::eval_D(
    real_type   s,
    real_type & x_D,
    real_type & y_D
  ) const {
    int_type idx = this->findAtS( s );
    Biarc const & c = this->get( idx );
    return c.eval_D( s - m_s0[idx], x_D, y_D );
  }

  // - - - - - - - - - - - - - - - - - - - - - - - - - - - - - - - - - - - - - -

  void
  BiarcList::eval_DD(
    real_type   s,
    real_type & x_DD,
    real_type & y_DD
  ) const {
    int_type idx = this->findAtS( s );
    Biarc const & c = this->get( idx );
    return c.eval_DD( s - m_s0[idx], x_DD, y_DD );
  }

  // - - - - - - - - - - - - - - - - - - - - - - - - - - - - - - - - - - - - - -

  void
  BiarcList::eval_DDD(
    real_type   s,
    real_type & x_DDD,
    real_type & y_DDD
  ) const {
    int_type idx = this->findAtS( s );
    Biarc const & c = this->get( idx );
    return c.eval_DDD( s - m_s0[idx], x_DDD, y_DDD );
  }

  /*\
   |         __  __          _
   |   ___  / _|/ _|___  ___| |_
   |  / _ \| |_| |_/ __|/ _ \ __|
   | | (_) |  _|  _\__ \  __/ |_
   |  \___/|_| |_| |___/\___|\__|
  \*/

  real_type
  BiarcList::X_ISO( real_type s, real_type offs ) const {
    int_type idx = this->findAtS( s );
    Biarc const & c = this->get( idx );
    return c.X_ISO( s - m_s0[idx], offs );
  }

  // - - - - - - - - - - - - - - - - - - - - - - - - - - - - - - - - - - - - - -

  real_type
  BiarcList::Y_ISO( real_type s, real_type offs ) const {
    int_type idx = this->findAtS( s );
    Biarc const & c = this->get( idx );
    return c.Y_ISO( s - m_s0[idx], offs );
  }

  // - - - - - - - - - - - - - - - - - - - - - - - - - - - - - - - - - - - - - -

  real_type
  BiarcList::X_ISO_D( real_type s, real_type offs ) const {
    int_type idx = this->findAtS( s );
    Biarc const & c = this->get( idx );
    return c.X_ISO_D( s - m_s0[idx], offs );
  }

  // - - - - - - - - - - - - - - - - - - - - - - - - - - - - - - - - - - - - - -

  real_type
  BiarcList::Y_ISO_D( real_type s, real_type offs ) const {
    int_type idx = this->findAtS( s );
    Biarc const & c = this->get( idx );
    return c.Y_ISO_D( s - m_s0[idx], offs );
  }

  // - - - - - - - - - - - - - - - - - - - - - - - - - - - - - - - - - - - - - -

  real_type
  BiarcList::X_ISO_DD( real_type s, real_type offs ) const {
    int_type idx = this->findAtS( s );
    Biarc const & c = this->get( idx );
    return c.X_ISO_DD( s - m_s0[idx], offs );
  }

  // - - - - - - - - - - - - - - - - - - - - - - - - - - - - - - - - - - - - - -

  real_type
  BiarcList::Y_ISO_DD( real_type s, real_type offs ) const {
    int_type idx = this->findAtS( s );
    Biarc const & c = this->get( idx );
    return c.Y_ISO_DD( s - m_s0[idx], offs );
  }

  // - - - - - - - - - - - - - - - - - - - - - - - - - - - - - - - - - - - - - -

  real_type
  BiarcList::X_ISO_DDD( real_type s, real_type offs ) const {
    int_type idx = this->findAtS( s );
    Biarc const & c = this->get( idx );
    return c.X_ISO_DDD( s - m_s0[idx], offs );
  }

  // - - - - - - - - - - - - - - - - - - - - - - - - - - - - - - - - - - - - - -

  real_type
  BiarcList::Y_ISO_DDD( real_type s, real_type offs ) const {
    int_type idx = this->findAtS( s );
    Biarc const & c = this->get( idx );
    return c.Y_ISO_DDD( s - m_s0[idx], offs );
  }

  // - - - - - - - - - - - - - - - - - - - - - - - - - - - - - - - - - - - - - -

  void
  BiarcList::eval_ISO(
    real_type   s,
    real_type   offs,
    real_type & x,
    real_type & y
  ) const {
    int_type idx = this->findAtS( s );
    Biarc const & c = this->get( idx );
    return c.eval_ISO( s - m_s0[idx], offs, x, y );
  }

  // - - - - - - - - - - - - - - - - - - - - - - - - - - - - - - - - - - - - - -

  void
  BiarcList::eval_ISO_D(
    real_type   s,
    real_type   offs,
    real_type & x_D,
    real_type & y_D
  ) const {
    int_type idx = this->findAtS( s );
    Biarc const & c = this->get( idx );
    return c.eval_ISO_D( s - m_s0[idx], offs, x_D, y_D );
  }

  // - - - - - - - - - - - - - - - - - - - - - - - - - - - - - - - - - - - - - -

  void
  BiarcList::eval_ISO_DD(
    real_type   s,
    real_type   offs,
    real_type & x_DD,
    real_type & y_DD
  ) const {
    int_type idx = this->findAtS( s );
    Biarc const & c = this->get( idx );
    return c.eval_ISO_DD( s - m_s0[idx], offs, x_DD, y_DD );
  }

  // - - - - - - - - - - - - - - - - - - - - - - - - - - - - - - - - - - - - - -

  void
  BiarcList::eval_ISO_DDD(
    real_type   s,
    real_type   offs,
    real_type & x_DDD,
    real_type & y_DDD
  ) const {
    int_type idx = this->findAtS( s );
    Biarc const & c = this->get( idx );
    return c.eval_ISO_DDD( s - m_s0[idx], offs, x_DDD, y_DDD );
  }

  /*\
   |  _                        __
   | | |_ _ __ __ _ _ __  ___ / _| ___  _ __ _ __ ___
   | | __| '__/ _` | '_ \/ __| |_ / _ \| '__| '_ ` _ \
   | | |_| | | (_| | | | \__ \  _| (_) | |  | | | | | |
   |  \__|_|  \__,_|_| |_|___/_|  \___/|_|  |_| |_| |_|
  \*/

  void
  BiarcList::translate( real_type tx, real_type ty ) {
    vector<Biarc>::iterator ic = m_biarcList.begin();
    for (; ic != m_biarcList.end(); ++ic ) ic->translate( tx, ty );
  }

  // - - - - - - - - - - - - - - - - - - - - - - - - - - - - - - - - - - - - - -

  void
  BiarcList::rotate( real_type angle, real_type cx, real_type cy ) {
    vector<Biarc>::iterator ic = m_biarcList.begin();
    for (; ic != m_biarcList.end(); ++ic ) ic->rotate( angle, cx, cy );
  }

  // - - - - - - - - - - - - - - - - - - - - - - - - - - - - - - - - - - - - - -

  void
  BiarcList::scale( real_type sfactor ) {
    vector<Biarc>::iterator ic = m_biarcList.begin();
    real_type newx0 = ic->xBegin();
    real_type newy0 = ic->yBegin();
    m_s0[0] = 0;
    for ( size_t k=0; ic != m_biarcList.end(); ++ic, ++k ) {
      ic->scale( sfactor );
      ic->changeOrigin( newx0, newy0 );
      newx0     = ic->xEnd();
      newy0     = ic->yEnd();
      m_s0[k+1] = m_s0[k] + ic->length();
    }
  }

  // - - - - - - - - - - - - - - - - - - - - - - - - - - - - - - - - - - - - - -

  void
  BiarcList::reverse() {
    std::reverse( m_biarcList.begin(), m_biarcList.end() );
    vector<Biarc>::iterator ic = m_biarcList.begin();
    ic->reverse();
    real_type newx0 = ic->xEnd();
    real_type newy0 = ic->yEnd();
    m_s0[0] = 0;
    m_s0[1] = ic->length();
    size_t k = 1;
    for ( ++ic; ic != m_biarcList.end(); ++ic, ++k ) {
      ic->reverse();
      ic->changeOrigin( newx0, newy0 );
      newx0     = ic->xEnd();
      newy0     = ic->yEnd();
      m_s0[k+1] = m_s0[k] + ic->length();
    }
  }

  // - - - - - - - - - - - - - - - - - - - - - - - - - - - - - - - - - - - - - -

  void
  BiarcList::changeOrigin( real_type newx0, real_type newy0 ) {
    vector<Biarc>::iterator ic = m_biarcList.begin();
    for (; ic != m_biarcList.end(); ++ic ) {
      ic->changeOrigin( newx0, newy0 );
      newx0 = ic->xEnd();
      newy0 = ic->yEnd();
    }
  }

  // - - - - - - - - - - - - - - - - - - - - - - - - - - - - - - - - - - - - - -

  void
  BiarcList::trim( real_type s_begin, real_type s_end ) {
    UTILS_ASSERT(
      s_begin >= m_s0.front() && s_end <= m_s0.back() && s_end > s_begin,
      "BiarcList::trim( s_begin={}, s_end={} ) bad range, must be in [ {}, {} ]\n",
      s_begin, s_end, m_s0.front(), m_s0.back()
    );

    size_t i_begin = size_t( findAtS( s_begin ) );
    size_t i_end   = size_t( findAtS( s_end ) );
    if ( i_begin == i_end ) {
      m_biarcList[i_begin].trim( s_begin-m_s0[i_begin], s_end-m_s0[i_begin] );
    } else {
      m_biarcList[i_begin].trim( s_begin-m_s0[i_begin], m_s0[i_begin+1]-m_s0[i_begin] );
      m_biarcList[i_end].trim( 0, s_end-m_s0[i_end] );
    }
    m_biarcList.erase( m_biarcList.begin()+i_end+1, m_biarcList.end() );
    m_biarcList.erase( m_biarcList.begin(), m_biarcList.begin()+i_begin );
    if ( m_biarcList.back().length() <= machepsi100 ) m_biarcList.pop_back();
    vector<Biarc>::iterator ic = m_biarcList.begin();
    m_s0.resize( m_biarcList.size() + 1 );
    m_s0[0] = 0;
    size_t k = 0;
    for ( ++ic; ic != m_biarcList.end(); ++ic, ++k )
      m_s0[k+1] = m_s0[k] + ic->length();
    this->resetLastInterval();
  }

  /*\
   |     _        _    ____  ____  _
   |    / \      / \  | __ )| __ )| |_ _ __ ___  ___
   |   / _ \    / _ \ |  _ \|  _ \| __| '__/ _ \/ _ \
   |  / ___ \  / ___ \| |_) | |_) | |_| | |  __/  __/
   | /_/   \_\/_/   \_\____/|____/ \__|_|  \___|\___|
  \*/

  #ifndef DOXYGEN_SHOULD_SKIP_THIS
  void
  BiarcList::build_AABBtree_ISO(
    real_type offs,
    real_type max_angle,
    real_type max_size
  ) const {

    if ( m_aabb_done &&
         Utils::isZero( offs-m_aabb_offs ) &&
         Utils::isZero( max_angle-m_aabb_max_angle ) &&
         Utils::isZero( max_size-m_aabb_max_size ) ) return;

    #ifdef G2LIB_USE_CXX11
    vector<shared_ptr<BBox const> > bboxes;
    #else
    vector<BBox const *> bboxes;
    #endif

    bbTriangles_ISO( offs, m_aabb_tri, max_angle, max_size );
    bboxes.reserve(m_aabb_tri.size());
    vector<Triangle2D>::const_iterator it;
    int_type ipos = 0;
    for ( it = m_aabb_tri.begin(); it != m_aabb_tri.end(); ++it, ++ipos ) {
      real_type xmin, ymin, xmax, ymax;
      it->bbox( xmin, ymin, xmax, ymax );
      #ifdef G2LIB_USE_CXX11
      bboxes.push_back( make_shared<BBox const>(
        xmin, ymin, xmax, ymax, G2LIB_CLOTHOID, ipos
      ) );
      #else
      bboxes.push_back(
        new BBox( xmin, ymin, xmax, ymax, G2LIB_CLOTHOID, ipos )
      );
      #endif
    }
    m_aabb_tree.build(bboxes);
    m_aabb_done      = true;
    m_aabb_offs      = offs;
    m_aabb_max_angle = max_angle;
    m_aabb_max_size  = max_size;
  }
  #endif

  /*\
   |   _       _                          _
   |  (_)_ __ | |_ ___ _ __ ___  ___  ___| |_
   |  | | '_ \| __/ _ \ '__/ __|/ _ \/ __| __|
   |  | | | | | ||  __/ |  \__ \  __/ (__| |_
   |  |_|_| |_|\__\___|_|  |___/\___|\___|\__|
  \*/

  // - - - - - - - - - - - - - - - - - - - - - - - - - - - - - - - - - - - - - -

  bool
  BiarcList::collision( BiarcList const & C ) const {
    this->build_AABBtree_ISO( 0 );
    C.build_AABBtree_ISO( 0 );
    T2D_collision_list_ISO fun( this, 0, &C, 0 );
    return m_aabb_tree.collision( C.m_aabb_tree, fun, false );
  }

  // - - - - - - - - - - - - - - - - - - - - - - - - - - - - - - - - - - - - - -

  bool
  BiarcList::collision_ISO(
    real_type            offs,
    BiarcList const & C,
    real_type            offs_C
  ) const {
    this->build_AABBtree_ISO( offs );
    C.build_AABBtree_ISO( offs_C );
    T2D_collision_list_ISO fun( this, offs, &C, offs_C );
    return m_aabb_tree.collision( C.m_aabb_tree, fun, false );
  }

  /*\
   |   _       _                          _
   |  (_)_ __ | |_ ___ _ __ ___  ___  ___| |_
   |  | | '_ \| __/ _ \ '__/ __|/ _ \/ __| __|
   |  | | | | | ||  __/ |  \__ \  __/ (__| |_
   |  |_|_| |_|\__\___|_|  |___/\___|\___|\__|
   |
  \*/

  void
  BiarcList::intersect_ISO(
    real_type         offs,
    BiarcList const & CL,
    real_type         offs_CL,
    IntersectList   & ilist,
    bool              swap_s_vals
  ) const {
    if ( intersect_with_AABBtree ) {
      this->build_AABBtree_ISO( offs );
      CL.build_AABBtree_ISO( offs_CL );
      AABBtree::VecPairPtrBBox iList;
      m_aabb_tree.intersect( CL.m_aabb_tree, iList );

      AABBtree::VecPairPtrBBox::const_iterator ip;
      for ( ip = iList.begin(); ip != iList.end(); ++ip ) {
        size_t ipos1 = size_t(ip->first->Ipos());
        size_t ipos2 = size_t(ip->second->Ipos());

        Triangle2D const & T1 = m_aabb_tri[ipos1];
        Triangle2D const & T2 = CL.m_aabb_tri[ipos2];

        Biarc const & C1 = m_biarcList[T1.Icurve()];
        Biarc const & C2 = CL.m_biarcList[T2.Icurve()];

        IntersectList ilist1;
        C1.intersect_ISO( offs, C2, offs_CL, ilist1, false );

        for ( IntersectList::const_iterator it = ilist1.begin();
              it != ilist1.end(); ++it ) {
          real_type ss1 = it->first  + m_s0[T1.Icurve()];
          real_type ss2 = it->second + CL.m_s0[T2.Icurve()];
          if ( swap_s_vals ) swap( ss1, ss2 );
          ilist.push_back( Ipair( ss1, ss2 ) );
        }
      }
    } else {
      bbTriangles_ISO( offs, m_aabb_tri, Utils::m_pi/18, 1e100 );
      CL.bbTriangles_ISO( offs_CL, CL.m_aabb_tri, Utils::m_pi/18, 1e100 );
      vector<Triangle2D>::const_iterator i1, i2;
      for ( i1 = m_aabb_tri.begin(); i1 != m_aabb_tri.end(); ++i1 ) {
        for ( i2 = CL.m_aabb_tri.begin(); i2 != CL.m_aabb_tri.end(); ++i2 ) {
          Triangle2D const & T1 = *i1;
          Triangle2D const & T2 = *i2;

          Biarc const & C1 = m_biarcList[T1.Icurve()];
          Biarc const & C2 = CL.m_biarcList[T2.Icurve()];

          IntersectList ilist1;
          C1.intersect_ISO( offs, C2, offs_CL, ilist1, false );

          for ( IntersectList::const_iterator it = ilist1.begin();
                it != ilist1.end(); ++it ) {
            real_type ss1 = it->first  + m_s0[T1.Icurve()];
            real_type ss2 = it->second + CL.m_s0[T2.Icurve()];
            if ( swap_s_vals ) swap( ss1, ss2 );
            ilist.push_back( Ipair( ss1, ss2 ) );
          }
        }
      }

    }
  }

  /*\
   |      _ _     _
   |   __| (_)___| |_ __ _ _ __   ___ ___
   |  / _` | / __| __/ _` | '_ \ / __/ _ \
   | | (_| | \__ \ || (_| | | | | (_|  __/
   |  \__,_|_|___/\__\__,_|_| |_|\___\___|
  \*/

  int_type
  BiarcList::closestPoint_internal(
    real_type   qx,
    real_type   qy,
    real_type   offs,
    real_type & x,
    real_type & y,
    real_type & s,
    real_type & DST
  ) const {

    this->build_AABBtree_ISO( offs );

    AABBtree::VecPtrBBox candidateList;
    m_aabb_tree.min_distance( qx, qy, candidateList );
    AABBtree::VecPtrBBox::const_iterator ic;
    UTILS_ASSERT0(
      candidateList.size() > 0,
      "BiarcList::closestPoint no candidate\n"
    );
    int_type icurve = 0;
    DST = numeric_limits<real_type>::infinity();
    for ( ic = candidateList.begin(); ic != candidateList.end(); ++ic ) {
      size_t ipos = size_t((*ic)->Ipos());
      Triangle2D const & T = m_aabb_tri[ipos];
      real_type dst = T.distMin( qx, qy );
      if ( dst < DST ) {
        // refine distance
        real_type xx, yy, ss, tt;
        m_biarcList[T.Icurve()].closestPoint_ISO( qx, qy, offs, xx, yy, ss, tt, dst );
        if ( dst < DST ) {
          DST    = dst;
          s      = ss + m_s0[T.Icurve()];
          x      = xx;
          y      = yy;
          icurve = T.Icurve();
        }
      }
    }
    return icurve;
  }

  int_type
  BiarcList::closestPoint_ISO(
    real_type   qx,
    real_type   qy,
    real_type   offs,
    real_type & x,
    real_type & y,
    real_type & s,
    real_type & t,
    real_type & DST
  ) const {

    int_type icurve = this->closestPoint_internal( qx, qy, offs, x, y, s, DST );

    // check if projection is orthogonal
    real_type nx, ny;
    m_biarcList[icurve].nor_ISO( s - m_s0[icurve], nx, ny );
    real_type qxx = qx - x;
    real_type qyy = qy - y;
    t = qxx * nx + qyy * ny - offs; // signed distance
    real_type pt = abs(qxx * ny - qyy * nx);
    G2LIB_DEBUG_MESSAGE(
      "BiarcList::closestPoint_ISO\n"
      "||P-P0|| = {} and {}, |(P-P0).T| = {}\n",
      DST, hypot(qxx,qyy), pt
    );
    return pt > GLIB2_TOL_ANGLE*hypot(qxx,qyy) ? -(icurve+1) : icurve;
  }

  // - - - - - - - - - - - - - - - - - - - - - - - - - - - - - - - - - - - - - -

  int_type
  BiarcList::closestPoint_ISO(
    real_type   qx,
    real_type   qy,
    real_type & x,
    real_type & y,
    real_type & s,
    real_type & t,
    real_type & dst
  ) const {
    return closestPoint_ISO( qx, qy, 0, x, y, s, t, dst );
  }

  // - - - - - - - - - - - - - - - - - - - - - - - - - - - - - - - - - - - - - -
  // - - - - - - - - - - - - - - - - - - - - - - - - - - - - - - - - - - - - - -
  // - - - - - - - - - - - - - - - - - - - - - - - - - - - - - - - - - - - - - -
  // - - - - - - - - - - - - - - - - - - - - - - - - - - - - - - - - - - - - - -

  void
  BiarcList::getSTK(
    real_type * s,
    real_type * theta,
    real_type * kappa
  ) const {
    vector<Biarc>::const_iterator ic = m_biarcList.begin();
    int_type  k  = 0;
    real_type ss = 0;
    while ( ic != m_biarcList.end() ) {
      s[k]     = ss;
      theta[k] = ic->thetaBegin();
      kappa[k] = ic->kappaBegin();
      ss      += ic->length();
      ++k;
      ++ic;
    }
    --ic;
    s[k]     = ss;
    theta[k] = ic->thetaEnd();
    kappa[k] = ic->kappaEnd();
  }

  // - - - - - - - - - - - - - - - - - - - - - - - - - - - - - - - - - - - - - -

  void
  BiarcList::getXY( real_type * x, real_type * y ) const {
    vector<Biarc>::const_iterator ic = m_biarcList.begin();
    int_type k  = 0;
    while ( ic != m_biarcList.end() ) {
      x[k] = ic->xBegin();
      y[k] = ic->yBegin();
      ++k; ++ic;
    }
    --ic;
    x[k] = ic->xEnd();
    y[k] = ic->yEnd();
  }

  // - - - - - - - - - - - - - - - - - - - - - - - - - - - - - - - - - - - - - -

  int_type
  BiarcList::findST1(
    real_type   x,
    real_type   y,
    real_type & s,
    real_type & t
  ) const {

    UTILS_ASSERT0( !m_biarcList.empty(), "BiarcList::findST, empty list\n" );
    vector<Biarc>::const_iterator     ic = m_biarcList.begin();
    vector<real_type>::const_iterator is = m_s0.begin();

    s = t = 0;
    int_type  ipos = 0;
    int_type  iseg = 0;
    real_type S, T;
    bool ok = ic->findST_ISO( x, y, S, T );
    if ( ok ) {
      s    = *is + S;
      t    = T;
      iseg = 0;
    }

    for ( ++ic, ++is, ++ipos;
          ic != m_biarcList.end();
          ++ic, ++is, ++ipos ) {
      bool ok1 = ic->findST_ISO( x, y, S, T );
      if ( ok && ok1 ) ok1 = abs(T) < abs(t);
      if ( ok1 ) {
        ok   = true;
        s    = *is + S;
        t    = T;
        iseg = ipos;
      }
    }

    return ok ? iseg : -(1+iseg);
  }

  // - - - - - - - - - - - - - - - - - - - - - - - - - - - - - - - - - - - - - -

  int_type
  BiarcList::findST1(
    int_type    ibegin,
    int_type    iend,
    real_type   x,
    real_type   y,
    real_type & s,
    real_type & t
  ) const {

    UTILS_ASSERT0(
      !m_biarcList.empty(), "BiarcList::findST, empty list\n"
    );
    UTILS_ASSERT(
      ibegin >= 0 && ibegin <= iend &&
      iend < int_type(m_biarcList.size()),
      "BiarcList::findST( ibegin={}, iend={}, x, y, s, t )\n"
      "bad range not in [0,{}]\n",
      ibegin, iend, m_biarcList.size()-1
    );
    s = t = 0;
    int_type iseg = 0;
    bool ok = false;
    for ( int_type k = ibegin; k <= iend; ++k ) {
      Biarc const & ck = m_biarcList[k];
      real_type S, T;
      bool ok1 = ck.findST_ISO( x, y, S, T );
      if ( ok && ok1 ) ok1 = abs(T) < abs(t);
      if ( ok1 ) {
        ok   = true;
        s    = m_s0[k] + S;
        t    = T;
        iseg = k;
      }
    }
    return ok ? iseg : -(1+iseg);
  }

  // - - - - - - - - - - - - - - - - - - - - - - - - - - - - - - - - - - - - - -

  ostream_type &
  operator << ( ostream_type & stream, BiarcList const & CL ) {
    vector<Biarc>::const_iterator ic = CL.m_biarcList.begin();
    for (; ic != CL.m_biarcList.end(); ++ic )
      stream << *ic << '\n';
    return stream;
  }

  // - - - - - - - - - - - - - - - - - - - - - - - - - - - - - - - - - - - - - -
}

// EOF: BiarcList.cc
```

### Quick search

### Table of Contents

- Matlab Interface Manual
- C++ API
- MATLAB API

«
hide menu

menu
sidebar
»

### Navigation

- index
- toc
- Clothoids »
- Program Listing for File BiarcList.cc

© Copyright 2021, Enrico Bertolazzi and Marco Frego.
Created using Sphinx 4.2.0.
