# Supplementary material for: Smooth Interpolating Curves with Local Control and Monotone Alternating Curvature
Source: Comput Graph Forum. 2022 Oct 6;41(5):25–38. doi: 10.1111/cgf.14600 (PMC9827861; doi:10.1111/cgf.14600)
Supplement: Supplementary file 1 — Supplement Material [file CGF-41-25-s001.zip › Local-Smooth-Interpolating-MonoCurvature/extern/clothoids/docs/api-cpp/program_listing_file_Circle.cc.html]

Program Listing for File Circle.cc — Clothoids v2.0.9

### Navigation

- index
- toc
- Clothoids »
- Program Listing for File Circle.cc

# Program Listing for File Circle.cc¶

↰ Return to documentation for file (`Circle.cc`)

```
/*--------------------------------------------------------------------------*\
 |                                                                          |
 |  Copyright (C) 2017                                                      |
 |                                                                          |
 |         , __                 , __                                        |
 |        /|/  \               /|/  \                                       |
 |         | __/ _   ,_         | __/ _   ,_                                |
 |         |   \|/  /  |  |   | |   \|/  /  |  |   |                        |
 |         |(__/|__/   |_/ \_/|/|(__/|__/   |_/ \_/|/                       |
 |                           /|                   /|                        |
 |                           \|                   \|                        |
 |                                                                          |
 |      Enrico Bertolazzi                                                   |
 |      Dipartimento di Ingegneria Industriale                              |
 |      Universita` degli Studi di Trento                                   |
 |      email: enrico.bertolazzi@unitn.it                                   |
 |                                                                          |
\*--------------------------------------------------------------------------*/

#include "Clothoids.hh"

// Workaround for Visual Studio
#ifdef min
  #undef min
#endif

#ifdef max
  #undef max
#endif

#include <cmath>
#include <algorithm>

namespace G2lib {

  using std::min;
  using std::max;
  using std::abs;
  using std::tan;
  using std::abs;
  using std::ceil;
  using std::floor;
  using std::swap;
  using std::vector;

  /*\
   |    ____ _          _         _
   |   / ___(_)_ __ ___| | ___   / \   _ __ ___
   |  | |   | | '__/ __| |/ _ \ / _ \ | '__/ __|
   |  | |___| | | | (__| |  __// ___ \| | | (__
   |   \____|_|_|  \___|_|\___/_/   \_\_|  \___|
  \*/

  CircleArc::CircleArc( BaseCurve const & C )
  : BaseCurve(G2LIB_CIRCLE)
  {
    switch ( C.type() ) {
    case G2LIB_LINE:
      {
        LineSegment const & LS = *static_cast<LineSegment const *>(&C);
        m_x0     = LS.xBegin();
        m_y0     = LS.yBegin();
        m_theta0 = LS.m_theta0;
        m_c0     = LS.m_c0;
        m_s0     = LS.m_s0;
        m_k      = 0;
        m_L      = LS.length();
      }
      break;
    case G2LIB_CIRCLE:
      *this = *static_cast<CircleArc const *>(&C);
      break;
    case G2LIB_CLOTHOID:
    case G2LIB_BIARC:
    case G2LIB_BIARC_LIST:
    case G2LIB_CLOTHOID_LIST:
    case G2LIB_POLYLINE:
      UTILS_ERROR(
        "CircleArc constructor cannot convert from: {}\n",
        CurveType_name[C.type()]
      );
    }
  }

  // - - - - - - - - - - - - - - - - - - - - - - - - - - - - - - - - - - - - - -

  bool
  CircleArc::build_G1(
    real_type x0,
    real_type y0,
    real_type theta0,
    real_type x1,
    real_type y1
  ) {

    real_type dx = x1 - x0;
    real_type dy = y1 - y0;
    real_type d  = hypot( dx, dy );

    if ( d > 0 ) {
      real_type th = atan2( dy, dx ) - theta0;
      m_x0     = x0;
      m_y0     = y0;
      m_theta0 = theta0;
      m_k      = 2*sin(th)/d;
      m_L      = d/Sinc(th);
      return true;
    }
    return false;
  }

  // - - - - - - - - - - - - - - - - - - - - - - - - - - - - - - - - - - - - - -

  bool
  CircleArc::build_3P(
    real_type _x0,
    real_type _y0,
    real_type _x1,
    real_type _y1,
    real_type _x2,
    real_type _y2
  ) {
    real_type dxa   = _x1 - _x0;
    real_type dya   = _y1 - _y0;
    real_type dxb   = _x2 - _x1;
    real_type dyb   = _y2 - _y1;
    real_type La    = hypot(dya,dxa);
    real_type Lb    = hypot(dyb,dxb);
    real_type cosom = (dxa*dxb + dya*dyb)/(La*Lb);
    if      ( cosom >  1 ) cosom = 1;
    else if ( cosom < -1 ) cosom = -1;
    real_type omega = acos(cosom);

    real_type alpha = omega - atan2(Lb*sin(omega),La+Lb*cos(omega));
    real_type dxc   = _x2 - _x0;
    real_type dyc   = _y2 - _y0;
    real_type Lc    = hypot(dyc,dxc);
    real_type cosal = (dxa*dxc + dya*dyc)/(La*Lc);
    if      ( cosal >  1 ) cosal = 1;
    else if ( cosal < -1 ) cosal = -1;
    alpha += acos(cosal);

    if ( dxa*dyb > dya*dxb ) alpha = -alpha;
    real_type _theta0 = atan2( dyc, dxc ) + alpha;
    return build_G1( _x0, _y0, _theta0, _x2, _y2 );
  }

  // - - - - - - - - - - - - - - - - - - - - - - - - - - - - - - - - - - - - - -

  real_type
  CircleArc::thetaMinMax( real_type & thMin, real_type & thMax ) const  {
    thMin = m_theta0;
    thMax = m_theta0 + m_L * m_k;
    if ( thMax < thMin ) swap( thMin, thMax );
    return thMax-thMin;
  }

  // - - - - - - - - - - - - - - - - - - - - - - - - - - - - - - - - - - - - - -

  real_type
  CircleArc::X( real_type s ) const {
    real_type sk = (s*m_k)/2;
    return m_x0+s*Sinc(sk)*cos(m_theta0+sk);
  }

  real_type
  CircleArc::X_D( real_type s ) const {
    return cos(m_theta0+s*m_k);
  }

  real_type
  CircleArc::X_DD( real_type s ) const {
    return -m_k*sin(m_theta0+s*m_k);
  }

  real_type
  CircleArc::X_DDD( real_type s ) const {
    return -(m_k*m_k)*cos(m_theta0+s*m_k);
  }

  // - - - - - - - - - - - - - - - - - - - - - - - - - - - - - - - - - - - - - -

  real_type
  CircleArc::Y( real_type s ) const {
    real_type sk = (s*m_k)/2;
    return m_y0+s*Sinc(sk)*sin(m_theta0+sk);
  }

  real_type
  CircleArc::Y_D( real_type s ) const {
    return sin(m_theta0+s*m_k);
  }

  real_type
  CircleArc::Y_DD( real_type s ) const {
    return m_k*cos(m_theta0+s*m_k);
  }

  real_type
  CircleArc::Y_DDD( real_type s ) const {
    return -(m_k*m_k)*sin(m_theta0+s*m_k);
  }

  // - - - - - - - - - - - - - - - - - - - - - - - - - - - - - - - - - - - - - -

  /*\
   |  _____                   _   _   _
   | |_   _|   __ _ _ __   __| | | \ | |
   |   | |    / _` | '_ \ / _` | |  \| |
   |   | |   | (_| | | | | (_| | | |\  |
   |   |_|    \__,_|_| |_|\__,_| |_| \_|
  \*/

  void
  CircleArc::tg(
    real_type   s,
    real_type & tx,
    real_type & ty
  ) const {
    real_type th = theta(s);
    tx = cos(th);
    ty = sin(th);
  }

  void
  CircleArc::tg_D(
    real_type   s,
    real_type & tx_D,
    real_type & ty_D
  ) const {
    real_type th = theta(s);
    tx_D = -sin(th)*m_k;
    ty_D = cos(th)*m_k;
  }

  void
  CircleArc::tg_DD(
    real_type   s,
    real_type & tx_DD,
    real_type & ty_DD
  ) const {
    real_type th = theta(s);
    real_type k2 = m_k*m_k;
    tx_DD = -cos(th)*k2;
    ty_DD = -sin(th)*k2;
  }

  void
  CircleArc::tg_DDD(
    real_type   s,
    real_type & tx_DDD,
    real_type & ty_DDD
  ) const {
    real_type th = theta(s);
    real_type k3 = m_k*m_k*m_k;
    tx_DDD = sin(th)*k3;
    ty_DDD = -cos(th)*k3;
  }

  // - - - - - - - - - - - - - - - - - - - - - - - - - - - - - - - - - - - - - -

  void
  CircleArc::eval(
    real_type   s,
    real_type & x,
    real_type & y
  ) const {
    real_type sk  = (s*m_k)/2;
    real_type LS  = s*Sinc(sk);
    real_type arg = m_theta0+sk;
    x = m_x0+LS*cos(arg);
    y = m_y0+LS*sin(arg);
  }

  // - - - - - - - - - - - - - - - - - - - - - - - - - - - - - - - - - - - - - -

  void
  CircleArc::eval_D(
    real_type   s,
    real_type & x_D,
    real_type & y_D
  ) const {
    real_type arg = m_theta0+s*m_k;
    x_D = cos(arg);
    y_D = sin(arg);
  }

  // - - - - - - - - - - - - - - - - - - - - - - - - - - - - - - - - - - - - - -

  void
  CircleArc::eval_DD(
    real_type   s,
    real_type & x_DD,
    real_type & y_DD
  ) const {
    real_type arg = m_theta0+s*m_k;
    x_DD = -m_k*sin(arg);
    y_DD = m_k*cos(arg);
  }

  // - - - - - - - - - - - - - - - - - - - - - - - - - - - - - - - - - - - - - -

  void
  CircleArc::eval_DDD(
    real_type   s,
    real_type & x_DDD,
    real_type & y_DDD
  ) const {
    real_type arg = m_theta0+s*m_k;
    real_type k2  = m_k*m_k;
    x_DDD = -k2*cos(arg);
    y_DDD = -k2*sin(arg);
  }

  // - - - - - - - - - - - - - - - - - - - - - - - - - - - - - - - - - - - - - -

  void
  CircleArc::trim( real_type s_begin, real_type s_end ) {
    UTILS_ASSERT(
      s_end > s_begin,
      "CircleArc::trim( begin={}, s_end={} ) s_end must be > s_begin\n",
      s_begin, s_end
    );
    real_type x, y;
    eval( s_begin, x, y );
    m_theta0 += s_begin * m_k;
    m_L  = s_end - s_begin;
    m_x0 = x;
    m_y0 = y;
    m_c0 = cos(m_theta0);
    m_s0 = sin(m_theta0);
  }

  // - - - - - - - - - - - - - - - - - - - - - - - - - - - - - - - - - - - - - -

  void
  CircleArc::rotate( real_type angle, real_type cx, real_type cy ) {
    real_type dx  = m_x0 - cx;
    real_type dy  = m_y0 - cy;
    real_type C   = cos(angle);
    real_type S   = sin(angle);
    real_type ndx = C*dx - S*dy;
    real_type ndy = C*dy + S*dx;
    m_x0      = cx + ndx;
    m_y0      = cy + ndy;
    m_theta0 += angle;
    m_c0      = cos(m_theta0);
    m_s0      = sin(m_theta0);
  }

  // - - - - - - - - - - - - - - - - - - - - - - - - - - - - - - - - - - - - - -

  void
  CircleArc::scale( real_type s ) {
    m_k /= s;
    m_L *= s;
  }

  // - - - - - - - - - - - - - - - - - - - - - - - - - - - - - - - - - - - - - -

  void
  CircleArc::reverse() {
    real_type xx, yy;
    eval( m_L, xx, yy );
    m_theta0 += m_L*m_k+Utils::m_pi;
    while ( m_theta0 >  Utils::m_pi ) m_theta0 -= Utils::m_2pi;
    while ( m_theta0 < -Utils::m_pi ) m_theta0 += Utils::m_2pi;
    m_x0 = xx;
    m_y0 = yy;
    m_c0 = cos(m_theta0);
    m_s0 = sin(m_theta0);
    m_k  = -m_k;
  }

  // - - - - - - - - - - - - - - - - - - - - - - - - - - - - - - - - - - - - - -

  void
  CircleArc::center( real_type & cx, real_type & cy ) const {
    real_type nx = -sin(m_theta0);
    real_type ny = cos(m_theta0);
    cx = m_x0 + nx/m_k;
    cy = m_y0 + ny/m_k;
  }

  // - - - - - - - - - - - - - - - - - - - - - - - - - - - - - - - - - - - - - -

  void
  CircleArc::changeCurvilinearOrigin( real_type new_s0, real_type newL ) {
    real_type new_x0, new_y0;
    eval( new_s0,  new_x0, new_y0 );
    m_x0      = new_x0;
    m_y0      = new_y0;
    m_theta0 += m_k*new_s0;
    m_L       = newL;
  }

  // - - - - - - - - - - - - - - - - - - - - - - - - - - - - - - - - - - - - - -

  bool
  CircleArc::bbTriangle(
    real_type & xx0, real_type & yy0,
    real_type & xx1, real_type & yy1,
    real_type & xx2, real_type & yy2
  ) const {
    real_type dtheta = m_L * m_k;
    bool ok = abs(dtheta) <= Utils::m_pi/3;
    if ( ok ) {
      xx0 = m_x0;
      yy0 = m_y0;
      eval( m_L, xx2, yy2 );
      xx1 = (xx0+xx2)/2;
      yy1 = (yy0+yy2)/2;
      real_type nx = yy0-yy2;
      real_type ny = xx2-xx0;
      real_type tg = tan(dtheta/2)/2;
      xx1 -= nx * tg;
      yy1 -= ny * tg;
    }
    return ok;
  }

  // - - - - - - - - - - - - - - - - - - - - - - - - - - - - - - - - - - - - - -

  bool
  CircleArc::bbTriangle_ISO(
    real_type   offs,
    real_type & xx0, real_type & yy0,
    real_type & xx1, real_type & yy1,
    real_type & xx2, real_type & yy2
  ) const {
    real_type dtheta = m_L * m_k;
    bool ok = abs(dtheta) <= Utils::m_pi/3;
    if ( ok ) {
      eval_ISO( 0,   offs, xx0, yy0 );
      eval_ISO( m_L, offs, xx2, yy2 );
      xx1 = (xx0+xx2)/2;
      yy1 = (yy0+yy2)/2;
      real_type nx = yy0-yy2;
      real_type ny = xx2-xx0;
      real_type tg = tan(dtheta/2)/2;
      xx1 -= nx * tg;
      yy1 -= ny * tg;
    }
    return ok;
  }

  // - - - - - - - - - - - - - - - - - - - - - - - - - - - - - - - - - - - - - -

  void
  CircleArc::bbTriangles(
    vector<Triangle2D> & tvec,
    real_type            max_angle,
    real_type            max_size,
    int_type             icurve
  ) const {
    real_type dtheta = abs( min(m_L,max_size) * m_k);
    int_type  n      = 1;
    if ( dtheta > max_angle ) {
      n       = int_type(ceil( dtheta/max_angle ));
      dtheta /= n;
    }
    real_type tg = tan(dtheta/2)/2;
    if ( m_k < 0 ) tg = -tg;
    tvec.reserve( size_t(n) );
    real_type xx0 = m_x0;
    real_type yy0 = m_y0;
    real_type ds  = m_L/n;
    real_type ss  = ds;
    for ( int_type iter = 0; iter < n; ++iter, ss += ds ) {
      real_type xx2, yy2;
      eval( ss, xx2, yy2 );
      real_type xx1 = (xx0+xx2)/2;
      real_type yy1 = (yy0+yy2)/2;
      real_type nx  = yy0-yy2;
      real_type ny  = xx2-xx0;
      xx1 -= nx * tg;
      yy1 -= ny * tg;
      tvec.push_back( Triangle2D( xx0, yy0, xx1, yy1, xx2, yy2, 0, 0, icurve ) );
      xx0 = xx2;
      yy0 = yy2;
    }
  }

  // - - - - - - - - - - - - - - - - - - - - - - - - - - - - - - - - - - - - - -

  void
  CircleArc::bbTriangles_ISO(
    real_type            offs,
    vector<Triangle2D> & tvec,
    real_type            max_angle,
    real_type            max_size,
    int_type             icurve
  ) const {
    real_type scale  = 1+m_k*offs;
    real_type dtheta = abs( min(m_L,max_size/scale) * m_k );
    int_type  n      = 1;
    if ( dtheta > max_angle ) {
      n       = int_type(ceil( dtheta/max_angle ));
      dtheta /= n;
    }
    tvec.reserve( size_t(n) );
    real_type ds = m_L/n;
    real_type ss = ds;
    real_type tg = scale * tan(dtheta/2)/2;
    if ( m_k < 0 ) tg = -tg;
    real_type xx0, yy0;
    eval_ISO( 0, offs, xx0, yy0 );
    for ( int_type iter = 0; iter < n; ++iter, ss += ds ) {
      real_type xx2, yy2;
      eval_ISO( ss, offs, xx2, yy2 );
      real_type xx1 = (xx0+xx2)/2;
      real_type yy1 = (yy0+yy2)/2;
      real_type nx = yy0-yy2;
      real_type ny = xx2-xx0;
      xx1 -= nx * tg;
      yy1 -= ny * tg;
      tvec.push_back( Triangle2D( xx0, yy0, xx1, yy1, xx2, yy2, 0, 0, icurve ) );
      xx0 = xx2;
      yy0 = yy2;
    }
  }

  // - - - - - - - - - - - - - - - - - - - - - - - - - - - - - - - - - - - - - -

  void
  CircleArc::bbox(
    real_type & xmin,
    real_type & ymin,
    real_type & xmax,
    real_type & ymax
  ) const {
    vector<Triangle2D> tvec;
    this->bbTriangles( tvec, Utils::m_pi/4 );
    tvec[0].bbox( xmin, ymin, xmax, ymax );
    for ( int_type iter = 1; iter < int_type(tvec.size()); ++iter ) {
      real_type xmin1, ymin1, xmax1, ymax1;
      tvec[size_t(iter)].bbox( xmin1, ymin1, xmax1, ymax1 );
      if ( xmin1 < xmin ) xmin = xmin1;
      if ( ymin1 < ymin ) ymin = ymin1;
      if ( xmax1 > xmax ) xmax = xmax1;
      if ( ymax1 > ymax ) ymax = ymax1;
    }
  }

  // - - - - - - - - - - - - - - - - - - - - - - - - - - - - - - - - - - - - - -

  void
  CircleArc::bbox_ISO(
    real_type   offs,
    real_type & xmin,
    real_type & ymin,
    real_type & xmax,
    real_type & ymax
  ) const {
    vector<Triangle2D> tvec;
    this->bbTriangles_ISO( offs, tvec, Utils::m_pi/4 );
    tvec[0].bbox( xmin, ymin, xmax, ymax );
    for ( int_type iter = 1; iter < int_type(tvec.size()); ++iter ) {
      real_type xmin1, ymin1, xmax1, ymax1;
      tvec[size_t(iter)].bbox( xmin1, ymin1, xmax1, ymax1 );
      if ( xmin1 < xmin ) xmin = xmin1;
      if ( ymin1 < ymin ) ymin = ymin1;
      if ( xmax1 > xmax ) xmax = xmax1;
      if ( ymax1 > ymax ) ymax = ymax1;
    }
  }

  /*\
   |   _       _                          _
   |  (_)_ __ | |_ ___ _ __ ___  ___  ___| |_
   |  | | '_ \| __/ _ \ '__/ __|/ _ \/ __| __|
   |  | | | | | ||  __/ |  \__ \  __/ (__| |_
   |  |_|_| |_|\__\___|_|  |___/\___|\___|\__|
  \*/

  // - - - - - - - - - - - - - - - - - - - - - - - - - - - - - - - - - - - - - -

  bool
  CircleArc::collision( CircleArc const & C ) const {
    real_type s1[2], s2[2];
    int_type ni = intersectCircleCircle(
      m_x0, m_y0, m_theta0, m_k,
      C.m_x0, C.m_y0, C.m_theta0, C.m_k, s1, s2
    );
    real_type eps1 = machepsi100*m_L;
    real_type eps2 = machepsi100*C.m_L;
    for ( int_type i = 0; i < ni; ++i ) {
      if ( s1[i] >= -eps1 && s1[i] <= m_L+eps1 &&
           s2[i] >= -eps2 && s2[i] <= m_L+eps2 )
        return true;
    }
    return false;
  }

  // - - - - - - - - - - - - - - - - - - - - - - - - - - - - - - - - - - - - - -

  bool
  CircleArc::collision_ISO(
    real_type         offs,
    CircleArc const & C,
    real_type         offs_C
  ) const {
    real_type s1[2], s2[2];
    real_type sc1 = 1+m_k*offs;
    real_type sc2 = 1+C.m_k*offs_C;
    int_type ni = intersectCircleCircle(
      this->X_ISO(0,offs),
      this->Y_ISO(0,offs),
      m_theta0,
      m_k/sc2,
      C.X_ISO(0,offs_C),
      C.Y_ISO(0,offs_C),
      C.m_theta0,
      C.m_k/sc2,
      s1, s2
    );
    real_type eps1 = machepsi100*m_L;
    real_type eps2 = machepsi100*C.m_L;
    for ( int_type i = 0; i < ni; ++i ) {
      real_type ss1 = s1[i]/sc1;
      real_type ss2 = s2[i]/sc2;
      if ( ss1 >= -eps1 && ss1 <= m_L+eps1 &&
           ss2 >= -eps2 && ss2 <= C.m_L+eps2 )
        return true;
    }
    return false;
  }

  // - - - - - - - - - - - - - - - - - - - - - - - - - - - - - - - - - - - - - -

  void
  CircleArc::intersect(
    CircleArc const & C,
    IntersectList   & ilist,
    bool              swap_s_vals
  ) const {
    real_type s1[2], s2[2];
    int_type ni = intersectCircleCircle(
      m_x0, m_y0, m_theta0, m_k,
      C.m_x0, C.m_y0, C.m_theta0, C.m_k, s1, s2
    );
    real_type eps1 = machepsi100*m_L;
    real_type eps2 = machepsi100*C.m_L;
    for ( int_type i = 0; i < ni; ++i ) {
      real_type ss1 = s1[i];
      real_type ss2 = s2[i];
      if ( ss1 >= -eps1 && ss1 <= m_L+eps1 &&
           ss2 >= -eps2 && ss2 <= C.m_L+eps2 ) {
        if ( swap_s_vals ) ilist.push_back( Ipair(ss2,ss1) );
        else               ilist.push_back( Ipair(ss1,ss2) );
      }
    }
  }

  // - - - - - - - - - - - - - - - - - - - - - - - - - - - - - - - - - - - - - -

  void
  CircleArc::intersect_ISO(
    real_type         offs,
    CircleArc const & C,
    real_type         offs_C,
    IntersectList   & ilist,
    bool              swap_s_vals
  ) const {
    real_type s1[2], s2[2];
    real_type sc1 = 1+m_k*offs;
    real_type sc2 = 1+C.m_k*offs_C;
    int_type ni = intersectCircleCircle(
      this->X_ISO(0,offs),
      this->Y_ISO(0,offs),
      m_theta0,
      m_k/sc2,
      C.X_ISO(0,offs_C),
      C.Y_ISO(0,offs_C),
      C.m_theta0,
      C.m_k/sc2,
      s1, s2
    );
    real_type eps1 = machepsi100*m_L;
    real_type eps2 = machepsi100*C.m_L;
    for ( int_type i = 0; i < ni; ++i ) {
      real_type ss1 = s1[i]/sc1;
      real_type ss2 = s2[i]/sc2;
      if ( ss1 >= -eps1 && ss1 <= m_L+eps1 &&
           ss2 >= -eps2 && ss2 <= C.m_L+eps2 ) {
        if ( swap_s_vals ) ilist.push_back( Ipair(ss2,ss1) );
        else               ilist.push_back( Ipair(ss1,ss2) );
      }
    }
  }

  /*\
   |        _                     _   ____       _       _
   |    ___| | ___  ___  ___  ___| |_|  _ \ ___ (_)_ __ | |_
   |   / __| |/ _ \/ __|/ _ \/ __| __| |_) / _ \| | '_ \| __|
   |  | (__| | (_) \__ \  __/\__ \ |_|  __/ (_) | | | | | |_
   |   \___|_|\___/|___/\___||___/\__|_|   \___/|_|_| |_|\__|
  \*/

  int_type
  CircleArc::closestPoint_ISO(
    real_type   qx,
    real_type   qy,
    real_type & x,
    real_type & y,
    real_type & s,
    real_type & t,
    real_type & dst
  ) const {
    real_type cc0 = cos(m_theta0);
    real_type ss0 = sin(m_theta0);
    s = projectPointOnCircleArc( m_x0, m_y0, cc0, ss0, m_k, m_L, qx, qy );
    int_type res = 1;
    if ( s < 0 || s > m_L ) {
      s = m_L;
      t = 0;
      eval( s, x, y );
      // costruisco piano
      real_type nx = x-m_x0;
      real_type ny = y-m_y0;
      real_type dx = 2*qx-(m_x0+x);
      real_type dy = 2*qy-(m_y0+y);
      if ( nx*dx + ny*dy <= 0 ) {
        s = 0;
        x = m_x0;
        y = m_y0;
      }
      res = -1;
    } else {
      eval( s, x, y );
    }
    real_type nx, ny;
    nor_ISO( s, nx, ny );
    real_type dx = qx-x;
    real_type dy = qy-y;
    t   = dx * nx + dy * ny;
    dst = hypot( dx, dy );
    return res;
  }

  // - - - - - - - - - - - - - - - - - - - - - - - - - - - - - - - - - - - - - -

  int_type
  CircleArc::closestPoint_ISO(
    real_type   qx,
    real_type   qy,
    real_type   offs,
    real_type & x,
    real_type & y,
    real_type & s,
    real_type & t,
    real_type & dst
  ) const  {
    real_type cc0 = cos(m_theta0);
    real_type ss0 = sin(m_theta0);
    real_type xx0 = m_x0+offs*nx_Begin_ISO();
    real_type yy0 = m_y0+offs*ny_Begin_ISO();
    real_type ff  = 1+m_k*offs;
    real_type LL  = m_L*ff;
    s = projectPointOnCircleArc( xx0, yy0, cc0, ss0, m_k/ff, LL, qx, qy );
    int_type res = 1;
    if ( s < 0 || s > LL ) {
      s = m_L;
      eval_ISO( s, offs, x, y );
      // costruisco piano
      real_type nx = x-xx0;
      real_type ny = y-yy0;
      real_type dx = 2*qx-(xx0+x);
      real_type dy = 2*qy-(yy0+y);
      if ( nx*dx + ny*dy <= 0 ) {
        s = 0;
        x = xx0;
        y = yy0;
      }
      res = -1;
    } else {
      eval_ISO( s, offs, x, y );
    }
    real_type nx, ny;
    nor_ISO( s, nx, ny );
    real_type dx = qx-x;
    real_type dy = qy-y;
    t   = dx * nx + dy * ny + offs;
    dst = hypot( dx, dy );
    return res;
  }

  // - - - - - - - - - - - - - - - - - - - - - - - - - - - - - - - - - - - - - -

  void
  CircleArc::paramNURBS(
    int_type & n_knots,
    int_type & n_pnts
  ) const {
    real_type dtheta = m_L*m_k;
    int_type  ns     = int_type(floor(3*abs(dtheta)/Utils::m_pi));
    if ( ns < 1 ) ns = 1;
    n_pnts  = 1+2*ns;
    n_knots = n_pnts+3;
  }

  // - - - - - - - - - - - - - - - - - - - - - - - - - - - - - - - - - - - - - -

  void
  CircleArc::toNURBS(
    real_type * knots,
    real_type   Poly[][3]
  ) const {

    real_type dtheta = m_L*m_k;
    int_type  ns     = int_type(floor(3*abs(dtheta)/Utils::m_pi));
    if ( ns < 1 ) ns = 1;

    real_type th = dtheta/(2*ns);
    real_type w  = cos(th);
    real_type tg = tan(th)/2;

    real_type p0[2], p2[2];
    p0[0] = m_x0;
    p0[1] = m_y0;

    knots[0] = knots[1] = knots[2] = 0;
    Poly[0][0] = p0[0];
    Poly[0][1] = p0[1];
    Poly[0][2] = 1;

    real_type s  = 0;
    real_type ds = m_L/ns;
    int_type  kk = 0;
    for ( int_type i = 0; i < ns; ++i ) {
      s += ds;
      eval( s, p2[0], p2[1] );

      real_type nx = p0[1]-p2[1];
      real_type ny = p2[0]-p0[0];
      real_type xm = (p0[0]+p2[0])/2;
      real_type ym = (p0[1]+p2[1])/2;

      ++kk;
      Poly[kk][0] = w*(xm - nx * tg);
      Poly[kk][1] = w*(ym - ny * tg);
      Poly[kk][2] = w;

      ++kk;
      Poly[kk][0] = p2[0];
      Poly[kk][1] = p2[1];
      Poly[kk][2] = 1;

      knots[kk+1] = i+1;
      knots[kk+2] = i+1;

      p0[0] = p2[0];
      p0[1] = p2[1];

    }
    knots[kk+3] = ns;
  }

  // - - - - - - - - - - - - - - - - - - - - - - - - - - - - - - - - - - - - - -

  real_type
  CircleArc::lenTolerance( real_type tol ) const {
    real_type absk = abs(m_k);
    real_type tmp  = absk*tol;
    if ( tmp > 0 ) {
      real_type dtheta = 2*(Utils::m_pi-acos(tmp-1));
      return dtheta/absk;
    } else {
      return m_L;
    }
  }

  // - - - - - - - - - - - - - - - - - - - - - - - - - - - - - - - - - - - - - -

  ostream_type &
  operator << ( ostream_type & stream, CircleArc const & c ) {
    fmt::print( stream,
      "x0     = {}\n"
      "y0     = {}\n"
      "theta0 = {}\n"
      "k      = {}\n"
      "L      = {}\n",
      c.m_x0, c.m_y0, c.m_theta0, c.m_k, c.m_L
    );
    return stream;
  }

}

// EOF: Circle.cc
```

### Quick search

### Table of Contents

- Matlab Interface Manual
- C++ API
- MATLAB API

«
hide menu

menu
sidebar
»

### Navigation

- index
- toc
- Clothoids »
- Program Listing for File Circle.cc

© Copyright 2021, Enrico Bertolazzi and Marco Frego.
Created using Sphinx 4.2.0.
