# Supplementary material for: Smooth Interpolating Curves with Local Control and Monotone Alternating Curvature
Source: Comput Graph Forum. 2022 Oct 6;41(5):25–38. doi: 10.1111/cgf.14600 (PMC9827861; doi:10.1111/cgf.14600)
Supplement: Supplementary file 1 — Supplement Material [file CGF-41-25-s001.zip › Local-Smooth-Interpolating-MonoCurvature/extern/clothoids/docs/api-cpp/program_listing_file_Clothoid.cc.html]

Program Listing for File Clothoid.cc — Clothoids v2.0.9

### Navigation

- index
- toc
- Clothoids »
- Program Listing for File Clothoid.cc

# Program Listing for File Clothoid.cc¶

↰ Return to documentation for file (`Clothoid.cc`)

```
/*--------------------------------------------------------------------------*\
 |                                                                          |
 |  Copyright (C) 2017                                                      |
 |                                                                          |
 |         , __                 , __                                        |
 |        /|/  \               /|/  \                                       |
 |         | __/ _   ,_         | __/ _   ,_                                |
 |         |   \|/  /  |  |   | |   \|/  /  |  |   |                        |
 |         |(__/|__/   |_/ \_/|/|(__/|__/   |_/ \_/|/                       |
 |                           /|                   /|                        |
 |                           \|                   \|                        |
 |                                                                          |
 |      Enrico Bertolazzi                                                   |
 |      Dipartimento di Ingegneria Industriale                              |
 |      Universita` degli Studi di Trento                                   |
 |      email: enrico.bertolazzi@unitn.it                                   |
 |                                                                          |
\*--------------------------------------------------------------------------*/

#include "Clothoids.hh"

// workaround for windows that defines max and min as macros!
#ifdef max
  #undef max
#endif
#ifdef min
  #undef min
#endif

#include <cmath>
#include <cfloat>
#include <algorithm>

namespace G2lib {

  using std::vector;
  using std::abs;
  using std::min;
  using std::max;
  using std::swap;
  using std::ceil;
  using std::floor;
  using std::isfinite;
  using std::numeric_limits;

  int_type  ClothoidCurve::m_max_iter  = 10;
  real_type ClothoidCurve::m_tolerance = 1e-9;

  // . . . . . . . . . . . . . . . . . . . . . . . . . . . . . . . . . . . . .

  ClothoidCurve::ClothoidCurve( BaseCurve const & C )
  : BaseCurve(G2LIB_CLOTHOID)
  , m_aabb_done(false)
  {
    switch ( C.type() ) {
    case G2LIB_LINE:
      build( *static_cast<LineSegment const *>(&C) );
      break;
    case G2LIB_CIRCLE:
      build( *static_cast<CircleArc const *>(&C) );
      break;
    case G2LIB_CLOTHOID:
      copy( *static_cast<ClothoidCurve const *>(&C) );
      break;
    case G2LIB_BIARC:
    case G2LIB_BIARC_LIST:
    case G2LIB_CLOTHOID_LIST:
    case G2LIB_POLYLINE:
      UTILS_ERROR(
        "ClothoidList constructor cannot convert from: {}\n",
        CurveType_name[C.type()]
      );
    }
  }

  // . . . . . . . . . . . . . . . . . . . . . . . . . . . . . . . . . . . . .

  void
  ClothoidCurve::build(
    real_type _x0,
    real_type _y0,
    real_type _theta0,
    real_type _k,
    real_type _dk,
    real_type _L
  ) {
    UTILS_ASSERT(
      _L > 0,
      "ClothoidCurve::build( x0={}, y0={}, theta0={}, k={}, dk={}, L={} )\n"
      "L must be positive!\n",
      _x0, _y0, _theta0, _k, _dk, _L
    );
    m_CD.x0     = _x0;
    m_CD.y0     = _y0;
    m_CD.theta0 = _theta0;
    m_CD.kappa0 = _k;
    m_CD.dk     = _dk;
    m_L         = _L;
    m_aabb_done = false;
    m_aabb_tree.clear();
  }

  // . . . . . . . . . . . . . . . . . . . . . . . . . . . . . . . . . . . . .

  void
  ClothoidCurve::optimized_sample_internal_ISO(
    real_type           s_begin,
    real_type           s_end,
    real_type           offs,
    real_type           ds,
    real_type           max_angle,
    vector<real_type> & s
  ) const {
    real_type ss  = s_begin;
    real_type thh = theta(s_begin);
    for ( int_type npts = 0; ss < s_end; ++npts ) {
      UTILS_ASSERT0(
        npts < 100000000,
        "ClothoidCurve::optimized_sample_internal "
        "is generating too much points (>100000000)\n"
        "something is going wrong or parameters are not well set\n"
      );
      // estimate angle variation and compute step accodingly
      real_type k   = m_CD.kappa( ss );
      real_type dss = ds/(1+k*offs); // scale length with offset
      real_type sss = ss + dss;
      if ( sss > s_end ) {
        sss = s_end;
        dss = s_end-ss;
      }
      if ( abs(k*dss) > max_angle ) {
        dss = abs(max_angle/k);
        sss = ss + dss;
      }
      // check and recompute if necessary
      real_type thhh = theta(sss);
      if ( abs(thh-thhh) > max_angle ) {
        k    = m_CD.kappa( sss );
        dss  = abs(max_angle/k);
        sss  = ss + dss;
        thhh = theta(sss);
      }
      ss  = sss;
      thh = thhh;
      s.push_back(ss);
    }
    s.back() = s_end;
  }

  // . . . . . . . . . . . . . . . . . . . . . . . . . . . . . . . . . . . . .

  void
  ClothoidCurve::optimized_sample_ISO(
    real_type           offs,
    int_type            npts,
    real_type           max_angle,
    vector<real_type> & s
  ) const {
    s.clear();
    s.reserve( size_t(npts) );
    s.push_back(0);

    real_type ds = m_L/npts;
    if ( m_CD.kappa0*m_CD.dk >= 0 || m_CD.kappa(m_L)*m_CD.dk <= 0 ) {
      optimized_sample_internal_ISO( 0, m_L, offs, ds, max_angle, s );
    } else {
      // flex inside, split clothoid
      real_type sflex = -m_CD.kappa0/m_CD.dk;
      optimized_sample_internal_ISO( 0,   sflex, offs, ds, max_angle, s );
      optimized_sample_internal_ISO( sflex, m_L, offs, ds, max_angle, s );
    }
  }

  // . . . . . . . . . . . . . . . . . . . . . . . . . . . . . . . . . . . . .

  /*\
   |  _    _   _____    _                _
   | | |__| |_|_   _| _(_)__ _ _ _  __ _| |___
   | | '_ \ '_ \| || '_| / _` | ' \/ _` | / -_)
   | |_.__/_.__/|_||_| |_\__,_|_||_\__, |_\___|
   |                               |___/
  \*/

  // . . . . . . . . . . . . . . . . . . . . . . . . . . . . . . . . . . . . .

  void
  ClothoidCurve::bbTriangles_internal_ISO(
    real_type            offs,
    vector<Triangle2D> & tvec,
    real_type            s_begin,
    real_type            s_end,
    real_type            max_angle,
    real_type            max_size,
    int_type             icurve
  ) const {

    static real_type const one_degree = Utils::m_pi/180;

    real_type ss  = s_begin;
    real_type thh = m_CD.theta(ss);
    real_type MX  = min( m_L, max_size );
    for ( int_type npts = 0; ss < s_end; ++npts ) {
      UTILS_ASSERT0(
        npts < 100000000,
        "ClothoidCurve::bbTriangles_internal "
        "is generating too much triangles (>100000000)\n"
        "something is going wrong or parameters are not well set\n"
      );

      // estimate angle variation and compute step accodingly
      real_type k   = m_CD.kappa( ss );
      real_type dss = MX/(1+k*offs); // scale length with offset
      real_type sss = ss + dss;
      if ( sss > s_end ) {
        sss = s_end;
        dss = s_end-ss;
      }
      if ( abs(k*dss) > max_angle ) {
        dss = abs(max_angle/k);
        sss = ss + dss;
      }
      // check and recompute if necessary
      real_type thhh = theta(sss);
      if ( abs(thh-thhh) > max_angle ) {
        k    = m_CD.kappa( sss );
        dss  = abs(max_angle/k);
        sss  = ss + dss;
        thhh = theta(sss);
      }

      real_type x0, y0, x1, y1;
      m_CD.eval_ISO( ss,  offs, x0, y0 );
      m_CD.eval_ISO( sss, offs, x1, y1 );

      real_type tx0    = cos(thh);
      real_type ty0    = sin(thh);
      real_type alpha  = sss-ss; // se angolo troppo piccolo uso approx piu rozza
      if ( abs(thh-thhh) > one_degree ) {
        real_type tx1 = cos(thhh);
        real_type ty1 = sin(thhh);
        real_type det = tx1 * ty0 - tx0 * ty1;
        real_type dx  = x1-x0;
        real_type dy  = y1-y0;
        alpha = (dy*tx1 - dx*ty1)/det;
      }

      real_type x2 = x0 + alpha*tx0;
      real_type y2 = y0 + alpha*ty0;
      Triangle2D t( x0, y0, x2, y2, x1, y1, ss, sss, icurve );

      tvec.push_back( t );

      ss  = sss;
      thh = thhh;
    }
  }

  // . . . . . . . . . . . . . . . . . . . . . . . . . . . . . . . . . . . . .

  void
  ClothoidCurve::bbTriangles_ISO(
    real_type            offs,
    vector<Triangle2D> & tvec,
    real_type            max_angle,
    real_type            max_size,
    int_type             icurve
  ) const {
    if ( m_CD.kappa0*m_CD.dk >= 0 || m_CD.kappa(m_L)*m_CD.dk <= 0 ) {
      bbTriangles_internal_ISO( offs, tvec, 0, m_L, max_angle, max_size, icurve );
    } else {
      // flex inside, split clothoid
      real_type sflex = -m_CD.kappa0/m_CD.dk;
      bbTriangles_internal_ISO( offs, tvec, 0,   sflex, max_angle, max_size, icurve );
      bbTriangles_internal_ISO( offs, tvec, sflex, m_L, max_angle, max_size, icurve );
    }
  }

  // . . . . . . . . . . . . . . . . . . . . . . . . . . . . . . . . . . . . .
  /*\
   |  ___ ___
   | | _ ) _ ) _____ __
   | | _ \ _ \/ _ \ \ /
   | |___/___/\___/_\_\
  \*/

  void
  ClothoidCurve::bbox_ISO(
    real_type   offs,
    real_type & xmin,
    real_type & ymin,
    real_type & xmax,
    real_type & ymax
  ) const {
    vector<Triangle2D> tvec;
    bbTriangles_ISO( offs, tvec, Utils::m_pi/18, 1e100 );
    xmin = ymin = numeric_limits<real_type>::infinity();
    xmax = ymax = -xmin;
    vector<Triangle2D>::const_iterator it;
    for ( it = tvec.begin(); it != tvec.end(); ++it ) {
      // - - - - - - - - - - - - - - - - - - - -
      if      ( it->x1() < xmin ) xmin = it->x1();
      else if ( it->x1() > xmax ) xmax = it->x1();
      if      ( it->x2() < xmin ) xmin = it->x2();
      else if ( it->x2() > xmax ) xmax = it->x2();
      if      ( it->x3() < xmin ) xmin = it->x3();
      else if ( it->x3() > xmax ) xmax = it->x3();
      // - - - - - - - - - - - - - - - - - - - -
      if      ( it->y1() < ymin ) ymin = it->y1();
      else if ( it->y1() > ymax ) ymax = it->y1();
      if      ( it->y2() < ymin ) ymin = it->y2();
      else if ( it->y2() > ymax ) ymax = it->y2();
      if      ( it->y3() < ymin ) ymin = it->y3();
      else if ( it->y3() > ymax ) ymax = it->y3();
    }
  }

  // . . . . . . . . . . . . . . . . . . . . . . . . . . . . . . . . . . . . .
  /*\
   |     _        _    ____  ____  _
   |    / \      / \  | __ )| __ )| |_ _ __ ___  ___
   |   / _ \    / _ \ |  _ \|  _ \| __| '__/ _ \/ _ \
   |  / ___ \  / ___ \| |_) | |_) | |_| | |  __/  __/
   | /_/   \_\/_/   \_\____/|____/ \__|_|  \___|\___|
  \*/

  #ifndef DOXYGEN_SHOULD_SKIP_THIS
  void
  ClothoidCurve::build_AABBtree_ISO(
    real_type offs,
    real_type max_angle,
    real_type max_size
  ) const {

    if ( m_aabb_done &&
         Utils::isZero( offs-m_aabb_offs ) &&
         Utils::isZero( max_angle-m_aabb_max_angle ) &&
         Utils::isZero( max_size-m_aabb_max_size ) ) return;

    #ifdef G2LIB_USE_CXX11
    vector<shared_ptr<BBox const> > bboxes;
    #else
    vector<BBox const *> bboxes;
    #endif

    bbTriangles_ISO( offs, m_aabb_tri, max_angle, max_size );
    bboxes.reserve(m_aabb_tri.size());
    vector<Triangle2D>::const_iterator it;
    int_type ipos = 0;
    for ( it = m_aabb_tri.begin(); it != m_aabb_tri.end(); ++it, ++ipos ) {
      real_type xmin, ymin, xmax, ymax;
      it->bbox( xmin, ymin, xmax, ymax );
      #ifdef G2LIB_USE_CXX11
      bboxes.push_back( make_shared<BBox const>(
        xmin, ymin, xmax, ymax, G2LIB_CLOTHOID, ipos
      ) );
      #else
      bboxes.push_back(
        new BBox( xmin, ymin, xmax, ymax, G2LIB_CLOTHOID, ipos )
      );
      #endif
    }
    m_aabb_tree.build(bboxes);
    m_aabb_done      = true;
    m_aabb_offs      = offs;
    m_aabb_max_angle = max_angle;
    m_aabb_max_size  = max_size;
  }
  #endif

  // - - - - - - - - - - - - - - - - - - - - - - - - - - - - - - - - - - - - - -

  /*\
   |            _ _ _     _
   |   ___ ___ | | (_)___(_) ___  _ __
   |  / __/ _ \| | | / __| |/ _ \| '_ \
   | | (_| (_) | | | \__ \ | (_) | | | |
   |  \___\___/|_|_|_|___/_|\___/|_| |_|
  \*/

  bool
  ClothoidCurve::collision( ClothoidCurve const & C ) const {
    this->build_AABBtree_ISO( 0 );
    C.build_AABBtree_ISO( 0 );
    T2D_collision_ISO fun( this, 0, &C, 0 );
    return m_aabb_tree.collision( C.m_aabb_tree, fun, false );
  }

  // - - - - - - - - - - - - - - - - - - - - - - - - - - - - - - - - - - - - - -

  bool
  ClothoidCurve::collision_ISO(
    real_type             offs,
    ClothoidCurve const & C,
    real_type             offs_C
  ) const {
    this->build_AABBtree_ISO( offs );
    C.build_AABBtree_ISO( offs_C );
    T2D_collision_ISO fun( this, offs, &C, offs_C );
    return m_aabb_tree.collision( C.m_aabb_tree, fun, false );
  }

  // - - - - - - - - - - - - - - - - - - - - - - - - - - - - - - - - - - - - - -

  bool
  ClothoidCurve::approximate_collision_ISO(
    real_type             offs,
    ClothoidCurve const & C,
    real_type             offs_C,
    real_type             max_angle,
    real_type             max_size
  ) const {
    this->build_AABBtree_ISO( offs, max_angle, max_size );
    C.build_AABBtree_ISO( offs_C, max_angle, max_size );
    T2D_approximate_collision fun( this, &C );
    return m_aabb_tree.collision( C.m_aabb_tree, fun, false );
  }

  // - - - - - - - - - - - - - - - - - - - - - - - - - - - - - - - - - - - - - -

  /*\
   |  _       _                          _
   | (_)_ __ | |_ ___ _ __ ___  ___  ___| |_
   | | | '_ \| __/ _ \ '__/ __|/ _ \/ __| __|
   | | | | | | ||  __/ |  \__ \  __/ (__| |_
   | |_|_| |_|\__\___|_|  |___/\___|\___|\__|
  \*/

  // - - - - - - - - - - - - - - - - - - - - - - - - - - - - - - - - - - - - - -

  bool
  ClothoidCurve::aabb_intersect_ISO(
    Triangle2D    const & T1,
    real_type             offs,
    ClothoidCurve const * pC,
    Triangle2D    const & T2,
    real_type             offs_C,
    real_type           & ss1,
    real_type           & ss2
  ) const {
    real_type eps1   = machepsi1000*m_L;
    real_type eps2   = machepsi1000*pC->m_L;
    real_type s1_min = T1.S0()-eps1;
    real_type s1_max = T1.S1()+eps1;
    real_type s2_min = T2.S0()-eps2;
    real_type s2_max = T2.S1()+eps2;
    int_type  nout   = 0;
    bool converged   = false;

    ss1 = (s1_min+s1_max)/2;
    ss2 = (s2_min+s2_max)/2;
    for ( int_type i = 0; i < m_max_iter && !converged; ++i ) {
      real_type t1[2], t2[2], p1[2], p2[2];
      m_CD.eval_ISO  ( ss1, offs, p1[0], p1[1] );
      m_CD.eval_ISO_D( ss1, offs, t1[0], t1[1] );
      pC->m_CD.eval_ISO  ( ss2, offs_C, p2[0], p2[1] );
      pC->m_CD.eval_ISO_D( ss2, offs_C, t2[0], t2[1] );
      /*
      // risolvo il sistema
      // p1 + alpha * t1 = p2 + beta * t2
      // alpha * t1 - beta * t2 = p2 - p1
      //
      //  / t1[0] -t2[0] \ / alpha \ = / p2[0] - p1[0] \
      //  \ t1[1] -t2[1] / \ beta  /   \ p2[1] - p1[1] /
      */
      real_type det = t2[0]*t1[1]-t1[0]*t2[1];
      real_type px  = p2[0]-p1[0];
      real_type py  = p2[1]-p1[1];
      ss1 += (py*t2[0] - px*t2[1])/det;
      ss2 += (t1[0]*py - t1[1]*px)/det;
      if ( ! ( isfinite(ss1) && isfinite(ss1) ) ) break;
      bool out = false;
      if      ( ss1 < s1_min ) { out = true; ss1 = s1_min; }
      else if ( ss1 > s1_max ) { out = true; ss1 = s1_max; }
      if      ( ss2 < s2_min ) { out = true; ss2 = s2_min; }
      else if ( ss2 > s2_max ) { out = true; ss2 = s2_max; }
      if ( out ) {
        if ( ++nout > 3 ) break;
      } else {
        converged = abs(px) <= m_tolerance && abs(py) <= m_tolerance;
      }
    }
    if ( converged ) {
      if      ( ss1 < T1.S0() ) ss1 = T1.S0();
      else if ( ss1 > T1.S1() ) ss1 = T1.S1();
      if      ( ss2 < T2.S0() ) ss2 = T2.S0();
      else if ( ss2 > T2.S1() ) ss2 = T2.S1();
    }
    return converged;
  }

  // - - - - - - - - - - - - - - - - - - - - - - - - - - - - - - - - - - - - - -

  void
  ClothoidCurve::intersect_ISO(
    real_type             offs,
    ClothoidCurve const & C,
    real_type             offs_C,
    IntersectList       & ilist,
    bool                  swap_s_vals
  ) const {
    if ( intersect_with_AABBtree ) {
      this->build_AABBtree_ISO( offs );
      C.build_AABBtree_ISO( offs_C );
      AABBtree::VecPairPtrBBox iList;
      m_aabb_tree.intersect( C.m_aabb_tree, iList );
      AABBtree::VecPairPtrBBox::const_iterator ip;

      for ( ip = iList.begin(); ip != iList.end(); ++ip ) {
        size_t ipos1 = size_t(ip->first->Ipos());
        size_t ipos2 = size_t(ip->second->Ipos());

        Triangle2D const & T1 = m_aabb_tri[ipos1];
        Triangle2D const & T2 = C.m_aabb_tri[ipos2];

        real_type ss1, ss2;
        bool converged = aabb_intersect_ISO( T1, offs, &C, T2, offs_C, ss1, ss2 );

        if ( converged ) {
          if ( swap_s_vals ) swap( ss1, ss2 );
          ilist.push_back( Ipair( ss1, ss2 ) );
        }
      }
    } else {
      bbTriangles_ISO( offs, m_aabb_tri, Utils::m_pi/18, 1e100 );
      C.bbTriangles_ISO( offs_C, C.m_aabb_tri, Utils::m_pi/18, 1e100 );
      vector<Triangle2D>::const_iterator i1, i2;
      for ( i1 = m_aabb_tri.begin(); i1 != m_aabb_tri.end(); ++i1 ) {
        for ( i2 = C.m_aabb_tri.begin(); i2 != C.m_aabb_tri.end(); ++i2 ) {
          Triangle2D const & T1 = *i1;
          Triangle2D const & T2 = *i2;

          real_type ss1, ss2;
          bool converged = aabb_intersect_ISO( T1, offs, &C, T2, offs_C, ss1, ss2 );

          if ( converged ) {
            if ( swap_s_vals ) swap( ss1, ss2 );
            ilist.push_back( Ipair( ss1, ss2 ) );
          }
        }
      }
    }
  }

  /*\
   |        _                     _   ____       _       _
   |    ___| | ___  ___  ___  ___| |_|  _ \ ___ (_)_ __ | |_
   |   / __| |/ _ \/ __|/ _ \/ __| __| |_) / _ \| | '_ \| __|
   |  | (__| | (_) \__ \  __/\__ \ |_|  __/ (_) | | | | | |_
   |   \___|_|\___/|___/\___||___/\__|_|   \___/|_|_| |_|\__|
  \*/

  void
  ClothoidCurve::closestPoint_internal(
    real_type   s_begin,
    real_type   s_end,
    real_type   qx,
    real_type   qy,
    real_type   offs,
    real_type & x,
    real_type & y,
    real_type & s,
    real_type & dst
  ) const {
    #if 1
    // minimize using circle approximation
    s = (s_begin + s_end)/2;
    int_type nout = 0;
    int_type n_ok = 0;
    for ( int_type iter = 0; iter < m_max_iter; ++iter ) {
      // osculating circle
      m_CD.eval_ISO( s, offs, x, y );
      real_type th = m_CD.theta( s );
      real_type kk = m_CD.kappa( s );
      real_type sc = 1+kk*offs;
      real_type ds = projectPointOnCircle( x, y, th, kk/sc, qx, qy )/sc;

      s += ds;

      bool out = false;
      if      ( s <= s_begin ) { out = true; s = s_begin; }
      else if ( s >= s_end   ) { out = true; s = s_end; }

      if ( out ) {
        if ( ++nout > 3 ) break;
      } else {
        // force one more itaration to improve accuracy
        if ( abs(ds) <= m_tolerance && ++n_ok >= 2 ) break;
      }
    }
    dst = hypot( qx-x, qy-y );
    #else
    real_type ds = (s_end-s_begin)/10;
    for ( int_type iter = 0; iter <= 10; ++iter ) {
      real_type ss = s_begin + iter * ds;
      real_type xx, yy;
      CD.eval( ss, offs, xx, yy );
      real_type dx = xx-qx;
      real_type dy = yy-qy;
      real_type dst1 = hypot( dx, dy );
      if ( dst1 < dst ) {
        s   = ss;
        x   = xx;
        y   = yy;
        dst = dst1;
      }
    }
    #endif
  }

  // - - - - - - - - - - - - - - - - - - - - - - - - - - - - - - - - - - - - - -

  void
  ClothoidCurve::closestPoint_internal(
    real_type   qx,
    real_type   qy,
    real_type   offs,
    real_type & x,
    real_type & y,
    real_type & s,
    real_type & DST
  ) const {
    DST = numeric_limits<real_type>::infinity();
    this->build_AABBtree_ISO( offs );

    AABBtree::VecPtrBBox candidateList;
    m_aabb_tree.min_distance( qx, qy, candidateList );
    AABBtree::VecPtrBBox::const_iterator ic;
    UTILS_ASSERT0(
      candidateList.size() > 0,
      "ClothoidCurve::closestPoint no candidate\n"
    );
    for ( ic = candidateList.begin(); ic != candidateList.end(); ++ic ) {
      size_t ipos = size_t((*ic)->Ipos());
      Triangle2D const & T = m_aabb_tri[ipos];
      real_type dst = T.distMin( qx, qy );
      if ( dst < DST ) {
        // refine distance
        real_type xx, yy, ss;
        closestPoint_internal(
          T.S0(), T.S1(), qx, qy, offs, xx, yy, ss, dst
        );
        if ( dst < DST ) {
          DST = dst;
          s   = ss;
          x   = xx;
          y   = yy;
        }
      }
    }
  }

  // - - - - - - - - - - - - - - - - - - - - - - - - - - - - - - - - - - - - - -

  int_type
  ClothoidCurve::closestPoint_ISO(
    real_type   qx,
    real_type   qy,
    real_type   offs,
    real_type & x,
    real_type & y,
    real_type & s,
    real_type & t,
    real_type & DST
  ) const {

    this->closestPoint_internal( qx, qy, offs, x, y, s, DST );

    // check if projection is orthogonal
    real_type nx, ny;
    nor_ISO( s, nx, ny );
    real_type qxx = qx - x;
    real_type qyy = qy - y;
    t = qxx * nx + qyy * ny - offs; // signed distance
    real_type pt = abs(qxx * ny - qyy * nx);
    G2LIB_DEBUG_MESSAGE(
      "Clothoid::closestPoint_ISO\n"
      "||P-P0|| = {} and {}, |(P-P0).T| = {}\n",
      DST, hypot(qxx,qyy), pt
    );
    return pt > GLIB2_TOL_ANGLE*hypot(qxx,qyy) ? -1 : 1;
  }

  // - - - - - - - - - - - - - - - - - - - - - - - - - - - - - - - - - - - - - -

  real_type
  ClothoidCurve::thetaTotalVariation() const {
    // cerco punto minimo parabola
    // root = -k/dk;
    real_type kL  = m_CD.kappa0;
    real_type kR  = m_CD.kappa(m_L);
    real_type thL = 0;
    real_type thR = m_CD.deltaTheta(m_L);
    if ( kL*kR < 0 ) {
      real_type root = -m_CD.kappa0/m_CD.dk;
      if ( root > 0 && root < m_L ) {
        real_type thM  = m_CD.deltaTheta(root);
        return abs( thR - thM ) + abs( thM - thL );
      }
    }
    return abs( thR - thL );
  }

  // - - - - - - - - - - - - - - - - - - - - - - - - - - - - - - - - - - - - - -

  real_type
  ClothoidCurve::thetaMinMax( real_type & thMin, real_type & thMax ) const {
    // cerco punto minimo parabola
    // root = -k/dk;
    real_type kL  = m_CD.kappa0;
    real_type kR  = m_CD.kappa(m_L);
    real_type thL = 0;
    real_type thR = m_CD.deltaTheta(m_L);
    if ( thL < thR ) { thMin = thL; thMax = thR; }
    else             { thMin = thR; thMax = thL; }
    if ( kL*kR < 0 ) {
      real_type root = -m_CD.kappa0/m_CD.dk;
      if ( root > 0 && root < m_L ) {
        real_type thM = m_CD.deltaTheta(root);
        if      ( thM < thMin ) thMin = thM;
        else if ( thM > thMax ) thMax = thM;
      }
    }
    return thMax - thMin;
  }

  // - - - - - - - - - - - - - - - - - - - - - - - - - - - - - - - - - - - - - -

  real_type
  ClothoidCurve::curvatureMinMax( real_type & kMin, real_type & kMax ) const {
    // cerco punto minimo parabola
    // root = -k/dk;
    kMin = m_CD.kappa0;
    kMax = m_CD.kappa(m_L);
    if ( kMax < kMin ) swap( kMax, kMin );
    return kMax - kMin;
  }

  // - - - - - - - - - - - - - - - - - - - - - - - - - - - - - - - - - - - - - -

  real_type
  ClothoidCurve::curvatureTotalVariation() const {
    // cerco punto minimo parabola
    // root = -k/dk;
    real_type km = m_CD.kappa0;
    real_type kp = m_CD.kappa(m_L);
    return abs(kp-km);
  }

  // - - - - - - - - - - - - - - - - - - - - - - - - - - - - - - - - - - - - - -

  real_type
  ClothoidCurve::integralCurvature2() const {
    return m_L*( m_CD.kappa0*(m_CD.kappa0+m_L*m_CD.dk) + (m_L*m_L)*m_CD.dk*m_CD.dk/3 );
  }

  // - - - - - - - - - - - - - - - - - - - - - - - - - - - - - - - - - - - - - -

  real_type
  ClothoidCurve::integralJerk2() const {
    real_type k2 = m_CD.kappa0*m_CD.kappa0;
    real_type k3 = m_CD.kappa0*k2;
    real_type k4 = k2*k2;
    real_type t1 = m_L;
    real_type t2 = m_L*t1;
    real_type t3 = m_L*t2;
    real_type t4 = m_L*t3;
    return ((((t4/5*m_CD.dk+t3*m_CD.kappa0)*m_CD.dk+(1+2*t2)*k2)*m_CD.dk+2*t1*k3)*m_CD.dk+k4)*m_L;
  }

  // - - - - - - - - - - - - - - - - - - - - - - - - - - - - - - - - - - - - - -

  real_type
  ClothoidCurve::integralSnap2() const {
    real_type k2  = m_CD.kappa0*m_CD.kappa0;
    real_type k3  = m_CD.kappa0*k2;
    real_type k4  = k2*k2;
    real_type k5  = k4*m_CD.kappa0;
    real_type k6  = k4*k2;
    real_type dk2 = m_CD.dk*m_CD.dk;
    real_type dk3 = m_CD.dk*dk2;
    real_type dk4 = dk2*dk2;
    real_type dk5 = dk4*m_CD.dk;
    real_type dk6 = dk4*dk2;
    real_type t2  = m_L;
    real_type t3  = m_L*t2;
    real_type t4  = m_L*t3;
    real_type t5  = m_L*t4;
    real_type t6  = m_L*t5;
    real_type t7  = m_L*t6;

    return ( (t7/7)*dk6 + dk5*m_CD.kappa0*t6 + 3*dk4*k2*t5 + 5*dk3*k3*t4 +
             5*dk2*k4*t3 + 3*dk3*t3 + 3*m_CD.dk*k5*t2 + 9*dk2*m_CD.kappa0*t2 +
             k6+9*k2*m_CD.dk ) * m_L;
  }

  // - - - - - - - - - - - - - - - - - - - - - - - - - - - - - - - - - - - - - -

  ostream_type &
  operator << ( ostream_type & stream, ClothoidCurve const & c ) {
    fmt::print( stream,
      "x0     = {:<12} x1     = {:<12}\n"
      "y0     = {:<12} y1     = {:<12}\n"
      "theta0 = {:<12} theta1 = {:<12}\n"
      "kappa0 = {:<12} kappa1 = {:<12}\n"
      "dk     = {:<12} L      = {:<12}\n",
      fmt::format("{:.6}",c.xBegin()),
      fmt::format("{:.6}",c.xEnd()),
      fmt::format("{:.6}",c.yBegin()),
      fmt::format("{:.6}",c.yEnd()),
      fmt::format("{:.6}",c.thetaBegin()),
      fmt::format("{:.6}",c.thetaEnd()),
      fmt::format("{:.6}",c.kappaBegin()),
      fmt::format("{:.6}",c.kappaEnd()),
      fmt::format("{:.6}",c.m_CD.dk),
      fmt::format("{:.6}",c.m_L)
    );
    return stream;
  }

}

// EOF: Clothoid.cc
```

### Quick search

### Table of Contents

- Matlab Interface Manual
- C++ API
- MATLAB API

«
hide menu

menu
sidebar
»

### Navigation

- index
- toc
- Clothoids »
- Program Listing for File Clothoid.cc

© Copyright 2021, Enrico Bertolazzi and Marco Frego.
Created using Sphinx 4.2.0.
