# Supplementary material for: Smooth Interpolating Curves with Local Control and Monotone Alternating Curvature
Source: Comput Graph Forum. 2022 Oct 6;41(5):25–38. doi: 10.1111/cgf.14600 (PMC9827861; doi:10.1111/cgf.14600)
Supplement: Supplementary file 1 — Supplement Material [file CGF-41-25-s001.zip › Local-Smooth-Interpolating-MonoCurvature/extern/clothoids/docs/api-cpp/program_listing_file_ClothoidAsyPlot.cc.html]

Program Listing for File ClothoidAsyPlot.cc — Clothoids v2.0.9

### Navigation

- index
- toc
- Clothoids »
- Program Listing for File ClothoidAsyPlot.cc

# Program Listing for File ClothoidAsyPlot.cc¶

↰ Return to documentation for file (`ClothoidAsyPlot.cc`)

```
#include "Clothoids.hh"

namespace G2lib {

  AsyPlot::AsyPlot( string _filename, bool _showAxes )
  : filename(_filename)
  , showAxes(_showAxes)
  {
    UTILS_ASSERT( openFile(), "Failed to open file {}\n", filename );
    initFile();
  }

  AsyPlot::~AsyPlot() {
    if ( showAxes ) displayAxes();
    if ( closeFile() ) compileFile();
  }

  void
  AsyPlot::compileFile() {
    string cmdComp = "asy -f pdf "+ filename;
    system(cmdComp.c_str());
    string pdfFile = filename.substr(0,filename.find(".asy")) + ".pdf";
    std::cout << pdfFile << std::endl;
    string cmdOpen = "(okular " + pdfFile + " &> /dev/null )&";
    system(cmdOpen.c_str());
  }

  void
  AsyPlot::initFile() {
    file
      << "// File generated automatically from C++ \n\n\n"
      << "import graph;\n"
      << "include \"clothoidLib.asylib\";\n"
      << "size(14cm,7cm);\n"
      << "\n\n\n";
  }

  void
  AsyPlot::drawClothoid( ClothoidCurve const & c,
                         string const & penna,
                         real_type offset ) const {
    if (offset == 0.) {
      file
        << "path pclot = clothoidPoints(("
        << c.xBegin()      << ','
        << c.yBegin()      << "),"
        << c.thetaBegin()  << ','
        << c.kappaBegin()  << ','
        << c.dkappa()      << ','
        << c.length()      << ','
        << "100,0);\n"
        << "pen penna = " << penna << ";\n"
        << "draw(pclot, penna);\n\n";
      } else {
      file
        << "path pclot = clothoidOffset(("
            << c.xBegin()     << ','
        << c.yBegin()     << "),"
        << c.thetaBegin() << ','
          << c.kappaBegin() << ','
        << c.dkappa()     << ','
        << c.length()     << ','
        << "100,"
        << offset
        << "); \n"
        << "pen penna = " << penna << ";\n"
        << "draw(pclot, penna);\n\n";
    }
  }

  void
  AsyPlot::dot( real_type x, real_type y, string const & penna ) const {
    file << "dot((" << x << "," << y << ")," << penna << ");\n\n";
  }

  void
  AsyPlot::triangle( Triangle2D const & t, string const & penna ) const {
    file
      << "draw((" << t.x1() << "," << t.y1() << ") -- "
      << '(' << t.x2() << ',' << t.y2() << ") -- "
      << '(' << t.x3() << ',' << t.y3() << ") -- cycle, "
      << penna << ");\n\n";
  }

  void
  AsyPlot::drawRect( real_type x0, real_type y0,
                     real_type x1, real_type y1,
                     real_type x2, real_type y2,
                     real_type x3, real_type y3,
                     string const & penna ) const {
    file
    << "fill((" << x0 << "," << y0 << ") -- "
    << '(' << x1 << ',' << y1 << ") -- "
    << '(' << x2 << ',' << y2 << ") -- "
    << '(' << x3 << ',' << y3 << ") -- cycle, "
    << penna << ");\n\n";
  }

  void
  AsyPlot::displayAxes() const {
    file
      << "xaxis(\"$x$\", black+fontsize(7pt),xmin=0,xmax=5,Ticks(Step=2,step=1,NoZero,Size=.8mm, size=.4mm));\n"
      << "yaxis(\"$y$\", black+fontsize(7pt),ymin=0,ymax=5,Ticks(Step=2,step=1,NoZero,Size=.8mm, size=.4mm));\n";
  }

  void
  AsyPlot::displayAxes( string const & labX,
                        string const & labY,
                        real_type xmin,
                        real_type xmax,
                        real_type ymin,
                        real_type ymax ) const {
      file
      << "xaxis(\"" << labX << "\", black+fontsize(7pt),xmin="
      << xmin << ",xmax=" << xmax
      << ",Ticks(Step=2,step=1,NoZero,Size=.8mm, size=.4mm));\n"
      << "yaxis(\"" << labY << "\", black+fontsize(7pt),ymin="
      << ymin << ",ymax=" << ymax
      << ",Ticks(Step=2,step=1,NoZero,Size=.8mm, size=.4mm));\n";
  }

  void
  AsyPlot::drawLine( real_type x0, real_type y0,
                     real_type x1, real_type y1,
                     string const & penna ) const {
    file
      << "draw((" << x0 << "," << y0 << ") -- "
      << "(" << x1 << "," << y1 << "), " << penna << ");\n\n";
  }

  void
  AsyPlot::label( string const & text,
                  real_type      x,
                  real_type      y,
                  string const & placement,
                  string const & penna ) const {
    file
      << "label(\"" << text << "\", (" << x << ", " << y << "), "
      << (!placement.empty() ? placement + ", " : "")
        << penna << ");\n\n";
  }

  bool
  AsyPlot::openFile() {
    file.open(filename.c_str());
    return file.is_open();
  }

  bool
  AsyPlot::closeFile() {
    file.close();
    return !file.is_open();
  }

}
```

### Quick search

### Table of Contents

- Matlab Interface Manual
- C++ API
- MATLAB API

«
hide menu

menu
sidebar
»

### Navigation

- index
- toc
- Clothoids »
- Program Listing for File ClothoidAsyPlot.cc

© Copyright 2021, Enrico Bertolazzi and Marco Frego.
Created using Sphinx 4.2.0.
