# Supplementary material for: Smooth Interpolating Curves with Local Control and Monotone Alternating Curvature
Source: Comput Graph Forum. 2022 Oct 6;41(5):25–38. doi: 10.1111/cgf.14600 (PMC9827861; doi:10.1111/cgf.14600)
Supplement: Supplementary file 1 — Supplement Material [file CGF-41-25-s001.zip › Local-Smooth-Interpolating-MonoCurvature/extern/clothoids/docs/api-cpp/program_listing_file_ClothoidDistance.cc.html]

Program Listing for File ClothoidDistance.cc — Clothoids v2.0.9

### Navigation

- index
- toc
- Clothoids »
- Program Listing for File ClothoidDistance.cc

# Program Listing for File ClothoidDistance.cc¶

↰ Return to documentation for file (`ClothoidDistance.cc`)

```
/*--------------------------------------------------------------------------*\
 |                                                                          |
 |  Copyright (C) 2017                                                      |
 |                                                                          |
 |         , __                 , __                                        |
 |        /|/  \               /|/  \                                       |
 |         | __/ _   ,_         | __/ _   ,_                                |
 |         |   \|/  /  |  |   | |   \|/  /  |  |   |                        |
 |         |(__/|__/   |_/ \_/|/|(__/|__/   |_/ \_/|/                       |
 |                           /|                   /|                        |
 |                           \|                   \|                        |
 |                                                                          |
 |      Enrico Bertolazzi                                                   |
 |      Dipartimento di Ingegneria Industriale                              |
 |      Universita` degli Studi di Trento                                   |
 |      email: enrico.bertolazzi@unitn.it                                   |
 |                                                                          |
\*--------------------------------------------------------------------------*/

#include "Clothoids.hh"

// Workaround for Visual Studio
#ifdef min
  #undef min
#endif

#ifdef max
  #undef max
#endif

namespace G2lib {

  using std::abs;
  using std::sqrt;
  using std::sin;
  using std::cos;
  using std::max;
  using std::min;

  // - - - - - - - - - - - - - - - - - - - - - - - - - - - - - - - - - - - - - -

  real_type
  ClothoidCurve::closestPointBySample(
    real_type   ds,
    real_type   qx,
    real_type   qy,
    real_type & X,
    real_type & Y,
    real_type & S
  ) const {
    S = 0;
    X = m_CD.x0;
    Y = m_CD.y0;
    real_type DST = hypot( X-qx, Y-qy );
    real_type SSS = ds;
    while ( SSS <= m_L ) {
      real_type theta, kappa, XS, YS;
      m_CD.evaluate( SSS, theta, kappa, XS, YS );
      real_type dst = hypot( XS-qx, YS-qy );
      if ( dst < DST ) {
        DST = dst;
        S   = SSS;
        X   = XS;
        Y   = YS;
      }
      SSS += ds;
    }
    return DST;
  }

  // - - - - - - - - - - - - - - - - - - - - - - - - - - - - - - - - - - - - - -

  #ifndef DOXYGEN_SHOULD_SKIP_THIS
  static
  bool
  closestPointQC2(
    real_type            epsi,
    ClothoidData const & CD,
    real_type            L,
    real_type            qx,
    real_type            qy,
    real_type          & S
  ) {

    // S = GUESS
    int nb = 0;
    real_type theta, kappa, dS, dx, dy;
    real_type s = S;
    for ( int iter = 0; iter < 20 && nb < 2; ++iter ) {
      CD.evaluate( s, theta, kappa, dx, dy ); dx -= qx; dy -= qy;

      real_type Cs  = cos(theta);
      real_type Ss  = sin(theta);
      real_type a0  = Cs * dy - Ss * dx;
      real_type b0  = Ss * dy + Cs * dx;
      real_type tmp = a0*kappa;

      // approx clothoid with a circle
      if ( 1+2*tmp > 0 ) {

        tmp = b0/(1+tmp);
        dS = -tmp*Atanc(tmp*kappa);

      } else {

        real_type om = atan2( b0, a0+1/kappa );
        if ( kappa < 0 ) {
          if ( om < 0 ) om += Utils::m_pi;
          else          om -= Utils::m_pi;
        }

        dS = -om/kappa;
      }

      s += dS;
      if ( abs( dS ) < epsi ) {
        if ( s < -epsi || s > L+epsi ) return false;
        S = s;
        return true;
      }

      // check divergence
      if      ( s < 0 ) { s = 0; ++nb; }
      else if ( s > L ) { s = L; ++nb; }
      else              { nb = 0; }

    }
    return false;
  }
  #endif

  // - - - - - - - - - - - - - - - - - - - - - - - - - - - - - - - - - - - - - -

  #ifndef DOXYGEN_SHOULD_SKIP_THIS
  static
  real_type
  closestPointQC1(
    real_type            epsi,
    ClothoidData const & CD,
    real_type            L,
    real_type            qx,
    real_type            qy,
    real_type          & X,
    real_type          & Y,
    real_type          & S
  ) {

    real_type phi0 = CD.theta0 - atan2( CD.y0 - qy, CD.x0 - qx );
    bool ok0 = cos(phi0) < 0; // distanza decrescente

    real_type theta1, kappa1, x1, y1;
    CD.evaluate( L, theta1, kappa1, x1, y1 );
    real_type phi1 = theta1 - atan2( y1 - qy, x1 - qx );
    bool ok1 = cos(phi1) > 0; // distanza crescente

    real_type s0 = 0, x0 = CD.x0, y0 = CD.y0;
    if ( ok0 ) ok0 = closestPointQC2( epsi, CD, L, qx, qy, s0 );
    if ( ok0 ) CD.eval( s0, x0, y0 );
    real_type d0 = hypot( x0-qx, y0-qy );

    real_type s1 = L;
    if ( ok1 ) ok1 = closestPointQC2( epsi, CD, L, qx, qy, s1 );
    if ( ok1 ) CD.eval( s1, x1, y1 );
    real_type d1 = hypot( x1-qx, y1-qy );

    if ( !ok0 && !ok1 ) { // s1 - s0 > 2 * epsi ) { // buoni entrambi estremi
      S = (s0+s1)/2;
      bool okm = closestPointQC2( epsi, CD, L, qx, qy, S );
      if ( okm ) {
        CD.eval( S, X, Y );
        real_type dm = hypot( X-qx, Y-qy );
        if ( dm < d0 && dm < d1 ) return dm;
      }
    }

    if ( d0 < d1 ) { S = s0; X = x0; Y = y0; return d0; }
    else           { S = s1; X = x1; Y = y1; return d1; }

  }
  #endif

  // - - - - - - - - - - - - - - - - - - - - - - - - - - - - - - - - - - - - - -

  #ifndef DOXYGEN_SHOULD_SKIP_THIS
  static
  real_type
  closestPointQC(
    real_type            epsi,
    ClothoidData const & CD,
    real_type            L,
    real_type            qx,
    real_type            qy,
    real_type          & X,
    real_type          & Y,
    real_type          & S
  ) {

    real_type DTheta = abs( CD.theta(L) - CD.theta0 );
    if ( DTheta <= Utils::m_2pi )
      return closestPointQC1( epsi, CD, L, qx, qy, X, Y, S );

    real_type cx = CD.c0x();
    real_type cy = CD.c0y();

    //if ( hypot( CD.x0 - cx, CD.y0 - cy ) <= hypot( qx - cx, qy - cy ) ) {
    if ( 1 <= abs(CD.kappa0) * hypot( qx - cx, qy - cy ) ) {
      real_type ell = CD.aplus(Utils::m_2pi);
      return closestPointQC1( epsi, CD, ell, qx, qy, X, Y, S );
    }

    ClothoidData CD1;
    CD.reverse( L, CD1 );
    cx = CD1.c0x();
    cy = CD1.c0y();

    //if ( hypot( CD1.x0 - cx, CD1.y0 - cy ) >= hypot( qx - cx, qy - cy ) ) {
    if ( 1 >= abs(CD1.kappa0) * hypot( qx - cx, qy - cy ) ) {
      real_type ell = CD1.aplus(Utils::m_2pi);
      real_type d   = closestPointQC1( epsi, CD1, ell, qx, qy, X, Y, S );
      S = L - S;
      return d;
    }

    real_type ell = CD.aplus(DTheta/2);
    real_type d0  = closestPointQC( epsi, CD, ell, qx, qy, X, Y, S );

    CD.eval( ell, CD1 );

    real_type X1, Y1, S1;
    real_type d1 = closestPointQC( epsi, CD1, L-ell, qx, qy, X1, Y1, S1 );

    if ( d1 < d0 ) { S = ell+S1; X = X1; Y = Y1; return d1; }

    return d0;

  }
  #endif

  // - - - - - - - - - - - - - - - - - - - - - - - - - - - - - - - - - - - - - -

  #ifndef DOXYGEN_SHOULD_SKIP_THIS
  static
  bool
  closestPointStandard3(
    real_type   epsi,
    real_type   a,
    real_type   b,
    real_type   qx,
    real_type   qy,
    real_type & S
  ) {
    // S = GUESS
    int nb = 0;
    real_type s = S, dS, dx, dy;
    for ( int iter = 0; iter < 20 && nb < 2; ++iter ) {
      // approx clothoid with a circle
      real_type kappa = Utils::m_pi * s;
      real_type theta = 0.5*(kappa*s);
      FresnelCS( s, dx, dy ); dx -= qx; dy -= qy;

      real_type Cs  = cos(theta);
      real_type Ss  = sin(theta);
      real_type a0  = Cs * dy - Ss * dx;
      real_type b0  = Ss * dy + Cs * dx;
      real_type tmp = a0*kappa;

      if ( 1+2*tmp > 0 ) {

        tmp = b0/(1+tmp);
        dS = -tmp*Atanc(tmp*kappa);

      } else {

        real_type om = atan2( b0, a0+1/kappa );
        if ( kappa < 0 ) {
          if ( om < 0 ) om += Utils::m_pi;
          else          om -= Utils::m_pi;
        }

        dS = -om/kappa;
      }

      s += dS;
      if ( abs( dS ) < epsi ) {
        if ( s < a-epsi|| s > b+epsi ) break;
        S = s;
        return true;
      }

      // check divergence
      if      ( s < a ) { s = a; ++nb; }
      else if ( s > b ) { s = b; ++nb; }
      else              { nb = 0; }

    }
    return false;
  }
  #endif

  // - - - - - - - - - - - - - - - - - - - - - - - - - - - - - - - - - - - - - -

  #ifndef DOXYGEN_SHOULD_SKIP_THIS
  static
  real_type
  closestPointStandard2(
    real_type   epsi,
    real_type   a,
    real_type   b,
    real_type   qx,
    real_type   qy,
    real_type & S
  ) {

    real_type dx, dy;
    FresnelCS( a, dx, dy ); dx -= qx; dy -= qy;
    real_type phia = Utils::m_pi_2 * (a*a) - atan2( dy, dx );
    bool ok0 = cos(phia) < 0; // distanza decrescente

    FresnelCS( b, dx, dy ); dx -= qx; dy -= qy;
    real_type phib = Utils::m_pi_2 * (b*b) - atan2( dy, dx );
    bool ok1 = cos(phib) > 0; // distanza crescente

    real_type s0 = a;
    if ( ok0 ) ok0 = closestPointStandard3( epsi, a, b, qx, qy, s0 );
    FresnelCS( s0, dx, dy ); dx -= qx; dy -= qy;
    real_type d0 = hypot( dx, dy );

    real_type s1 = b;
    if ( ok1 ) ok1 = closestPointStandard3( epsi, a, b, qx, qy, s1 );
    FresnelCS( s1, dx, dy ); dx -= qx; dy -= qy;
    real_type d1 = hypot( dx, dy );

    if ( !ok0 && !ok1 ) {  // s1 - s0 > 2 * epsi ) { // buoni entrambi estremi
      S = (s0+s1)/2;
      bool ok = closestPointStandard3( epsi, a, b, qx, qy, S );
      if ( ok ) {
        FresnelCS( S, dx, dy ); dx -= qx; dy -= qy;
        real_type dm = hypot( dx, dy );
        if ( dm < d0 && dm < d1 ) return dm;
      }
    }

    if ( d0 < d1 ) { S = s0; return d0; }
    else           { S = s1; return d1; }

  }
  #endif

  // - - - - - - - - - - - - - - - - - - - - - - - - - - - - - - - - - - - - - -

  #ifndef DOXYGEN_SHOULD_SKIP_THIS
  static
  real_type
  closestPointStandard(
    real_type            epsi,
    ClothoidData const & CD,
    real_type            L,
    real_type            qx,
    real_type            qy,
    real_type          & S
  ) {

    // transform to standard clothoid
    real_type sflex  = -CD.kappa0/CD.dk;

    UTILS_ASSERT( sflex <= 0, " bad sflex = {}\n", sflex );

    real_type thflex = CD.theta0 + 0.5*CD.kappa0*sflex;
    real_type ssf    = sin(thflex);
    real_type csf    = cos(thflex);
    real_type gamma  = sqrt(abs(CD.dk)/Utils::m_pi);
    real_type a      = -sflex*gamma;
    real_type b      = (L-sflex)*gamma;
    real_type xflex, yflex;
    CD.eval( sflex, xflex, yflex );
    real_type xx = qx - xflex;
    real_type yy = qy - yflex;
    // gamma * R^(-1)
    real_type qqx = gamma * (  csf * xx + ssf * yy );
    real_type qqy = gamma * ( -ssf * xx + csf * yy );
    // M^(-1)
    if ( CD.dk < 0 ) qqy = -qqy;

    // now in standard form
    if ( b*b-a*a <= 4 ) {
      real_type d = closestPointStandard2( epsi, a, b, qqx, qqy, S );
      S = sflex + S/gamma;
      return d/gamma;
    }

    FresnelCS( a, xx, yy );
    real_type di = hypot(qqx-0.5,qqy-0.5);
    real_type da = hypot(xx-0.5,yy-0.5);

    if ( di >= da ) {
      real_type La = 4/(a+sqrt(a*a+4));
      real_type d  = closestPointStandard2( epsi, a, a+La, qqx, qqy, S );
      S = sflex + S/gamma;
      return d/gamma;
    }

    FresnelCS( b, xx, yy );
    real_type db = hypot(xx-0.5,yy-0.5);

    if ( di <= db ) {
      real_type Lb = 4/(b+sqrt(b*b-4));
      real_type d  = closestPointStandard2( epsi, b-Lb, b, qqx, qqy, S );
      S = sflex + S/gamma;
      return d/gamma;
    }

    real_type ss = a;
    bool converged = false;
    for ( int iter = 0; iter < 20 && !converged; ++iter ) {
      FresnelCS( ss, xx, yy );
      real_type kappa = Utils::m_pi * ss;
      real_type theta = Utils::m_pi_2 * (ss*ss);
      real_type rhox  = xx - 0.5;
      real_type rhoy  = yy - 0.5;
      real_type rho   = hypot( rhox, rhoy );
      real_type f     = rho - di;
      //if ( abs(f) < epsi ) break;
      real_type tphi  = theta - atan2( rhoy, rhox );
      real_type df    = cos( tphi );
      real_type t     = sin( tphi );
      real_type ddf   = t*(kappa-t/rho);
      real_type ds    = (f*df)/((df*df)-f*ddf/2);
      ss -= ds;
      converged = abs(ds) < epsi;
    }

    UTILS_ASSERT0( converged, "closestPointStandard not converged\n" );

    real_type Lp = min( b-ss, 4/(ss+sqrt(ss*ss+4)) );
    real_type Lm = min( ss-a, 4/(ss+sqrt(ss*ss-4)) );

    real_type sp, sm;
    real_type dp = closestPointStandard2( epsi, ss, ss+Lp, qqx, qqy, sp );
    real_type dm = closestPointStandard2( epsi, ss-Lm, ss, qqx, qqy, sm );

    if ( dp < dm ) { S = sflex + sp/gamma; return dp/gamma; }
    else           { S = sflex + sm/gamma; return dm/gamma; }
  }
  #endif

  // - - - - - - - - - - - - - - - - - - - - - - - - - - - - - - - - - - - - - -

  #ifndef DOXYGEN_SHOULD_SKIP_THIS
  static
  real_type
  closestPoint1(
    real_type            epsi,
    ClothoidData const & CD,
    real_type            L,
    real_type            qx,
    real_type            qy,
    real_type          & X,
    real_type          & Y,
    real_type          & S
  ) {

    real_type NT = 4; // number of turn of the clothid after wich is considered quasi-circular
    real_type DK = sqrt(NT*Utils::m_2pi*abs(CD.dk));
    if ( abs(CD.kappa0) >= DK ) {
      return closestPointQC( epsi, CD, L, qx, qy, X, Y, S );
    }

    if ( abs(CD.kappa0)+abs(CD.dk)*L <= DK ) {
      real_type d = closestPointStandard( epsi, CD, L, qx, qy, S );
      CD.eval( S, X, Y );
      return d;
    }

    real_type ell = (DK-abs(CD.kappa0))/abs(CD.dk);

    UTILS_ASSERT( ell > 0 && ell < L, "bad ell = {} L = {}\n", ell, L );

    ClothoidData CDS;
    CD.eval( ell, CDS );

    real_type S0;
    real_type d0 = closestPointStandard( epsi, CD, ell, qx, qy, S0 );
    real_type d1 = closestPointQC( epsi, CDS, L-ell, qx, qy, X, Y, S );
    if ( d0 < d1 ) {
      S = S0;
      CD.eval( S, X, Y );
      return d0;
    } else {
      S += ell;
      return d1;
    }
  }
  #endif

  // - - - - - - - - - - - - - - - - - - - - - - - - - - - - - - - - - - - - - -

  int_type
  ClothoidCurve::closestPoint_ISO(
    real_type   qx,
    real_type   qy,
    real_type & X,
    real_type & Y,
    real_type & S,
    real_type & T,
    real_type & dst
  ) const {

    real_type epsi = 1e-10;

    // check if flex is inside curve, if so then split

    if ( m_CD.kappa0*m_CD.dk >= 0 ) { // flex on the left
      dst = closestPoint1( epsi, m_CD, m_L, qx, qy, X, Y, S );
    } else if ( m_CD.dk*m_CD.kappa(m_L) <= 0 ) { // flex on the right, reverse curve
      ClothoidData CD1;
      m_CD.reverse( m_L, CD1 );
      dst = closestPoint1( epsi, CD1, m_L, qx, qy, X, Y, S );
      S   = m_L-S;
    } else {
      // flex inside, split clothoid
      ClothoidData C0, C1;
      real_type sflex = m_CD.split_at_flex( C0, C1 );

      real_type d0 = closestPoint1( epsi, C0, m_L-sflex, qx, qy, X, Y, S  );
      real_type x1, y1, s1;
      real_type d1 = closestPoint1( epsi, C1, sflex, qx, qy, x1, y1, s1 );

      if ( d1 < d0 ) {
        S   = sflex - s1;
        X   = x1;
        Y   = y1;
        dst = d1;
      } else {
        S  += sflex;
        dst = d0;
      }
    }
    // check if projection is orthogonal
    real_type nx, ny;
    nor_ISO( S, nx, ny );
    real_type qxx = qx - X;
    real_type qyy = qy - Y;
    T = qxx * nx + qyy * ny; // signed distance
    real_type pt = abs(qxx * ny - qyy * nx);
    G2LIB_DEBUG_MESSAGE(
      "ClothoidCurve::closestPoint_ISO: ||P-P0|| = {}, |(P-P0).T| = {}\n",
      dst, pt
    );
    return pt > GLIB2_TOL_ANGLE*dst ? -1 : 1;
  }

/*
+      // approx clothoid with a circle
       real_type theta, kappa;
       eval( S, theta, kappa, X, Y );
-      real_type dx   = X-x;
-      real_type dy   = Y-y;
-      real_type d    = hypot( dx, dy );
-      real_type tphi = theta - atan2( dy, dx );
-      real_type f    = d*cos(tphi);
-      real_type g    = d*sin(tphi);
-      real_type df   = 1-kappa*g;
-      real_type ddf  = -kappa*f*(dk+kappa);
-
*/

}

// EOF: ClothoidDistance.cc
```

### Quick search

### Table of Contents

- Matlab Interface Manual
- C++ API
- MATLAB API

«
hide menu

menu
sidebar
»

### Navigation

- index
- toc
- Clothoids »
- Program Listing for File ClothoidDistance.cc

© Copyright 2021, Enrico Bertolazzi and Marco Frego.
Created using Sphinx 4.2.0.
