# Supplementary material for: Smooth Interpolating Curves with Local Control and Monotone Alternating Curvature
Source: Comput Graph Forum. 2022 Oct 6;41(5):25–38. doi: 10.1111/cgf.14600 (PMC9827861; doi:10.1111/cgf.14600)
Supplement: Supplementary file 1 — Supplement Material [file CGF-41-25-s001.zip › Local-Smooth-Interpolating-MonoCurvature/extern/clothoids/docs/api-cpp/program_listing_file_ClothoidG2.cc.html]

Program Listing for File ClothoidG2.cc — Clothoids v2.0.9

### Navigation

- index
- toc
- Clothoids »
- Program Listing for File ClothoidG2.cc

# Program Listing for File ClothoidG2.cc¶

↰ Return to documentation for file (`ClothoidG2.cc`)

```
/*--------------------------------------------------------------------------*\
 |                                                                          |
 |  Copyright (C) 2017                                                      |
 |                                                                          |
 |         , __                 , __                                        |
 |        /|/  \               /|/  \                                       |
 |         | __/ _   ,_         | __/ _   ,_                                |
 |         |   \|/  /  |  |   | |   \|/  /  |  |   |                        |
 |         |(__/|__/   |_/ \_/|/|(__/|__/   |_/ \_/|/                       |
 |                           /|                   /|                        |
 |                           \|                   \|                        |
 |                                                                          |
 |      Enrico Bertolazzi                                                   |
 |      Dipartimento di Ingegneria Industriale                              |
 |      Universita` degli Studi di Trento                                   |
 |      email: enrico.bertolazzi@unitn.it                                   |
 |                                                                          |
\*--------------------------------------------------------------------------*/

#include "Clothoids.hh"

#include <cfloat>

#ifdef __GNUC__
#pragma GCC diagnostic push
#pragma GCC diagnostic ignored "-Wsign-conversion"
#pragma GCC diagnostic ignored "-Wswitch-enum"
#endif
#ifdef __clang__
#pragma clang diagnostic push
#pragma clang diagnostic ignored "-Wsign-conversion"
#pragma clang diagnostic ignored "-Wswitch-enum"
#endif

namespace G2lib {

  #ifndef DOXYGEN_SHOULD_SKIP_THIS

  using std::abs;
  using std::fpclassify;
  using std::copy;
  using std::back_inserter;
  using std::fill;
  using std::vector;

  inline
  real_type
  power2( real_type a )
  { return a*a; }

  inline
  real_type
  power3( real_type a )
  { return a*a*a; }

  inline
  real_type
  power4( real_type a )
  { real_type a2 = a*a; return a2*a2; }

  #endif

  /*\
   |    ____ ____            _           ____
   |   / ___|___ \ ___  ___ | |_   _____|___ \ __ _ _ __ ___
   |  | |  _  __) / __|/ _ \| \ \ / / _ \ __) / _` | '__/ __|
   |  | |_| |/ __/\__ \ (_) | |\ V /  __// __/ (_| | | | (__
   |   \____|_____|___/\___/|_| \_/ \___|_____\__,_|_|  \___|
  \*/

  int
  G2solve2arc::build(
    real_type _x0,
    real_type _y0,
    real_type _theta0,
    real_type _kappa0,
    real_type _x1,
    real_type _y1,
    real_type _theta1,
    real_type _kappa1
  ) {

    x0     = _x0;
    y0     = _y0;
    theta0 = _theta0;
    kappa0 = _kappa0;
    x1     = _x1;
    y1     = _y1;
    theta1 = _theta1;
    kappa1 = _kappa1;

    // scale problem
    real_type dx = x1 - x0;
    real_type dy = y1 - y0;
    phi    = atan2( dy, dx );
    lambda = hypot( dx, dy );

    real_type C = dx/lambda;
    real_type S = dy/lambda;
    lambda /= 2;

    xbar = -(x0*C+y0*S+lambda);
    ybar = x0*S-y0*C;

    th0 = theta0 - phi;
    th1 = theta1 - phi;

    k0 = kappa0*lambda;
    k1 = kappa1*lambda;

    DeltaK     = k1  - k0;
    DeltaTheta = th1 - th0;

    return solve();
  }

  // - - - - - - - - - - - - - - - - - - - - - - - - - - - - - - - - - - - - - -

  void
  G2solve2arc::setTolerance( real_type tol ) {
    UTILS_ASSERT(
      tol > 0 && tol <= 0.1,
      "G2solve2arc::setTolerance, tolerance = {} must be in (0,0.1]\n", tol
    );
    tolerance = tol;
  }

  // - - - - - - - - - - - - - - - - - - - - - - - - - - - - - - - - - - - - - -

  void
  G2solve2arc::setMaxIter( int miter ) {
    UTILS_ASSERT(
      miter > 0 && miter <= 1000,
      "G2solve2arc::setMaxIter, maxIter = {} must be in [1,1000]\n", miter
    );
    maxIter = miter;
  }

  // - - - - - - - - - - - - - - - - - - - - - - - - - - - - - - - - - - - - - -

  void
  G2solve2arc::evalA(
    real_type   alpha,
    real_type   L,
    real_type & A
  ) const {
    real_type K  = k0+k1;
    real_type aK = alpha*DeltaK;
    A = alpha*(L*(aK-K)+2*DeltaTheta);
  }

  // - - - - - - - - - - - - - - - - - - - - - - - - - - - - - - - - - - - - - -

  void
  G2solve2arc::evalA(
    real_type   alpha,
    real_type   L,
    real_type & A,
    real_type & A_1,
    real_type & A_2
  ) const {
    real_type K  = k0+k1;
    real_type aK = alpha*DeltaK;
    A   = alpha*(L*(aK-K)+2*DeltaTheta);
    A_1 = (2*aK-K)*L+2*DeltaTheta;
    A_2 = alpha*(aK-K);
  }

  // - - - - - - - - - - - - - - - - - - - - - - - - - - - - - - - - - - - - - -

  void
  G2solve2arc::evalG(
    real_type alpha,
    real_type L,
    real_type th,
    real_type k,
    real_type G[2]
  ) const {
    real_type A, X, Y;
    evalA( alpha, L, A );
    real_type ak = alpha*k;
    GeneralizedFresnelCS( A, ak*L, th, X, Y );
    G[0] = alpha*X;
    G[1] = alpha*Y;
  }

  // - - - - - - - - - - - - - - - - - - - - - - - - - - - - - - - - - - - - - -

  void
  G2solve2arc::evalG(
    real_type alpha,
    real_type L,
    real_type th,
    real_type k,
    real_type G[2],
    real_type G_1[2],
    real_type G_2[2]
  ) const {

    real_type A, A_1, A_2, X[3], Y[3];
    evalA( alpha, L, A, A_1, A_2 );
    real_type ak = alpha*k;
    real_type Lk = L*k;
    GeneralizedFresnelCS( 3, A, ak*L, th, X, Y );

    G[0]   = alpha*X[0];
    G_1[0] = X[0]-alpha*(Y[2]*A_1/2+Y[1]*Lk);
    G_2[0] =     -alpha*(Y[2]*A_2/2+Y[1]*ak);

    G[1]   = alpha*Y[0];
    G_1[1] = Y[0]+alpha*(X[2]*A_1/2+X[1]*Lk);
    G_2[1] =      alpha*(X[2]*A_2/2+X[1]*ak);

  }

  // - - - - - - - - - - - - - - - - - - - - - - - - - - - - - - - - - - - - - -

  void
  G2solve2arc::evalF( real_type const vars[2], real_type F[2] ) const {
    real_type alpha = vars[0];
    real_type L     = vars[1];
    real_type G[2];
    evalG( alpha, L, th0, k0, G );
    F[0] = G[0] - 2/L;  F[1] = G[1];
    evalG( alpha-1, L, th1, k1, G );
    F[0] -= G[0]; F[1] -= G[1];
  }

  // - - - - - - - - - - - - - - - - - - - - - - - - - - - - - - - - - - - - - -

  void
  G2solve2arc::evalFJ(
    real_type const vars[2],
    real_type       F[2],
    real_type       J[2][2]
  ) const {

    real_type alpha = vars[0];
    real_type L     = vars[1];
    real_type G[2], G_1[2], G_2[2];

    evalG( alpha, L, th0, k0, G, G_1, G_2 );

    F[0]    = G[0] - 2/L;       F[1]    = G[1];
    J[0][0] = G_1[0];           J[1][0] = G_1[1];
    J[0][1] = G_2[0] + 2/(L*L); J[1][1] = G_2[1];

    evalG( alpha-1, L, th1, k1, G, G_1, G_2 );
    F[0]    -= G[0];   F[1]    -= G[1];
    J[0][0] -= G_1[0]; J[1][0] -= G_1[1];
    J[0][1] -= G_2[0]; J[1][1] -= G_2[1];
  }

  // - - - - - - - - - - - - - - - - - - - - - - - - - - - - - - - - - - - - - -

  int
  G2solve2arc::solve() {
    Solve2x2 solver;
    real_type X[2] = { 0.5, 2 };
    int iter = 0;
    bool converged = false;
    do {
      real_type F[2], J[2][2], d[2];
      evalFJ( X, F, J );
      if ( !solver.factorize( J ) ) break;
      solver.solve( F, d );
      real_type lenF = hypot(F[0],F[1]);
      #if 0
      X[0] -= d[0];
      X[1] -= d[1];
      #else
      real_type FF[2], dd[2], XX[2];
      // Affine invariant Newton solver
      real_type nd = hypot( d[0], d[1] );
      bool step_found = false;
      real_type tau = 2;
      do {
        tau  /= 2;
        XX[0] = X[0]-tau*d[0];
        XX[1] = X[1]-tau*d[1];
        evalF(XX, FF);
        solver.solve(FF, dd);
        step_found = hypot( dd[0], dd[1] ) <= (1-tau/2)*nd + 1e-6
                     && XX[0] > 0 && XX[0] < 1 && XX[1] > 0;
      } while ( tau > 1e-6 && !step_found );
      if ( !step_found ) break;
      X[0] = XX[0];
      X[1] = XX[1];
      #endif
      converged = lenF < tolerance;
    } while ( ++iter < maxIter && !converged );
    if ( converged ) converged = X[1] > 0 && X[0] > 0 && X[0] < 1;
    if ( converged ) buildSolution( X[0], X[1] );
    return converged ? iter : -1;
  }

  // - - - - - - - - - - - - - - - - - - - - - - - - - - - - - - - - - - - - - -

  void
  G2solve2arc::buildSolution( real_type alpha, real_type L ) {
    real_type beta = 1-alpha;
    real_type s0   = L*alpha;
    real_type s1   = L*beta;
    real_type tmp  = 2*DeltaTheta-L*(k0+k1);
    real_type A0   = alpha*(s0*DeltaK+tmp);
    real_type A1   = beta*(s1*DeltaK-tmp);

    real_type dk0  = A0/(s0*s0);
    real_type dk1  = A1/(s1*s1);

    // transform solution from (-1,0)--(1,0) to (x0,y0)--(x1,y1)
    //S0.build( -1, 0, th0, k0, dk0, s0 );
    //S1.build(  1, 0, th1, k1, dk1, s1 );
    //S1.changeCurvilinearOrigin( -s1, s1 );
    s0  *= lambda;
    s1  *= lambda;
    dk0 /= lambda*lambda;
    dk1 /= lambda*lambda;

    S0.build( x0, y0, theta0, kappa0, dk0, s0 );
    S1.build( x1, y1, theta1, kappa1, dk1, s1 );
    S1.changeCurvilinearOrigin( -s1, s1 );
  }

  /*\
   |    ____ ____            _            ____ _     ____
   |   / ___|___ \ ___  ___ | |_   _____ / ___| |   / ___|
   |  | |  _  __) / __|/ _ \| \ \ / / _ \ |   | |  | |
   |  | |_| |/ __/\__ \ (_) | |\ V /  __/ |___| |__| |___
   |   \____|_____|___/\___/|_| \_/ \___|\____|_____\____|
  \*/

  int
  G2solveCLC::build(
    real_type _x0,
    real_type _y0,
    real_type _theta0,
    real_type _kappa0,
    real_type _x1,
    real_type _y1,
    real_type _theta1,
    real_type _kappa1
  ) {

    x0     = _x0;
    y0     = _y0;
    theta0 = _theta0;
    kappa0 = _kappa0;
    x1     = _x1;
    y1     = _y1;
    theta1 = _theta1;
    kappa1 = _kappa1;

    // scale problem
    real_type dx = x1 - x0;
    real_type dy = y1 - y0;
    phi    = atan2( dy, dx );
    lambda = hypot( dx, dy );

    real_type C = dx/lambda;
    real_type S = dy/lambda;
    lambda /= 2;

    xbar = -(x0*C+y0*S+lambda);
    ybar = x0*S-y0*C;

    th0 = theta0 - phi;
    th1 = theta1 - phi;

    k0 = kappa0*lambda;
    k1 = kappa1*lambda;

    return solve();
  }

  // - - - - - - - - - - - - - - - - - - - - - - - - - - - - - - - - - - - - - -

  void
  G2solveCLC::setTolerance( real_type tol ) {
    UTILS_ASSERT(
      tol > 0 && tol <= 0.1,
      "G2solveCLC::setTolerance, tolerance = {} must be in (0,0.1]\n", tol
    );
    tolerance = tol;
  }

  // - - - - - - - - - - - - - - - - - - - - - - - - - - - - - - - - - - - - - -

  void
  G2solveCLC::setMaxIter( int miter ) {
    UTILS_ASSERT(
      miter > 0 && miter <= 1000,
      "G2solveCLC::setMaxIter, maxIter = {} must be in [1,1000]\n", miter
    );
    maxIter = miter;
  }

  // - - - - - - - - - - - - - - - - - - - - - - - - - - - - - - - - - - - - - -

  int
  G2solveCLC::solve() {
    real_type X0[3], Y0[3], X1[3], Y1[3];
    real_type thM = 0, sM = 0.0;
    int iter = 0;
    bool converged = false;
    do {
      real_type D0 = thM - th0;
      real_type D1 = thM - th1;

      GeneralizedFresnelCS( 3, 2*D0, -2*D0, D0, X0, Y0 );
      GeneralizedFresnelCS( 3, 2*D1, -2*D1, D1, X1, Y1 );

      real_type F  = D0*k1*Y0[0]-D1*k0*Y1[0] - k0*k1*sin(thM);
      real_type dF = D0*k1*(X0[2]-2*X0[1]+X0[0])
                     - D1*k0*(X1[2]-2*X1[1]+X1[0])
                     - k0*k1*cos(thM)
                     + k1*Y0[0]-k0*Y1[0];

      if ( abs(dF) < 1e-10 ) break;
      real_type d = F/dF;
      #if 0
      thM -= d;
      #else
      real_type FF, dd, thM1;
      // Affine invariant Newton solver
      bool step_found = false;
      real_type tau = 2;
      do {
        tau  /= 2;
        thM1 = thM-tau*d;
        D0 = thM1 - th0;
        D1 = thM1 - th1;
        GeneralizedFresnelCS( 1, 2*D0, -2*D0, D0, X0, Y0 );
        GeneralizedFresnelCS( 1, 2*D1, -2*D1, D1, X1, Y1 );
        FF = D0*k1*Y0[0]-D1*k0*Y1[0] - k0*k1*sin(thM1);
        dd = FF/dF;
        step_found = abs( dd ) <= (1-tau/2)*abs(d) + 1e-6;
      } while ( tau > 1e-6 && !step_found );
      if ( !step_found ) break;
      thM = thM1;
      #endif
      converged = abs(d) < tolerance;
    } while ( ++iter < maxIter && !converged );
    if ( converged ) {
      real_type D0 = thM - th0;
      real_type D1 = thM - th1;
      GeneralizedFresnelCS( 1, 2*D0, -2*D0, D0, X0, Y0 );
      GeneralizedFresnelCS( 1, 2*D1, -2*D1, D1, X1, Y1 );
      sM = cos(thM) + D1*X1[0]/k1 - D0*X0[0]/k0;
      converged = sM > 0 && sM < 1e100;
    }
    if ( converged ) converged = buildSolution( sM, thM );
    return converged ? iter : -1;
  }

  // - - - - - - - - - - - - - - - - - - - - - - - - - - - - - - - - - - - - - -

  bool
  G2solveCLC::buildSolution( real_type sM, real_type thM ) {
    real_type dk0 = 0.5*power2(k0/lambda)/(th0-thM);
    real_type dk1 = 0.5*power2(k1/lambda)/(th1-thM);
    real_type L0  = 2*lambda*(thM-th0)/k0;
    real_type L1  = 2*lambda*(th1-thM)/k1;

    if ( ! ( L0 > 0 && L1 > 0 ) ) return false;

    S0.build( x0, y0, theta0, kappa0, dk0, L0 );
    S1.build( x1, y1, theta1, kappa1, dk1, L1 );
    S1.changeCurvilinearOrigin( -L1, L1 );
    SM.build( S0.xEnd(), S0.yEnd(), S0.thetaEnd(), 0, 0, 2*sM*lambda );

    return true;
  }

  /*\
   |    ____ ____            _           _____
   |   / ___|___ \ ___  ___ | |_   _____|___ /  __ _ _ __ ___
   |  | |  _  __) / __|/ _ \| \ \ / / _ \ |_ \ / _` | '__/ __|
   |  | |_| |/ __/\__ \ (_) | |\ V /  __/___) | (_| | | | (__
   |   \____|_____|___/\___/|_| \_/ \___|____/ \__,_|_|  \___|
  \*/

  void
  G2solve3arc::setTolerance( real_type tol ) {
    UTILS_ASSERT(
      tol > 0 && tol <= 0.1,
      "G2solve3arc::setTolerance, tolerance = {} must be in (0,0.1]\n", tol
    );
    tolerance = tol;
  }

  // - - - - - - - - - - - - - - - - - - - - - - - - - - - - - - - - - - - - - -

  void
  G2solve3arc::setMaxIter( int miter ) {
    UTILS_ASSERT(
      miter > 0 && miter <= 1000,
      "G2solve3arc::setMaxIter, maxIter = {} must be in [1,1000]\n", miter
    );
    maxIter = miter;
  }

  // - - - - - - - - - - - - - - - - - - - - - - - - - - - - - - - - - - - - - -

  int
  G2solve3arc::build(
    real_type _x0,
    real_type _y0,
    real_type _theta0,
    real_type _kappa0,
    real_type _x1,
    real_type _y1,
    real_type _theta1,
    real_type _kappa1,
    real_type Dmax,
    real_type dmax
  ) {
    try {
      // save data
      x0     = _x0;
      y0     = _y0;
      theta0 = _theta0;
      kappa0 = _kappa0;
      x1     = _x1;
      y1     = _y1;
      theta1 = _theta1;
      kappa1 = _kappa1;

      // transform to reference frame
      real_type dx = x1 - x0;
      real_type dy = y1 - y0;
      phi    = atan2( dy, dx );
      Lscale = 2/hypot( dx, dy );

      th0 = theta0 - phi;
      th1 = theta1 - phi;

      // put in range
      rangeSymm(th0);
      rangeSymm(th1);

      K0 = (kappa0/Lscale); // k0
      K1 = (kappa1/Lscale); // k1

      if ( Dmax <= 0 ) Dmax = Utils::m_pi;
      if ( dmax <= 0 ) dmax = Utils::m_pi/8;

      if ( Dmax > Utils::m_2pi  ) Dmax = Utils::m_2pi;
      if ( dmax > Utils::m_pi/4 ) dmax = Utils::m_pi/4;

      // compute guess G1
      ClothoidCurve SG;
      SG.build_G1( -1, 0, th0, 1, 0, th1 );

      real_type kA = SG.kappaBegin();
      real_type kB = SG.kappaEnd();
      real_type dk = abs(SG.dkappa());
      real_type L3 = SG.length()/3;

      real_type tmp = 0.5*abs(K0-kA)/dmax;
      s0 = L3;
      if ( tmp*s0 > 1 ) s0 = 1/tmp;
      tmp = (abs(K0+kA)+s0*dk)/(2*Dmax);
      if ( tmp*s0 > 1 ) s0 = 1/tmp;

      tmp = 0.5*abs(K1-kB)/dmax;
      s1 = L3;
      if ( tmp*s1 > 1 ) s1 = 1/tmp;
      tmp = (abs(K1+kB)+s1*dk)/(2*Dmax);
      if ( tmp*s1 > 1 ) s1 = 1/tmp;

      real_type dth   = abs(th0-th1) / Utils::m_2pi;
      real_type scale = power3(cos( power4(dth)*Utils::m_pi_2 ));
      s0 *= scale;
      s1 *= scale;

      real_type L   = (3*L3-s0-s1)/2;
      real_type thM = SG.theta(s0+L);
      th0 = SG.thetaBegin();
      th1 = SG.thetaEnd();

      // setup

      K0 *= s0;
      K1 *= s1;

      real_type t0 = 2*th0+K0;
      real_type t1 = 2*th1-K1;

      c0  = s0*s1;
      c1  = 2 * s0;
      c2  = 0.25*((K1-6*(K0+th0)-2*th1)*s0 - 3*K0*s1);
      c3  = -c0 * (K0 + th0);
      c4  = 2 * s1;
      c5  = 0.25*((6*(K1-th1)-K0-2*th0)*s1 + 3*K1*s0);
      c6  = c0 * (K1 - th1);
      c7  = -0.5*(s0 + s1);
      c8  = th0 + th1 + 0.5*(K0 - K1);
      c9  = 0.25*(t1*s0 + t0*s1);
      c10 = 0.5*(s1 - s0);
      c11 = 0.5*(th1 - th0) - 0.25*(K0 + K1);
      c12 = 0.25*(t1*s0 - t0*s1);
      c13 = 0.5*s0*s1;
      c14 = 0.75*(s0 + s1);
      return solve( L, thM );
    } catch (...) {
      return -1;
      // nothing to do
    }
  }

  // - - - - - - - - - - - - - - - - - - - - - - - - - - - - - - - - - - - - - -

  int
  G2solve3arc::build_fixed_length(
    real_type _s0,
    real_type _x0,
    real_type _y0,
    real_type _theta0,
    real_type _kappa0,
    real_type _s1,
    real_type _x1,
    real_type _y1,
    real_type _theta1,
    real_type _kappa1
  ) {
    try {
      // save data
      x0     = _x0;
      y0     = _y0;
      theta0 = _theta0;
      kappa0 = _kappa0;
      x1     = _x1;
      y1     = _y1;
      theta1 = _theta1;
      kappa1 = _kappa1;

      // transform to reference frame
      real_type dx = x1 - x0;
      real_type dy = y1 - y0;
      phi    = atan2( dy, dx );
      Lscale = 2/hypot( dx, dy );

      th0 = theta0 - phi;
      th1 = theta1 - phi;

      // put in range
      rangeSymm(th0);
      rangeSymm(th1);

      K0 = (kappa0/Lscale); // k0
      K1 = (kappa1/Lscale); // k1

      // compute guess G1
      ClothoidCurve SG;
      SG.build_G1( -1, 0, th0, 1, 0, th1 );

      s0 = _s0 * Lscale;
      s1 = _s1 * Lscale;

      real_type L   = (SG.length()-s0-s1)/2;
      real_type thM = SG.theta(s0+L);
      th0 = SG.thetaBegin();
      th1 = SG.thetaEnd();

      // setup

      K0 *= s0;
      K1 *= s1;

      real_type t0 = 2*th0+K0;
      real_type t1 = 2*th1-K1;

      c0  = s0*s1;
      c1  = 2 * s0;
      c2  = 0.25*((K1-6*(K0+th0)-2*th1)*s0 - 3*K0*s1);
      c3  = -c0 * (K0 + th0);
      c4  = 2 * s1;
      c5  = 0.25*((6*(K1-th1)-K0-2*th0)*s1 + 3*K1*s0);
      c6  = c0 * (K1 - th1);
      c7  = -0.5*(s0 + s1);
      c8  = th0 + th1 + 0.5*(K0 - K1);
      c9  = 0.25*(t1*s0 + t0*s1);
      c10 = 0.5*(s1 - s0);
      c11 = 0.5*(th1 - th0) - 0.25*(K0 + K1);
      c12 = 0.25*(t1*s0 - t0*s1);
      c13 = 0.5*s0*s1;
      c14 = 0.75*(s0 + s1);

      return solve( L, thM );

    } catch (...) {

      return -1;
      // nothing to do
    }
  }

  // - - - - - - - - - - - - - - - - - - - - - - - - - - - - - - - - - - - - - -

  void
  G2solve3arc::evalF( real_type const vars[2], real_type F[2] ) const {

    real_type sM  = vars[0];
    real_type thM = vars[1];

    real_type dsM = 1.0 / (c13+(c14+sM)*sM);
    real_type dK0 = dsM*(c0*thM + sM*(c1*thM - K0*sM + c2) + c3);
    real_type dK1 = dsM*(c0*thM + sM*(c4*thM + K1*sM + c5) + c6);
    real_type dKM = dsM*sM*( thM*(c7-2*sM) + c8*sM + c9);
    real_type KM  = dsM*sM*(c10*thM + c11*sM + c12);

    real_type X0, Y0, X1, Y1, XMp, YMp, XMm, YMm;
    GeneralizedFresnelCS( dK0,  K0, th0, X0,  Y0);
    GeneralizedFresnelCS( dK1, -K1, th1, X1,  Y1);
    GeneralizedFresnelCS( dKM,  KM, thM, XMp, YMp);
    GeneralizedFresnelCS( dKM, -KM, thM, XMm, YMm);

    // in the standard problem dx = 2, dy = 0
    F[0] = s0*X0 + s1*X1 + sM*(XMm + XMp) - 2;
    F[1] = s0*Y0 + s1*Y1 + sM*(YMm + YMp) - 0;
  }

  // - - - - - - - - - - - - - - - - - - - - - - - - - - - - - - - - - - - - - -

  void
  G2solve3arc::evalFJ(
    real_type const vars[2],
    real_type       F[2],
    real_type       J[2][2]
  ) const {

    real_type sM  = vars[0];
    real_type thM = vars[1];

    real_type dsM   = 1.0 / (c13+(c14+sM)*sM);
    real_type dsMsM = dsM*sM;
    real_type dK0   = dsM*(c0*thM + sM*(c1*thM + c2 - sM*K0) + c3);
    real_type dK1   = dsM*(c0*thM + sM*(c4*thM + c5 + sM*K1) + c6);
    real_type dKM   = dsMsM*(thM*(c7-2*sM) + c8*sM + c9);
    real_type KM    = dsMsM*(c10*thM + c11*sM + c12);

    real_type X0[3],  Y0[3],
              X1[3],  Y1[3],
              XMp[3], YMp[3],
              XMm[3], YMm[3];
    GeneralizedFresnelCS( 3, dK0,  K0, th0, X0,  Y0);
    GeneralizedFresnelCS( 3, dK1, -K1, th1, X1,  Y1);
    GeneralizedFresnelCS( 3, dKM,  KM, thM, XMp, YMp);
    GeneralizedFresnelCS( 3, dKM, -KM, thM, XMm, YMm);

    // in the standard problem dx = 2, dy = 0
    real_type t0 = XMp[0]+XMm[0];
    real_type t1 = YMp[0]+YMm[0];
    F[0] = s0*X0[0] + s1*X1[0] + sM*t0 - 2;
    F[1] = s0*Y0[0] + s1*Y1[0] + sM*t1 - 0;

    // calcolo J(F)
    real_type dsM2 = dsM*dsM;
    real_type g0   = -(2 * sM + c14)*dsM2;
    real_type g1   = (c13 - sM*sM)*dsM2;
    real_type g2   = sM*(sM*c14+2*c13)*dsM2;

    real_type dK0_sM  = (c0*thM+c3)*g0 + (c1*thM+c2)*g1 - K0*g2;
    real_type dK1_sM  = (c0*thM+c6)*g0 + (c4*thM+c5)*g1 + K1*g2;
    real_type dKM_sM  = (c7*thM+c9)*g1 + (c8-2*thM)*g2;
    real_type KM_sM   = (c10*thM+c12)*g1 + c11*g2;

    real_type dK0_thM = (c0+c1*sM)*dsM;
    real_type dK1_thM = (c0+c4*sM)*dsM;
    real_type dKM_thM = (c7-2*sM)*dsMsM;
    real_type KM_thM  = c10*dsMsM;

    // coeff fresnel per f_j per lo jacobiano
    real_type f0 = -0.5*s0*Y0[2];
    real_type f1 = -0.5*s1*Y1[2];
    real_type f2 = -0.5*sM*(YMm[2] + YMp[2]);
    real_type f3 = sM*(YMm[1] - YMp[1]);
    real_type f4 = 0.5*s0*X0[2];
    real_type f5 = 0.5*s1*X1[2];
    real_type f6 = 0.5*sM*(XMm[2] + XMp[2]);
    real_type f7 = sM*(XMp[1] - XMm[1]);

    J[0][0] = f0 * dK0_sM  + f1 * dK1_sM  + f2 * dKM_sM  + f3 * KM_sM  + t0;
    J[0][1] = f0 * dK0_thM + f1 * dK1_thM + f2 * dKM_thM + f3 * KM_thM - sM * t1;
    J[1][0] = f4 * dK0_sM  + f5 * dK1_sM  + f6 * dKM_sM  + f7 * KM_sM  + t1;
    J[1][1] = f4 * dK0_thM + f5 * dK1_thM + f6 * dKM_thM + f7 * KM_thM + sM * t0;
  }

  // - - - - - - - - - - - - - - - - - - - - - - - - - - - - - - - - - - - - - -

  int
  G2solve3arc::solve( real_type sM_guess, real_type thM_guess ) {

    Solve2x2 solver;
    real_type F[2], d[2], X[2], J[2][2];
    X[0] = sM_guess;
    X[1] = thM_guess;

    //real_type thmin = min(th0,th1)-2*m_2pi;
    //real_type thmax = max(th0,th1)+2*m_2pi;

    int iter = 0;
    bool converged = false;
    try {
      do {
        evalFJ(X, F, J);
        real_type lenF = hypot(F[0], F[1]);
        converged = lenF < tolerance;
        if ( converged || !solver.factorize(J) ) break;
        solver.solve(F, d);
        #if 1
        // use undamped Newton
        X[0] -= d[0];
        X[1] -= d[1];
        #else
        real_type FF[2], dd[2], XX[2];
        // Affine invariant Newton solver
        real_type nd = hypot( d[0], d[1] );
        bool step_found = false;
        real_type tau = 2;
        do {
          tau  /= 2;
          XX[0] = X[0]-tau*d[0];
          XX[1] = X[1]-tau*d[1];
          evalF(XX, FF);
          solver.solve(FF, dd);
          step_found = hypot( dd[0], dd[1] ) <= (1-tau/2)*nd + 1e-6;
                       //&& XX[0] > 0; // && XX[0] > X[0]/4 && XX[0] < 4*X[0];
                       //&& XX[1] > thmin && XX[1] < thmax;
        } while ( tau > 1e-6 && !step_found );
        if ( !step_found ) break;
        X[0] = XX[0];
        X[1] = XX[1];
        #endif
      } while ( ++iter < maxIter );

      // re-check solution
      if ( converged )
        converged = FP_INFINITE != fpclassify(X[0]) &&
                    FP_NAN      != fpclassify(X[0]) &&
                    FP_INFINITE != fpclassify(X[1]) &&
                    FP_NAN      != fpclassify(X[1]);
    }
    catch (...) {
      std::cerr << "G2solve3arc::solve, something go wrong\n";
      // nothing to do
    }
    if ( converged ) buildSolution(X[0], X[1]); // costruisco comunque soluzione
    return converged ? iter : -1;
  }

  // - - - - - - - - - - - - - - - - - - - - - - - - - - - - - - - - - - - - - -

  void
  G2solve3arc::buildSolution( real_type sM, real_type thM ) {
    // soluzione nel frame di riferimento
    /* real_type k0 = K0
     S0.build( -1, 0, th0, k0, dK0,   0, L0 );
     S1.build( x1, y1, phi+th1, kappa1, dK1, -L1, 0  );
     S1.change_origin(-L1);
    */

    // ricostruzione dati clotoidi trasformati
    real_type dsM = 1.0 / (c13+(c14+sM)*sM);
    real_type dK0 = dsM*(c0*thM + sM*(c1*thM - K0*sM + c2) + c3);
    real_type dK1 = dsM*(c0*thM + sM*(c4*thM + K1*sM + c5) + c6);
    real_type dKM = dsM*sM*(c7*thM + sM*(c8 - 2*thM) + c9);
    real_type KM  = dsM*sM*(c10*thM + c11*sM + c12);

    real_type xa, ya, xmL, ymL;
    GeneralizedFresnelCS( dK0,  K0, th0, xa,  ya  );
    GeneralizedFresnelCS( dKM, -KM, thM, xmL, ymL );

    real_type xM = s0 * xa + sM * xmL - 1;
    real_type yM = s0 * ya + sM * ymL;

    // rovescia trasformazione standard
    real_type L0 = s0/Lscale;
    real_type L1 = s1/Lscale;
    real_type LM = sM/Lscale;

    dK0 *= power2(Lscale/s0);
    dK1 *= power2(Lscale/s1);
    dKM *= power2(Lscale/sM);
    KM  *= Lscale/sM;

    //th0 = theta0 - phi;
    //th1 = theta1 - phi;
    S0.build( x0, y0, phi+th0, kappa0, dK0, L0 );
    S1.build( x1, y1, phi+th1, kappa1, dK1, L1 );
    S1.changeCurvilinearOrigin( -L1, L1 );

    // la trasformazione inversa da [-1,1] a (x0,y0)-(x1,y1)
    // g(x,y) = RotInv(phi)*(1/lambda*[X;Y] - [xbar;ybar]) = [x;y]

    real_type C  = cos(phi);
    real_type S  = sin(phi);
    real_type dx = (xM + 1) / Lscale;
    real_type dy = yM / Lscale;
    SM.build(
      x0 + C * dx - S * dy,
      y0 + C * dy + S * dx,
      thM + phi, KM, dKM, 2*LM
    );
    SM.changeCurvilinearOrigin( -LM, 2*LM );
  }

  // - - - - - - - - - - - - - - - - - - - - - - - - - - - - - - - - - - - - - -

  real_type
  G2solve3arc::thetaMinMax( real_type & thMin, real_type & thMax ) const {
    real_type thMin1, thMax1;
    S0.thetaMinMax( thMin,  thMax );
    S1.thetaMinMax( thMin1, thMax1 );
    if ( thMin > thMin1 ) thMin = thMin1;
    if ( thMax < thMax1 ) thMax = thMax1;
    SM.thetaMinMax( thMin1, thMax1 );
    if ( thMin > thMin1 ) thMin = thMin1;
    if ( thMax < thMax1 ) thMax = thMax1;
    return thMax-thMin;
  }

  // - - - - - - - - - - - - - - - - - - - - - - - - - - - - - - - - - - - - - -

  real_type
  G2solve3arc::curvatureMinMax( real_type & kMin, real_type & kMax ) const {
    real_type kMin1, kMax1;
    S0.curvatureMinMax( kMin,  kMax );
    S1.curvatureMinMax( kMin1, kMax1 );
    if ( kMin > kMin1 ) kMin = kMin1;
    if ( kMax < kMax1 ) kMax = kMax1;
    SM.curvatureMinMax( kMin1, kMax1 );
    if ( kMin > kMin1 ) kMin = kMin1;
    if ( kMax < kMax1 ) kMax = kMax1;
    return kMax-kMin;
  }

  // - - - - - - - - - - - - - - - - - - - - - - - - - - - - - - - - - - - - - -

  real_type
  G2solve3arc::theta( real_type s ) const {
    if ( s < S0.length() ) return S0.theta(s);
    s -= S0.length();
    if ( s < SM.length() ) return SM.theta(s);
    s -= S0.length();
    return S1.theta(s);
  }

  // - - - - - - - - - - - - - - - - - - - - - - - - - - - - - - - - - - - - - -

  real_type
  G2solve3arc::theta_D( real_type s ) const {
    if ( s < S0.length() ) return S0.theta_D(s);
    s -= S0.length();
    if ( s < SM.length() ) return SM.theta_D(s);
    s -= S0.length();
    return S1.theta_D(s);
  }

  // - - - - - - - - - - - - - - - - - - - - - - - - - - - - - - - - - - - - - -

  real_type
  G2solve3arc::theta_DD( real_type s ) const {
    if ( s < S0.length() ) return S0.theta_DD(s);
    s -= S0.length();
    if ( s < SM.length() ) return SM.theta_DD(s);
    s -= S0.length();
    return S1.theta_DD(s);
  }

  // - - - - - - - - - - - - - - - - - - - - - - - - - - - - - - - - - - - - - -

  real_type
  G2solve3arc::theta_DDD( real_type s ) const {
    if ( s < S0.length() ) return S0.theta_DDD(s);
    s -= S0.length();
    if ( s < SM.length() ) return SM.theta_DDD(s);
    s -= S0.length();
    return S1.theta_DDD(s);
  }

  // - - - - - - - - - - - - - - - - - - - - - - - - - - - - - - - - - - - - - -

  real_type
  G2solve3arc::X( real_type s ) const {
    if ( s < S0.length() ) return S0.X(s);
    s -= S0.length();
    if ( s < SM.length() ) return SM.X(s);
    s -= S0.length();
    return S1.X(s);
  }

  // - - - - - - - - - - - - - - - - - - - - - - - - - - - - - - - - - - - - - -

  real_type
  G2solve3arc::Y( real_type s ) const {
    if ( s < S0.length() ) return S0.Y(s);
    s -= S0.length();
    if ( s < SM.length() ) return SM.Y(s);
    s -= S0.length();
    return S1.Y(s);
  }

  // - - - - - - - - - - - - - - - - - - - - - - - - - - - - - - - - - - - - - -

  void
  G2solve3arc::eval(
    real_type   s,
    real_type & theta,
    real_type & kappa,
    real_type & x,
    real_type & y
  ) const {
    if ( s < S0.length() ) {
      S0.evaluate( s, theta, kappa, x, y );
    } else {
      s -= S0.length();
      if ( s < SM.length() ) {
        SM.evaluate( s, theta, kappa, x, y );
      } else {
        s -= SM.length();
        S1.evaluate( s, theta, kappa, x, y );
      }
    }
  }

  // - - - - - - - - - - - - - - - - - - - - - - - - - - - - - - - - - - - - - -

  void
  G2solve3arc::eval(
    real_type   s,
    real_type & x,
    real_type & y
  ) const {
    if ( s < S0.length() ) {
      S0.eval(s, x, y );
    } else {
      s -= S0.length();
      if ( s < SM.length() ) {
        SM.eval(s, x, y );
      } else {
        s -= SM.length();
        S1.eval(s, x, y );
      }
    }
  }

  // - - - - - - - - - - - - - - - - - - - - - - - - - - - - - - - - - - - - - -

  void
  G2solve3arc::eval_D(
    real_type   s,
    real_type & x_D,
    real_type & y_D
  ) const {
    if ( s < S0.length() ) {
      S0.eval_D(s, x_D, y_D );
    } else {
      s -= S0.length();
      if ( s < SM.length() ) {
        SM.eval_D(s, x_D, y_D );
      } else {
        s -= SM.length();
        S1.eval_D(s, x_D, y_D );
      }
    }
  }

  // - - - - - - - - - - - - - - - - - - - - - - - - - - - - - - - - - - - - - -

  void
  G2solve3arc::eval_DD(
    real_type   s,
    real_type & x_DD,
    real_type & y_DD
  ) const {
    if ( s < S0.length() ) {
      S0.eval_DD(s, x_DD, y_DD );
    } else {
      s -= S0.length();
      if ( s < SM.length() ) {
        SM.eval_DD(s, x_DD, y_DD );
      } else {
        s -= SM.length();
        S1.eval_DD(s, x_DD, y_DD );
      }
    }
  }

  // - - - - - - - - - - - - - - - - - - - - - - - - - - - - - - - - - - - - - -

  void
  G2solve3arc::eval_DDD(
    real_type   s,
    real_type & x_DDD,
    real_type & y_DDD
  ) const {
    if ( s < S0.length() ) {
      S0.eval_DDD(s, x_DDD, y_DDD );
    } else {
      s -= S0.length();
      if ( s < SM.length() ) {
        SM.eval_DDD(s, x_DDD, y_DDD );
      } else {
        s -= SM.length();
        S1.eval_DDD(s, x_DDD, y_DDD );
      }
    }
  }

  // - - - - - - - - - - - - - - - - - - - - - - - - - - - - - - - - - - - - - -

  // offset curve
  void
  G2solve3arc::eval_ISO(
    real_type   s,
    real_type   offs,
    real_type & x,
    real_type & y
  ) const {
    if ( s < S0.length() ) {
      S0.eval_ISO( s, offs, x, y );
    } else {
      s -= S0.length();
      if ( s < SM.length() ) {
        SM.eval_ISO( s, offs, x, y );
      } else {
        s -= SM.length();
        S1.eval_ISO( s, offs, x, y );
      }
    }
  }

  // - - - - - - - - - - - - - - - - - - - - - - - - - - - - - - - - - - - - - -

  void
  G2solve3arc::eval_ISO_D(
    real_type   s,
    real_type   offs,
    real_type & x_D,
    real_type & y_D
  ) const {
    if ( s < S0.length() ) {
      S0.eval_ISO_D( s, offs, x_D, y_D );
    } else {
      s -= S0.length();
      if ( s < SM.length() ) {
        SM.eval_ISO_D( s, offs, x_D, y_D );
      } else {
        s -= SM.length();
        S1.eval_ISO_D( s, offs, x_D, y_D );
      }
    }
  }

  // - - - - - - - - - - - - - - - - - - - - - - - - - - - - - - - - - - - - - -

  void
  G2solve3arc::eval_ISO_DD(
    real_type   s,
    real_type   offs,
    real_type & x_DD,
    real_type & y_DD
  ) const {
    if ( s < S0.length() ) {
      S0.eval_ISO_DD( s, offs, x_DD, y_DD );
    } else {
      s -= S0.length();
      if ( s < SM.length() ) {
        SM.eval_ISO_DD( s, offs, x_DD, y_DD );
      } else {
        s -= SM.length();
        S1.eval_ISO_DD( s, offs, x_DD, y_DD );
      }
    }
  }

  // - - - - - - - - - - - - - - - - - - - - - - - - - - - - - - - - - - - - - -

  void
  G2solve3arc::eval_ISO_DDD(
    real_type   s,
    real_type   offs,
    real_type & x_DDD,
    real_type & y_DDD
  ) const {
    if ( s < S0.length() ) {
      S0.eval_ISO_DDD( s, offs, x_DDD, y_DDD );
    } else {
      s -= S0.length();
      if ( s < SM.length() ) {
        SM.eval_ISO_DDD( s, offs, x_DDD, y_DDD );
      } else {
        s -= SM.length();
        S1.eval_ISO_DDD( s, offs, x_DDD, y_DDD );
      }
    }
  }

  /*\
   |
   |    ___ _     _   _        _    _ ___      _ _           ___ ___
   |   / __| |___| |_| |_  ___(_)__| / __|_ __| (_)_ _  ___ / __|_  )
   |  | (__| / _ \  _| ' \/ _ \ / _` \__ \ '_ \ | | ' \/ -_) (_ |/ /
   |   \___|_\___/\__|_||_\___/_\__,_|___/ .__/_|_|_||_\___|\___/___|
   |                                     |_|
  \*/

  void
  ClothoidSplineG2::guess(
    real_type * theta_guess,
    real_type * theta_min,
    real_type * theta_max
  ) const {
    size_t nn = size_t( m_npts );
    Utils::Malloc<real_type> mem( "ClothoidSplineG2::guess" );
    mem.allocate( 2*nn );
    real_type * omega = mem(nn);
    real_type * len   = mem(nn);
    G2lib::xy_to_guess_angle(
      m_npts, m_x, m_y, theta_guess, theta_min, theta_max, omega, len
    );
  }

  void
  ClothoidSplineG2::build(
    real_type const * xvec,
    real_type const * yvec,
    int_type          n
  ) {
    m_npts = n;
    size_t n1 = size_t(n-1);

    realValues.reallocate( 2*size_t(n) + 10 * n1 );

    m_x    = realValues( size_t(n) );
    m_y    = realValues( size_t(n) );
    m_k    = realValues( n1 );
    m_dk   = realValues( n1 );
    m_L    = realValues( n1 );
    m_kL   = realValues( n1 );
    m_L_1  = realValues( n1 );
    m_L_2  = realValues( n1 );
    m_k_1  = realValues( n1 );
    m_k_2  = realValues( n1 );
    m_dk_1 = realValues( n1 );
    m_dk_2 = realValues( n1 );
    std::copy_n( xvec, n, m_x );
    std::copy_n( yvec, n, m_y );
  }

  // - - - - - - - - - - - - - - - - - - - - - - - - - - - - - - - - - - - - - -

  int_type
  ClothoidSplineG2::numTheta() const { return m_npts; }

  // - - - - - - - - - - - - - - - - - - - - - - - - - - - - - - - - - - - - - -

  int_type
  ClothoidSplineG2::numConstraints() const {
    switch (m_tt) {
      case P1:
      case P2: return m_npts;
      default: break;
    }
    return m_npts-2;
  }

  // - - - - - - - - - - - - - - - - - - - - - - - - - - - - - - - - - - - - - -

  bool
  ClothoidSplineG2::objective(
    real_type const * theta,
    real_type       & f
  ) const {
    ClothoidCurve cL, cR, c;
    int_type ne  = m_npts - 1;
    int_type ne1 = m_npts - 2;
    switch (m_tt) {
    case P1:
    case P2:
      f = 0;
      break;
    case P3:
      // forward target
      break;
    case P4:
      cL.build_G1( m_x[0],   m_y[0],   theta[0],   m_x[1],  m_y[1],  theta[1] );
      cR.build_G1( m_x[ne1], m_y[ne1], theta[ne1], m_x[ne], m_y[ne], theta[ne] );
      { real_type dk_L = cL.dkappa();
        real_type dk_R = cR.dkappa();
        f = dk_L*dk_L+dk_R*dk_R;
      }
      break;
    case P5:
      cL.build_G1( m_x[0],   m_y[0],   theta[0],   m_x[1],  m_y[1],  theta[1] );
      cR.build_G1( m_x[ne1], m_y[ne1], theta[ne1], m_x[ne], m_y[ne], theta[ne] );
      f = cL.length()+cR.length();
      break;
    case P6:
      f = 0;
      for ( int_type j = 0; j < ne; ++j ) {
        c.build_G1( m_x[j], m_y[j], theta[j], m_x[j+1], m_y[j+1], theta[j+1] );
        f += c.length();
      }
      break;
    case P7:
      f = 0;
      for ( int_type j = 0; j < ne; ++j ) {
        c.build_G1( m_x[j], m_y[j], theta[j], m_x[j+1], m_y[j+1], theta[j+1] );
        real_type Len  = c.length();
        real_type kur  = c.kappaBegin();
        real_type dkur = c.dkappa();
        f = f + Len * ( Len * ( dkur*( (dkur*Len)/3 + kur) ) + kur*kur );
      }
      break;
    case P8:
      f = 0;
      for ( int_type j = 0; j < ne; ++j ) {
        c.build_G1( m_x[j], m_y[j], theta[j], m_x[j+1], m_y[j+1], theta[j+1] );
        real_type Len  = c.length();
        real_type dkur = c.dkappa();
        f += Len*dkur*dkur;
      }
      break;
    case P9:
      f = 0;
      for ( int_type j = 0; j < ne; ++j ) {
        c.build_G1( m_x[j], m_y[j], theta[j], m_x[j+1], m_y[j+1], theta[j+1] );
        real_type Len  = c.length();
        real_type kur  = c.kappaBegin();
        real_type k2   = kur*kur;
        real_type k3   = k2*kur;
        real_type k4   = k2*k2;
        real_type dkur = c.dkappa();
        real_type dk2  = dkur*dkur;
        real_type dk3  = dkur*dk2;
        f = f + (k4+dk2+(2*k3*dkur+(2*k2*dk2+(dk3*(kur+dkur*Len/5))*Len)*Len)*Len)*Len;
      }
      break;
    }
    return true;
  }

  // - - - - - - - - - - - - - - - - - - - - - - - - - - - - - - - - - - - - - -

  bool
  ClothoidSplineG2::gradient(
    real_type const * theta,
    real_type       * g
  ) const {
    ClothoidCurve cL, cR, c;
    real_type     LL_D[2], kL_D[2], dkL_D[2];
    real_type     LR_D[2], kR_D[2], dkR_D[2];
    std::fill_n( g, m_npts, 0 );
    int_type ne  = m_npts - 1;
    int_type ne1 = m_npts - 2;
    switch (m_tt) {
    case P1:
    case P2:
      break;
    case P3:
      break;
    case P4:
      cL.build_G1_D(
        m_x[0], m_y[0], theta[0],
        m_x[1], m_y[1], theta[1],
        LL_D, kL_D, dkL_D
      );
      cR.build_G1_D(
        m_x[ne1], m_y[ne1], theta[ne1],
        m_x[ne],  m_y[ne],  theta[ne],
        LR_D, kR_D, dkR_D
      );
      {
        real_type dkL = cL.dkappa();
        real_type dkR = cR.dkappa();
        g[0]   = 2*dkL*dkL_D[0];
        g[1]   = 2*dkL*dkL_D[1];
        g[ne1] = 2*dkR*dkR_D[0];
        g[ne]  = 2*dkR*dkR_D[1];
      }
      break;
    case P5:
      cL.build_G1_D(
        m_x[0], m_y[0], theta[0],
        m_x[1], m_y[1], theta[1],
        LL_D, kL_D, dkL_D
      );
      cR.build_G1_D(
        m_x[ne1], m_y[ne1], theta[ne1],
        m_x[ne],  m_y[ne],  theta[ne],
        LR_D, kR_D, dkR_D
      );
      g[0]   = LL_D[0];
      g[1]   = LL_D[1];
      g[ne1] = LR_D[0];
      g[ne]  = LR_D[1];
      break;
    case P6:
      for ( int_type j = 0; j < ne; ++j ) {
        real_type L_D[2], k_D[2], dk_D[2];
        c.build_G1_D(
          m_x[j],   m_y[j],   theta[j],
          m_x[j+1], m_y[j+1], theta[j+1],
          L_D, k_D, dk_D
        );
        g[j]   += L_D[0];
        g[j+1] += L_D[1];
      }
      break;
    case P7:
      for ( int_type j = 0; j < ne; ++j ) {
        real_type L_D[2], k_D[2], dk_D[2];
        c.build_G1_D(
          m_x[j],   m_y[j],   theta[j],
          m_x[j+1], m_y[j+1], theta[j+1],
          L_D, k_D, dk_D
        );
        real_type Len  = c.length();
        real_type L2   = Len*Len;
        real_type L3   = Len*L2;
        real_type kur  = c.kappaBegin();
        real_type k2   = kur*kur;
        real_type dkur = c.dkappa();
        real_type dk2  = dkur*dkur;
        g[j]   += 2*(dkur*dk_D[0]*L3)/3
                  + (dk2*L2*L_D[0])
                  + dk_D[0]*L2*kur
                  + 2*dkur*Len*L_D[0]*kur
                  + dkur*L2*k_D[0]
                  + L_D[0]*k2
                  + 2*Len*kur*k_D[0];
        g[j+1] += 2*(dkur*dk_D[1]*L3)/3
                  + (dk2*L2*L_D[1])
                  + dk_D[1]*L2*kur
                  + 2*dkur*Len*L_D[1]*kur
                  + dkur*L2*k_D[1]
                  + L_D[1]*k2
                  + 2*Len*kur*k_D[1];
      }
      break;
    case P8:
      for ( int_type j = 0; j < ne; ++j ) {
        real_type L_D[2], k_D[2], dk_D[2];
        c.build_G1_D(
          m_x[j],   m_y[j],   theta[j],
          m_x[j+1], m_y[j+1], theta[j+1],
          L_D, k_D, dk_D
        );
        real_type Len  = c.length();
        real_type dkur = c.dkappa();
        g[j]   += (2*Len*dk_D[0] + L_D[0]*dkur)*dkur;
        g[j+1] += (2*Len*dk_D[1] + L_D[1]*dkur)*dkur;
      }
      break;
    case P9:
      for ( int_type j = 0; j < ne; ++j ) {
        real_type L_D[2], k_D[2], dk_D[2];
        c.build_G1_D(
          m_x[j],   m_y[j],   theta[j],
          m_x[j+1], m_y[j+1], theta[j+1],
          L_D, k_D, dk_D
        );
        real_type Len  = c.length();
        real_type kur  = c.kappaBegin();
        real_type k2   = kur*kur;
        real_type k3   = kur*k2;
        real_type dkur = c.dkappa();
        real_type dk2  = dkur*dkur;
        real_type dkL  = dkur*Len;
        real_type A = ( ( (dkL+4*kur)*dkL + 6*k2)*dkL + 4*k3) * dkL + dk2 + k2*k2;
        real_type B = ( ( ( ( 3*kur + 0.8*dkL ) * dkL + 4*k2 ) * dkL +2*k3 ) * Len + 2*dkur ) * Len;
        real_type C = ( ( ( dkL + 4*kur ) * dkL + 6*k2 ) * dkL + 4*k3 ) * Len;
        g[j]   += A*L_D[0] + B*dk_D[0] + C*k_D[0];
        g[j+1] += A*L_D[1] + B*dk_D[1] + C*k_D[1];
      }
      break;
    }
    return true;
  }

  // - - - - - - - - - - - - - - - - - - - - - - - - - - - - - - - - - - - - - -

  bool
  ClothoidSplineG2::constraints(
    real_type const * theta,
    real_type       * c
  ) const {
    ClothoidCurve cc;
    int_type ne  = m_npts - 1;
    int_type ne1 = m_npts - 2;

    for ( int_type j = 0; j < ne; ++j ) {
      cc.build_G1( m_x[j], m_y[j], theta[j], m_x[j+1], m_y[j+1], theta[j+1] );
      m_k[j]  = cc.kappaBegin();
      m_dk[j] = cc.dkappa();
      m_L[j]  = cc.length();
      m_kL[j] = m_k[j]+m_dk[j]*m_L[j];
    }

    for ( int_type j = 0; j < ne1; ++j ) c[j] = m_kL[j]-m_k[j+1];

    switch (m_tt) {
    case P1:
      c[ne1] = diff2pi( theta[0]  - m_theta_I );
      c[ne]  = diff2pi( theta[ne] - m_theta_F );
      break;
    case P2:
      c[ne1] = m_kL[ne1] - m_k[0];
      c[ne]  = diff2pi( theta[0] - theta[ne] );
      break;
    default:
      break;
    }
    return true;
  }

  // - - - - - - - - - - - - - - - - - - - - - - - - - - - - - - - - - - - - - -

  int_type
  ClothoidSplineG2::jacobian_nnz() const {
    int_type nnz = 3*(m_npts-2);
    switch (m_tt) {
    case P1: nnz += 2; break;
    case P2: nnz += 6; break;
    default:            break;
    }
    return nnz;
  }

  // - - - - - - - - - - - - - - - - - - - - - - - - - - - - - - - - - - - - - -

  bool
  ClothoidSplineG2::jacobian_pattern(
    int_type * ii,
    int_type * jj
  ) const {
    ClothoidCurve cc;
    int_type ne  = m_npts - 1;
    int_type ne1 = m_npts - 2;

    int_type kk = 0;
    for ( int_type j = 0; j < ne1; ++j ) {
      ii[kk] = j; jj[kk] = j; ++kk;
      ii[kk] = j; jj[kk] = j+1; ++kk;
      ii[kk] = j; jj[kk] = j+2; ++kk;
    }

    switch (m_tt) {
    case P1:
      ii[kk] = ne1; jj[kk] = 0; ++kk;
      ii[kk] = ne; jj[kk] = ne; ++kk;
      break;
    case P2:
      ii[kk] = ne1; jj[kk] = 0; ++kk;
      ii[kk] = ne1; jj[kk] = 1; ++kk;
      ii[kk] = ne1; jj[kk] = ne1; ++kk;
      ii[kk] = ne1; jj[kk] = ne; ++kk;
      ii[kk] = ne; jj[kk] = 0; ++kk;
      ii[kk] = ne; jj[kk] = ne; ++kk;
      break;
    default:
      break;
    }

    return true;
  }

  // - - - - - - - - - - - - - - - - - - - - - - - - - - - - - - - - - - - - - -

  bool
  ClothoidSplineG2::jacobian_pattern_matlab(
    real_type * ii,
    real_type * jj
  ) const {
    ClothoidCurve cc;
    int_type ne  = m_npts - 1;
    int_type ne1 = m_npts - 2;

    int_type kk = 0;
    for ( int_type j = 1; j <= ne1; ++j ) {
      ii[kk] = j; jj[kk] = j;   ++kk;
      ii[kk] = j; jj[kk] = j+1; ++kk;
      ii[kk] = j; jj[kk] = j+2; ++kk;
    }

    switch (m_tt) {
    case P1:
      ii[kk] = ne;     jj[kk] = 1;      ++kk;
      ii[kk] = m_npts; jj[kk] = m_npts; ++kk;
      break;
    case P2:
      ii[kk] = ne;     jj[kk] = 1;      ++kk;
      ii[kk] = ne;     jj[kk] = 2;      ++kk;
      ii[kk] = ne;     jj[kk] = ne;     ++kk;
      ii[kk] = ne;     jj[kk] = m_npts; ++kk;
      ii[kk] = m_npts; jj[kk] = 1;      ++kk;
      ii[kk] = m_npts; jj[kk] = m_npts; ++kk;
      break;
    default:
      break;
    }

    return true;
  }

  // - - - - - - - - - - - - - - - - - - - - - - - - - - - - - - - - - - - - - -

  bool
  ClothoidSplineG2::jacobian(
    real_type const * theta,
    real_type       * vals
  ) const {
    ClothoidCurve cc;
    int_type ne  = m_npts - 1;
    int_type ne1 = m_npts - 2;

    for ( int_type j = 0; j < ne; ++j ) {
      real_type L_D[2], k_D[2], dk_D[2];
      cc.build_G1_D(
        m_x[j],   m_y[j],   theta[j],
        m_x[j+1], m_y[j+1], theta[j+1],
        L_D, k_D, dk_D
      );
      m_k[j]    = cc.kappaBegin();
      m_dk[j]   = cc.dkappa();
      m_L[j]    = cc.length();
      m_kL[j]   = m_k[j]+m_dk[j]*m_L[j];
      m_L_1[j]  = L_D[0];  m_L_2[j]  = L_D[1];
      m_k_1[j]  = k_D[0];  m_k_2[j]  = k_D[1];
      m_dk_1[j] = dk_D[0]; m_dk_2[j] = dk_D[1];
    }

    int_type kk = 0;
    for ( int_type j = 0; j < ne1; ++j ) {
      vals[kk++] =  m_k_1[j] + m_dk_1[j]*m_L[j] + m_dk[j]*m_L_1[j];
      vals[kk++] =  m_k_2[j] + m_dk_2[j]*m_L[j] + m_dk[j]*m_L_2[j] - m_k_1[j+1];
      vals[kk++] = -m_k_2[j+1];
    }

    switch (m_tt) {
    case P1:
      vals[kk++] = 1;
      vals[kk++] = 1;
      break;
    case P2:
      vals[kk++] = -m_k_1[0];
      vals[kk++] = -m_k_2[0];
      vals[kk++] = m_k_1[ne1]+m_L_1[ne1]*m_dk[ne1]+m_L[ne1]*m_dk_1[ne1];
      vals[kk++] = m_k_2[ne1]+m_L_2[ne1]*m_dk[ne1]+m_L[ne1]*m_dk_2[ne1];
      vals[kk++] = 1;
      vals[kk++] = -1;
      break;
    default:
      break;
    }
    return true;
  }

  // - - - - - - - - - - - - - - - - - - - - - - - - - - - - - - - - - - - - - -

  ostream_type &
  operator << ( ostream_type & stream, ClothoidSplineG2 const & c ) {
    fmt::print( stream,
      "npts   = {}\n"
      "target = {}\n",
      c.m_npts, int(c.m_tt)
    );
    return stream;
  }

}

// EOF: ClothoidG2.cc
```

### Quick search

### Table of Contents

- Matlab Interface Manual
- C++ API
- MATLAB API

«
hide menu

menu
sidebar
»

### Navigation

- index
- toc
- Clothoids »
- Program Listing for File ClothoidG2.cc

© Copyright 2021, Enrico Bertolazzi and Marco Frego.
Created using Sphinx 4.2.0.
