# Supplementary material for: Smooth Interpolating Curves with Local Control and Monotone Alternating Curvature
Source: Comput Graph Forum. 2022 Oct 6;41(5):25–38. doi: 10.1111/cgf.14600 (PMC9827861; doi:10.1111/cgf.14600)
Supplement: Supplementary file 1 — Supplement Material [file CGF-41-25-s001.zip › Local-Smooth-Interpolating-MonoCurvature/extern/clothoids/docs/api-cpp/program_listing_file_ClothoidList.cc.html]

Program Listing for File ClothoidList.cc — Clothoids v2.0.9

### Navigation

- index
- toc
- Clothoids »
- Program Listing for File ClothoidList.cc

# Program Listing for File ClothoidList.cc¶

↰ Return to documentation for file (`ClothoidList.cc`)

```
/*--------------------------------------------------------------------------*\
 |                                                                          |
 |  Copyright (C) 2017                                                      |
 |                                                                          |
 |         , __                 , __                                        |
 |        /|/  \               /|/  \                                       |
 |         | __/ _   ,_         | __/ _   ,_                                |
 |         |   \|/  /  |  |   | |   \|/  /  |  |   |                        |
 |         |(__/|__/   |_/ \_/|/|(__/|__/   |_/ \_/|/                       |
 |                           /|                   /|                        |
 |                           \|                   \|                        |
 |                                                                          |
 |      Enrico Bertolazzi                                                   |
 |      Dipartimento di Ingegneria Industriale                              |
 |      Universita` degli Studi di Trento                                   |
 |      email: enrico.bertolazzi@unitn.it                                   |
 |                                                                          |
\*--------------------------------------------------------------------------*/

#include "Clothoids.hh"

#include <cfloat>
#include <limits>

#ifdef __GNUC__
#pragma GCC diagnostic push
#pragma GCC diagnostic ignored "-Wsign-conversion"
#endif
#ifdef __clang__
#pragma clang diagnostic push
#pragma clang diagnostic ignored "-Wsign-conversion"
#endif

namespace G2lib {

  using std::numeric_limits;
  using std::lower_bound;
  using std::vector;
  using std::swap;
  using std::abs;

  /*\
   |   ____ _       _   _           _     _ _     _     _
   |  / ___| | ___ | |_| |__   ___ (_) __| | |   (_)___| |_
   | | |   | |/ _ \| __| '_ \ / _ \| |/ _` | |   | / __| __|
   | | |___| | (_) | |_| | | | (_) | | (_| | |___| \__ \ |_
   |  \____|_|\___/ \__|_| |_|\___/|_|\__,_|_____|_|___/\__|
   |
  \*/

  ClothoidList::ClothoidList( LineSegment const & LS )
  : BaseCurve(G2LIB_CLOTHOID_LIST)
  , m_curve_is_closed(false)
  , m_aabb_done(false)
  {
    this->resetLastInterval();
    this->init();
    this->push_back( LS );
  }

  ClothoidList::ClothoidList( CircleArc const & C )
  : BaseCurve(G2LIB_CLOTHOID_LIST)
  , m_curve_is_closed(false)
  , m_aabb_done(false)
  {
    this->resetLastInterval();
    this->init();
    this->push_back( C );
  }

  ClothoidList::ClothoidList( Biarc const & C )
  : BaseCurve(G2LIB_CLOTHOID_LIST)
  , m_curve_is_closed(false)
  , m_aabb_done(false)
  {
    this->resetLastInterval();
    this->init();
    this->push_back( C.C0() );
    this->push_back( C.C1() );
  }

  ClothoidList::ClothoidList( BiarcList const & c )
  : BaseCurve(G2LIB_CLOTHOID_LIST)
  , m_curve_is_closed(false)
  , m_aabb_done(false)
  {
    this->resetLastInterval();
    this->init();
    this->push_back( c );
  }

  ClothoidList::ClothoidList( ClothoidCurve const & c )
  : BaseCurve(G2LIB_CLOTHOID_LIST)
  , m_curve_is_closed(false)
  , m_aabb_done(false)
  {
    this->resetLastInterval();
    this->init();
    this->push_back( c );
  }

  ClothoidList::ClothoidList( PolyLine const & pl )
  : BaseCurve(G2LIB_CLOTHOID_LIST)
  , m_curve_is_closed(false)
  , m_aabb_done(false)
  {
    this->resetLastInterval();
    this->init();
    this->push_back( pl );
  }

  ClothoidList::ClothoidList( BaseCurve const & C )
  : BaseCurve(G2LIB_CLOTHOID_LIST)
  , m_curve_is_closed(false)
  , m_aabb_done(false)
  {
    this->resetLastInterval();
    this->init();
    switch ( C.type() ) {
    case G2LIB_LINE:
      push_back( *static_cast<LineSegment const *>(&C) );
      break;
    case G2LIB_CIRCLE:
      push_back( *static_cast<CircleArc const *>(&C) );
      break;
    case G2LIB_CLOTHOID:
      push_back( *static_cast<ClothoidCurve const *>(&C) );
      break;
    case G2LIB_BIARC:
      push_back( *static_cast<Biarc const *>(&C) );
      break;
    case G2LIB_BIARC_LIST:
      push_back( *static_cast<BiarcList const *>(&C) );
      break;
    case G2LIB_CLOTHOID_LIST:
      copy( *static_cast<ClothoidList const *>(&C) );
      break;
    case G2LIB_POLYLINE:
      push_back( *static_cast<PolyLine const *>(&C) );
      break;
    }
  }

  // - - - - - - - - - - - - - - - - - - - - - - - - - - - - - - - - - - - - - -

  int_type
  ClothoidList::findAtS( real_type & s ) const {
    bool ok;
    int_type & lastInterval = *m_lastInterval.search( std::this_thread::get_id(), ok );
    Utils::searchInterval<int_type,real_type>(
      static_cast<int_type>(m_s0.size()),
      &m_s0.front(), s, lastInterval, m_curve_is_closed, true
    );
    return lastInterval;
  }

  // - - - - - - - - - - - - - - - - - - - - - - - - - - - - - - - - - - - - - -

  void
  ClothoidList::wrap_in_range( real_type & s ) const {
    real_type a = m_s0.front();
    real_type b = m_s0.back();
    real_type L = b-a;
    s -= a;
    s  = fmod( s, L );
    if ( s < 0 ) s += L;
    // while ( s < 0 ) s += L;
    // while ( s > L ) s -= L;
    s += a;
  }

  // - - - - - - - - - - - - - - - - - - - - - - - - - - - - - - - - - - - - - -

  void
  ClothoidList::init() {
    m_s0.clear();
    m_clotoidList.clear();
    this->resetLastInterval();
    m_aabb_done = false;
    this->resetLastInterval();
  }

  // - - - - - - - - - - - - - - - - - - - - - - - - - - - - - - - - - - - - - -

  void
  ClothoidList::copy( ClothoidList const & L ) {
    this->init();
    m_clotoidList.reserve( L.m_clotoidList.size() );
    std::copy(
      L.m_clotoidList.begin(), L.m_clotoidList.end(), back_inserter(m_clotoidList)
    );
    m_s0.reserve( L.m_s0.size() );
    std::copy( L.m_s0.begin(), L.m_s0.end(), back_inserter(m_s0) );
  }

  // - - - - - - - - - - - - - - - - - - - - - - - - - - - - - - - - - - - - - -

  void
  ClothoidList::reserve( int_type n ) {
    m_s0.reserve(size_t(n+1));
    m_clotoidList.reserve(size_t(n));
  }

  // - - - - - - - - - - - - - - - - - - - - - - - - - - - - - - - - - - - - - -

  void
  ClothoidList::push_back( LineSegment const & LS ) {
    if ( m_clotoidList.empty() ) {
      m_s0.push_back(0);
      m_s0.push_back(LS.length());
    } else {
      m_s0.push_back(m_s0.back()+LS.length());
    }
    m_clotoidList.push_back(ClothoidCurve(LS));
  }

  // - - - - - - - - - - - - - - - - - - - - - - - - - - - - - - - - - - - - - -

  void
  ClothoidList::push_back( CircleArc const & C ) {
    if ( m_clotoidList.empty() ) {
      m_s0.push_back(0);
      m_s0.push_back(C.length());
    } else {
      m_s0.push_back(m_s0.back()+C.length());
    }
    m_clotoidList.push_back(ClothoidCurve(C));
  }

  // - - - - - - - - - - - - - - - - - - - - - - - - - - - - - - - - - - - - - -

  void
  ClothoidList::push_back( Biarc const & c ) {
    if ( m_clotoidList.empty() ) m_s0.push_back( 0 );
    CircleArc const & C0 = c.C0();
    CircleArc const & C1 = c.C1();
    m_s0.push_back( m_s0.back()+C0.length() );
    m_s0.push_back( m_s0.back()+C1.length() );
    m_clotoidList.push_back( ClothoidCurve(C0) );
    m_clotoidList.push_back( ClothoidCurve(C1) );
  }

  // - - - - - - - - - - - - - - - - - - - - - - - - - - - - - - - - - - - - - -

  void
  ClothoidList::push_back( ClothoidCurve const & c ) {
    if ( m_clotoidList.empty() ) {
      m_s0.push_back(0);
      m_s0.push_back(c.length());
    } else {
      m_s0.push_back(m_s0.back()+c.length());
    }
    m_clotoidList.push_back(c);
  }

  // - - - - - - - - - - - - - - - - - - - - - - - - - - - - - - - - - - - - - -

  void
  ClothoidList::push_back( BiarcList const & c ) {
    m_s0.reserve( m_s0.size() + c.m_biarcList.size() + 1 );
    m_clotoidList.reserve( m_clotoidList.size() + 2*c.m_biarcList.size() );

    if ( m_s0.empty() ) m_s0.push_back(0);

    vector<Biarc>::const_iterator ip = c.m_biarcList.begin();
    for (; ip != c.m_biarcList.end(); ++ip ) {
      m_s0.push_back(m_s0.back()+ip->length());
      Biarc const & b = *ip;
      m_clotoidList.push_back(ClothoidCurve(b.C0()));
      m_clotoidList.push_back(ClothoidCurve(b.C1()));
    }
  }

  // - - - - - - - - - - - - - - - - - - - - - - - - - - - - - - - - - - - - - -

  void
  ClothoidList::push_back( PolyLine const & c ) {
    m_s0.reserve( m_s0.size() + c.m_polylineList.size() + 1 );
    m_clotoidList.reserve( m_clotoidList.size() + c.m_polylineList.size() );

    if ( m_s0.empty() ) m_s0.push_back(0);

    vector<LineSegment>::const_iterator ip = c.m_polylineList.begin();
    for (; ip != c.m_polylineList.end(); ++ip ) {
      m_s0.push_back(m_s0.back()+ip->length());
      m_clotoidList.push_back(ClothoidCurve(*ip));
    }
  }

  // - - - - - - - - - - - - - - - - - - - - - - - - - - - - - - - - - - - - - -

  void
  ClothoidList::push_back( ClothoidList const & c ) {
    m_s0.reserve( m_s0.size() + c.m_clotoidList.size() + 1 );
    m_clotoidList.reserve( m_clotoidList.size() + c.m_clotoidList.size() );

    if ( m_s0.empty() ) m_s0.push_back(0);

    vector<ClothoidCurve>::const_iterator ip = c.m_clotoidList.begin();
    for (; ip != c.m_clotoidList.end(); ++ip ) {
      m_s0.push_back(m_s0.back()+ip->length());
      m_clotoidList.push_back(*ip);
    }
  }

  // - - - - - - - - - - - - - - - - - - - - - - - - - - - - - - - - - - - - - -

  void
  ClothoidList::push_back(
    real_type kappa0,
    real_type dkappa,
    real_type L
  ) {
    UTILS_ASSERT0(
      !m_clotoidList.empty(),
      "ClothoidList::push_back_G1(...) empty list!\n"
    );
    ClothoidCurve c;
    real_type x0     = m_clotoidList.back().xEnd();
    real_type y0     = m_clotoidList.back().yEnd();
    real_type theta0 = m_clotoidList.back().thetaEnd();
    c.build( x0, y0, theta0, kappa0, dkappa, L );
    push_back( c );
  }

  // - - - - - - - - - - - - - - - - - - - - - - - - - - - - - - - - - - - - - -

  void
  ClothoidList::push_back(
    real_type x0,
    real_type y0,
    real_type theta0,
    real_type kappa0,
    real_type dkappa,
    real_type L
  ) {
    ClothoidCurve c;
    c.build( x0, y0, theta0, kappa0, dkappa, L );
    push_back( c );
  }
  // - - - - - - - - - - - - - - - - - - - - - - - - - - - - - - - - - - - - - -

  void
  ClothoidList::push_back_G1(
    real_type x1,
    real_type y1,
    real_type theta1
  ) {
    UTILS_ASSERT0(
      !m_clotoidList.empty(),
      "ClothoidList::push_back_G1(...) empty list!\n"
    );
    ClothoidCurve c;
    real_type x0     = m_clotoidList.back().xEnd();
    real_type y0     = m_clotoidList.back().yEnd();
    real_type theta0 = m_clotoidList.back().thetaEnd();
    c.build_G1( x0, y0, theta0, x1, y1, theta1 );
    push_back( c );
  }

  // - - - - - - - - - - - - - - - - - - - - - - - - - - - - - - - - - - - - - -

  void
  ClothoidList::push_back_G1(
    real_type x0,
    real_type y0,
    real_type theta0,
    real_type x1,
    real_type y1,
    real_type theta1
  ) {
    ClothoidCurve c;
    c.build_G1( x0, y0, theta0, x1, y1, theta1 );
    push_back( c );
  }

  // - - - - - - - - - - - - - - - - - - - - - - - - - - - - - - - - - - - - - -

  bool
  ClothoidList::build_G1(
    int_type          n,
    real_type const * x,
    real_type const * y
  ) {
    init();
    reserve( n-1 );
    ClothoidCurve c;

    UTILS_ASSERT0(
      n > 1, "ClothoidList::build_G1, at least 2 points are necessary\n"
    );

    if ( n == 2 ) {

      real_type theta = atan2( y[1] - y[0], x[1] - x[0] );
      c.build_G1( x[0], y[0], theta, x[1], y[1], theta );
      push_back(c);

    } else {

      Biarc b;
      bool ok, ciclic = hypot( x[0]-x[n-1], y[0]-y[n-1] ) < 1e-10;
      real_type thetaC(0);
      if ( ciclic ) {
        ok = b.build_3P( x[n-2], y[n-2], x[0], y[0], x[1], y[1] );
        UTILS_ASSERT0( ok, "ClothoidList::build_G1, failed\n" );
        thetaC = b.thetaMiddle();
      }
      ok = b.build_3P( x[0], y[0], x[1], y[1], x[2], y[2] );
      UTILS_ASSERT0( ok, "ClothoidList::build_G1, failed\n" );
      real_type theta0 = ciclic ? thetaC : b.thetaBegin();
      real_type theta1 = b.thetaMiddle();
      c.build_G1( x[0], y[0], theta0, x[1], y[1], theta1 );
      push_back(c);
      for ( int_type k = 2; k < n-1; ++k ) {
        theta0 = theta1;
        ok = b.build_3P( x[k-1], y[k-1], x[k], y[k], x[k+1], y[k+1] );
        UTILS_ASSERT0( ok, "ClothoidList::build_G1, failed\n" );
        theta1 = b.thetaMiddle();
        c.build_G1( x[k-1], y[k-1], theta0, x[k], y[k], theta1 );
        push_back(c);
      }
      theta0 = theta1;
      theta1 = ciclic ? thetaC : b.thetaEnd();
      c.build_G1( x[n-2], y[n-2], theta0, x[n-1], y[n-1], theta1 );
      push_back(c);

    }
    return true;
  }

  // - - - - - - - - - - - - - - - - - - - - - - - - - - - - - - - - - - - - - -

  bool
  ClothoidList::build_G1(
    int_type          n,
    real_type const * x,
    real_type const * y,
    real_type const * theta
  ) {

    UTILS_ASSERT0(
      n > 1, "ClothoidList::build_G1, at least 2 points are necessary\n"
    );

    init();
    reserve( n-1 );
    ClothoidCurve c;
    for ( int_type k = 1; k < n; ++k ) {
      c.build_G1( x[k-1], y[k-1], theta[k-1], x[k], y[k], theta[k] );
      push_back(c);
    }
    return true;
  }

  // - - - - - - - - - - - - - - - - - - - - - - - - - - - - - - - - - - - - - -

  bool
  ClothoidList::build(
    real_type         x0,
    real_type         y0,
    real_type         theta0,
    int_type          n,
    real_type const * s,
    real_type const * kappa
  ) {
    if ( n < 2 ) return false;
    real_type tol = abs(s[n-1]-s[0])*machepsi10; // minimum admissible length

    init();
    real_type k  = kappa[0];
    real_type L  = s[1]-s[0];
    real_type dk = (kappa[1]-k)/L;
    UTILS_ASSERT(
      Utils::isRegular( k ) && Utils::isRegular( L ) && Utils::isRegular( dk ),
      "ClothoidList::build, failed first segment found\n"
      "L = {} k = {} dk = {}\n",
      L, k, dk
    );
    push_back( x0, y0, theta0, k, dk, L );
    for ( int_type i = 2; i < n; ++i ) {
      k  = kappa[i-1];
      L  = s[i]-s[i-1];
      if ( abs(L) < tol ) {
        fmt::print( "ClothoidList::build, skipping segment N.{}\n", i);
        continue; // skip too small segment
      }
      dk = (kappa[i]-k)/L;
      UTILS_ASSERT(
        Utils::isRegular( k ) && Utils::isRegular( L ) && Utils::isRegular( dk ),
        "ClothoidList::build, failed at segment N.{} found\n"
        "L = {} k = {} dk = {}\n",
        i, L, k, dk
      );
      push_back( k, dk, L );
    }
    return true;
  }

  // - - - - - - - - - - - - - - - - - - - - - - - - - - - - - - - - - - - - - -

  bool
  ClothoidList::build_raw(
    int_type          n,
    real_type const * x,
    real_type const * y,
    real_type const * abscissa,
    real_type const * theta,
    real_type const * kappa
  ) {
    if ( n < 2 ) return false;
    init();
    m_clotoidList.reserve(size_t(n-1));
    real_type const * px = x;
    real_type const * py = y;
    real_type const * pa = abscissa;
    real_type const * pt = theta;
    real_type const * pk = kappa;
    for ( int_type i = 1; i < n; ++i, ++px, ++py, ++pa, ++pt, ++pk ) {
      real_type dk  = pk[1]-pk[0];
      real_type L   = pa[1]-pa[0];
      push_back( *px, *py, *pt, *pk, dk, L );
    }
    return true;
  }

  // - - - - - - - - - - - - - - - - - - - - - - - - - - - - - - - - - - - - - -

  ClothoidCurve const &
  ClothoidList::get( int_type idx ) const {
    UTILS_ASSERT(
      !m_clotoidList.empty(), "ClothoidList::get( {} ) empty list\n", idx
    );
    UTILS_ASSERT(
      idx >= 0 && idx < int_type(m_clotoidList.size()),
      "ClothoidList::get( {} ) bad index, must be in [0,{}]\n",
      idx, m_clotoidList.size()-1
    );
    return m_clotoidList[idx];
  }

  // - - - - - - - - - - - - - - - - - - - - - - - - - - - - - - - - - - - - - -

  ClothoidCurve const &
  ClothoidList::getAtS( real_type s ) const {
    int_type idx = this->findAtS(s);
    return get( idx );
  }

  /*\
   |   _                  _   _
   |  | | ___ _ __   __ _| |_| |__
   |  | |/ _ \ '_ \ / _` | __| '_ \
   |  | |  __/ | | | (_| | |_| | | |
   |  |_|\___|_| |_|\__, |\__|_| |_|
   |                |___/
  \*/

  real_type
  ClothoidList::length() const
  {
    if ( m_clotoidList.empty() ) return 0.0;
    return m_s0.back() - m_s0.front();
  }

  real_type
  ClothoidList::length_ISO( real_type offs ) const {
    real_type L = 0;
    vector<ClothoidCurve>::const_iterator is = m_clotoidList.begin();
    for (; is != m_clotoidList.end(); ++is ) L += is->length_ISO( offs );
    return L;
  }

  real_type
  ClothoidList::segment_length( int_type nseg ) const {
    ClothoidCurve const & c = get( nseg );
    return c.length();
  }

  real_type
  ClothoidList::segment_length_ISO( int_type nseg, real_type offs ) const {
    ClothoidCurve const & c = get( nseg );
    return c.length_ISO( offs );
  }

  /*\
   |  _    _   _____    _                _
   | | |__| |_|_   _| _(_)__ _ _ _  __ _| |___
   | | '_ \ '_ \| || '_| / _` | ' \/ _` | / -_)
   | |_.__/_.__/|_||_| |_\__,_|_||_\__, |_\___|
   |                               |___/
  \*/

  void
  ClothoidList::bbTriangles(
    vector<Triangle2D> & tvec,
    real_type            max_angle,
    real_type            max_size,
    int_type             icurve
  ) const {
    vector<ClothoidCurve>::const_iterator ic = m_clotoidList.begin();
    for ( int_type ipos = icurve; ic != m_clotoidList.end(); ++ic, ++ipos )
      ic->bbTriangles( tvec, max_angle, max_size, ipos );
  }

  // . . . . . . . . . . . . . . . . . . . . . . . . . . . . . . . . . . . . .

  void
  ClothoidList::bbTriangles_ISO(
    real_type            offs,
    vector<Triangle2D> & tvec,
    real_type            max_angle,
    real_type            max_size,
    int_type             icurve
  ) const {
    vector<ClothoidCurve>::const_iterator ic = m_clotoidList.begin();
    for ( int_type ipos = icurve; ic != m_clotoidList.end(); ++ic, ++ipos )
      ic->bbTriangles_ISO( offs, tvec, max_angle, max_size, ipos );
  }

  /*\
   |   _     _
   |  | |__ | |__   _____  __
   |  | '_ \| '_ \ / _ \ \/ /
   |  | |_) | |_) | (_) >  <
   |  |_.__/|_.__/ \___/_/\_\
  \*/

  void
  ClothoidList::bbox_ISO(
    real_type   offs,
    real_type & xmin,
    real_type & ymin,
    real_type & xmax,
    real_type & ymax
  ) const {
    vector<Triangle2D> tvec;
    bbTriangles_ISO( offs, tvec, Utils::m_pi/18, 1e100 );
    xmin = ymin = numeric_limits<real_type>::infinity();
    xmax = ymax = -xmin;
    vector<Triangle2D>::const_iterator it;
    for ( it = tvec.begin(); it != tvec.end(); ++it ) {
      // - - - - - - - - - - - - - - - - - - - -
      if      ( it->x1() < xmin ) xmin = it->x1();
      else if ( it->x1() > xmax ) xmax = it->x1();
      if      ( it->x2() < xmin ) xmin = it->x2();
      else if ( it->x2() > xmax ) xmax = it->x2();
      if      ( it->x3() < xmin ) xmin = it->x3();
      else if ( it->x3() > xmax ) xmax = it->x3();
      // - - - - - - - - - - - - - - - - - - - -
      if      ( it->y1() < ymin ) ymin = it->y1();
      else if ( it->y1() > ymax ) ymax = it->y1();
      if      ( it->y2() < ymin ) ymin = it->y2();
      else if ( it->y2() > ymax ) ymax = it->y2();
      if      ( it->y3() < ymin ) ymin = it->y3();
      else if ( it->y3() > ymax ) ymax = it->y3();
    }
  }

  /*\
   |  _   _          _
   | | |_| |__   ___| |_ __ _
   | | __| '_ \ / _ \ __/ _` |
   | | |_| | | |  __/ || (_| |
   |  \__|_| |_|\___|\__\__,_|
  \*/

  real_type
  ClothoidList::theta( real_type s ) const {
    int_type idx = findAtS( s );
    ClothoidCurve const & c = get( idx );
    return c.theta( s - m_s0[idx] );
  }

  // - - - - - - - - - - - - - - - - - - - - - - - - - - - - - - - - - - - - - -

  real_type
  ClothoidList::theta_D( real_type s ) const {
    int_type idx = findAtS( s );
    ClothoidCurve const & c = get( idx );
    return c.theta_D( s - m_s0[idx] );
  }

  // - - - - - - - - - - - - - - - - - - - - - - - - - - - - - - - - - - - - - -

  real_type
  ClothoidList::theta_DD( real_type s ) const  {
    int_type idx = findAtS( s );
    ClothoidCurve const & c = get( idx );
    return c.theta_DD( s - m_s0[idx] );
  }

  // - - - - - - - - - - - - - - - - - - - - - - - - - - - - - - - - - - - - - -

  real_type
  ClothoidList::theta_DDD( real_type s ) const {
    int_type idx = findAtS( s );
    ClothoidCurve const & c = get( idx );
    return c.theta_DDD( s - m_s0[idx] );
  }

  /*\
   |  _____                   _    _   _
   | |_   _|   __ _ _ __   __| |  | \ | |
   |   | |    / _` | '_ \ / _` |  |  \| |
   |   | |   | (_| | | | | (_| |  | |\  |
   |   |_|    \__,_|_| |_|\__,_|  |_| \_|
  \*/

  real_type
  ClothoidList::tx( real_type s ) const {
    int_type idx = findAtS( s );
    ClothoidCurve const & c = get( idx );
    return c.tx( s - m_s0[idx] );
  }

  // - - - - - - - - - - - - - - - - - - - - - - - - - - - - - - - - - - - - - -

  real_type
  ClothoidList::ty( real_type s ) const {
    int_type idx = findAtS( s );
    ClothoidCurve const & c = get( idx );
    return c.ty( s - m_s0[idx] );
  }

  // - - - - - - - - - - - - - - - - - - - - - - - - - - - - - - - - - - - - - -

  real_type
  ClothoidList::tx_D( real_type s ) const {
    int_type idx = findAtS( s );
    ClothoidCurve const & c = get( idx );
    return c.tx_D( s - m_s0[idx] );
  }

  // - - - - - - - - - - - - - - - - - - - - - - - - - - - - - - - - - - - - - -

  real_type
  ClothoidList::ty_D( real_type s ) const {
    int_type idx = findAtS( s );
    ClothoidCurve const & c = get( idx );
    return c.ty_D( s - m_s0[idx] );
  }

  // - - - - - - - - - - - - - - - - - - - - - - - - - - - - - - - - - - - - - -

  real_type
  ClothoidList::tx_DD( real_type s ) const {
    int_type idx = findAtS( s );
    ClothoidCurve const & c = get( idx );
    return c.tx_DD( s - m_s0[idx] );
  }

  // - - - - - - - - - - - - - - - - - - - - - - - - - - - - - - - - - - - - - -

  real_type
  ClothoidList::ty_DD( real_type s ) const {
    int_type idx = findAtS( s );
    ClothoidCurve const & c = get( idx );
    return c.ty_DD( s - m_s0[idx] );
  }

  // - - - - - - - - - - - - - - - - - - - - - - - - - - - - - - - - - - - - - -

  real_type
  ClothoidList::tx_DDD( real_type s ) const {
    int_type idx = findAtS( s );
    ClothoidCurve const & c = get( idx );
    return c.tx_DDD( s - m_s0[idx] );
  }

  // - - - - - - - - - - - - - - - - - - - - - - - - - - - - - - - - - - - - - -

  real_type
  ClothoidList::ty_DDD( real_type s ) const {
    int_type idx = findAtS( s );
    ClothoidCurve const & c = get( idx );
    return c.ty_DDD( s - m_s0[idx] );
  }

  // - - - - - - - - - - - - - - - - - - - - - - - - - - - - - - - - - - - - - -

  void
  ClothoidList::tg(
    real_type   s,
    real_type & tg_x,
    real_type & tg_y
  ) const {
    int_type idx = findAtS( s );
    ClothoidCurve const & c = get( idx );
    return c.tg( s - m_s0[idx], tg_x, tg_y );
  }

  // - - - - - - - - - - - - - - - - - - - - - - - - - - - - - - - - - - - - - -

  void
  ClothoidList::tg_D(
    real_type   s,
    real_type & tg_x_D,
    real_type & tg_y_D
  ) const {
    int_type idx = findAtS( s );
    ClothoidCurve const & c = get( idx );
    return c.tg_D( s - m_s0[idx], tg_x_D, tg_y_D );
  }

  // - - - - - - - - - - - - - - - - - - - - - - - - - - - - - - - - - - - - - -

  void
  ClothoidList::tg_DD(
    real_type   s,
    real_type & tg_x_DD,
    real_type & tg_y_DD
  ) const {
    int_type idx = findAtS( s );
    ClothoidCurve const & c = get( idx );
    return c.tg_DD( s - m_s0[idx], tg_x_DD, tg_y_DD );
  }

  // - - - - - - - - - - - - - - - - - - - - - - - - - - - - - - - - - - - - - -

  void
  ClothoidList::tg_DDD(
    real_type   s,
    real_type & tg_x_DDD,
    real_type & tg_y_DDD
  ) const {
    int_type idx = findAtS( s );
    ClothoidCurve const & c = get( idx );
    return c.tg_DDD( s - m_s0[idx], tg_x_DDD, tg_y_DDD );
  }

  // - - - - - - - - - - - - - - - - - - - - - - - - - - - - - - - - - - - - - -

  void
  ClothoidList::evaluate(
    real_type   s,
    real_type & th,
    real_type & k,
    real_type & x,
    real_type & y
  ) const {
    int_type idx = findAtS( s );
    ClothoidCurve const & c = get( idx );
    c.evaluate( s - m_s0[idx], th, k, x, y );
  }

  // - - - - - - - - - - - - - - - - - - - - - - - - - - - - - - - - - - - - - -

  void
  ClothoidList::evaluate_ISO(
    real_type   s,
    real_type   offs,
    real_type & th,
    real_type & k,
    real_type & x,
    real_type & y
  ) const {
    int_type idx = findAtS( s );
    ClothoidCurve const & c = get( idx );
    c.evaluate_ISO( s - m_s0[idx], offs, th, k, x, y );
  }

  // - - - - - - - - - - - - - - - - - - - - - - - - - - - - - - - - - - - - - -

  real_type
  ClothoidList::X( real_type s ) const {
    int_type idx = findAtS( s );
    ClothoidCurve const & c = get( idx );
    return c.X( s - m_s0[idx] );
  }

  // - - - - - - - - - - - - - - - - - - - - - - - - - - - - - - - - - - - - - -

  real_type
  ClothoidList::Y( real_type s ) const  {
    int_type idx = findAtS( s );
    ClothoidCurve const & c = get( idx );
    return c.Y( s - m_s0[idx] );
  }

  // - - - - - - - - - - - - - - - - - - - - - - - - - - - - - - - - - - - - - -

  real_type
  ClothoidList::X_D( real_type s ) const {
    int_type idx = findAtS( s );
    ClothoidCurve const & c = get( idx );
    return c.X_D( s - m_s0[idx] );
  }

  // - - - - - - - - - - - - - - - - - - - - - - - - - - - - - - - - - - - - - -

  real_type
  ClothoidList::Y_D( real_type s ) const {
    int_type idx = findAtS( s );
    ClothoidCurve const & c = get( idx );
    return c.Y_D( s - m_s0[idx] );
  }

  // - - - - - - - - - - - - - - - - - - - - - - - - - - - - - - - - - - - - - -

  real_type
  ClothoidList::X_DD( real_type s ) const {
    int_type idx = findAtS( s );
    ClothoidCurve const & c = get( idx );
    return c.X_DD( s - m_s0[idx] );
  }

  // - - - - - - - - - - - - - - - - - - - - - - - - - - - - - - - - - - - - - -

  real_type
  ClothoidList::Y_DD( real_type s ) const {
    int_type idx = findAtS( s );
    ClothoidCurve const & c = get( idx );
    return c.Y_DD( s - m_s0[idx] );
  }

  // - - - - - - - - - - - - - - - - - - - - - - - - - - - - - - - - - - - - - -

  real_type
  ClothoidList::X_DDD( real_type s ) const {
    int_type idx = findAtS( s );
    ClothoidCurve const & c = get( idx );
    return c.X_DDD( s - m_s0[idx] );
  }

  // - - - - - - - - - - - - - - - - - - - - - - - - - - - - - - - - - - - - - -

  real_type
  ClothoidList::Y_DDD( real_type s ) const {
    int_type idx = findAtS( s );
    ClothoidCurve const & c = get( idx );
    return c.Y_DDD( s - m_s0[idx] );
  }

  // - - - - - - - - - - - - - - - - - - - - - - - - - - - - - - - - - - - - - -

  void
  ClothoidList::eval(
    real_type   s,
    real_type & x,
    real_type & y
  ) const {
    int_type idx = findAtS( s );
    ClothoidCurve const & c = get( idx );
    return c.eval( s - m_s0[idx], x, y );
  }

  // - - - - - - - - - - - - - - - - - - - - - - - - - - - - - - - - - - - - - -

  void
  ClothoidList::eval_D(
    real_type   s,
    real_type & x_D,
    real_type & y_D
  ) const {
    int_type idx = findAtS( s );
    ClothoidCurve const & c = get( idx );
    return c.eval_D( s - m_s0[idx], x_D, y_D );
  }

  // - - - - - - - - - - - - - - - - - - - - - - - - - - - - - - - - - - - - - -

  void
  ClothoidList::eval_DD(
    real_type   s,
    real_type & x_DD,
    real_type & y_DD
  ) const {
    int_type idx = findAtS( s );
    ClothoidCurve const & c = get( idx );
    return c.eval_DD( s - m_s0[idx], x_DD, y_DD );
  }

  // - - - - - - - - - - - - - - - - - - - - - - - - - - - - - - - - - - - - - -

  void
  ClothoidList::eval_DDD(
    real_type   s,
    real_type & x_DDD,
    real_type & y_DDD
  ) const {
    int_type idx = findAtS( s );
    ClothoidCurve const & c = get( idx );
    return c.eval_DDD( s - m_s0[idx], x_DDD, y_DDD );
  }

  /*\
   |         __  __          _
   |   ___  / _|/ _|___  ___| |_
   |  / _ \| |_| |_/ __|/ _ \ __|
   | | (_) |  _|  _\__ \  __/ |_
   |  \___/|_| |_| |___/\___|\__|
  \*/

  real_type
  ClothoidList::X_ISO( real_type s, real_type offs ) const {
    int_type idx = findAtS( s );
    ClothoidCurve const & c = get( idx );
    return c.X_ISO( s - m_s0[idx], offs );
  }

  // - - - - - - - - - - - - - - - - - - - - - - - - - - - - - - - - - - - - - -

  real_type
  ClothoidList::Y_ISO( real_type s, real_type offs ) const {
    int_type idx = findAtS( s );
    ClothoidCurve const & c = get( idx );
    return c.Y_ISO( s - m_s0[idx], offs );
  }

  // - - - - - - - - - - - - - - - - - - - - - - - - - - - - - - - - - - - - - -

  real_type
  ClothoidList::X_ISO_D( real_type s, real_type offs ) const {
    int_type idx = findAtS( s );
    ClothoidCurve const & c = get( idx );
    return c.X_ISO_D( s - m_s0[idx], offs );
  }

  // - - - - - - - - - - - - - - - - - - - - - - - - - - - - - - - - - - - - - -

  real_type
  ClothoidList::Y_ISO_D( real_type s, real_type offs ) const {
    int_type idx = findAtS( s );
    ClothoidCurve const & c = get( idx );
    return c.Y_ISO_D( s - m_s0[idx], offs );
  }

  // - - - - - - - - - - - - - - - - - - - - - - - - - - - - - - - - - - - - - -

  real_type
  ClothoidList::X_ISO_DD( real_type s, real_type offs ) const {
    int_type idx = findAtS( s );
    ClothoidCurve const & c = get( idx );
    return c.X_ISO_DD( s - m_s0[idx], offs );
  }

  // - - - - - - - - - - - - - - - - - - - - - - - - - - - - - - - - - - - - - -

  real_type
  ClothoidList::Y_ISO_DD( real_type s, real_type offs ) const {
    int_type idx = findAtS( s );
    ClothoidCurve const & c = get( idx );
    return c.Y_ISO_DD( s - m_s0[idx], offs );
  }

  // - - - - - - - - - - - - - - - - - - - - - - - - - - - - - - - - - - - - - -

  real_type
  ClothoidList::X_ISO_DDD( real_type s, real_type offs ) const {
    int_type idx = findAtS( s );
    ClothoidCurve const & c = get( idx );
    return c.X_ISO_DDD( s - m_s0[idx], offs );
  }

  // - - - - - - - - - - - - - - - - - - - - - - - - - - - - - - - - - - - - - -

  real_type
  ClothoidList::Y_ISO_DDD( real_type s, real_type offs ) const {
    int_type idx = findAtS( s );
    ClothoidCurve const & c = get( idx );
    return c.Y_ISO_DDD( s - m_s0[idx], offs );
  }

  // - - - - - - - - - - - - - - - - - - - - - - - - - - - - - - - - - - - - - -

  void
  ClothoidList::eval_ISO(
    real_type   s,
    real_type   offs,
    real_type & x,
    real_type & y
  ) const {
    int_type idx = findAtS( s );
    ClothoidCurve const & c = get( idx );
    return c.eval_ISO( s - m_s0[idx], offs, x, y );
  }

  // - - - - - - - - - - - - - - - - - - - - - - - - - - - - - - - - - - - - - -

  void
  ClothoidList::eval_ISO_D(
    real_type   s,
    real_type   offs,
    real_type & x_D,
    real_type & y_D
  ) const {
    int_type idx = findAtS( s );
    ClothoidCurve const & c = get( idx );
    return c.eval_ISO_D( s - m_s0[idx], offs, x_D, y_D );
  }

  // - - - - - - - - - - - - - - - - - - - - - - - - - - - - - - - - - - - - - -

  void
  ClothoidList::eval_ISO_DD(
    real_type   s,
    real_type   offs,
    real_type & x_DD,
    real_type & y_DD
  ) const {
    int_type idx = findAtS( s );
    ClothoidCurve const & c = get( idx );
    return c.eval_ISO_DD( s - m_s0[idx], offs, x_DD, y_DD );
  }

  // - - - - - - - - - - - - - - - - - - - - - - - - - - - - - - - - - - - - - -

  void
  ClothoidList::eval_ISO_DDD(
    real_type   s,
    real_type   offs,
    real_type & x_DDD,
    real_type & y_DDD
  ) const {
    int_type idx = findAtS( s );
    ClothoidCurve const & c = get( idx );
    return c.eval_ISO_DDD( s - m_s0[idx], offs, x_DDD, y_DDD );
  }

  /*\
   |  _                        __
   | | |_ _ __ __ _ _ __  ___ / _| ___  _ __ _ __ ___
   | | __| '__/ _` | '_ \/ __| |_ / _ \| '__| '_ ` _ \
   | | |_| | | (_| | | | \__ \  _| (_) | |  | | | | | |
   |  \__|_|  \__,_|_| |_|___/_|  \___/|_|  |_| |_| |_|
  \*/

  void
  ClothoidList::translate( real_type tx, real_type ty ) {
    vector<ClothoidCurve>::iterator ic = m_clotoidList.begin();
    for (; ic != m_clotoidList.end(); ++ic ) ic->translate( tx, ty );
  }

  // - - - - - - - - - - - - - - - - - - - - - - - - - - - - - - - - - - - - - -

  void
  ClothoidList::rotate( real_type angle, real_type cx, real_type cy ) {
    vector<ClothoidCurve>::iterator ic = m_clotoidList.begin();
    for (; ic != m_clotoidList.end(); ++ic ) ic->rotate( angle, cx, cy );
  }

  // - - - - - - - - - - - - - - - - - - - - - - - - - - - - - - - - - - - - - -

  void
  ClothoidList::scale( real_type sfactor ) {
    vector<ClothoidCurve>::iterator ic = m_clotoidList.begin();
    real_type newx0 = ic->xBegin();
    real_type newy0 = ic->yBegin();
    m_s0[0] = 0;
    for ( size_t k=0; ic != m_clotoidList.end(); ++ic, ++k ) {
      ic->scale( sfactor );
      ic->changeOrigin( newx0, newy0 );
      newx0     = ic->xEnd();
      newy0     = ic->yEnd();
      m_s0[k+1] = m_s0[k] + ic->length();
    }
  }

  // - - - - - - - - - - - - - - - - - - - - - - - - - - - - - - - - - - - - - -

  void
  ClothoidList::reverse() {
    std::reverse( m_clotoidList.begin(), m_clotoidList.end() );
    vector<ClothoidCurve>::iterator ic = m_clotoidList.begin();
    ic->reverse();
    real_type newx0 = ic->xEnd();
    real_type newy0 = ic->yEnd();
    m_s0[0] = 0;
    m_s0[1] = ic->length();
    size_t k = 1;
    for ( ++ic; ic != m_clotoidList.end(); ++ic, ++k ) {
      ic->reverse();
      ic->changeOrigin( newx0, newy0 );
      newx0     = ic->xEnd();
      newy0     = ic->yEnd();
      m_s0[k+1] = m_s0[k] + ic->length();
    }
  }

  // - - - - - - - - - - - - - - - - - - - - - - - - - - - - - - - - - - - - - -

  void
  ClothoidList::changeOrigin( real_type newx0, real_type newy0 ) {
    vector<ClothoidCurve>::iterator ic = m_clotoidList.begin();
    for (; ic != m_clotoidList.end(); ++ic ) {
      ic->changeOrigin( newx0, newy0 );
      newx0 = ic->xEnd();
      newy0 = ic->yEnd();
    }
  }

  // - - - - - - - - - - - - - - - - - - - - - - - - - - - - - - - - - - - - - -

  void
  ClothoidList::trim( real_type s_begin, real_type s_end ) {

    ClothoidList newCL;
    this->trim( s_begin, s_end, newCL );
    this->copy( newCL );

#if 0
    UTILS_ASSERT(
      s_begin >= m_s0.front() && s_end <= m_s0.back() && s_end > s_begin,
      "ClothoidList::trim( s_begin={}, s_end={} ) bad range, must be in [{},{}]\n",
      s_begin, s_end, m_s0.front(), m_s0.back()
    );

    size_t i_begin = size_t( findAtS( s_begin ) );
    size_t i_end   = size_t( findAtS( s_end   ) );
    if ( i_begin == i_end ) {
      m_clotoidList[i_begin].trim( s_begin-m_s0[i_begin], s_end-m_s0[i_begin] );
    } else {
      m_clotoidList[i_begin].trim( s_begin-m_s0[i_begin], m_s0[i_begin+1]-m_s0[i_begin] );
      m_clotoidList[i_end].trim( 0, s_end-m_s0[i_end] );
    }
    m_clotoidList.erase( m_clotoidList.begin()+i_end+1, m_clotoidList.end() );
    m_clotoidList.erase( m_clotoidList.begin(), m_clotoidList.begin()+i_begin );
    if ( m_clotoidList.back().m_L <= machepsi100 ) m_clotoidList.pop_back();
    vector<ClothoidCurve>::iterator ic = m_clotoidList.begin();
    m_s0.resize( m_clotoidList.size() + 1 );
    m_s0[0] = 0;
    size_t k = 0;
    for (; ic != m_clotoidList.end(); ++ic, ++k )
      m_s0[k+1] = m_s0[k] + ic->length();
    this->resetLastInterval();
#endif
  }

  // - - - - - - - - - - - - - - - - - - - - - - - - - - - - - - - - - - - - - -

  void
  ClothoidList::trim( real_type s_begin, real_type s_end, ClothoidList & newCL ) const {

    newCL.init();

    if ( m_clotoidList.empty() ) return;

    // put in range
    real_type L = this->length();
    while ( s_begin > L ) s_begin -= L;
    while ( s_begin < 0 ) s_begin += L;
    while ( s_end   > L ) s_end   -= L;
    while ( s_end   < 0 ) s_end   += L;

    int_type n_seg   = int_type( m_clotoidList.size() );
    int_type i_begin = findAtS( s_begin );
    int_type i_end   = findAtS( s_end );

    if ( s_begin < s_end ) {
      // get initial and final segment
      if ( i_begin == i_end ) { // stesso segmento
        real_type   ss0 = m_s0[i_begin];
        ClothoidCurve C = m_clotoidList[i_begin];
        C.trim( s_begin-ss0, s_end-ss0 );
        newCL.push_back( C );
      } else {
        ClothoidCurve C0 = m_clotoidList[i_begin];
        C0.trim( s_begin - m_s0[i_begin], C0.length() );
        newCL.push_back( C0 );

        for ( ++i_begin; i_begin < i_end; ++i_begin )
          newCL.push_back( m_clotoidList[i_begin] );

        ClothoidCurve C1 = m_clotoidList[i_end];
        C1.trim( 0, s_end - m_s0[i_end] );
        newCL.push_back( C1 );
      }
    } else {
      ClothoidCurve C0 = m_clotoidList[i_begin];
      C0.trim( s_begin - m_s0[i_begin], C0.length() );
      newCL.push_back( C0 );

      for ( ++i_begin; i_begin < n_seg; ++i_begin )
        newCL.push_back( m_clotoidList[i_begin] );

      for ( i_begin = 0; i_begin < i_end; ++i_begin )
        newCL.push_back( m_clotoidList[i_begin] );

      ClothoidCurve C1 = m_clotoidList[i_end];
      C1.trim( 0, s_end - m_s0[i_end] );
      newCL.push_back( C1 );
    }
  }

  /*\
   |     _        _    ____  ____  _
   |    / \      / \  | __ )| __ )| |_ _ __ ___  ___
   |   / _ \    / _ \ |  _ \|  _ \| __| '__/ _ \/ _ \
   |  / ___ \  / ___ \| |_) | |_) | |_| | |  __/  __/
   | /_/   \_\/_/   \_\____/|____/ \__|_|  \___|\___|
  \*/

  #ifndef DOXYGEN_SHOULD_SKIP_THIS
  void
  ClothoidList::build_AABBtree_ISO(
    real_type offs,
    real_type max_angle,
    real_type max_size
  ) const {

    if ( m_aabb_done &&
         Utils::isZero( offs-m_aabb_offs ) &&
         Utils::isZero( max_angle-m_aabb_max_angle ) &&
         Utils::isZero( max_size-m_aabb_max_size ) ) return;

    #ifdef G2LIB_USE_CXX11
    vector<shared_ptr<BBox const> > bboxes;
    #else
    vector<BBox const *> bboxes;
    #endif

    bbTriangles_ISO( offs, m_aabb_tri, max_angle, max_size );
    bboxes.reserve(m_aabb_tri.size());
    vector<Triangle2D>::const_iterator it;
    int_type ipos = 0;
    for ( it = m_aabb_tri.begin(); it != m_aabb_tri.end(); ++it, ++ipos ) {
      real_type xmin, ymin, xmax, ymax;
      it->bbox( xmin, ymin, xmax, ymax );
      #ifdef G2LIB_USE_CXX11
      bboxes.push_back( make_shared<BBox const>(
        xmin, ymin, xmax, ymax, G2LIB_CLOTHOID, ipos
      ) );
      #else
      bboxes.push_back(
        new BBox( xmin, ymin, xmax, ymax, G2LIB_CLOTHOID, ipos )
      );
      #endif
    }
    m_aabb_tree.build(bboxes);
    m_aabb_done      = true;
    m_aabb_offs      = offs;
    m_aabb_max_angle = max_angle;
    m_aabb_max_size  = max_size;
  }
  #endif

  /*\
   |   _       _                          _
   |  (_)_ __ | |_ ___ _ __ ___  ___  ___| |_
   |  | | '_ \| __/ _ \ '__/ __|/ _ \/ __| __|
   |  | | | | | ||  __/ |  \__ \  __/ (__| |_
   |  |_|_| |_|\__\___|_|  |___/\___|\___|\__|
  \*/

  // - - - - - - - - - - - - - - - - - - - - - - - - - - - - - - - - - - - - - -

  bool
  ClothoidList::collision( ClothoidList const & C ) const {
    this->build_AABBtree_ISO( 0 );
    C.build_AABBtree_ISO( 0 );
    T2D_collision_list_ISO fun( this, 0, &C, 0 );
    return m_aabb_tree.collision( C.m_aabb_tree, fun, false );
  }

  // - - - - - - - - - - - - - - - - - - - - - - - - - - - - - - - - - - - - - -

  bool
  ClothoidList::collision_ISO(
    real_type            offs,
    ClothoidList const & C,
    real_type            offs_C
  ) const {
    this->build_AABBtree_ISO( offs );
    C.build_AABBtree_ISO( offs_C );
    T2D_collision_list_ISO fun( this, offs, &C, offs_C );
    return m_aabb_tree.collision( C.m_aabb_tree, fun, false );
  }

  /*\
   |   _       _                          _
   |  (_)_ __ | |_ ___ _ __ ___  ___  ___| |_
   |  | | '_ \| __/ _ \ '__/ __|/ _ \/ __| __|
   |  | | | | | ||  __/ |  \__ \  __/ (__| |_
   |  |_|_| |_|\__\___|_|  |___/\___|\___|\__|
   |
  \*/

  void
  ClothoidList::intersect_ISO(
    real_type            offs,
    ClothoidList const & CL,
    real_type            offs_CL,
    IntersectList      & ilist,
    bool                 swap_s_vals
  ) const {
    if ( intersect_with_AABBtree ) {
      this->build_AABBtree_ISO( offs );
      CL.build_AABBtree_ISO( offs_CL );
      AABBtree::VecPairPtrBBox iList;
      m_aabb_tree.intersect( CL.m_aabb_tree, iList );

      AABBtree::VecPairPtrBBox::const_iterator ip;
      for ( ip = iList.begin(); ip != iList.end(); ++ip ) {
        size_t ipos1 = size_t(ip->first->Ipos());
        size_t ipos2 = size_t(ip->second->Ipos());

        Triangle2D const & T1 = m_aabb_tri[ipos1];
        Triangle2D const & T2 = CL.m_aabb_tri[ipos2];

        ClothoidCurve const & C1 = m_clotoidList[T1.Icurve()];
        ClothoidCurve const & C2 = CL.m_clotoidList[T2.Icurve()];

        real_type ss1, ss2;
        bool converged = C1.aabb_intersect_ISO( T1, offs, &C2, T2, offs_CL, ss1, ss2 );

        if ( converged ) {
          ss1 += m_s0[T1.Icurve()];
          ss2 += CL.m_s0[T2.Icurve()];
          if ( swap_s_vals ) swap( ss1, ss2 );
          ilist.push_back( Ipair( ss1, ss2 ) );
        }
      }
    } else {
      bbTriangles_ISO( offs, m_aabb_tri, Utils::m_pi/18, 1e100 );
      CL.bbTriangles_ISO( offs_CL, CL.m_aabb_tri, Utils::m_pi/18, 1e100 );
      vector<Triangle2D>::const_iterator i1, i2;
      for ( i1 = m_aabb_tri.begin(); i1 != m_aabb_tri.end(); ++i1 ) {
        for ( i2 = CL.m_aabb_tri.begin(); i2 != CL.m_aabb_tri.end(); ++i2 ) {
          Triangle2D const & T1 = *i1;
          Triangle2D const & T2 = *i2;

          ClothoidCurve const & C1 = m_clotoidList[T1.Icurve()];
          ClothoidCurve const & C2 = CL.m_clotoidList[T2.Icurve()];

          real_type ss1, ss2;
          bool converged = C1.aabb_intersect_ISO( T1, offs, &C2, T2, offs_CL, ss1, ss2 );

          if ( converged ) {
            ss1 += m_s0[T1.Icurve()];
            ss2 += CL.m_s0[T2.Icurve()];
            if ( swap_s_vals ) swap( ss1, ss2 );
            ilist.push_back( Ipair( ss1, ss2 ) );
          }
        }
      }

    }
  }

  /*\
   |      _ _     _
   |   __| (_)___| |_ __ _ _ __   ___ ___
   |  / _` | / __| __/ _` | '_ \ / __/ _ \
   | | (_| | \__ \ || (_| | | | | (_|  __/
   |  \__,_|_|___/\__\__,_|_| |_|\___\___|
  \*/

  int_type
  ClothoidList::closestPoint_internal(
    real_type   qx,
    real_type   qy,
    real_type   offs,
    real_type & x,
    real_type & y,
    real_type & s,
    real_type & DST
  ) const {

    this->build_AABBtree_ISO( offs );

    AABBtree::VecPtrBBox candidateList;
    m_aabb_tree.min_distance( qx, qy, candidateList );
    AABBtree::VecPtrBBox::const_iterator ic;
    UTILS_ASSERT0(
      candidateList.size() > 0, "ClothoidList::closestPoint no candidate\n"
    );
    int_type icurve = 0;
    DST = numeric_limits<real_type>::infinity();
    for ( ic = candidateList.begin(); ic != candidateList.end(); ++ic ) {
      size_t ipos = size_t((*ic)->Ipos());
      Triangle2D const & T = m_aabb_tri[ipos];
      real_type dst = T.distMin( qx, qy );
      if ( dst < DST ) {
        // refine distance
        real_type xx, yy, ss;
        m_clotoidList[T.Icurve()].closestPoint_internal(
          T.S0(), T.S1(), qx, qy, offs, xx, yy, ss, dst
        );
        if ( dst < DST ) {
          DST    = dst;
          s      = ss + m_s0[T.Icurve()];
          x      = xx;
          y      = yy;
          icurve = T.Icurve();
        }
      }
    }
    return icurve;
  }

  // - - - - - - - - - - - - - - - - - - - - - - - - - - - - - - - - - - - - - -

  int_type
  ClothoidList::closestPoint_ISO(
    real_type   qx,
    real_type   qy,
    real_type   offs,
    real_type & x,
    real_type & y,
    real_type & s,
    real_type & t,
    real_type & DST
  ) const {

    int_type icurve = this->closestPoint_internal( qx, qy, offs, x, y, s, DST );

    // check if projection is orthogonal
    real_type nx, ny;
    m_clotoidList[icurve].nor_ISO( s - m_s0[icurve], nx, ny );
    real_type qxx = qx - x;
    real_type qyy = qy - y;
    t = qxx * nx + qyy * ny - offs; // signed distance
    real_type pt = abs(qxx * ny - qyy * nx);
    G2LIB_DEBUG_MESSAGE(
      "ClothoidList::closestPoint_ISO\n"
      "||P-P0|| = {} and {}, |(P-P0).T| = {}\n",
      DST, hypot(qxx,qyy), pt
    );
    return pt > GLIB2_TOL_ANGLE*hypot(qxx,qyy) ? -(icurve+1) : icurve;
  }

  // - - - - - - - - - - - - - - - - - - - - - - - - - - - - - - - - - - - - - -

  int_type
  ClothoidList::closestPoint_ISO(
    real_type   qx,
    real_type   qy,
    real_type & x,
    real_type & y,
    real_type & s,
    real_type & t,
    real_type & dst
  ) const {
    return closestPoint_ISO( qx, qy, 0, x, y, s, t, dst );
  }

  /*\
   |      _ _     _
   |   __| (_)___| |_ __ _ _ __   ___ ___
   |  / _` | / __| __/ _` | '_ \ / __/ _ \
   | | (_| | \__ \ || (_| | | | | (_|  __/
   |  \__,_|_|___/\__\__,_|_| |_|\___\___|
  \*/

  int_type
  ClothoidList::closestSegment( real_type qx, real_type qy ) const {
    this->build_AABBtree_ISO( 0 );

    AABBtree::VecPtrBBox candidateList;
    m_aabb_tree.min_distance( qx, qy, candidateList );
    AABBtree::VecPtrBBox::const_iterator ic;
    UTILS_ASSERT0(
      candidateList.size() > 0, "ClothoidList::closestSegment no candidate\n"
    );
    int_type icurve = 0;
    real_type DST = numeric_limits<real_type>::infinity();
    for ( ic = candidateList.begin(); ic != candidateList.end(); ++ic ) {
      size_t ipos = size_t((*ic)->Ipos());
      Triangle2D const & T = m_aabb_tri[ipos];
      real_type dst = T.distMin( qx, qy );
      if ( dst < DST ) {
        // refine distance
        real_type xx, yy, ss;
        m_clotoidList[T.Icurve()].closestPoint_internal(
          T.S0(), T.S1(), qx, qy, 0, xx, yy, ss, dst
        );
        if ( dst < DST ) {
          DST    = dst;
          icurve = T.Icurve();
        }
      }
    }
    return icurve;
  }

  // - - - - - - - - - - - - - - - - - - - - - - - - - - - - - - - - - - - - - -

  int_type
  ClothoidList::closestPointInRange_ISO(
    real_type   qx,
    real_type   qy,
    int_type    icurve_begin,
    int_type    icurve_end,
    real_type & x,
    real_type & y,
    real_type & s,
    real_type & t,
    real_type & dst,
    int_type  & icurve
  ) const {
    UTILS_ASSERT0(
      !m_clotoidList.empty(),
      "ClothoidList::closestPointInRange_ISO, empty list\n"
    );
    int_type nsegs = this->numSegments();
    if ( nsegs == 1 ) { // only 1 segment to check
      icurve = 0;
      int_type res = m_clotoidList.front().closestPoint_ISO( qx, qy, x, y, s, t, dst );
      s += m_s0[0];
      return res;
    }

    int_type ib = icurve_begin % nsegs; // to avoid infinite loop in case of bad input
    int_type ie = icurve_end   % nsegs; // to avoid infinite loop in case of bad input
    if ( ib < 0 ) ib += nsegs;
    if ( ie < 0 ) ie += nsegs;
    UTILS_ASSERT(
      ib >= 0 && ie >= 0,
      "ClothoidList::closestPointInRange_ISO, ib = {} ie = {}\n",
      ib, ie
    );

    icurve = ib;
    int_type res = m_clotoidList[icurve].closestPoint_ISO( qx, qy, x, y, s, t, dst );
    s += m_s0[icurve];

    G2LIB_DEBUG_MESSAGE(
      "ClothoidList::closestPointInRange_ISO\n"
      "first segment #{} dst = {} res = {}\n",
      icurve, dst, res
    );

    if ( ib == ie ) return res; // only one segment to check

    int_type iseg = ib;
    do {
      if ( ++iseg >= nsegs ) iseg -= nsegs; // next segment
      real_type C_x, C_y, C_s, C_t, C_dst;
      int_type C_res = m_clotoidList[iseg].closestPoint_ISO(
        qx, qy, C_x, C_y, C_s, C_t, C_dst
      );
      G2LIB_DEBUG_MESSAGE(
        "ClothoidList::closestPointInRange_ISO: segment #{} dst = {} res = {}\n",
        iseg, C_dst, C_res
      );
      if ( C_dst < dst ) {
        dst    = C_dst;
        x      = C_x;
        y      = C_y;
        s      = C_s + m_s0[iseg];
        t      = C_t;
        icurve = iseg;
        res    = C_res;
        G2LIB_DEBUG_MESSAGE(
          "ClothoidList::closestPointInRange_ISO, new min at s = {}, res = {}\n", s, res
        );
      }
    } while ( iseg != ie );
    return res;
  }

  // - - - - - - - - - - - - - - - - - - - - - - - - - - - - - - - - - - - - - -

  int_type
  ClothoidList::closestPointInSRange_ISO(
    real_type   qx,
    real_type   qy,
    real_type   s_begin,
    real_type   s_end,
    real_type & x,
    real_type & y,
    real_type & s,
    real_type & t,
    real_type & dst,
    int_type  & icurve
  ) const {
    UTILS_ASSERT0(
      !m_clotoidList.empty(),
      "ClothoidList::closestPointInSRange_ISO, empty list\n"
    );
    // put in range
    while ( s_begin < 0              ) s_begin += this->length();
    while ( s_begin > this->length() ) s_begin -= this->length();
    while ( s_end   < 0              ) s_end   += this->length();
    while ( s_end   > this->length() ) s_end   -= this->length();

    // get initial and final segment
    int_type i_begin = findAtS( s_begin );
    int_type i_end   = findAtS( s_end );
    int_type res     = 0;
    if ( i_begin == i_end ) {
      // stesso segmento
      real_type     ss0 = m_s0[i_begin];
      ClothoidCurve C   = m_clotoidList[i_begin]; // crea copia
      C.trim( s_begin-ss0, s_end-ss0 );
      res = C.closestPoint_ISO( qx, qy, x, y, s, t, dst );
      s += s_begin;
    } else {
      // segmenti consecutivi
      int_type  res1;
      real_type x1, y1, s1, t1, dst1;

      real_type     ss0 = m_s0[i_begin];
      real_type     ss1 = m_s0[i_end];
      ClothoidCurve C0  = m_clotoidList[i_begin]; // crea copia
      ClothoidCurve C1  = m_clotoidList[i_end];   // crea copia

      // taglia il segmento
      C0.trim( s_begin-ss0, C0.length() );

      // calcolo closest point
      res = C0.closestPoint_ISO( qx, qy, x, y, s, t, dst );
      s  += s_begin;
      icurve = i_begin;

      G2LIB_DEBUG_MESSAGE(
        "ClothoidList::closestPointInSRange_ISO: first segment {} dst = {} res = {}\n",
        i_begin, dst, res
      );

      C1.trim( 0, s_end-ss1 );
      res1 = C1.closestPoint_ISO( qx, qy, x1, y1, s1, t1, dst1 );
      s1 += ss1;

      G2LIB_DEBUG_MESSAGE(
        "ClothoidList::closestPointInSRange_ISO: last segment {} dst = {} res = {}\n",
        i_end, dst1, res1
      );

      if ( dst1 < dst ) {
        x = x1; y = y1; s = s1; t = t1;
        dst = dst1; res = res1; icurve = i_end;
      }

      // ci sono altri segmenti?
      if ( i_end < i_begin ) i_end += int_type(m_clotoidList.size());
      ++i_begin;
      if ( i_begin < i_end ) {
        int_type icurve1;
        res1 = this->closestPointInRange_ISO(
          qx, qy, i_begin, i_end, x1, y1, s1, t1, dst1, icurve1
        );
        G2LIB_DEBUG_MESSAGE(
          "ClothoidList::closestPointInSRange_ISO: range [{},{}] dst = {} res = {}\n",
          i_begin, i_end, dst1, res1
        );
        if ( dst1 < dst ) {
          x      = x1;
          y      = y1;
          s      = s1;
          t      = t1;
          dst    = dst1;
          res    = res1;
          icurve = icurve1;
          G2LIB_DEBUG_MESSAGE(
            "ClothoidList::closestPointInSRange_ISO: new min at s = {}, res = {}\n", s, res
          );
        }
      }
    }
    return res;
  }

  // - - - - - - - - - - - - - - - - - - - - - - - - - - - - - - - - - - - - - -
  // - - - - - - - - - - - - - - - - - - - - - - - - - - - - - - - - - - - - - -
  // - - - - - - - - - - - - - - - - - - - - - - - - - - - - - - - - - - - - - -
  // - - - - - - - - - - - - - - - - - - - - - - - - - - - - - - - - - - - - - -

  void
  ClothoidList::getSK( real_type * s, real_type * kappa ) const {
    vector<ClothoidCurve>::const_iterator ic = m_clotoidList.begin();
    int_type  k  = 0;
    real_type ss = 0;
    while ( ic != m_clotoidList.end() ) {
      s[k]     = ss;
      kappa[k] = ic->kappaBegin();
      ss      += ic->length();
      ++k;
      ++ic;
    }
    --ic; // last element
    s[k]     = ss;
    kappa[k] = ic->kappaEnd();
  }

  // - - - - - - - - - - - - - - - - - - - - - - - - - - - - - - - - - - - - - -
  // - - - - - - - - - - - - - - - - - - - - - - - - - - - - - - - - - - - - - -
  // - - - - - - - - - - - - - - - - - - - - - - - - - - - - - - - - - - - - - -
  // - - - - - - - - - - - - - - - - - - - - - - - - - - - - - - - - - - - - - -

  void
  ClothoidList::getSTK(
    real_type * s,
    real_type * theta,
    real_type * kappa
  ) const {
    vector<ClothoidCurve>::const_iterator ic = m_clotoidList.begin();
    int_type  k  = 0;
    real_type ss = 0;
    while ( ic != m_clotoidList.end() ) {
      s[k]     = ss;
      theta[k] = ic->thetaBegin();
      kappa[k] = ic->kappaBegin();
      ss      += ic->length();
      ++k;
      ++ic;
    }
    --ic; // last element
    s[k]     = ss;
    theta[k] = ic->thetaEnd();
    kappa[k] = ic->kappaEnd();
  }

  // - - - - - - - - - - - - - - - - - - - - - - - - - - - - - - - - - - - - - -

  void
  ClothoidList::getXY( real_type * x, real_type * y ) const {
    vector<ClothoidCurve>::const_iterator ic = m_clotoidList.begin();
    int_type k  = 0;
    while ( ic != m_clotoidList.end() ) {
      x[k] = ic->xBegin();
      y[k] = ic->yBegin();
      ++k; ++ic;
    }
    --ic;
    x[k] = ic->xEnd();
    y[k] = ic->yEnd();
  }

  // - - - - - - - - - - - - - - - - - - - - - - - - - - - - - - - - - - - - - -

  void
  ClothoidList::getDeltaTheta( real_type * deltaTheta ) const {
    vector<ClothoidCurve>::const_iterator ic = m_clotoidList.begin();
    int_type k = 0;
    for ( ++ic; ic != m_clotoidList.end(); ++ic, ++k ) {
      real_type tmp = ic->thetaBegin()-ic[-1].thetaEnd();
      if      ( tmp >  Utils::m_pi ) tmp -= Utils::m_2pi;
      else if ( tmp < -Utils::m_pi ) tmp += Utils::m_2pi;
      deltaTheta[k] = tmp;
    }
  }

  // - - - - - - - - - - - - - - - - - - - - - - - - - - - - - - - - - - - - - -

  void
  ClothoidList::getDeltaKappa( real_type * deltaKappa ) const {
    vector<ClothoidCurve>::const_iterator ic = m_clotoidList.begin();
    int_type k = 0;
    for ( ++ic; ic != m_clotoidList.end(); ++ic, ++k  )
      deltaKappa[k] = ic->kappaBegin()-ic[-1].kappaEnd();

  }

  // - - - - - - - - - - - - - - - - - - - - - - - - - - - - - - - - - - - - - -

  int_type
  ClothoidList::findST1(
    real_type   x,
    real_type   y,
    real_type & s,
    real_type & t
  ) const {

    UTILS_ASSERT0( !m_clotoidList.empty(), "ClothoidList::findST, empty list\n" );
    vector<ClothoidCurve>::const_iterator ic = m_clotoidList.begin();
    vector<real_type>::const_iterator     is = m_s0.begin();

    s = t = 0;
    int_type  ipos = 0;
    int_type  iseg = 0;
    real_type S, T;
    bool ok = ic->findST_ISO( x, y, S, T );
    if ( ok ) {
      s    = *is + S;
      t    = T;
      iseg = 0;
    }

    for ( ++ic, ++is, ++ipos;
          ic != m_clotoidList.end();
          ++ic, ++is, ++ipos ) {
      bool ok1 = ic->findST_ISO( x, y, S, T );
      if ( ok && ok1 ) ok1 = abs(T) < abs(t);
      if ( ok1 ) {
        ok   = true;
        s    = *is + S;
        t    = T;
        iseg = ipos;
      }
    }

    return ok ? iseg : -(1+iseg);
  }

  // - - - - - - - - - - - - - - - - - - - - - - - - - - - - - - - - - - - - - -

  int_type
  ClothoidList::findST1(
    int_type    ibegin,
    int_type    iend,
    real_type   x,
    real_type   y,
    real_type & s,
    real_type & t
  ) const {

    UTILS_ASSERT0(
      !m_clotoidList.empty(),
      "ClothoidList::findST, empty list\n"
    );
    UTILS_ASSERT(
      ibegin >= 0 && ibegin <= iend && iend < int_type(m_clotoidList.size()),
      "ClothoidList::findST( ibegin={}, iend={}, x, y, s, t ) bad range not in [0,{}]\n",
      ibegin, iend, m_clotoidList.size()-1
    );
    s = t = 0;
    int_type iseg = 0;
    bool ok = false;
    for ( int_type k = ibegin; k <= iend; ++k ) {
      ClothoidCurve const & ck = m_clotoidList[k];
      real_type S, T;
      bool ok1 = ck.findST_ISO( x, y, S, T );
      if ( ok && ok1 ) ok1 = abs(T) < abs(t);
      if ( ok1 ) {
        ok   = true;
        s    = m_s0[k] + S;
        t    = T;
        iseg = k;
      }
    }
    return ok ? iseg : -(1+iseg);
  }

  // - - - - - - - - - - - - - - - - - - - - - - - - - - - - - - - - - - - - - -

  void
  ClothoidList::export_table( ostream_type & stream ) const {
    stream << "x\ty\ttheta0\tkappa0\tdkappa\tL\n";
    vector<ClothoidCurve>::const_iterator ic = m_clotoidList.begin();
    for (; ic != m_clotoidList.end(); ++ic )
      stream
        << ic->xBegin()     << '\t'
        << ic->yBegin()     << '\t'
        << ic->thetaBegin() << '\t'
        << ic->kappaBegin() << '\t'
        << ic->dkappa()     << '\t'
        << ic->length()     << '\n';
  }

  // - - - - - - - - - - - - - - - - - - - - - - - - - - - - - - - - - - - - - -

  void
  ClothoidList::export_ruby( ostream_type & stream ) const {
    stream << "data = {\n";
    vector<ClothoidCurve>::const_iterator ic = m_clotoidList.begin();
    for (; ic != m_clotoidList.end(); ++ic )
      stream
        << ic->xBegin()     << '\t'
        << ic->yBegin()     << '\t'
        << ic->thetaBegin() << '\t'
        << ic->kappaBegin() << '\t'
        << ic->dkappa()     << '\t'
        << ic->length()     << '\n';
    stream << "}\n";
  }

  // - - - - - - - - - - - - - - - - - - - - - - - - - - - - - - - - - - - - - -

  ostream_type &
  operator << ( ostream_type & stream, ClothoidList const & CL ) {
    vector<ClothoidCurve>::const_iterator ic = CL.m_clotoidList.begin();
    for (; ic != CL.m_clotoidList.end(); ++ic )
      stream << *ic << '\n';
    return stream;
  }

  // - - - - - - - - - - - - - - - - - - - - - - - - - - - - - - - - - - - - - -

  #ifndef DOXYGEN_SHOULD_SKIP_THIS
  static
  void
  save_segment( ostream_type & stream, ClothoidCurve const & c ) {
    fmt::print( stream,
      "{:<24}\t{:<24}\t{:<24}\t{:<24}\n"
      "{:<24}\t{:<24}\t{:<24}\t{:<24}\n",
      //------------------
      fmt::format("{:.20}",c.xBegin()),
      fmt::format("{:.20}",c.yBegin()),
      fmt::format("{:.20}",c.thetaBegin()),
      fmt::format("{:.20}",c.kappaBegin()),
      //------------------
      fmt::format("{:.20}",c.xEnd()),
      fmt::format("{:.20}",c.yEnd()),
      fmt::format("{:.20}",c.thetaEnd()),
      fmt::format("{:.20}",c.kappaEnd())
    );
  }
  #endif

  // - - - - - - - - - - - - - - - - - - - - - - - - - - - - - - - - - - - - - -

  #ifndef DOXYGEN_SHOULD_SKIP_THIS
  static
  bool
  load_segment(
    istream_type  & stream,
    ClothoidCurve & c,
    real_type       epsi
  ) {
    string line1, line2;
    while ( stream.good() ) {
      if ( !getline(stream,line1) ) return false;
      if ( line1[0] != '#' ) break;
    }
    if ( !stream.good() ) return false;
    while ( stream.good() ) {
      if ( !getline(stream,line2) ) return false;
      if ( line2[0] != '#' ) break;
    }
    if ( !stream.good() ) return false;
    std::istringstream iss1(line1);
    std::istringstream iss2(line2);
    real_type x0, y0, x1, y1, theta0, theta1, kappa0, kappa1;
    iss1 >> x0 >> y0 >> theta0 >> kappa0;
    iss2 >> x1 >> y1 >> theta1 >> kappa1;
    c.build_G1( x0, y0, theta0, x1, y1, theta1 );
    // check segment
    real_type err1 = std::abs( kappa0 - c.kappaBegin() ) * c.length();
    real_type err2 = std::abs( kappa1 - c.kappaEnd() ) * c.length();
    UTILS_ASSERT(
      err1 < epsi && err2 < epsi,
      "load_segment, failed tolerance on curvature\n"
      "begin error = {}, end error = {}\n",
      err1, err2
    );
    return true;
  }
  #endif

  // - - - - - - - - - - - - - - - - - - - - - - - - - - - - - - - - - - - - - -

  void
  ClothoidList::save( ostream_type & stream ) const {
    vector<ClothoidCurve>::const_iterator ic = m_clotoidList.begin();
    stream << "# x y theta kappa\n";
    for ( int_type nseg = 1; ic != m_clotoidList.end(); ++ic, ++nseg ) {
      stream << "# segment n." << nseg << '\n';
      save_segment( stream, *ic );
    }
    stream << "# EOF\n";
  }

  // - - - - - - - - - - - - - - - - - - - - - - - - - - - - - - - - - - - - - -

  void
  ClothoidList::load( istream_type & stream, real_type epsi ) {
    this->init();
    while ( stream.good() ) {
      ClothoidCurve c;
      bool ok = load_segment( stream, c, epsi );
      if ( !ok ) break;
      this->push_back( c );
    }
  }

  // - - - - - - - - - - - - - - - - - - - - - - - - - - - - - - - - - - - - - -

  void
  G2solveCLC::save( ostream_type & stream ) const {
    stream << "# x y theta kappa\n";
    save_segment( stream, S0 );
    save_segment( stream, SM );
    save_segment( stream, S1 );
    stream << "# EOF\n";
  }

  // - - - - - - - - - - - - - - - - - - - - - - - - - - - - - - - - - - - - - -

  void
  G2solve3arc::save( ostream_type & stream ) const {
    stream << "# x y theta kappa\n";
    save_segment( stream, S0 );
    save_segment( stream, SM );
    save_segment( stream, S1 );
    stream << "# EOF\n";
  }

  // - - - - - - - - - - - - - - - - - - - - - - - - - - - - - - - - - - - - - -

}

// EOF: ClothoidList.cc
```

### Quick search

### Table of Contents

- Matlab Interface Manual
- C++ API
- MATLAB API

«
hide menu

menu
sidebar
»

### Navigation

- index
- toc
- Clothoids »
- Program Listing for File ClothoidList.cc

© Copyright 2021, Enrico Bertolazzi and Marco Frego.
Created using Sphinx 4.2.0.
