# Supplementary material for: Smooth Interpolating Curves with Local Control and Monotone Alternating Curvature
Source: Comput Graph Forum. 2022 Oct 6;41(5):25–38. doi: 10.1111/cgf.14600 (PMC9827861; doi:10.1111/cgf.14600)
Supplement: Supplementary file 1 — Supplement Material [file CGF-41-25-s001.zip › Local-Smooth-Interpolating-MonoCurvature/extern/clothoids/docs/api-cpp/program_listing_file_Clothoids.hh.html]

Program Listing for File Clothoids.hh — Clothoids v2.0.9

### Navigation

- index
- toc
- Clothoids »
- Program Listing for File Clothoids.hh

# Program Listing for File Clothoids.hh¶

↰ Return to documentation for file (`Clothoids.hh`)

```
/*--------------------------------------------------------------------------*\
 |                                                                          |
 |  Copyright (C) 2018                                                      |
 |                                                                          |
 |         , __                 , __                                        |
 |        /|/  \               /|/  \                                       |
 |         | __/ _   ,_         | __/ _   ,_                                |
 |         |   \|/  /  |  |   | |   \|/  /  |  |   |                        |
 |         |(__/|__/   |_/ \_/|/|(__/|__/   |_/ \_/|/                       |
 |                           /|                   /|                        |
 |                           \|                   \|                        |
 |                                                                          |
 |      Paolo Bevilacqua and Enrico Bertolazzi                              |
 |                                                                          |
 |      (1) Dipartimento di Ingegneria e Scienza dell'Informazione          |
 |      (2) Dipartimento di Ingegneria Industriale                          |
 |                                                                          |
 |      Universita` degli Studi di Trento                                   |
 |      email: paolo.bevilacqua@unitn.it                                    |
 |      email: enrico.bertolazzi@unitn.it                                   |
 |                                                                          |
\*--------------------------------------------------------------------------*/


#pragma once

#ifndef CLOTHOIDS_dot_HH
#define CLOTHOIDS_dot_HH

#include "Utils.hh"

#include <string>
#include <fstream>
#include <iostream>
#include <iomanip>
#include <cmath>

#include <vector>
#include <map>
#include <utility>
#include <algorithm>
#include <iterator>

// check if compiler is C++11
#ifndef G2LIB_DO_NOT_USE_CXX11
  #define G2LIB_USE_CXX11
#endif

#ifdef G2LIB_USE_CXX11
#include <memory>  // shared_ptr
#endif

#ifdef G2LIB_DEBUG
  #define G2LIB_DEBUG_MESSAGE(...) std::cout << fmt::format(__VA_ARGS__) << std::flush
#else
  #define G2LIB_DEBUG_MESSAGE(...)
#endif

#ifndef GLIB2_TOL_ANGLE
  #define GLIB2_TOL_ANGLE 1e-8
#endif

namespace G2lib {
  typedef std::basic_istream<char> istream_type;
  typedef std::basic_ostream<char> ostream_type;
  typedef double real_type;
  typedef int    int_type;
}

#include "Clothoids/G2lib.hxx"
#include "Clothoids/Triangle2D.hxx"
#include "Clothoids/BaseCurve.hxx"
#include "Clothoids/AABBtree.hxx"
#include "Clothoids/Fresnel.hxx"
#include "Clothoids/Line.hxx"
#include "Clothoids/Circle.hxx"
#include "Clothoids/Biarc.hxx"
#include "Clothoids/Clothoid.hxx"
#include "Clothoids/PolyLine.hxx"
#include "Clothoids/BiarcList.hxx"
#include "Clothoids/ClothoidList.hxx"
#include "Clothoids/ClothoidAsyPlot.hxx"

#endif
```

### Quick search

### Table of Contents

- Matlab Interface Manual
- C++ API
- MATLAB API

«
hide menu

menu
sidebar
»

### Navigation

- index
- toc
- Clothoids »
- Program Listing for File Clothoids.hh

© Copyright 2021, Enrico Bertolazzi and Marco Frego.
Created using Sphinx 4.2.0.
