# Supplementary material for: Smooth Interpolating Curves with Local Control and Monotone Alternating Curvature
Source: Comput Graph Forum. 2022 Oct 6;41(5):25–38. doi: 10.1111/cgf.14600 (PMC9827861; doi:10.1111/cgf.14600)
Supplement: Supplementary file 1 — Supplement Material [file CGF-41-25-s001.zip › Local-Smooth-Interpolating-MonoCurvature/extern/clothoids/docs/api-cpp/program_listing_file_Clothoids_AABBtree.hxx.html]

Program Listing for File AABBtree.hxx — Clothoids v2.0.9

### Navigation

- index
- toc
- Clothoids »
- Program Listing for File AABBtree.hxx

# Program Listing for File AABBtree.hxx¶

↰ Return to documentation for file (`Clothoids/AABBtree.hxx`)

```
/*--------------------------------------------------------------------------*\
 |                                                                          |
 |  Copyright (C) 2018                                                      |
 |                                                                          |
 |         , __                 , __                                        |
 |        /|/  \               /|/  \                                       |
 |         | __/ _   ,_         | __/ _   ,_                                |
 |         |   \|/  /  |  |   | |   \|/  /  |  |   |                        |
 |         |(__/|__/   |_/ \_/|/|(__/|__/   |_/ \_/|/                       |
 |                           /|                   /|                        |
 |                           \|                   \|                        |
 |                                                                          |
 |      Paolo Bevilacqua and Enrico Bertolazzi                              |
 |                                                                          |
 |      (1) Dipartimento di Ingegneria e Scienza dell'Informazione          |
 |      (2) Dipartimento di Ingegneria Industriale                          |
 |                                                                          |
 |      Universita` degli Studi di Trento                                   |
 |      email: paolo.bevilacqua@unitn.it                                    |
 |      email: enrico.bertolazzi@unitn.it                                   |
 |                                                                          |
\*--------------------------------------------------------------------------*/


namespace G2lib {

  using std::setw;
  using std::vector;
  using std::pair;

  #ifdef G2LIB_USE_CXX11
  using std::make_shared;
  using std::shared_ptr; // promemoria shared_ptr<Foo>(&foo, [](void*){});
  #endif

  class AABBtree;

  /*\
   |   ____  ____
   |  | __ )| __ )  _____  __
   |  |  _ \|  _ \ / _ \ \/ /
   |  | |_) | |_) | (_) >  <
   |  |____/|____/ \___/_/\_\
  \*/
  class BBox {
  public:
    #ifdef G2LIB_USE_CXX11
    typedef shared_ptr<BBox const> PtrBBox;
    #else
    typedef BBox const * PtrBBox;
    #endif

  private:
    real_type m_xmin;
    real_type m_ymin;
    real_type m_xmax;
    real_type m_ymax;
    int_type  m_id;
    int_type  m_ipos;
    BBox();
    BBox( BBox const & );

  public:

    BBox(
      real_type xmin,
      real_type ymin,
      real_type xmax,
      real_type ymax,
      int_type  id,
      int_type  ipos
    ) {
      m_xmin = xmin;
      m_ymin = ymin;
      m_xmax = xmax;
      m_ymax = ymax;
      m_id   = id;
      m_ipos = ipos;
    }

    BBox(
      vector<PtrBBox> const & bboxes,
      int_type                id,
      int_type                ipos
    ) {
      m_id   = id;
      m_ipos = ipos;
      this -> join( bboxes );
    }

    // - - - - - - - - - - - - - - - - - - - - - - - - - - - - - - - - - - - - -

    real_type Xmin() const { return m_xmin; }
    real_type Ymin() const { return m_ymin; }
    real_type Xmax() const { return m_xmax; }
    real_type Ymax() const { return m_ymax; }

    int_type const & Id()   const { return m_id; }
    int_type const & Ipos() const { return m_ipos; }

    BBox const &
    operator = ( BBox const & rhs ) {
      m_xmin = rhs.m_xmin;
      m_ymin = rhs.m_ymin;
      m_xmax = rhs.m_xmax;
      m_ymax = rhs.m_ymax;
      m_id   = rhs.m_id;
      m_ipos = rhs.m_ipos;
      return *this;
    }

    bool
    collision( BBox const & box ) const {
      return !( (box.m_xmin > m_xmax ) ||
                (box.m_xmax < m_xmin ) ||
                (box.m_ymin > m_ymax ) ||
                (box.m_ymax < m_ymin ) );
    }

    void
    join( vector<PtrBBox> const & bboxes );

    real_type
    distance( real_type x, real_type y ) const;

    real_type
    maxDistance( real_type x, real_type y ) const;

    void
    print( ostream_type & stream ) const {
      fmt::print( stream,
        "BBOX (xmin,ymin,xmax,ymax) = ( {}, {}, {}, {} )\n",
        m_xmin, m_ymin, m_xmax, m_ymax
      );
    }

    friend class AABBtree;
  };

  inline
  ostream_type &
  operator << ( ostream_type & stream, BBox const & bb ) {
    bb.print(stream);
    return stream;
  }

  /*\
   |      _        _    ____  ____  _
   |     / \      / \  | __ )| __ )| |_ _ __ ___  ___
   |    / _ \    / _ \ |  _ \|  _ \| __| '__/ _ \/ _ \
   |   / ___ \  / ___ \| |_) | |_) | |_| | |  __/  __/
   |  /_/   \_\/_/   \_\____/|____/ \__|_|  \___|\___|
  \*/
  class AABBtree {
  public:

  #ifdef G2LIB_USE_CXX11
    typedef shared_ptr<BBox const> PtrBBox;
    typedef shared_ptr<AABBtree>   PtrAABB;
  #else
    typedef BBox const *           PtrBBox;
    typedef AABBtree *             PtrAABB;
  #endif

  typedef pair<PtrBBox,PtrBBox> PairPtrBBox;
  typedef vector<PtrBBox>       VecPtrBBox;
  typedef vector<PairPtrBBox>   VecPairPtrBBox;

  private:

    // bbox of the tree
    PtrBBox         pBBox;
    vector<PtrAABB> children;

    AABBtree( AABBtree const & tree );

    static
    real_type
    min_maxdist(
      real_type        x,
      real_type        y,
      AABBtree const & tree,
      real_type        mmDist
    );

    static
    void
    min_maxdist_select(
      real_type        x,
      real_type        y,
      real_type        mmDist,
      AABBtree const & tree,
      VecPtrBBox     & candidateList
    );

  public:

    AABBtree();

    ~AABBtree();

    // - - - - - - - - - - - - - - - - - - - - - - - - - - - - - - - - - - - - -

    void clear();

    bool empty() const;

    void
    bbox(
      real_type & xmin,
      real_type & ymin,
      real_type & xmax,
      real_type & ymax
    ) const {
      xmin = pBBox->m_xmin;
      ymin = pBBox->m_ymin;
      xmax = pBBox->m_xmax;
      ymax = pBBox->m_ymax;
    }

    void
    build( vector<PtrBBox> const & bboxes );

    void
    print( ostream_type & stream, int level = 0 ) const;

    template <typename COLLISION_fun>
    bool
    collision(
      AABBtree const & tree,
      COLLISION_fun    ifun,
      bool             swap_tree = false
    ) const {

      // check bbox with
      if ( !tree.pBBox->collision(*pBBox) ) return false;

      int icase = (children.empty() ? 0 : 1) +
                  (tree.children.empty()? 0 : 2);

      switch ( icase ) {
      case 0: // both leaf, use GeomPrimitive intersection algorithm
        if ( swap_tree ) return ifun( tree.pBBox, pBBox );
        else             return ifun( pBBox, tree.pBBox );
      case 1: // first is a tree, second is a leaf
        { typename vector<PtrAABB>::const_iterator it;
          for ( it = children.begin(); it != children.end(); ++it )
            if ( tree.collision( **it, ifun, !swap_tree ) )
              return true;
        }
        break;
      case 2: // first leaf, second is a tree
        { typename vector<PtrAABB>::const_iterator it;
          for ( it = tree.children.begin();
                it != tree.children.end(); ++it )
            if ( this->collision( **it, ifun, swap_tree ) )
              return true;
        }
        break;
      case 3: // first is a tree, second is a tree
        { typename vector<PtrAABB>::const_iterator c1;
          typename vector<PtrAABB>::const_iterator c2;
          for ( c1 = children.begin(); c1 != children.end(); ++c1 )
            for ( c2 = tree.children.begin();
                  c2 != tree.children.end(); ++c2 )
              if ( (*c1)->collision( **c2, ifun, swap_tree ) )
                return true;
        }
        break;
      }
      return false;
    }

    void
    intersect(
      AABBtree const & tree,
      VecPairPtrBBox & intersectionList,
      bool             swap_tree = false
    ) const;

    void
    min_distance(
      real_type    x,
      real_type    y,
      VecPtrBBox & candidateList
    ) const;

  };

}
```

### Quick search

### Table of Contents

- Matlab Interface Manual
- C++ API
- MATLAB API

«
hide menu

menu
sidebar
»

### Navigation

- index
- toc
- Clothoids »
- Program Listing for File AABBtree.hxx

© Copyright 2021, Enrico Bertolazzi and Marco Frego.
Created using Sphinx 4.2.0.
