# Supplementary material for: Smooth Interpolating Curves with Local Control and Monotone Alternating Curvature
Source: Comput Graph Forum. 2022 Oct 6;41(5):25–38. doi: 10.1111/cgf.14600 (PMC9827861; doi:10.1111/cgf.14600)
Supplement: Supplementary file 1 — Supplement Material [file CGF-41-25-s001.zip › Local-Smooth-Interpolating-MonoCurvature/extern/clothoids/docs/api-cpp/program_listing_file_Clothoids_BaseCurve.hxx.html]

Program Listing for File BaseCurve.hxx — Clothoids v2.0.9

### Navigation

- index
- toc
- Clothoids »
- Program Listing for File BaseCurve.hxx

# Program Listing for File BaseCurve.hxx¶

↰ Return to documentation for file (`Clothoids/BaseCurve.hxx`)

```
/*--------------------------------------------------------------------------*\
 |                                                                          |
 |  Copyright (C) 2017                                                      |
 |                                                                          |
 |         , __                 , __                                        |
 |        /|/  \               /|/  \                                       |
 |         | __/ _   ,_         | __/ _   ,_                                |
 |         |   \|/  /  |  |   | |   \|/  /  |  |   |                        |
 |         |(__/|__/   |_/ \_/|/|(__/|__/   |_/ \_/|/                       |
 |                           /|                   /|                        |
 |                           \|                   \|                        |
 |                                                                          |
 |      Enrico Bertolazzi                                                   |
 |      Dipartimento di Ingegneria Industriale                              |
 |      Universita` degli Studi di Trento                                   |
 |      email: enrico.bertolazzi@unitn.it                                   |
 |                                                                          |
\*--------------------------------------------------------------------------*/


namespace G2lib {

  // - - - - - - - - - - - - - - - - - - - - - - - - - - - - - - - - - - - - - -
  /*\
   |  ____                  ____
   | | __ )  __ _ ___  ___ / ___|   _ _ ____   _____
   | |  _ \ / _` / __|/ _ \ |  | | | | '__\ \ / / _ \
   | | |_) | (_| \__ \  __/ |__| |_| | |   \ V /  __/
   | |____/ \__,_|___/\___|\____\__,_|_|    \_/ \___|
  \*/

  typedef enum {
    G2LIB_LINE=0,
    G2LIB_POLYLINE,
    G2LIB_CIRCLE,
    G2LIB_BIARC,
    G2LIB_BIARC_LIST,
    G2LIB_CLOTHOID,
    G2LIB_CLOTHOID_LIST
  } CurveType;

  extern char const *CurveType_name[];

  typedef std::pair<real_type,real_type> Ipair;
  typedef std::vector<Ipair>             IntersectList;

  /*\
   |   _       _                          _
   |  (_)_ __ | |_ ___ _ __ ___  ___  ___| |_
   |  | | '_ \| __/ _ \ '__/ __|/ _ \/ __| __|
   |  | | | | | ||  __/ |  \__ \  __/ (__| |_
   |  |_|_| |_|\__\___|_|  |___/\___|\___|\__|
  \*/

  class BaseCurve;

  bool
  collision( BaseCurve const & C1, BaseCurve const & C2 );

  bool
  collision_ISO(
    BaseCurve const & C1,
    real_type         offs_C1,
    BaseCurve const & C2,
    real_type         offs_C2
  );

  inline
  bool
  collision_SAE(
    BaseCurve const & C1,
    real_type         offs_C1,
    BaseCurve const & C2,
    real_type         offs_C2
  ) {
    return collision_ISO( C1, -offs_C1, C2, -offs_C2 );
  }

  #ifdef G2LIB_COMPATIBILITY_MODE
  inline
  bool
  collision(
    BaseCurve const & C1,
    real_type         offs_C1,
    BaseCurve const & C2,
    real_type         offs_C2
  ) {
    if ( G2lib::use_ISO ) return collision_ISO( C1, offs_C1, C2, offs_C2 );
    else                  return collision_SAE( C1, offs_C1, C2, offs_C2 );
  }
  #endif

  void
  intersect(
    BaseCurve const & C1,
    BaseCurve const & C2,
    IntersectList   & ilist,
    bool              swap_s_vals
  );

  void
  intersect_ISO(
    BaseCurve const & C1,
    real_type         offs_C1,
    BaseCurve const & C2,
    real_type         offs_C2,
    IntersectList   & ilist,
    bool              swap_s_vals
  );

  inline
  void
  intersect_SAE(
    BaseCurve const & C1,
    real_type         offs_C1,
    BaseCurve const & C2,
    real_type         offs_C2,
    IntersectList   & ilist,
    bool              swap_s_vals
  ) {
    intersect_ISO( C1, -offs_C1, C2, -offs_C2, ilist, swap_s_vals );
  }

  #ifdef G2LIB_COMPATIBILITY_MODE
  inline
  void
  intersect(
    BaseCurve const & C1,
    real_type         offs_C1,
    BaseCurve const & C2,
    real_type         offs_C2,
    IntersectList   & ilist,
    bool              swap_s_vals
  ) {
    if ( G2lib::use_ISO ) intersect_ISO( C1, offs_C1, C2, offs_C2, ilist, swap_s_vals );
    else                  intersect_SAE( C1, offs_C1, C2, offs_C2, ilist, swap_s_vals );
  }
  #endif

  class BaseCurve {

    // block default constructor
    BaseCurve( BaseCurve const & ) = delete;
    BaseCurve const & operator = ( BaseCurve const & ) = delete;

  protected:
    CurveType m_type;

  public:

    BaseCurve( CurveType const & type )
    : m_type(type)
    {}

    virtual
    ~BaseCurve() {}

    CurveType type() const { return m_type; }

    // . . . . . . . . . . . . . . . . . . . . . . . . . . . . . . . . . . . . .

    virtual real_type length() const = 0;

    virtual real_type length_ISO( real_type offs ) const = 0;

    real_type
    length_SAE( real_type offs ) const
    { return this->length_ISO(-offs); }

    #ifdef G2LIB_COMPATIBILITY_MODE
    real_type
    length( real_type offs ) const
    { return G2lib::use_ISO ? this->length_ISO(offs) : this->length_SAE(offs); }
    #endif

    /*\
     |   _     _
     |  | |__ | |__   _____  __
     |  | '_ \| '_ \ / _ \ \/ /
     |  | |_) | |_) | (_) >  <
     |  |_.__/|_.__/ \___/_/\_\
    \*/

    virtual
    void
    bbox(
      real_type & xmin,
      real_type & ymin,
      real_type & xmax,
      real_type & ymax
    ) const = 0;

    virtual
    void
    bbox_ISO(
      real_type   offs,
      real_type & xmin,
      real_type & ymin,
      real_type & xmax,
      real_type & ymax
    ) const = 0;

    void
    bbox_SAE(
      real_type   offs,
      real_type & xmin,
      real_type & ymin,
      real_type & xmax,
      real_type & ymax
    ) const {
      this->bbox_ISO( -offs, xmin, ymin, xmax, ymax );
    }

    #ifdef G2LIB_COMPATIBILITY_MODE
    void
    bbox(
      real_type   offs,
      real_type & xmin,
      real_type & ymin,
      real_type & xmax,
      real_type & ymax
    ) const {
      if ( G2lib::use_ISO ) this->bbox_ISO( offs, xmin, ymin, xmax, ymax );
      else                  this->bbox_SAE( offs, xmin, ymin, xmax, ymax );
    }
    #endif

    /*\
     |   _    _   _____    _                _
     |  | |__| |_|_   _| _(_)__ _ _ _  __ _| |___
     |  | '_ \ '_ \| || '_| / _` | ' \/ _` | / -_)
     |  |_.__/_.__/|_||_| |_\__,_|_||_\__, |_\___|
     |                                |___/
    \*/

    virtual
    void
    bbTriangles(
      std::vector<Triangle2D> & tvec,
      real_type                 max_angle = Utils::m_pi/18,
      real_type                 max_size  = 1e100,
      int_type                  icurve    = 0
    ) const = 0;

    virtual
    void
    bbTriangles_ISO(
      real_type                 offs,
      std::vector<Triangle2D> & tvec,
      real_type                 max_angle = Utils::m_pi/18,
      real_type                 max_size  = 1e100,
      int_type                  icurve    = 0
    ) const = 0;

    virtual
    void
    bbTriangles_SAE(
      real_type                 offs,
      std::vector<Triangle2D> & tvec,
      real_type                 max_angle = Utils::m_pi/18,
      real_type                 max_size  = 1e100,
      int_type                  icurve    = 0
    ) const = 0;

    /*\
     |   ____             _          _______           _
     |  | __ )  ___  __ _(_)_ __    / / ____|_ __   __| |
     |  |  _ \ / _ \/ _` | | '_ \  / /|  _| | '_ \ / _` |
     |  | |_) |  __/ (_| | | | | |/ / | |___| | | | (_| |
     |  |____/ \___|\__, |_|_| |_/_/  |_____|_| |_|\__,_|
     |              |___/
    \*/

    virtual real_type thetaBegin() const { return this->theta(0); }

    virtual real_type thetaEnd() const { return this->theta(this->length()); }

    virtual real_type kappaBegin() const { return this->kappa(0); }

    virtual real_type kappaEnd() const { return this->kappa(this->length()); }

    virtual real_type xBegin() const { return this->X(0); }

    virtual real_type yBegin() const { return this->Y(0); }

    virtual real_type xEnd() const { return this->X(this->length()); }

    virtual real_type yEnd() const { return this->Y(this->length()); }

    virtual real_type xBegin_ISO( real_type offs ) const { return this->X_ISO(0,offs); }

    virtual real_type yBegin_ISO( real_type offs ) const { return this->Y_ISO(0,offs); }

    virtual real_type xEnd_ISO( real_type offs ) const { return this->X_ISO(this->length(),offs); }

    virtual real_type yEnd_ISO( real_type offs ) const { return this->Y_ISO(this->length(),offs); }

    real_type xBegin_SAE( real_type offs ) const { return this->xBegin_ISO(-offs); }

    real_type yBegin_SAE( real_type offs ) const { return this->yBegin_ISO(-offs); }

    real_type xEnd_SAE( real_type offs ) const { return this->xEnd_ISO(-offs); }

    real_type yEnd_SAE( real_type offs ) const { return this->yEnd_ISO(-offs); }

    #ifdef G2LIB_COMPATIBILITY_MODE
    real_type
    xBegin( real_type offs ) const
    { return G2lib::use_ISO ? this->xBegin_ISO(offs) : this->xBegin_SAE(offs); }

    real_type
    yBegin( real_type offs ) const
    { return G2lib::use_ISO ? this->yBegin_ISO(offs) : this->yBegin_SAE(offs); }

    real_type
    xEnd( real_type offs ) const
    { return G2lib::use_ISO ? this->xEnd_ISO(offs) : this->xEnd_SAE(offs); }

    real_type
    yEnd( real_type offs ) const
    { return G2lib::use_ISO ? this->yEnd_ISO(offs) : this->yEnd_SAE(offs); }
    #endif

    virtual real_type tx_Begin() const { return this->tx(0); }

    virtual real_type ty_Begin() const { return this->ty(0); }

    virtual real_type tx_End() const { return this->tx(this->length()); }

    virtual real_type ty_End() const { return this->ty(this->length()); }

    virtual real_type nx_Begin_ISO() const { return -this->ty(0); }

    virtual real_type ny_Begin_ISO() const { return this->tx(0); }

    virtual real_type nx_End_ISO() const { return -this->ty(this->length()); }

    virtual real_type ny_End_ISO() const { return this->tx(this->length()); }

    real_type nx_Begin_SAE() const { return -nx_Begin_ISO(); }

    real_type ny_Begin_SAE() const { return -ny_Begin_ISO(); }

    real_type nx_End_SAE() const { return -nx_End_ISO(); }

    real_type ny_End_SAE() const { return -ny_End_ISO(); }

    #ifdef G2LIB_COMPATIBILITY_MODE
    real_type
    nx_Begin() const
    { return G2lib::use_ISO ? this->nx_Begin_ISO() : this->nx_Begin_SAE(); }

    real_type
    ny_Begin() const
    { return G2lib::use_ISO ? this->ny_Begin_ISO() : this->ny_Begin_SAE(); }

    real_type
    nx_End() const
    { return G2lib::use_ISO ? this->nx_End_ISO() : this->nx_End_SAE(); }

    real_type
    ny_End() const
    { return G2lib::use_ISO ? this->ny_End_ISO() : this->ny_End_SAE(); }
    #endif

    /*\
     |  _   _          _
     | | |_| |__   ___| |_ __ _
     | | __| '_ \ / _ \ __/ _` |
     | | |_| | | |  __/ || (_| |
     |  \__|_| |_|\___|\__\__,_|
    \*/

    virtual real_type theta( real_type s ) const = 0;

    virtual real_type theta_D( real_type s ) const = 0;

    virtual real_type theta_DD( real_type s ) const = 0;

    virtual real_type theta_DDD( real_type s ) const = 0;

    /*\
     |   _
     |  | | ____ _ _ __  _ __   __ _
     |  | |/ / _` | '_ \| '_ \ / _` |
     |  |   < (_| | |_) | |_) | (_| |
     |  |_|\_\__,_| .__/| .__/ \__,_|
     |            |_|   |_|
    \*/

    real_type kappa( real_type s ) const { return theta_D(s); }

    real_type kappa_D ( real_type s ) const { return theta_DD(s); }

    real_type kappa_DD( real_type s ) const { return theta_DDD(s); }

    /*\
     |  _____                   _   _   _
     | |_   _|   __ _ _ __   __| | | \ | |
     |   | |    / _` | '_ \ / _` | |  \| |
     |   | |   | (_| | | | | (_| | | |\  |
     |   |_|    \__,_|_| |_|\__,_| |_| \_|
    \*/

    virtual real_type tx( real_type s ) const;

    virtual real_type ty( real_type s ) const;

    virtual real_type tx_D( real_type s ) const;

    virtual real_type ty_D( real_type s ) const;

    virtual real_type tx_DD( real_type s ) const;

    virtual real_type ty_DD( real_type s ) const;

    virtual real_type tx_DDD( real_type s ) const;

    virtual real_type ty_DDD( real_type s ) const;

    // . . . . . . . . . . . . . . . . . . . . . . . . . . . . . . . . . . . . .
    real_type nx_ISO( real_type s ) const { return -ty(s); }

    real_type nx_ISO_D( real_type s ) const { return -ty_D(s); }

    real_type nx_ISO_DD( real_type s ) const { return -ty_DD(s); }

    real_type nx_ISO_DDD( real_type s ) const { return -ty_DDD(s); }

    real_type ny_ISO( real_type s ) const { return tx(s); }

    real_type ny_ISO_D( real_type s ) const { return tx_D(s); }

    real_type ny_ISO_DD( real_type s ) const { return tx_DD(s); }

    real_type ny_ISO_DDD( real_type s ) const { return tx_DDD(s); }

    real_type nx_SAE( real_type s ) const { return ty(s); }

    real_type nx_SAE_D( real_type s ) const { return ty_D(s); }

    real_type nx_SAE_DD( real_type s ) const { return ty_DD(s); }

    real_type nx_SAE_DDD( real_type s ) const { return ty_DDD(s); }

    real_type ny_SAE( real_type s ) const { return -tx(s); }

    real_type ny_SAE_D( real_type s ) const { return -tx_D(s); }

    real_type ny_SAE_DD ( real_type s ) const { return -tx_DD(s); }

    real_type ny_SAE_DDD( real_type s ) const { return -tx_DDD(s); }

    #ifdef G2LIB_COMPATIBILITY_MODE
    real_type nx( real_type s ) const
    { return G2lib::use_ISO ? this->nx_ISO(s) : this->nx_SAE(s); }

    real_type nx_D( real_type s ) const
    { return G2lib::use_ISO ? this->nx_ISO_D(s) : this->nx_SAE_D(s); }

    real_type nx_DD( real_type s ) const
    { return G2lib::use_ISO ? this->nx_ISO_DD(s) : this->nx_SAE_DD(s); }

    real_type nx_DDD( real_type s ) const
    { return G2lib::use_ISO ? this->nx_ISO_DDD(s) : this->nx_SAE_DDD(s); }

    real_type ny( real_type s ) const
    { return G2lib::use_ISO ? this->ny_ISO(s) : this->ny_SAE(s); }

    real_type ny_D( real_type s ) const
    { return G2lib::use_ISO ? this->ny_ISO_D(s) : this->ny_SAE_D(s); }

    real_type ny_DD( real_type s ) const
    { return G2lib::use_ISO ? this->ny_ISO_DD(s) : this->ny_SAE_DD(s); }

    real_type ny_DDD( real_type s ) const
    { return G2lib::use_ISO ? this->ny_ISO_DDD(s) : this->ny_SAE_DDD(s); }
    #endif

    // . . . . . . . . . . . . . . . . . . . . . . . . . . . . . . . . . . . . .

    virtual
    void
    tg( real_type s, real_type & tg_x, real_type & tg_y ) const {
      tg_x = this->tx(s);
      tg_y = this->ty(s);
    }

    virtual
    void
    tg_D( real_type s, real_type & tg_x_D, real_type & tg_y_D ) const {
      tg_x_D = this->tx_D(s);
      tg_y_D = this->ty_D(s);
    }

    virtual
    void
    tg_DD( real_type s, real_type & tg_x_DD, real_type & tg_y_DD ) const {
      tg_x_DD = this->tx_DD(s);
      tg_y_DD = this->ty_DD(s);
    }

    virtual
    void
    tg_DDD( real_type s, real_type & tg_x_DDD, real_type & tg_y_DDD ) const {
      tg_x_DDD = this->tx_DDD(s);
      tg_y_DDD = this->ty_DDD(s);
    }

    // . . . . . . . . . . . . . . . . . . . . . . . . . . . . . . . . . . . . .

    void
    nor_ISO( real_type s, real_type & nx, real_type & ny ) const
    { tg( s, ny, nx ); nx = -nx; }

    void
    nor_ISO_D( real_type s, real_type & nx_D, real_type & ny_D ) const
    { tg_D( s, ny_D, nx_D ); nx_D = -nx_D; }

    void
    nor_ISO_DD( real_type s, real_type & nx_DD, real_type & ny_DD ) const
    { tg_DD( s, ny_DD, nx_DD ); nx_DD = -nx_DD; }

    void
    nor_ISO_DDD( real_type s, real_type & nx_DDD, real_type & ny_DDD ) const
    { tg_DDD( s, ny_DDD, nx_DDD ); nx_DDD = -nx_DDD; }

    void
    nor_SAE( real_type s, real_type & nx, real_type & ny ) const
    { tg( s, ny, nx ); ny = -ny; }

    void
    nor_SAE_D( real_type s, real_type & nx_D, real_type & ny_D ) const
    { tg_D( s, ny_D, nx_D ); ny_D = -ny_D; }

    void
    nor_SAE_DD( real_type s, real_type & nx_DD, real_type & ny_DD ) const
    { tg_DD( s, ny_DD, nx_DD ); ny_DD = -ny_DD; }

    void
    nor_SAE_DDD( real_type s, real_type & nx_DDD, real_type & ny_DDD ) const
    { tg_DDD( s, ny_DDD, nx_DDD ); ny_DDD = -ny_DDD; }

    #ifdef G2LIB_COMPATIBILITY_MODE
    void
    nor( real_type s, real_type & nx, real_type & ny ) const {
      if ( G2lib::use_ISO ) this->nor_ISO(s,nx,ny);
      else                  this->nor_SAE(s,nx,ny);
    }

    void
    nor_D( real_type s, real_type & nx_D, real_type & ny_D ) const {
      if ( G2lib::use_ISO ) this->nor_ISO_D(s,nx_D,ny_D);
      else                  this->nor_SAE_D(s,nx_D,ny_D);
    }

    void
    nor_DD( real_type s, real_type & nx_DD, real_type & ny_DD ) const {
      if ( G2lib::use_ISO ) this->nor_ISO_DD(s,nx_DD,ny_DD);
      else                  this->nor_SAE_DD(s,nx_DD,ny_DD);
    }

    void
    nor_DDD( real_type s, real_type & nx_DDD, real_type & ny_DDD ) const {
      if ( G2lib::use_ISO ) this->nor_ISO_DDD(s,nx_DDD,ny_DDD);
      else                  this->nor_SAE_DDD(s,nx_DDD,ny_DDD);
    }
    #endif

    // . . . . . . . . . . . . . . . . . . . . . . . . . . . . . . . . . . . . .

    virtual
    void
    evaluate(
      real_type   s,
      real_type & th,
      real_type & k,
      real_type & x,
      real_type & y
    ) const {
      eval( s, x, y );
      th = theta( s );
      k  = theta_D( s );
    }

    virtual
    void
    evaluate_ISO(
      real_type   s,
      real_type   offs,
      real_type & th,
      real_type & k,
      real_type & x,
      real_type & y
    ) const {
      eval_ISO( s, offs, x, y );
      th = theta( s );
      k  = theta_D( s );
      k /= 1+offs*k; // scale curvature
    }

    virtual
    void
    evaluate_SAE(
      real_type   s,
      real_type   offs,
      real_type & th,
      real_type & k,
      real_type & x,
      real_type & y
    ) const {
      eval_SAE( s, offs, x, y );
      th = theta( s );
      k  = theta_D( s );
      k /= 1-offs*k; // scale curvature
    }

    #ifdef G2LIB_COMPATIBILITY_MODE
    void
    evaluate(
      real_type   s,
      real_type   offs,
      real_type & th,
      real_type & k,
      real_type & x,
      real_type & y
    ) const {
      if ( G2lib::use_ISO ) this->evaluate_ISO( s, offs, th, k, x, y );
      else                  this->evaluate_SAE( s, offs, th, k, x, y );
    }
    #endif

    // . . . . . . . . . . . . . . . . . . . . . . . . . . . . . . . . . . . . .

    virtual real_type X( real_type s ) const = 0;

    virtual real_type Y( real_type s ) const = 0;

    virtual real_type X_D( real_type s ) const = 0;

    virtual real_type Y_D( real_type s ) const = 0;

    virtual real_type X_DD( real_type s ) const = 0;

    virtual real_type Y_DD( real_type s ) const = 0;

    virtual real_type X_DDD( real_type s ) const = 0;

    virtual real_type Y_DDD( real_type s ) const = 0;

    // . . . . . . . . . . . . . . . . . . . . . . . . . . . . . . . . . . . . .

    virtual
    void
    eval( real_type s, real_type & x, real_type & y ) const = 0;

    virtual
    void
    eval_D( real_type s, real_type & x_D, real_type & y_D ) const = 0;

    virtual
    void
    eval_DD( real_type s, real_type & x_DD, real_type & y_DD ) const = 0;

    virtual
    void
    eval_DDD( real_type s, real_type & x_DDD, real_type & y_DDD ) const = 0;

    /*\
     |         __  __          _
     |   ___  / _|/ _|___  ___| |_
     |  / _ \| |_| |_/ __|/ _ \ __|
     | | (_) |  _|  _\__ \  __/ |_
     |  \___/|_| |_| |___/\___|\__|
    \*/

    virtual real_type X_ISO( real_type s, real_type offs ) const;

    virtual real_type Y_ISO( real_type s, real_type offs ) const;

    virtual real_type X_ISO_D( real_type s, real_type offs ) const;

    virtual real_type Y_ISO_D( real_type s, real_type offs ) const;

    virtual real_type X_ISO_DD( real_type s, real_type offs ) const;

    virtual real_type Y_ISO_DD( real_type s, real_type offs ) const;

    virtual real_type X_ISO_DDD( real_type s, real_type offs ) const;

    virtual real_type Y_ISO_DDD( real_type s, real_type offs ) const;

    real_type X_SAE( real_type s, real_type offs ) const { return this->X_ISO(s,-offs); }

    real_type Y_SAE( real_type s, real_type offs ) const { return this->Y_ISO(s,-offs); }

    real_type X_SAE_D( real_type s, real_type offs ) const { return this->X_ISO_D(s,-offs); }

    real_type Y_SAE_D( real_type s, real_type offs ) const { return this->Y_ISO_D(s,-offs); }

    real_type X_SAE_DD( real_type s, real_type offs ) const { return this->X_ISO_DD(s,-offs); }

    real_type Y_SAE_DD( real_type s, real_type offs ) const { return this->Y_ISO_DD(s,-offs); }

    real_type X_SAE_DDD( real_type s, real_type offs ) const { return this->X_ISO_DDD(s,-offs); }

    real_type Y_SAE_DDD( real_type s, real_type offs ) const { return this->Y_ISO_DDD(s,-offs); }

    #ifdef G2LIB_COMPATIBILITY_MODE
    real_type
    X( real_type s, real_type offs ) const
    { return G2lib::use_ISO ? this->X_ISO( s, offs ) : this->X_SAE( s, offs ); }

    real_type
    Y( real_type s, real_type offs ) const
    { return G2lib::use_ISO ? this->Y_ISO( s, offs ) : this->Y_SAE( s, offs ); }

    real_type
    X_D( real_type s, real_type offs ) const
    { return G2lib::use_ISO ? this->X_ISO_D( s, offs ) : this->X_SAE_D( s, offs ); }

    real_type
    Y_D( real_type s, real_type offs ) const
    { return G2lib::use_ISO ? this->Y_ISO_D( s, offs ) : this->Y_SAE_D( s, offs ); }

    real_type
    X_DD( real_type s, real_type offs ) const
    { return G2lib::use_ISO ? this->X_ISO_DD( s, offs ) : this->X_SAE_DD( s, offs ); }

    real_type
    Y_DD( real_type s, real_type offs ) const
    { return G2lib::use_ISO ? this->Y_ISO_DD( s, offs ) : this->Y_SAE_DD( s, offs ); }

    real_type
    X_DDD( real_type s, real_type offs ) const
    { return G2lib::use_ISO ? this->X_ISO_DDD( s, offs ) : this->X_SAE_DDD( s, offs ); }

    real_type
    Y_DDD( real_type s, real_type offs ) const
    { return G2lib::use_ISO ? this->Y_ISO_DDD( s, offs ) : this->Y_SAE_DDD( s, offs ); }
    #endif

    // . . . . . . . . . . . . . . . . . . . . . . . . . . . . . . . . . . . . .

    virtual
    void
    eval_ISO(
      real_type   s,
      real_type   offs,
      real_type & x,
      real_type & y
    ) const;

    void
    eval_SAE(
      real_type   s,
      real_type   offs,
      real_type & x,
      real_type & y
    ) const {
      this->eval_ISO( s, -offs, x, y );
    }

    #ifdef G2LIB_COMPATIBILITY_MODE
    void
    eval(
      real_type   s,
      real_type   offs,
      real_type & x,
      real_type & y
    ) const {
      if ( G2lib::use_ISO ) this->eval_ISO( s, offs, x, y );
      else                  this->eval_SAE( s, offs, x, y );
    }
    #endif

    virtual
    void
    eval_ISO_D(
      real_type   s,
      real_type   offs,
      real_type & x_D,
      real_type & y_D
    ) const;

    void
    eval_SAE_D(
      real_type   s,
      real_type   offs,
      real_type & x_D,
      real_type & y_D
    ) const {
      this->eval_ISO_D( s, -offs, x_D, y_D );
    }

    #ifdef G2LIB_COMPATIBILITY_MODE
    void
    eval_D(
      real_type   s,
      real_type   offs,
      real_type & x_D,
      real_type & y_D
    ) const {
      if ( G2lib::use_ISO ) this->eval_ISO_D( s, offs, x_D, y_D );
      else                  this->eval_SAE_D( s, offs, x_D, y_D );
    }
    #endif

    virtual
    void
    eval_ISO_DD(
      real_type   s,
      real_type   offs,
      real_type & x_DD,
      real_type & y_DD
    ) const;

    void
    eval_SAE_DD(
      real_type   s,
      real_type   offs,
      real_type & x_DD,
      real_type & y_DD
    ) const {
      this->eval_ISO_DD( s, -offs, x_DD, y_DD );
    }

    #ifdef G2LIB_COMPATIBILITY_MODE
    void
    eval_DD(
      real_type   s,
      real_type   offs,
      real_type & x_DD,
      real_type & y_DD
    ) const {
      if ( G2lib::use_ISO ) this->eval_ISO_DD( s, offs, x_DD, y_DD );
      else                  this->eval_SAE_DD( s, offs, x_DD, y_DD );
    }
    #endif

    virtual
    void
    eval_ISO_DDD(
      real_type   s,
      real_type   offs,
      real_type & x_DDD,
      real_type & y_DDD
    ) const;

    void
    eval_SAE_DDD(
      real_type   s,
      real_type   offs,
      real_type & x_DDD,
      real_type & y_DDD
    ) const {
      this->eval_ISO_DDD( s, -offs, x_DDD, y_DDD );
    }

    #ifdef G2LIB_COMPATIBILITY_MODE
    void
    eval_DDD(
      real_type   s,
      real_type   offs,
      real_type & x_DDD,
      real_type & y_DDD
    ) const {
      if ( G2lib::use_ISO ) this->eval_ISO_DDD( s, offs, x_DDD, y_DDD );
      else                  this->eval_SAE_DDD( s, offs, x_DDD, y_DDD );
    }
    #endif

    /*\
     |  _                        __
     | | |_ _ __ __ _ _ __  ___ / _| ___  _ __ _ __ ___
     | | __| '__/ _` | '_ \/ __| |_ / _ \| '__| '_ ` _ \
     | | |_| | | (_| | | | \__ \  _| (_) | |  | | | | | |
     |  \__|_|  \__,_|_| |_|___/_|  \___/|_|  |_| |_| |_|
    \*/

    virtual
    void
    translate( real_type tx, real_type ty ) = 0;

    virtual
    void
    rotate( real_type angle, real_type cx, real_type cy ) = 0;

    virtual
    void
    scale( real_type sc ) = 0;

    virtual
    void
    reverse() = 0;

    virtual
    void
    changeOrigin( real_type newx0, real_type newy0 ) = 0;

    virtual
    void
    trim( real_type s_begin, real_type s_end ) = 0;

    /*\
     |   _       _                          _
     |  (_)_ __ | |_ ___ _ __ ___  ___  ___| |_
     |  | | '_ \| __/ _ \ '__/ __|/ _ \/ __| __|
     |  | | | | | ||  __/ |  \__ \  __/ (__| |_
     |  |_|_| |_|\__\___|_|  |___/\___|\___|\__|
    \*/

    bool
    collision( BaseCurve const & C ) const
    { return G2lib::collision( *this, C ); }

    bool
    collision_ISO(
      real_type         offs,
      BaseCurve const & C,
      real_type         offs_C
    ) const {
      return G2lib::collision_ISO( *this, offs, C, offs_C );
    }

    bool
    collision_SAE(
      real_type         offs,
      BaseCurve const & C,
      real_type         offs_C
    ) const {
      return G2lib::collision_SAE( *this, offs, C, offs_C );
    }

    #ifdef G2LIB_COMPATIBILITY_MODE
    bool
    collision(
      real_type         offs,
      BaseCurve const & C,
      real_type         offs_C
    ) const {
      if ( G2lib::use_ISO )
        return G2lib::collision_ISO( *this, offs, C, offs_C );
      else
        return G2lib::collision_SAE( *this, offs, C, offs_C );
    }
    #endif

    void
    intersect(
      BaseCurve const & C,
      IntersectList   & ilist,
      bool              swap_s_vals
    ) const {
      G2lib::intersect( *this, C, ilist, swap_s_vals );
    }

    void
    intersect_ISO(
      real_type         offs,
      BaseCurve const & C,
      real_type         offs_C,
      IntersectList   & ilist,
      bool              swap_s_vals
    ) const {
      G2lib::intersect_ISO( *this, offs, C, offs_C, ilist, swap_s_vals );
    }

    void
    intersect_SAE(
      real_type         offs,
      BaseCurve const & C,
      real_type         offs_C,
      IntersectList   & ilist,
      bool              swap_s_vals
    ) const {
      G2lib::intersect_SAE( *this, offs, C, offs_C, ilist, swap_s_vals );
    }

    #ifdef G2LIB_COMPATIBILITY_MODE
    void
    intersect(
      real_type         offs,
      BaseCurve const & C,
      real_type         offs_C,
      IntersectList   & ilist,
      bool              swap_s_vals
    ) const {
      if ( G2lib::use_ISO )
        G2lib::intersect_ISO( *this, offs, C, offs_C, ilist, swap_s_vals );
      else
        G2lib::intersect_SAE( *this, offs, C, offs_C, ilist, swap_s_vals );
    }
    #endif

    /*\
     |      _ _     _
     |   __| (_)___| |_ __ _ _ __   ___ ___
     |  / _` | / __| __/ _` | '_ \ / __/ _ \
     | | (_| | \__ \ || (_| | | | | (_|  __/
     |  \__,_|_|___/\__\__,_|_| |_|\___\___|
    \*/

    virtual
    int_type
    closestPoint_ISO(
      real_type   qx,
      real_type   qy,
      real_type & x,
      real_type & y,
      real_type & s,
      real_type & t,
      real_type & dst
    ) const = 0;

    int_type
    closestPoint_SAE(
      real_type   qx,
      real_type   qy,
      real_type & x,
      real_type & y,
      real_type & s,
      real_type & t,
      real_type & dst
    ) const {
      int_type res = this->closestPoint_ISO( qx, qy, x, y, s, t, dst );
      t = -t;
      return res;
    }

    #ifdef G2LIB_COMPATIBILITY_MODE
    int_type
    closestPoint(
      real_type   qx,
      real_type   qy,
      real_type & x,
      real_type & y,
      real_type & s,
      real_type & t,
      real_type & dst
    ) const {
      if ( G2lib::use_ISO )
        return this->closestPoint_ISO( qx, qy, x, y, s, t, dst );
      else
        return this->closestPoint_SAE( qx, qy, x, y, s, t, dst );
    }
    #endif

    virtual
    int_type // true if projection is unique and orthogonal
    closestPoint_ISO(
      real_type   qx,
      real_type   qy,
      real_type   offs,
      real_type & x,
      real_type & y,
      real_type & s,
      real_type & t,
      real_type & dst
    ) const = 0;

    int_type
    closestPoint_SAE(
      real_type   qx,
      real_type   qy,
      real_type   offs,
      real_type & x,
      real_type & y,
      real_type & s,
      real_type & t,
      real_type & dst
    ) const {
      int_type res = this->closestPoint_ISO( qx, qy, -offs, x, y, s, t, dst );
      t = -t;
      return res;
    }

    #ifdef G2LIB_COMPATIBILITY_MODE
    int_type
    closestPoint(
      real_type   qx,
      real_type   qy,
      real_type   offs,
      real_type & x,
      real_type & y,
      real_type & s,
      real_type & t,
      real_type & dst
    ) const {
      if ( G2lib::use_ISO )
        return this->closestPoint_ISO( qx, qy, offs, x, y, s, t, dst );
      else
        return this->closestPoint_SAE( qx, qy, offs, x, y, s, t, dst );
    }
    #endif

    virtual
    real_type
    distance( real_type qx, real_type qy ) const {
      real_type x, y, s, t, dst;
      closestPoint_ISO( qx, qy, x, y, s, t, dst );
      return dst;
    }

    real_type
    distance_ISO(
      real_type qx,
      real_type qy,
      real_type offs
    ) const {
      real_type x, y, s, t, dst;
      this->closestPoint_ISO( qx, qy, offs, x, y, s, t, dst );
      return dst;
    }

    real_type
    distance_SAE(
      real_type qx,
      real_type qy,
      real_type offs
    ) const {
      real_type x, y, s, t, dst;
      this->closestPoint_SAE( qx, qy, offs, x, y, s, t, dst );
      return dst;
    }

    #ifdef G2LIB_COMPATIBILITY_MODE
    virtual
    real_type
    distance(
      real_type qx,
      real_type qy,
      real_type offs
    ) const {
      real_type x, y, s, t, dst;
      this->closestPoint( qx, qy, offs, x, y, s, t, dst );
      return dst;
    }
    #endif

    /*\
     |    __ _           _ ____ _____
     |   / _(_)_ __   __| / ___|_   _|
     |  | |_| | '_ \ / _` \___ \ | |
     |  |  _| | | | | (_| |___) || |
     |  |_| |_|_| |_|\__,_|____/ |_|
    \*/

    bool
    findST_ISO(
      real_type   x,
      real_type   y,
      real_type & s,
      real_type & t
    ) const {
      real_type X, Y, dst;
      int_type icode = this->closestPoint_ISO( x, y, X, Y, s, t, dst );
      return icode >= 0;
    }

    bool
    findST_SAE(
      real_type   x,
      real_type   y,
      real_type & s,
      real_type & t
    ) const {
      real_type X, Y, dst;
      int_type icode = this->closestPoint_SAE( x, y, X, Y, s, t, dst );
      return icode >= 0;
    }

    #ifdef G2LIB_COMPATIBILITY_MODE
    bool
    findST(
      real_type   x,
      real_type   y,
      real_type & s,
      real_type & t
    ) const {
      real_type X, Y, dst;
      int_type icode = this->closestPoint( x, y, X, Y, s, t, dst );
      return icode >= 0;
    }
    #endif

    // - - - - - - - - - - - - - - - - - - - - - - - - - - - - - - - - - - - - - -

    virtual
    void
    info( ostream_type & stream ) const = 0;

  };

}
```

### Quick search

### Table of Contents

- Matlab Interface Manual
- C++ API
- MATLAB API

«
hide menu

menu
sidebar
»

### Navigation

- index
- toc
- Clothoids »
- Program Listing for File BaseCurve.hxx

© Copyright 2021, Enrico Bertolazzi and Marco Frego.
Created using Sphinx 4.2.0.
