# Supplementary material for: Smooth Interpolating Curves with Local Control and Monotone Alternating Curvature
Source: Comput Graph Forum. 2022 Oct 6;41(5):25–38. doi: 10.1111/cgf.14600 (PMC9827861; doi:10.1111/cgf.14600)
Supplement: Supplementary file 1 — Supplement Material [file CGF-41-25-s001.zip › Local-Smooth-Interpolating-MonoCurvature/extern/clothoids/docs/api-cpp/program_listing_file_Clothoids_BaseCurve_using.hxx.html]

Program Listing for File BaseCurve\_using.hxx — Clothoids v2.0.9

### Navigation

- index
- toc
- Clothoids »
- Program Listing for File BaseCurve\_using.hxx

# Program Listing for File BaseCurve\_using.hxx¶

↰ Return to documentation for file (`Clothoids/BaseCurve_using.hxx`)

```
/*--------------------------------------------------------------------------*\
 |                                                                          |
 |  Copyright (C) 2018                                                      |
 |                                                                          |
 |         , __                 , __                                        |
 |        /|/  \               /|/  \                                       |
 |         | __/ _   ,_         | __/ _   ,_                                |
 |         |   \|/  /  |  |   | |   \|/  /  |  |   |                        |
 |         |(__/|__/   |_/ \_/|/|(__/|__/   |_/ \_/|/                       |
 |                           /|                   /|                        |
 |                           \|                   \|                        |
 |                                                                          |
 |      Paolo Bevilacqua and Enrico Bertolazzi                              |
 |                                                                          |
 |      (1) Dipartimento di Ingegneria e Scienza dell'Informazione          |
 |      (2) Dipartimento di Ingegneria Industriale                          |
 |                                                                          |
 |      Universita` degli Studi di Trento                                   |
 |      email: paolo.bevilacqua@unitn.it                                    |
 |      email: enrico.bertolazzi@unitn.it                                   |
 |                                                                          |
\*--------------------------------------------------------------------------*/


using BaseCurve::thetaBegin;
using BaseCurve::thetaEnd;

using BaseCurve::xBegin;
using BaseCurve::yBegin;
using BaseCurve::xEnd;
using BaseCurve::yEnd;

using BaseCurve::xBegin_ISO;
using BaseCurve::yBegin_ISO;
using BaseCurve::xEnd_ISO;
using BaseCurve::yEnd_ISO;

using BaseCurve::xBegin_SAE;
using BaseCurve::yBegin_SAE;
using BaseCurve::xEnd_SAE;
using BaseCurve::yEnd_SAE;

using BaseCurve::tx_Begin;
using BaseCurve::ty_Begin;
using BaseCurve::tx_End;
using BaseCurve::ty_End;


#ifdef G2LIB_COMPATIBILITY_MODE
using BaseCurve::nx_Begin;
using BaseCurve::ny_Begin;
using BaseCurve::nx_End;
using BaseCurve::ny_End;
#endif

using BaseCurve::nx_Begin_ISO;
using BaseCurve::ny_Begin_ISO;
using BaseCurve::nx_End_ISO;
using BaseCurve::ny_End_ISO;

using BaseCurve::nx_Begin_SAE;
using BaseCurve::ny_Begin_SAE;
using BaseCurve::nx_End_SAE;
using BaseCurve::ny_End_SAE;

using BaseCurve::X;
using BaseCurve::X_D;
using BaseCurve::X_DD;
using BaseCurve::X_DDD;

using BaseCurve::Y;
using BaseCurve::Y_D;
using BaseCurve::Y_DD;
using BaseCurve::Y_DDD;

using BaseCurve::X_SAE;
using BaseCurve::X_SAE_D;
using BaseCurve::X_SAE_DD;
using BaseCurve::X_SAE_DDD;

using BaseCurve::Y_SAE;
using BaseCurve::Y_SAE_D;
using BaseCurve::Y_SAE_DD;
using BaseCurve::Y_SAE_DDD;

using BaseCurve::X_ISO;
using BaseCurve::X_ISO_D;
using BaseCurve::X_ISO_DD;
using BaseCurve::X_ISO_DDD;

using BaseCurve::Y_ISO;
using BaseCurve::Y_ISO_D;
using BaseCurve::Y_ISO_DD;
using BaseCurve::Y_ISO_DDD;

#ifdef G2LIB_COMPATIBILITY_MODE
using BaseCurve::evaluate;
#endif
using BaseCurve::evaluate_ISO;
using BaseCurve::evaluate_SAE;

using BaseCurve::eval;
using BaseCurve::eval_D;
using BaseCurve::eval_DD;
using BaseCurve::eval_DDD;

using BaseCurve::eval_ISO;
using BaseCurve::eval_ISO_D;
using BaseCurve::eval_ISO_DD;
using BaseCurve::eval_ISO_DDD;

using BaseCurve::eval_SAE;
using BaseCurve::eval_SAE_D;
using BaseCurve::eval_SAE_DD;
using BaseCurve::eval_SAE_DDD;

#ifdef G2LIB_COMPATIBILITY_MODE
using BaseCurve::closestPoint;
using BaseCurve::distance;
#endif
using BaseCurve::closestPoint_ISO;
using BaseCurve::closestPoint_SAE;
using BaseCurve::distance_ISO;
using BaseCurve::distance_SAE;
```

### Quick search

### Table of Contents

- Matlab Interface Manual
- C++ API
- MATLAB API

«
hide menu

menu
sidebar
»

### Navigation

- index
- toc
- Clothoids »
- Program Listing for File BaseCurve\_using.hxx

© Copyright 2021, Enrico Bertolazzi and Marco Frego.
Created using Sphinx 4.2.0.
