# Supplementary material for: Smooth Interpolating Curves with Local Control and Monotone Alternating Curvature
Source: Comput Graph Forum. 2022 Oct 6;41(5):25–38. doi: 10.1111/cgf.14600 (PMC9827861; doi:10.1111/cgf.14600)
Supplement: Supplementary file 1 — Supplement Material [file CGF-41-25-s001.zip › Local-Smooth-Interpolating-MonoCurvature/extern/clothoids/docs/api-cpp/program_listing_file_Clothoids_Biarc.hxx.html]

Program Listing for File Biarc.hxx — Clothoids v2.0.9

### Navigation

- index
- toc
- Clothoids »
- Program Listing for File Biarc.hxx

# Program Listing for File Biarc.hxx¶

↰ Return to documentation for file (`Clothoids/Biarc.hxx`)

```
/*--------------------------------------------------------------------------*\
 |                                                                          |
 |  Copyright (C) 2017                                                      |
 |                                                                          |
 |         , __                 , __                                        |
 |        /|/  \               /|/  \                                       |
 |         | __/ _   ,_         | __/ _   ,_                                |
 |         |   \|/  /  |  |   | |   \|/  /  |  |   |                        |
 |         |(__/|__/   |_/ \_/|/|(__/|__/   |_/ \_/|/                       |
 |                           /|                   /|                        |
 |                           \|                   \|                        |
 |                                                                          |
 |      Enrico Bertolazzi                                                   |
 |      Dipartimento di Ingegneria Industriale                              |
 |      Universita` degli Studi di Trento                                   |
 |      email: enrico.bertolazzi@unitn.it                                   |
 |                                                                          |
\*--------------------------------------------------------------------------*/


namespace G2lib {

  /*\
   |   ____  _
   |  | __ )(_) __ _ _ __ ___
   |  |  _ \| |/ _` | '__/ __|
   |  | |_) | | (_| | | | (__
   |  |____/|_|\__,_|_|  \___|
  \*/


  class Biarc : public BaseCurve {
    CircleArc m_C0, m_C1;

    void
    gfun( real_type alpha, real_type g[3] ) const {
      real_type so  = sin(alpha);
      real_type co  = cos(alpha);
      real_type oco = alpha*co;
      g[0] = so + oco;
      g[1] = 2*co - alpha*so;
      g[2] = -3*so - oco;
    }

  public:

    #include "BaseCurve_using.hxx"

    ~Biarc() override {}

    Biarc()
    : BaseCurve(G2LIB_BIARC)
    {}

    Biarc( Biarc const & ba )
    : BaseCurve(G2LIB_BIARC)
    { copy(ba); }

    explicit
    Biarc(
      real_type x0,
      real_type y0,
      real_type theta0,
      real_type x1,
      real_type y1,
      real_type theta1
    )
    : BaseCurve(G2LIB_BIARC)
    {
      bool ok = build( x0, y0, theta0, x1, y1, theta1 );
      UTILS_ASSERT(
        ok,
        "Biarc( x0={}, y0={}, theta0={}, x1={}, y1={}, theta1={}) cannot be computed\n",
        x0, y0, theta0, x1, y1, theta1
      );
    }

    explicit
    Biarc( BaseCurve const & C );

    void
    copy( Biarc const & c ) {
      m_C0.copy(c.m_C0);
      m_C1.copy(c.m_C1);
    }

    Biarc const & operator = ( Biarc const & ba )
    { copy(ba); return *this; }

    CircleArc const & C0() const { return m_C0; }

    CircleArc const & C1() const { return m_C1; }

    bool
    build(
      real_type x0,
      real_type y0,
      real_type theta0,
      real_type x1,
      real_type y1,
      real_type theta1
    );

    bool
    build_3P(
      real_type x0,
      real_type y0,
      real_type x1,
      real_type y1,
      real_type x2,
      real_type y2
    );

    // . . . . . . . . . . . . . . . . . . . . . . . . . . . . . . . . . . . . .

    void
    bbox(
      real_type & xmin,
      real_type & ymin,
      real_type & xmax,
      real_type & ymax
    ) const override;

    void
    bbox_ISO(
      real_type   offs,
      real_type & xmin,
      real_type & ymin,
      real_type & xmax,
      real_type & ymax
    ) const override;

    // . . . . . . . . . . . . . . . . . . . . . . . . . . . . . . . . . . . . .

    real_type
    length() const override
    { return m_C0.length()+m_C1.length(); }

    real_type
    length_ISO( real_type offs ) const override
    { return m_C0.length_ISO(offs)+m_C1.length_ISO(offs); }

    real_type thetaBegin()   const override { return m_C0.thetaBegin(); }
    real_type thetaEnd()     const override { return m_C1.thetaEnd(); }
    real_type kappaBegin()   const override { return m_C0.kappaBegin(); }
    real_type kappaEnd()     const override { return m_C1.kappaEnd(); }
    real_type xBegin()       const override { return m_C0.xBegin(); }
    real_type xEnd()         const override { return m_C1.xEnd(); }
    real_type yBegin()       const override { return m_C0.yBegin(); }
    real_type yEnd()         const override { return m_C1.yEnd(); }
    real_type tx_Begin()     const override { return m_C0.tx_Begin(); }
    real_type tx_End()       const override { return m_C1.tx_End(); }
    real_type ty_Begin()     const override { return m_C0.ty_Begin(); }
    real_type ty_End()       const override { return m_C1.ty_End(); }
    real_type nx_Begin_ISO() const override { return m_C0.nx_Begin_ISO(); }
    real_type nx_End_ISO()   const override { return m_C1.nx_End_ISO(); }
    real_type ny_Begin_ISO() const override { return m_C0.ny_Begin_ISO(); }
    real_type ny_End_ISO()   const override { return m_C1.ny_End_ISO(); }

    // . . . . . . . . . . . . . . . . . . . . . . . . . . . . . . . . . . . . .

    real_type theta    ( real_type ) const override;
    real_type theta_D  ( real_type ) const override;
    real_type theta_DD ( real_type ) const override { return 0; }
    real_type theta_DDD( real_type ) const override { return 0; }

    void
    evaluate(
      real_type   s,
      real_type & th,
      real_type & k,
      real_type & x,
      real_type & y
    ) const override;

    // . . . . . . . . . . . . . . . . . . . . . . . . . . . . . . . . . . . . .

    real_type X( real_type s ) const override;
    real_type Y( real_type s ) const override;

    real_type X_D( real_type ) const override;
    real_type Y_D( real_type ) const override;

    real_type X_DD( real_type ) const override;
    real_type Y_DD( real_type ) const override;

    real_type X_DDD( real_type ) const override;
    real_type Y_DDD( real_type ) const override;

    real_type X_ISO( real_type s, real_type offs ) const override;
    real_type Y_ISO( real_type s, real_type offs ) const override;

    real_type X_ISO_D( real_type, real_type offs ) const override;
    real_type Y_ISO_D( real_type, real_type offs ) const override;

    real_type X_ISO_DD( real_type, real_type offs ) const override;
    real_type Y_ISO_DD( real_type, real_type offs ) const override;

    real_type X_ISO_DDD( real_type, real_type offs ) const override;
    real_type Y_ISO_DDD( real_type, real_type offs ) const override;

    // . . . . . . . . . . . . . . . . . . . . . . . . . . . . . . . . . . . . .

    void
    eval(
      real_type   s,
      real_type & x,
      real_type & y
    ) const override;

    void
    eval_D(
      real_type   s,
      real_type & x_D,
      real_type & y_D
    ) const override;

    void
    eval_DD(
      real_type   s,
      real_type & x_DD,
      real_type & y_DD
    ) const override;

    void
    eval_DDD(
      real_type   s,
      real_type & x_DDD,
      real_type & y_DDD
    ) const override;

    void
    eval_ISO(
      real_type   s,
      real_type   offs,
      real_type & x,
      real_type & y
    ) const override;

    void
    eval_ISO_D(
      real_type   s,
      real_type   offs,
      real_type & x_D,
      real_type & y_D
    ) const override;

    void
    eval_ISO_DD(
      real_type   s,
      real_type   offs,
      real_type & x_DD,
      real_type & y_DD
    ) const override;

    void
    eval_ISO_DDD(
      real_type   s,
      real_type   offs,
      real_type & x_DDD,
      real_type & y_DDD
    ) const override;

    /*\
     |  _____                   _   _   _
     | |_   _|   __ _ _ __   __| | | \ | |
     |   | |    / _` | '_ \ / _` | |  \| |
     |   | |   | (_| | | | | (_| | | |\  |
     |   |_|    \__,_|_| |_|\__,_| |_| \_|
    \*/

    real_type tx    ( real_type s ) const override;
    real_type tx_D  ( real_type s ) const override;
    real_type tx_DD ( real_type s ) const override;
    real_type tx_DDD( real_type s ) const override;
    real_type ty    ( real_type s ) const override;
    real_type ty_D  ( real_type s ) const override;
    real_type ty_DD ( real_type s ) const override;
    real_type ty_DDD( real_type s ) const override;

    // . . . . . . . . . . . . . . . . . . . . . . . . . . . . . . . . . . . . .

    void
    tg(
      real_type   s,
      real_type & tx,
      real_type & ty
    ) const override;

    void
    tg_D(
      real_type   s,
      real_type & tx_D,
      real_type & ty_D
    ) const override;

    void
    tg_DD(
      real_type   s,
      real_type & tx_DD,
      real_type & ty_DD
    ) const override;

    void
    tg_DDD(
      real_type   s,
      real_type & tx_DDD,
      real_type & ty_DDD
    ) const override;

    /*\
     |  _                        __
     | | |_ _ __ __ _ _ __  ___ / _| ___  _ __ _ __ ___
     | | __| '__/ _` | '_ \/ __| |_ / _ \| '__| '_ ` _ \
     | | |_| | | (_| | | | \__ \  _| (_) | |  | | | | | |
     |  \__|_|  \__,_|_| |_|___/_|  \___/|_|  |_| |_| |_|
    \*/

    void
    translate( real_type tx, real_type ty ) override
    { m_C0.translate(tx,ty); m_C1.translate(tx,ty); }

    void
    rotate( real_type angle, real_type cx, real_type cy ) override
    { m_C0.rotate(angle,cx,cy); m_C1.rotate(angle,cx,cy); }

    void
    reverse() override;

    void
    changeOrigin( real_type newx0, real_type newy0 ) override;

    void
    trim( real_type s_begin, real_type s_end ) override;

    void
    scale( real_type s ) override;

    /*\
     |        _                     _   ____       _       _
     |    ___| | ___  ___  ___  ___| |_|  _ \ ___ (_)_ __ | |_
     |   / __| |/ _ \/ __|/ _ \/ __| __| |_) / _ \| | '_ \| __|
     |  | (__| | (_) \__ \  __/\__ \ |_|  __/ (_) | | | | | |_
     |   \___|_|\___/|___/\___||___/\__|_|   \___/|_|_| |_|\__|
    \*/

    int_type
    closestPoint_ISO(
      real_type   qx,
      real_type   qy,
      real_type & x,
      real_type & y,
      real_type & s,
      real_type & t,
      real_type & dst
    ) const override;

    int_type
    closestPoint_ISO(
      real_type   qx,
      real_type   qy,
      real_type   offs,
      real_type & x,
      real_type & y,
      real_type & s,
      real_type & t,
      real_type & dst
    ) const override;

    // . . . . . . . . . . . . . . . . . . . . . . . . . . . . . . . . . . . . .
    // . . . . . . . . . . . . . . . . . . . . . . . . . . . . . . . . . . . . .

    real_type xMiddle() const { return m_C1.xBegin(); }

    real_type yMiddle() const { return m_C1.yBegin(); }

    real_type thetaMiddle() const { return m_C1.thetaBegin(); }

    real_type kappa0() const { return m_C0.curvature(); }

    real_type length0() const { return m_C0.length(); }

    real_type kappa1() const { return m_C1.curvature(); }

    real_type length1() const { return m_C1.length(); }

    real_type delta_theta() const { return m_C0.delta_theta() + m_C1.delta_theta(); }

    void
    bbTriangles(
      std::vector<Triangle2D> & tvec,
      real_type                 max_angle = Utils::m_pi/18,
      real_type                 max_size  = 1e100,
      int_type                  icurve    = 0
    ) const override {
      m_C0.bbTriangles( tvec, max_angle, max_size, icurve );
      m_C1.bbTriangles( tvec, max_angle, max_size, icurve );
    }

    void
    bbTriangles_ISO(
      real_type                 offs,
      std::vector<Triangle2D> & tvec,
      real_type                 max_angle = Utils::m_pi/18,
      real_type                 max_size  = 1e100,
      int_type                  icurve    = 0
    ) const override {
      m_C0.bbTriangles_ISO( offs, tvec, max_angle, max_size, icurve );
      m_C1.bbTriangles_ISO( offs, tvec, max_angle, max_size, icurve );
    }

    void
    bbTriangles_SAE(
      real_type                 offs,
      std::vector<Triangle2D> & tvec,
      real_type                 max_angle = Utils::m_pi/18,
      real_type                 max_size  = 1e100,
      int_type                  icurve    = 0
    ) const override {
      m_C0.bbTriangles_SAE( offs, tvec, max_angle, max_size, icurve );
      m_C1.bbTriangles_SAE( offs, tvec, max_angle, max_size, icurve );
    }

    /*\
     |             _ _ _     _
     |    ___ ___ | | (_)___(_) ___  _ __
     |   / __/ _ \| | | / __| |/ _ \| '_ \
     |  | (_| (_) | | | \__ \ | (_) | | | |
     |   \___\___/|_|_|_|___/_|\___/|_| |_|
    \*/

    bool
    collision( Biarc const & B ) const {
      return m_C0.collision( B.m_C0 ) || m_C0.collision( B.m_C1 ) ||
             m_C1.collision( B.m_C0 ) || m_C1.collision( B.m_C1 );
    }

    bool
    collision_ISO(
      real_type     offs,
      Biarc const & B,
      real_type     offs_B
    ) const {
      return m_C0.collision_ISO( offs, B.m_C0, offs_B ) ||
             m_C0.collision_ISO( offs, B.m_C1, offs_B ) ||
             m_C1.collision_ISO( offs, B.m_C0, offs_B ) ||
             m_C1.collision_ISO( offs, B.m_C1, offs_B );
    }

    /*\
     |   _       _                          _
     |  (_)_ __ | |_ ___ _ __ ___  ___  ___| |_
     |  | | '_ \| __/ _ \ '__/ __|/ _ \/ __| __|
     |  | | | | | ||  __/ |  \__ \  __/ (__| |_
     |  |_|_| |_|\__\___|_|  |___/\___|\___|\__|
    \*/

    void
    intersect(
      Biarc const   & B,
      IntersectList & ilist,
      bool            swap_s_vals
    ) const;

    void
    intersect_ISO(
      real_type       offs,
      Biarc const   & B,
      real_type       offs_B,
      IntersectList & ilist,
      bool            swap_s_vals
    ) const;

    void
    info( ostream_type & stream ) const override
    { stream << "BiArc\n" << *this << '\n'; }

    friend
    ostream_type &
    operator << ( ostream_type & stream, Biarc const & bi );

  };

  // - - - - - - - - - - - - - - - - - - - - - - - - - - - - - - - - - - - - - -

  bool
  build_guess_theta(
    int_type          n,
    real_type const * x,
    real_type const * y,
    real_type       * theta
  );

}
```

### Quick search

### Table of Contents

- Matlab Interface Manual
- C++ API
- MATLAB API

«
hide menu

menu
sidebar
»

### Navigation

- index
- toc
- Clothoids »
- Program Listing for File Biarc.hxx

© Copyright 2021, Enrico Bertolazzi and Marco Frego.
Created using Sphinx 4.2.0.
