# Supplementary material for: Smooth Interpolating Curves with Local Control and Monotone Alternating Curvature
Source: Comput Graph Forum. 2022 Oct 6;41(5):25–38. doi: 10.1111/cgf.14600 (PMC9827861; doi:10.1111/cgf.14600)
Supplement: Supplementary file 1 — Supplement Material [file CGF-41-25-s001.zip › Local-Smooth-Interpolating-MonoCurvature/extern/clothoids/docs/api-cpp/program_listing_file_Clothoids_BiarcList.hxx.html]

Program Listing for File BiarcList.hxx — Clothoids v2.0.9

### Navigation

- index
- toc
- Clothoids »
- Program Listing for File BiarcList.hxx

# Program Listing for File BiarcList.hxx¶

↰ Return to documentation for file (`Clothoids/BiarcList.hxx`)

```
/*--------------------------------------------------------------------------*\
 |                                                                          |
 |  Copyright (C) 2017                                                      |
 |                                                                          |
 |         , __                 , __                                        |
 |        /|/  \               /|/  \                                       |
 |         | __/ _   ,_         | __/ _   ,_                                |
 |         |   \|/  /  |  |   | |   \|/  /  |  |   |                        |
 |         |(__/|__/   |_/ \_/|/|(__/|__/   |_/ \_/|/                       |
 |                           /|                   /|                        |
 |                           \|                   \|                        |
 |                                                                          |
 |      Enrico Bertolazzi                                                   |
 |      Dipartimento di Ingegneria Industriale                              |
 |      Universita` degli Studi di Trento                                   |
 |      email: enrico.bertolazzi@unitn.it                                   |
 |                                                                          |
\*--------------------------------------------------------------------------*/


namespace G2lib {

  using std::vector;

  class ClothoidList;

  /*\
   |  ____  _                _     _     _
   | | __ )(_) __ _ _ __ ___| |   (_)___| |_
   | |  _ \| |/ _` | '__/ __| |   | / __| __|
   | | |_) | | (_| | | | (__| |___| \__ \ |_
   | |____/|_|\__,_|_|  \___|_____|_|___/\__|
  \*/
  class BiarcList : public BaseCurve {

    friend class ClothoidList;

    vector<real_type> m_s0;
    vector<Biarc>     m_biarcList;

    mutable Utils::BinarySearch<int_type> m_lastInterval;

    mutable bool               m_aabb_done;
    mutable AABBtree           m_aabb_tree;
    mutable real_type          m_aabb_offs;
    mutable real_type          m_aabb_max_angle;
    mutable real_type          m_aabb_max_size;
    mutable vector<Triangle2D> m_aabb_tri;

    #ifndef DOXYGEN_SHOULD_SKIP_THIS
    class T2D_collision_list_ISO {
      BiarcList const * m_pList1;
      real_type const   m_offs1;
      BiarcList const * m_pList2;
      real_type const   m_offs2;
    public:
      T2D_collision_list_ISO(
        BiarcList const * pList1,
        real_type const   offs1,
        BiarcList const * pList2,
        real_type const   offs2
      )
      : m_pList1(pList1)
      , m_offs1(offs1)
      , m_pList2(pList2)
      , m_offs2(offs2)
      {}

      bool
      operator () ( BBox::PtrBBox ptr1, BBox::PtrBBox ptr2 ) const {
        Triangle2D const & T1 = m_pList1->m_aabb_tri[size_t(ptr1->Ipos())];
        Triangle2D const & T2 = m_pList2->m_aabb_tri[size_t(ptr2->Ipos())];
        Biarc      const & C1 = m_pList1->get(T1.Icurve());
        Biarc      const & C2 = m_pList2->get(T2.Icurve());
        return C1.collision_ISO( m_offs1, C2, m_offs2 );
      }
    };
    #endif

    void
    resetLastInterval() {
      bool ok;
      int_type & lastInterval = *m_lastInterval.search( std::this_thread::get_id(), ok );
      lastInterval = 0;
    }

    int_type
    closestPoint_internal(
      real_type   qx,
      real_type   qy,
      real_type   offs,
      real_type & x,
      real_type & y,
      real_type & s,
      real_type & dst
    ) const;

  public:

    #include "BaseCurve_using.hxx"

    BiarcList()
    : BaseCurve(G2LIB_BIARC_LIST)
    , m_aabb_done(false)
    { this->resetLastInterval(); }

    ~BiarcList() override {
      m_s0.clear();
      m_biarcList.clear();
      m_aabb_tri.clear();
    }

    BiarcList( BiarcList const & s )
    : BaseCurve(G2LIB_BIARC_LIST)
    , m_aabb_done(false)
    { this->resetLastInterval(); copy(s); }

    void init();

    void reserve( int_type n );

    void copy( BiarcList const & L );

    BiarcList const & operator = ( BiarcList const & s )
    { copy(s); return *this; }

    explicit BiarcList( LineSegment const & LS );

    explicit BiarcList( CircleArc const & C );

    explicit BiarcList( Biarc const & C );

    explicit BiarcList( PolyLine const & pl );

    explicit BiarcList( BaseCurve const & C );

    void push_back( LineSegment const & c );

    void push_back( CircleArc const & c );

    void push_back( Biarc const & c );

    void push_back( PolyLine const & c );

    void push_back_G1( real_type x1, real_type y1, real_type theta1 );

    void
    push_back_G1(
      real_type x0, real_type y0, real_type theta0,
      real_type x1, real_type y1, real_type theta1
    );

    bool
    build_G1(
      int_type          n,
      real_type const * x,
      real_type const * y
    );

    bool
    build_G1(
      int_type          n,
      real_type const * x,
      real_type const * y,
      real_type const * theta
    );

    Biarc const & get( int_type idx ) const;

    Biarc const & getAtS( real_type s ) const;

    int_type numSegments() const { return int_type(m_biarcList.size()); }

    int_type findAtS( real_type & s ) const;

    // . . . . . . . . . . . . . . . . . . . . . . . . . . . . . . . . . . . . .

    real_type length() const override;
    real_type length_ISO( real_type offs ) const override;

    real_type
    segment_length( int_type nseg ) const;

    real_type
    segment_length_ISO( int_type nseg, real_type offs ) const;

    /*\
     |  _    _   _____    _                _
     | | |__| |_|_   _| _(_)__ _ _ _  __ _| |___
     | | '_ \ '_ \| || '_| / _` | ' \/ _` | / -_)
     | |_.__/_.__/|_||_| |_\__,_|_||_\__, |_\___|
     |                               |___/
    \*/

    void
    bbTriangles_ISO(
      real_type                 offs,
      std::vector<Triangle2D> & tvec,
      real_type                 max_angle = Utils::m_pi/6, // 30 degree
      real_type                 max_size  = 1e100,
      int_type                  icurve    = 0
    ) const override;

    void
    bbTriangles_SAE(
      real_type                 offs,
      std::vector<Triangle2D> & tvec,
      real_type                 max_angle = Utils::m_pi/6, // 30 degree
      real_type                 max_size  = 1e100,
      int_type                  icurve    = 0
    ) const override;

    void
    bbTriangles(
      std::vector<Triangle2D> & tvec,
      real_type                 max_angle = Utils::m_pi/6, // 30 degree
      real_type                 max_size  = 1e100,
      int_type                  icurve    = 0
    ) const override;

    #ifndef DOXYGEN_SHOULD_SKIP_THIS
    void
    build_AABBtree_ISO(
      real_type offs,
      real_type max_angle = Utils::m_pi/6, // 30 degree
      real_type max_size  = 1e100
    ) const;

    void
    build_AABBtree_SAE(
      real_type offs,
      real_type max_angle = Utils::m_pi/6, // 30 degree
      real_type max_size  = 1e100
    ) const {
      build_AABBtree_ISO( -offs, max_angle, max_size );
    }
    #endif

    /*\
     |   _     _
     |  | |__ | |__   _____  __
     |  | '_ \| '_ \ / _ \ \/ /
     |  | |_) | |_) | (_) >  <
     |  |_.__/|_.__/ \___/_/\_\
    \*/

    void
    bbox(
      real_type & xmin,
      real_type & ymin,
      real_type & xmax,
      real_type & ymax
    ) const override {
      bbox_ISO( 0, xmin, ymin, xmax, ymax );
    }

    void
    bbox_ISO(
      real_type   offs,
      real_type & xmin,
      real_type & ymin,
      real_type & xmax,
      real_type & ymax
    ) const override;

    /*\
     |   ____             _          _______           _
     |  | __ )  ___  __ _(_)_ __    / / ____|_ __   __| |
     |  |  _ \ / _ \/ _` | | '_ \  / /|  _| | '_ \ / _` |
     |  | |_) |  __/ (_| | | | | |/ / | |___| | | | (_| |
     |  |____/ \___|\__, |_|_| |_/_/  |_____|_| |_|\__,_|
     |              |___/
    \*/

    real_type
    thetaBegin() const override
    { return m_biarcList.front().thetaBegin(); }

    real_type
    thetaEnd() const override
    { return m_biarcList.back().thetaEnd(); }

    real_type
    xBegin() const override
    { return m_biarcList.front().xBegin(); }

    real_type
    yBegin() const override
    { return m_biarcList.front().yBegin(); }

    real_type
    xEnd() const override
    { return m_biarcList.back().xEnd(); }

    real_type
    yEnd() const override
    { return m_biarcList.back().yEnd(); }

    real_type
    xBegin_ISO( real_type offs ) const override
    { return m_biarcList.front().xBegin_ISO( offs ); }

    real_type
    yBegin_ISO( real_type offs ) const override
    { return m_biarcList.front().yBegin_ISO( offs ); }

    real_type
    xEnd_ISO( real_type offs ) const override
    { return m_biarcList.back().xEnd_ISO( offs ); }

    real_type
    yEnd_ISO( real_type offs ) const override
    { return m_biarcList.back().yEnd_ISO( offs ); }

    real_type
    tx_Begin() const override
    { return m_biarcList.front().tx_Begin(); }

    real_type
    ty_Begin() const override
    { return m_biarcList.front().ty_Begin(); }

    real_type
    tx_End() const override
    { return m_biarcList.back().tx_End(); }

    real_type
    ty_End() const override
    { return m_biarcList.back().ty_End(); }

    real_type
    nx_Begin_ISO() const override
    { return m_biarcList.front().nx_Begin_ISO(); }

    real_type
    ny_Begin_ISO() const override
    { return m_biarcList.front().ny_Begin_ISO(); }

    real_type
    nx_End_ISO() const override
    { return m_biarcList.back().nx_End_ISO(); }

    real_type
    ny_End_ISO() const override
    { return m_biarcList.back().ny_End_ISO(); }

    /*\
     |  _   _          _
     | | |_| |__   ___| |_ __ _
     | | __| '_ \ / _ \ __/ _` |
     | | |_| | | |  __/ || (_| |
     |  \__|_| |_|\___|\__\__,_|
    \*/

    real_type theta    ( real_type ) const override;
    real_type theta_D  ( real_type ) const override;
    real_type theta_DD ( real_type ) const override;
    real_type theta_DDD( real_type ) const override;

    /*\
     |  _____                   _   _   _
     | |_   _|   __ _ _ __   __| | | \ | |
     |   | |    / _` | '_ \ / _` | |  \| |
     |   | |   | (_| | | | | (_| | | |\  |
     |   |_|    \__,_|_| |_|\__,_| |_| \_|
    \*/

    real_type tx    ( real_type ) const override;
    real_type ty    ( real_type ) const override;
    real_type tx_D  ( real_type ) const override;
    real_type ty_D  ( real_type ) const override;
    real_type tx_DD ( real_type ) const override;
    real_type ty_DD ( real_type ) const override;
    real_type tx_DDD( real_type ) const override;
    real_type ty_DDD( real_type ) const override;

    // . . . . . . . . . . . . . . . . . . . . . . . . . . . . . . . . . . . . .

    void
    tg(
      real_type   s,
      real_type & tg_x,
      real_type & tg_y
    ) const override;

    void
    tg_D(
      real_type   s,
      real_type & tg_x_D,
      real_type & tg_y_D
    ) const override;

    void
    tg_DD(
      real_type   s,
      real_type & tg_x_DD,
      real_type & tg_y_DD
    ) const override;

    void
    tg_DDD(
      real_type   s,
      real_type & tg_x_DDD,
      real_type & tg_y_DDD
    ) const override;

    // . . . . . . . . . . . . . . . . . . . . . . . . . . . . . . . . . . . . .

    void
    evaluate(
      real_type   s,
      real_type & th,
      real_type & k,
      real_type & x,
      real_type & y
    ) const override;

    // . . . . . . . . . . . . . . . . . . . . . . . . . . . . . . . . . . . . .

    void
    evaluate_ISO(
      real_type   s,
      real_type   offs,
      real_type & th,
      real_type & k,
      real_type & x,
      real_type & y
    ) const override;

    // . . . . . . . . . . . . . . . . . . . . . . . . . . . . . . . . . . . . .

    real_type X    ( real_type ) const override;
    real_type Y    ( real_type ) const override;
    real_type X_D  ( real_type ) const override;
    real_type Y_D  ( real_type ) const override;
    real_type X_DD ( real_type ) const override;
    real_type Y_DD ( real_type ) const override;
    real_type X_DDD( real_type ) const override;
    real_type Y_DDD( real_type ) const override;

    // . . . . . . . . . . . . . . . . . . . . . . . . . . . . . . . . . . . . .

    void
    eval(
      real_type   s,
      real_type & x,
      real_type & y
    ) const override;

    void
    eval_D(
      real_type   s,
      real_type & x_D,
      real_type & y_D
    ) const override;

    void
    eval_DD(
      real_type   s,
      real_type & x_DD,
      real_type & y_DD
    ) const override;

    void
    eval_DDD(
      real_type   s,
      real_type & x_DDD,
      real_type & y_DDD
    ) const override;

    /*\
     |         __  __          _
     |   ___  / _|/ _|___  ___| |_
     |  / _ \| |_| |_/ __|/ _ \ __|
     | | (_) |  _|  _\__ \  __/ |_
     |  \___/|_| |_| |___/\___|\__|
    \*/

    real_type X_ISO    ( real_type s, real_type offs ) const override;
    real_type Y_ISO    ( real_type s, real_type offs ) const override;
    real_type X_ISO_D  ( real_type s, real_type offs ) const override;
    real_type Y_ISO_D  ( real_type s, real_type offs ) const override;
    real_type X_ISO_DD ( real_type s, real_type offs ) const override;
    real_type Y_ISO_DD ( real_type s, real_type offs ) const override;
    real_type X_ISO_DDD( real_type s, real_type offs ) const override;
    real_type Y_ISO_DDD( real_type s, real_type offs ) const override;

    // . . . . . . . . . . . . . . . . . . . . . . . . . . . . . . . . . . . . .

    void
    eval_ISO(
      real_type   s,
      real_type   offs,
      real_type & x,
      real_type & y
    ) const override;

    void
    eval_ISO_D(
      real_type   s,
      real_type   offs,
      real_type & x_D,
      real_type & y_D
    ) const override;

    void
    eval_ISO_DD(
      real_type   s,
      real_type   offs,
      real_type & x_DD,
      real_type & y_DD
    ) const override;

    void
    eval_ISO_DDD(
      real_type   s,
      real_type   offs,
      real_type & x_DDD,
      real_type & y_DDD
    ) const override;

    /*\
     |  _                        __
     | | |_ _ __ __ _ _ __  ___ / _| ___  _ __ _ __ ___
     | | __| '__/ _` | '_ \/ __| |_ / _ \| '__| '_ ` _ \
     | | |_| | | (_| | | | \__ \  _| (_) | |  | | | | | |
     |  \__|_|  \__,_|_| |_|___/_|  \___/|_|  |_| |_| |_|
    \*/

    void translate( real_type tx, real_type ty ) override;
    void rotate( real_type angle, real_type cx, real_type cy ) override;
    void scale( real_type sc ) override;
    void reverse() override;
    void changeOrigin( real_type newx0, real_type newy0 ) override;
    void trim( real_type s_begin, real_type s_end ) override;

    /*\
     |      _ _     _
     |   __| (_)___| |_ __ _ _ __   ___ ___
     |  / _` | / __| __/ _` | '_ \ / __/ _ \
     | | (_| | \__ \ || (_| | | | | (_|  __/
     |  \__,_|_|___/\__\__,_|_| |_|\___\___|
    \*/

    int_type
    closestPoint_ISO(
      real_type   qx,
      real_type   qy,
      real_type & x,
      real_type & y,
      real_type & s,
      real_type & t,
      real_type & dst
    ) const override;

    int_type // true if projection is unique and orthogonal
    closestPoint_ISO(
      real_type   qx,
      real_type   qy,
      real_type   offs,
      real_type & x,
      real_type & y,
      real_type & s,
      real_type & t,
      real_type & dst
    ) const override;

    void
    info( ostream_type & stream ) const override
    { stream << "BiarcList\n" << *this << '\n'; }

    friend
    ostream_type &
    operator << ( ostream_type & stream, BiarcList const & CL );

    void
    getSTK(
      real_type * s,
      real_type * theta,
      real_type * kappa
    ) const;

    void
    getXY( real_type * x, real_type * y ) const;

    int_type
    findST1(
      real_type   x,
      real_type   y,
      real_type & s,
      real_type & t
    ) const;

    int_type
    findST1(
      int_type    ibegin,
      int_type    iend,
      real_type   x,
      real_type   y,
      real_type & s,
      real_type & t
    ) const;

    /*\
     |             _ _ _     _
     |    ___ ___ | | (_)___(_) ___  _ __
     |   / __/ _ \| | | / __| |/ _ \| '_ \
     |  | (_| (_) | | | \__ \ | (_) | | | |
     |   \___\___/|_|_|_|___/_|\___/|_| |_|
    \*/

    bool
    collision( BiarcList const & C ) const;

    bool
    collision_ISO(
      real_type         offs,
      BiarcList const & CL,
      real_type         offs_C
    ) const;

    /*\
     |   _       _                          _
     |  (_)_ __ | |_ ___ _ __ ___  ___  ___| |_
     |  | | '_ \| __/ _ \ '__/ __|/ _ \/ __| __|
     |  | | | | | ||  __/ |  \__ \  __/ (__| |_
     |  |_|_| |_|\__\___|_|  |___/\___|\___|\__|
    \*/

    void
    intersect(
      BiarcList const & CL,
      IntersectList   & ilist,
      bool              swap_s_vals
    ) const {
      intersect_ISO( 0, CL, 0, ilist, swap_s_vals );
    }

    void
    intersect_ISO(
      real_type         offs,
      BiarcList const & CL,
      real_type         offs_obj,
      IntersectList   & ilist,
      bool              swap_s_vals
    ) const;

  };

}
```

### Quick search

### Table of Contents

- Matlab Interface Manual
- C++ API
- MATLAB API

«
hide menu

menu
sidebar
»

### Navigation

- index
- toc
- Clothoids »
- Program Listing for File BiarcList.hxx

© Copyright 2021, Enrico Bertolazzi and Marco Frego.
Created using Sphinx 4.2.0.
