# Supplementary material for: Smooth Interpolating Curves with Local Control and Monotone Alternating Curvature
Source: Comput Graph Forum. 2022 Oct 6;41(5):25–38. doi: 10.1111/cgf.14600 (PMC9827861; doi:10.1111/cgf.14600)
Supplement: Supplementary file 1 — Supplement Material [file CGF-41-25-s001.zip › Local-Smooth-Interpolating-MonoCurvature/extern/clothoids/docs/api-cpp/program_listing_file_Clothoids_Circle.hxx.html]

Program Listing for File Circle.hxx — Clothoids v2.0.9

### Navigation

- index
- toc
- Clothoids »
- Program Listing for File Circle.hxx

# Program Listing for File Circle.hxx¶

↰ Return to documentation for file (`Clothoids/Circle.hxx`)

```
/*--------------------------------------------------------------------------*\
 |                                                                          |
 |  Copyright (C) 2017                                                      |
 |                                                                          |
 |         , __                 , __                                        |
 |        /|/  \               /|/  \                                       |
 |         | __/ _   ,_         | __/ _   ,_                                |
 |         |   \|/  /  |  |   | |   \|/  /  |  |   |                        |
 |         |(__/|__/   |_/ \_/|/|(__/|__/   |_/ \_/|/                       |
 |                           /|                   /|                        |
 |                           \|                   \|                        |
 |                                                                          |
 |      Enrico Bertolazzi                                                   |
 |      Dipartimento di Ingegneria Industriale                              |
 |      Universita` degli Studi di Trento                                   |
 |      email: enrico.bertolazzi@unitn.it                                   |
 |                                                                          |
\*--------------------------------------------------------------------------*/


namespace G2lib {

  /*\
   |    ____ _          _         _
   |   / ___(_)_ __ ___| | ___   / \   _ __ ___
   |  | |   | | '__/ __| |/ _ \ / _ \ | '__/ __|
   |  | |___| | | | (__| |  __// ___ \| | | (__
   |   \____|_|_|  \___|_|\___/_/   \_\_|  \___|
  \*/

  class CircleArc : public BaseCurve {

    friend class Biarc;

    real_type m_x0;
    real_type m_y0;
    real_type m_theta0;
    real_type m_c0;
    real_type m_s0;
    real_type m_k;

    real_type m_L;

  public:

    #include "BaseCurve_using.hxx"

    CircleArc()
    : BaseCurve(G2LIB_CIRCLE)
    , m_x0(0)
    , m_y0(0)
    , m_theta0(0)
    , m_c0(1)
    , m_s0(0)
    , m_k(0)
    , m_L(0)
    {}

    CircleArc( CircleArc const & s )
    : BaseCurve(G2LIB_CIRCLE)
    { copy(s); }

    explicit
    CircleArc(
      real_type x0,
      real_type y0,
      real_type theta0,
      real_type k,
      real_type L
    )
    : BaseCurve(G2LIB_CIRCLE)
    , m_x0(x0)
    , m_y0(y0)
    , m_theta0(theta0)
    , m_c0(cos(theta0))
    , m_s0(sin(theta0))
    , m_k(k)
    , m_L(L)
    {}

    explicit
    CircleArc( LineSegment const & LS )
    : BaseCurve(G2LIB_CIRCLE)
    , m_x0(LS.xBegin())
    , m_y0(LS.yBegin())
    , m_theta0(LS.m_theta0)
    , m_c0(LS.m_c0)
    , m_s0(LS.m_s0)
    , m_k(0)
    , m_L(LS.length())
    {}

    void
    copy( CircleArc const & c ) {
      m_x0     = c.m_x0;
      m_y0     = c.m_y0;
      m_theta0 = c.m_theta0;
      m_c0     = c.m_c0;
      m_s0     = c.m_s0;
      m_k      = c.m_k;
      m_L      = c.m_L;
    }

    explicit
    CircleArc( BaseCurve const & C );

    CircleArc const &
    operator = ( CircleArc const & s )
    { copy(s); return *this; }

    void
    build(
      real_type x0,
      real_type y0,
      real_type theta0,
      real_type k,
      real_type L
    ) {
      m_x0     = x0;
      m_y0     = y0;
      m_theta0 = theta0;
      m_k      = k;
      m_L      = L;
    }

    bool
    build_G1(
      real_type x0,
      real_type y0,
      real_type theta0,
      real_type x1,
      real_type y1
    );

    bool
    build_3P(
      real_type x0,
      real_type y0,
      real_type x1,
      real_type y1,
      real_type x2,
      real_type y2
    );

    // . . . . . . . . . . . . . . . . . . . . . . . . . . . . . . . . . . . . .

    bool
    bbTriangle(
      real_type & x0, real_type & y0,
      real_type & x1, real_type & y1,
      real_type & x2, real_type & y2
    ) const;

    bool
    bbTriangle_ISO(
      real_type   offs,
      real_type & x0, real_type & y0,
      real_type & x1, real_type & y1,
      real_type & x2, real_type & y2
    ) const;

    bool
    bbTriangle_SAE(
      real_type   offs,
      real_type & x0, real_type & y0,
      real_type & x1, real_type & y1,
      real_type & x2, real_type & y2
    ) const {
      return this->bbTriangle_ISO( -offs, x0, y0, x1, y1, x2, y2 );
    }

    bool
    bbTriangle(
      real_type * p0,
      real_type * p1,
      real_type * p2
    ) const {
      return bbTriangle( p0[0], p0[1], p1[0], p1[1], p2[0], p2[1] );
    }

    bool
    bbTriangle_ISO(
      real_type   offs,
      real_type * p0,
      real_type * p1,
      real_type * p2
    ) const {
      return bbTriangle_ISO( offs, p0[0], p0[1], p1[0], p1[1], p2[0], p2[1] );
    }

    bool
    bbTriangle_SAE(
      real_type   offs,
      real_type * p0,
      real_type * p1,
      real_type * p2
    ) const {
      return bbTriangle_SAE( offs, p0[0], p0[1], p1[0], p1[1], p2[0], p2[1] );
    }

    bool
    bbTriangle(
      Triangle2D & t,
      real_type    ss0    = 0,
      real_type    ss1    = 0,
      int_type     icurve = 0
    ) const {
      real_type p0[2], p1[2], p2[2];
      bool ok = bbTriangle( p0, p1, p2 );
      if ( ok ) t.build( p0, p1, p2, ss0, ss1, icurve );
      return ok;
    }

    bool
    bbTriangle_ISO(
      real_type    offs,
      Triangle2D & t,
      real_type    ss0    = 0,
      real_type    ss1    = 0,
      int_type     icurve = 0
    ) const {
      real_type p0[2], p1[2], p2[2];
      bool ok = bbTriangle_ISO( offs, p0, p1, p2 );
      if ( ok ) t.build( p0, p1, p2, ss0, ss1, icurve );
      return ok;
    }

    bool
    bbTriangle_SAE(
      real_type    offs,
      Triangle2D & t,
      real_type    ss0    = 0,
      real_type    ss1    = 0,
      int_type     icurve = 0
    ) const {
      return this->bbTriangle_ISO( -offs, t, ss0, ss1, icurve );
    }

    void
    bbTriangles(
      std::vector<Triangle2D> & tvec,
      real_type max_angle = Utils::m_pi/18,
      real_type max_size  = 1e100,
      int_type  icurve    = 0
    ) const override; // 10 degree

    void
    bbTriangles_ISO(
      real_type offs,
      std::vector<Triangle2D> & tvec,
      real_type max_angle = Utils::m_pi/18,
      real_type max_size  = 1e100,
      int_type  icurve    = 0
    ) const override; // 10 degree

    void
    bbTriangles_SAE(
      real_type offs,
      std::vector<Triangle2D> & tvec,
      real_type max_angle = Utils::m_pi/18,
      real_type max_size  = 1e100,
      int_type  icurve    = 0
    ) const override {
      this->bbTriangles_ISO( -offs, tvec, max_angle, max_size, icurve );
    }

    // . . . . . . . . . . . . . . . . . . . . . . . . . . . . . . . . . . . . .

    void
    bbox(
      real_type & xmin,
      real_type & ymin,
      real_type & xmax,
      real_type & ymax
    ) const override;

    void
    bbox_ISO(
      real_type   offs,
      real_type & xmin,
      real_type & ymin,
      real_type & xmax,
      real_type & ymax
    ) const override;

    // . . . . . . . . . . . . . . . . . . . . . . . . . . . . . . . . . . . . .

    real_type
    length() const override
    { return m_L; }

    real_type
    length_ISO( real_type offs ) const override
    { return m_L*(1+m_k*offs); }

    real_type thetaBegin()   const override { return m_theta0; }
    real_type kappaBegin()   const override { return m_k; }
    real_type kappaEnd()     const override { return m_k; }
    real_type xBegin()       const override { return m_x0; }
    real_type yBegin()       const override { return m_y0; }
    real_type tx_Begin()     const override { return m_c0; }
    real_type ty_Begin()     const override { return m_s0; }
    real_type nx_Begin_ISO() const override { return m_s0; }
    real_type ny_Begin_ISO() const override { return -m_c0; }

    // . . . . . . . . . . . . . . . . . . . . . . . . . . . . . . . . . . . . .

    real_type theta    ( real_type s ) const override { return m_theta0 + s*m_k; }
    real_type theta_D  ( real_type   ) const override { return m_k; }
    real_type theta_DD ( real_type   ) const override { return 0; }
    real_type theta_DDD( real_type   ) const override { return 0; }

    // . . . . . . . . . . . . . . . . . . . . . . . . . . . . . . . . . . . . .

    void
    evaluate(
      real_type   s,
      real_type & th,
      real_type & kappa,
      real_type & x,
      real_type & y
    ) const override {
      eval( s, x, y );
      th     = m_theta0 + s*m_k;
      kappa  = m_k;
    }

    // . . . . . . . . . . . . . . . . . . . . . . . . . . . . . . . . . . . . .

    real_type X( real_type s ) const override;
    real_type Y( real_type s ) const override;

    real_type X_D( real_type ) const override;
    real_type Y_D( real_type ) const override;

    real_type X_DD( real_type ) const override;
    real_type Y_DD( real_type ) const override;

    real_type X_DDD( real_type ) const override;
    real_type Y_DDD( real_type ) const override;

    // . . . . . . . . . . . . . . . . . . . . . . . . . . . . . . . . . . . . .

    void
    eval(
      real_type   s,
      real_type & x,
      real_type & y
    ) const override;

    void
    eval_D(
      real_type,
      real_type & x_D,
      real_type & y_D
    ) const override;

    void
    eval_DD(
      real_type,
      real_type & x_DD,
      real_type & y_DD
    ) const override;

    void
    eval_DDD(
      real_type,
      real_type & x_DDD,
      real_type & y_DDD
    ) const override;

    /*\
     |  _____                   _   _   _
     | |_   _|   __ _ _ __   __| | | \ | |
     |   | |    / _` | '_ \ / _` | |  \| |
     |   | |   | (_| | | | | (_| | | |\  |
     |   |_|    \__,_|_| |_|\__,_| |_| \_|
    \*/

    real_type
    tx( real_type s ) const override
    { return cos(theta(s)); }

    real_type
    tx_D( real_type s ) const override
    { return -sin(theta(s))*m_k; }

    real_type
    tx_DD( real_type s ) const override
    { return -cos(theta(s))*m_k*m_k; }

    real_type
    tx_DDD( real_type s ) const override
    { return sin(theta(s))*m_k*m_k*m_k; }

    real_type
    ty( real_type s ) const override
    { return sin(theta(s)); }

    real_type
    ty_D( real_type s ) const override
    { return cos(theta(s))*m_k; }

    real_type
    ty_DD( real_type s ) const override
    { return -sin(theta(s))*m_k*m_k; }

    real_type
    ty_DDD( real_type s ) const override
    { return -cos(theta(s))*m_k*m_k*m_k; }

    // . . . . . . . . . . . . . . . . . . . . . . . . . . . . . . . . . . . . .

    void tg( real_type s, real_type & tx, real_type & ty ) const override;
    void tg_D( real_type s, real_type & tx_D, real_type & ty_D ) const override;
    void tg_DD( real_type s, real_type & tx_DD, real_type & ty_DD ) const override;
    void tg_DDD( real_type s, real_type & tx_DDD, real_type & ty_DDD ) const override;

    /*\
     |  _                        __
     | | |_ _ __ __ _ _ __  ___ / _| ___  _ __ _ __ ___
     | | __| '__/ _` | '_ \/ __| |_ / _ \| '__| '_ ` _ \
     | | |_| | | (_| | | | \__ \  _| (_) | |  | | | | | |
     |  \__|_|  \__,_|_| |_|___/_|  \___/|_|  |_| |_| |_|
    \*/

    void
    translate( real_type tx, real_type ty ) override
    { m_x0 += tx; m_y0 += ty; }

    void rotate( real_type angle, real_type cx, real_type cy ) override;
    void reverse() override;

    void
    changeOrigin( real_type newx0, real_type newy0 ) override
    { m_x0 = newx0; m_y0 = newy0; }

    void scale( real_type s ) override;
    void trim( real_type s_begin, real_type s_end ) override;

    /*\
     |        _                     _   ____       _       _
     |    ___| | ___  ___  ___  ___| |_|  _ \ ___ (_)_ __ | |_
     |   / __| |/ _ \/ __|/ _ \/ __| __| |_) / _ \| | '_ \| __|
     |  | (__| | (_) \__ \  __/\__ \ |_|  __/ (_) | | | | | |_
     |   \___|_|\___/|___/\___||___/\__|_|   \___/|_|_| |_|\__|
     |
    \*/

    int_type
    closestPoint_ISO(
      real_type   qx,
      real_type   qy,
      real_type & x,
      real_type & y,
      real_type & s,
      real_type & t,
      real_type & dst
    ) const override;

    int_type
    closestPoint_ISO(
      real_type   qx,
      real_type   qy,
      real_type   offs,
      real_type & x,
      real_type & y,
      real_type & s,
      real_type & t,
      real_type & dst
    ) const override;

    void
    info( ostream_type & stream ) const override
    { stream << "CircleArc\n" << *this << '\n'; }

    // . . . . . . . . . . . . . . . . . . . . . . . . . . . . . . . . . . . . .
    // . . . . . . . . . . . . . . . . . . . . . . . . . . . . . . . . . . . . .
    // . . . . . . . . . . . . . . . . . . . . . . . . . . . . . . . . . . . . .

    /*\
     |             _ _ _     _
     |    ___ ___ | | (_)___(_) ___  _ __
     |   / __/ _ \| | | / __| |/ _ \| '_ \
     |  | (_| (_) | | | \__ \ | (_) | | | |
     |   \___\___/|_|_|_|___/_|\___/|_| |_|
    \*/

    bool
    collision( CircleArc const & ) const;

    bool
    collision_ISO(
      real_type         offs,
      CircleArc const & C,
      real_type         offs_obj
    ) const;

    /*\
     |   _       _                          _
     |  (_)_ __ | |_ ___ _ __ ___  ___  ___| |_
     |  | | '_ \| __/ _ \ '__/ __|/ _ \/ __| __|
     |  | | | | | ||  __/ |  \__ \  __/ (__| |_
     |  |_|_| |_|\__\___|_|  |___/\___|\___|\__|
    \*/

    void
    intersect(
      CircleArc const & obj,
      IntersectList   & ilist,
      bool              swap_s_vals
    ) const;

    void
    intersect_ISO(
      real_type         offs,
      CircleArc const & C,
      real_type         offs_obj,
      IntersectList   & ilist,
      bool              swap_s_vals
    ) const;

    real_type sinTheta0() const { return sin(m_theta0); }

    real_type cosTheta0() const { return cos(m_theta0); }

    real_type curvature() const { return m_k; }

    real_type lenTolerance( real_type tol ) const;

    real_type delta_theta() const { return m_L*m_k; }

    real_type thetaTotalVariation() const { return std::abs(m_L*m_k); }

    real_type
    thetaMinMax( real_type & thMin, real_type & thMax ) const;

    real_type
    deltaTheta() const
    { real_type thMin, thMax; return thetaMinMax( thMin, thMax ); }

    void
    changeCurvilinearOrigin( real_type s0, real_type newL );

    void
    center( real_type & cx, real_type & cy ) const;

    real_type ray() const { return 1/std::abs(m_k); }

    /*\
     |   _   _ _   _ ____  ____ ____
     |  | \ | | | | |  _ \| __ ) ___|
     |  |  \| | | | | |_) |  _ \___ \
     |  | |\  | |_| |  _ <| |_) |__) |
     |  |_| \_|\___/|_| \_\____/____/
    \*/

    void
    paramNURBS( int_type & n_knots, int_type & n_pnts ) const;

    void
    toNURBS( real_type * knots, real_type Poly[][3] ) const;

    friend
    ostream_type &
    operator << ( ostream_type & stream, CircleArc const & c );

    friend class ClothoidCurve;

  };

}
```

### Quick search

### Table of Contents

- Matlab Interface Manual
- C++ API
- MATLAB API

«
hide menu

menu
sidebar
»

### Navigation

- index
- toc
- Clothoids »
- Program Listing for File Circle.hxx

© Copyright 2021, Enrico Bertolazzi and Marco Frego.
Created using Sphinx 4.2.0.
