# Supplementary material for: Smooth Interpolating Curves with Local Control and Monotone Alternating Curvature
Source: Comput Graph Forum. 2022 Oct 6;41(5):25–38. doi: 10.1111/cgf.14600 (PMC9827861; doi:10.1111/cgf.14600)
Supplement: Supplementary file 1 — Supplement Material [file CGF-41-25-s001.zip › Local-Smooth-Interpolating-MonoCurvature/extern/clothoids/docs/api-cpp/program_listing_file_Clothoids_Clothoid.hxx.html]

Program Listing for File Clothoid.hxx — Clothoids v2.0.9

### Navigation

- index
- toc
- Clothoids »
- Program Listing for File Clothoid.hxx

# Program Listing for File Clothoid.hxx¶

↰ Return to documentation for file (`Clothoids/Clothoid.hxx`)

```
/*--------------------------------------------------------------------------*\
 |                                                                          |
 |  Copyright (C) 2017                                                      |
 |                                                                          |
 |         , __                 , __                                        |
 |        /|/  \               /|/  \                                       |
 |         | __/ _   ,_         | __/ _   ,_                                |
 |         |   \|/  /  |  |   | |   \|/  /  |  |   |                        |
 |         |(__/|__/   |_/ \_/|/|(__/|__/   |_/ \_/|/                       |
 |                           /|                   /|                        |
 |                           \|                   \|                        |
 |                                                                          |
 |      Enrico Bertolazzi                                                   |
 |      Dipartimento di Ingegneria Industriale                              |
 |      Universita` degli Studi di Trento                                   |
 |      email: enrico.bertolazzi@unitn.it                                   |
 |                                                                          |
\*--------------------------------------------------------------------------*/


namespace G2lib {

  using std::vector;

  /*\
   |    ____ _       _   _           _     _  ____
   |   / ___| | ___ | |_| |__   ___ (_) __| |/ ___|   _ _ ____   _____
   |  | |   | |/ _ \| __| '_ \ / _ \| |/ _` | |  | | | | '__\ \ / / _ \
   |  | |___| | (_) | |_| | | | (_) | | (_| | |__| |_| | |   \ V /  __/
   |   \____|_|\___/ \__|_| |_|\___/|_|\__,_|\____\__,_|_|    \_/ \___|
  \*/
  class ClothoidCurve : public BaseCurve {
    friend class ClothoidList;
  private:

    ClothoidData m_CD;
    real_type    m_L;

    void
    optimized_sample_internal_ISO(
      real_type           s_begin,
      real_type           s_end,
      real_type           offs,
      real_type           ds,
      real_type           max_angle,
      vector<real_type> & s
    ) const;

    void
    bbTriangles_internal_ISO(
      real_type            offs,
      vector<Triangle2D> & tvec,
      real_type            s0,
      real_type            s1,
      real_type            max_angle,
      real_type            max_size,
      int_type             icurve
    ) const;

    void
    closestPoint_internal(
      real_type   s_begin,
      real_type   s_end,
      real_type   qx,
      real_type   qy,
      real_type   offs,
      real_type & x,
      real_type & y,
      real_type & s,
      real_type & dst
    ) const;

    void
    closestPoint_internal(
      real_type   qx,
      real_type   qy,
      real_type   offs,
      real_type & x,
      real_type & y,
      real_type & s,
      real_type & dst
    ) const;

    static int_type  m_max_iter;
    static real_type m_tolerance;

    mutable bool               m_aabb_done;
    mutable AABBtree           m_aabb_tree;
    mutable real_type          m_aabb_offs;
    mutable real_type          m_aabb_max_angle;
    mutable real_type          m_aabb_max_size;
    mutable vector<Triangle2D> m_aabb_tri;

    bool
    aabb_intersect_ISO(
      Triangle2D    const & T1,
      real_type             offs,
      ClothoidCurve const * pC,
      Triangle2D    const & T2,
      real_type             C_offs,
      real_type           & ss1,
      real_type           & ss2
    ) const;

    #ifndef DOXYGEN_SHOULD_SKIP_THIS
    class T2D_approximate_collision {
      ClothoidCurve const * pC1;
      ClothoidCurve const * pC2;
    public:
      T2D_approximate_collision(
        ClothoidCurve const * _pC1,
        ClothoidCurve const * _pC2
      )
      : pC1(_pC1)
      , pC2(_pC2)
      {}

      bool
      operator () ( BBox::PtrBBox ptr1, BBox::PtrBBox ptr2 ) const {
        Triangle2D const & T1 = pC1->m_aabb_tri[size_t(ptr1->Ipos())];
        Triangle2D const & T2 = pC2->m_aabb_tri[size_t(ptr2->Ipos())];
        return T1.overlap(T2);
      }
    };

    class T2D_collision_ISO {
      ClothoidCurve const * pC1;
      real_type     const   m_offs1;
      ClothoidCurve const * pC2;
      real_type     const   m_offs2;
    public:
      T2D_collision_ISO(
        ClothoidCurve const * _pC1,
        real_type     const   _offs1,
        ClothoidCurve const * _pC2,
        real_type     const   _offs2
      )
      : pC1(_pC1)
      , m_offs1(_offs1)
      , pC2(_pC2)
      , m_offs2(_offs2)
      {}

      bool
      operator () ( BBox::PtrBBox ptr1, BBox::PtrBBox ptr2 ) const {
        Triangle2D const & T1 = pC1->m_aabb_tri[size_t(ptr1->Ipos())];
        Triangle2D const & T2 = pC2->m_aabb_tri[size_t(ptr2->Ipos())];
        real_type ss1, ss2;
        return pC1->aabb_intersect_ISO( T1, m_offs1, pC2, T2, m_offs2, ss1, ss2 );
      }
    };
    #endif

  public:

    #include "BaseCurve_using.hxx"

    ClothoidCurve()
    : BaseCurve(G2LIB_CLOTHOID)
    , m_aabb_done(false)
    {
      m_CD.x0     = 0;
      m_CD.y0     = 0;
      m_CD.theta0 = 0;
      m_CD.kappa0 = 0;
      m_CD.dk     = 0;
      m_L         = 0;
    }

    ClothoidCurve( ClothoidCurve const & s )
    : BaseCurve(G2LIB_CLOTHOID)
    , m_aabb_done(false)
    { copy(s); }

    explicit
    ClothoidCurve(
      real_type x0,
      real_type y0,
      real_type theta0,
      real_type k,
      real_type dk,
      real_type L
    )
    : BaseCurve(G2LIB_CLOTHOID)
    , m_aabb_done(false)
    {
      m_CD.x0     = x0;
      m_CD.y0     = y0;
      m_CD.theta0 = theta0;
      m_CD.kappa0 = k;
      m_CD.dk     = dk;
      m_L         = L;
    }

    explicit
    ClothoidCurve(
      real_type const * P0,
      real_type         theta0,
      real_type const * P1,
      real_type         theta1
    )
    : BaseCurve(G2LIB_CLOTHOID)
    , m_aabb_done(false)
    {
      build_G1( P0[0], P0[1], theta0, P1[0], P1[1], theta1 );
    }

    void
    copy( ClothoidCurve const & c ) {
      m_CD = c.m_CD;
      m_L  = c.m_L;
      m_aabb_done = false;
      m_aabb_tree.clear();
    }

    explicit
    ClothoidCurve( LineSegment const & LS )
    : BaseCurve(G2LIB_CLOTHOID)
    , m_aabb_done(false)
    {
      m_CD.x0     = LS.m_x0;
      m_CD.y0     = LS.m_y0;
      m_CD.theta0 = LS.m_theta0;
      m_CD.kappa0 = 0;
      m_CD.dk     = 0;
      m_L         = LS.m_L;
    }

    explicit
    ClothoidCurve( CircleArc const & C )
    : BaseCurve(G2LIB_CLOTHOID)
    , m_aabb_done(false)
    {
      m_CD.x0     = C.m_x0;
      m_CD.y0     = C.m_y0;
      m_CD.theta0 = C.m_theta0;
      m_CD.kappa0 = C.m_k;
      m_CD.dk     = 0;
      m_L         = C.m_L;
    }

    explicit
    ClothoidCurve( BaseCurve const & C );

    ClothoidCurve const & operator = ( ClothoidCurve const & s )
    { copy(s); return *this; }

    /*\
     |  _         _ _    _
     | | |__ _  _(_) |__| |
     | | '_ \ || | | / _` |
     | |_.__/\_,_|_|_\__,_|
    \*/
    void
    build(
      real_type x0,
      real_type y0,
      real_type theta0,
      real_type k,
      real_type dk,
      real_type L
    );

    int
    build_G1(
      real_type x0,
      real_type y0,
      real_type theta0,
      real_type x1,
      real_type y1,
      real_type theta1,
      real_type tol = 1e-12
    ) {
      m_aabb_done = false;
      m_aabb_tree.clear();
      return m_CD.build_G1( x0, y0, theta0, x1, y1, theta1, tol, m_L );
    }

    int
    build_G1_D(
      real_type x0,
      real_type y0,
      real_type theta0,
      real_type x1,
      real_type y1,
      real_type theta1,
      real_type L_D[2],
      real_type k_D[2],
      real_type dk_D[2],
      real_type tol = 1e-12
    ) {
      m_aabb_done = false;
      m_aabb_tree.clear();
      return m_CD.build_G1(
        x0, y0, theta0, x1, y1, theta1, tol, m_L, true, L_D, k_D, dk_D
      );
    }

    bool
    build_forward(
      real_type x0,
      real_type y0,
      real_type theta0,
      real_type kappa0,
      real_type x1,
      real_type y1,
      real_type tol = 1e-12
    ) {
      m_aabb_done = false;
      m_aabb_tree.clear();
      return m_CD.build_forward( x0, y0, theta0, kappa0, x1, y1, tol, m_L );
    }

    void
    build( LineSegment const & LS ) {
      m_CD.x0     = LS.m_x0;
      m_CD.y0     = LS.m_y0;
      m_CD.theta0 = LS.m_theta0;
      m_CD.kappa0 = 0;
      m_CD.dk     = 0;
      m_L         = LS.m_L;
      m_aabb_done = false;
      m_aabb_tree.clear();
    }

    void
    build( CircleArc const & C ) {
      m_CD.x0     = C.m_x0;
      m_CD.y0     = C.m_y0;
      m_CD.theta0 = C.m_theta0;
      m_CD.kappa0 = C.m_k;
      m_CD.dk     = 0;
      m_L         = C.m_L;
      m_aabb_done = false;
      m_aabb_tree.clear();
    }

    void
    Pinfinity( real_type & x, real_type & y, bool plus = true ) const
    { m_CD.Pinfinity( x, y, plus ); }

    real_type dkappa() const { return m_CD.dk; }

    real_type
    thetaTotalVariation() const;

    real_type
    thetaMinMax( real_type & thMin, real_type & thMax ) const;

    real_type
    deltaTheta() const
    { real_type thMin, thMax; return thetaMinMax( thMin, thMax ); }

    real_type
    curvatureMinMax( real_type & kMin, real_type & kMax ) const;

    real_type curvatureTotalVariation() const;

    real_type integralCurvature2() const;

    real_type integralJerk2() const;

    real_type integralSnap2() const;

    void
    optimized_sample_ISO(
      real_type                offs,
      int_type                 npts,
      real_type                max_angle,
      std::vector<real_type> & s
    ) const;

    void
    optimized_sample_SAE(
      real_type                offs,
      int_type                 npts,
      real_type                max_angle,
      std::vector<real_type> & s
    ) const {
      optimized_sample_ISO( -offs, npts, max_angle, s );
    }

    /*\
     |     _ _    _
     |  __| (_)__| |_ __ _ _ _  __ ___
     | / _` | (_-<  _/ _` | ' \/ _/ -_)
     | \__,_|_/__/\__\__,_|_||_\__\___|
    \*/
    real_type
    closestPointBySample(
      real_type   ds,
      real_type   qx,
      real_type   qy,
      real_type & X,
      real_type & Y,
      real_type & S
    ) const;

    real_type
    distanceBySample(
      real_type   ds,
      real_type   qx,
      real_type   qy,
      real_type & S
    ) const {
      real_type X, Y;
      return closestPointBySample( ds, qx, qy, X, Y, S );
    }

    real_type
    distanceBySample(
      real_type ds,
      real_type qx,
      real_type qy
    ) const {
      real_type X, Y, S;
      return closestPointBySample( ds, qx, qy, X, Y, S );
    }

    /*\
     |  _    _   _____    _                _
     | | |__| |_|_   _| _(_)__ _ _ _  __ _| |___
     | | '_ \ '_ \| || '_| / _` | ' \/ _` | / -_)
     | |_.__/_.__/|_||_| |_\__,_|_||_\__, |_\___|
     |                               |___/
    \*/

    bool
    bbTriangle(
      real_type & xx0, real_type & yy0,
      real_type & xx1, real_type & yy1,
      real_type & xx2, real_type & yy2
    ) const {
      return m_CD.bbTriangle( m_L, xx0, yy0, xx1, yy1, xx2, yy2 );
    }

    bool
    bbTriangle_ISO(
      real_type offs,
      real_type & xx0, real_type & yy0,
      real_type & xx1, real_type & yy1,
      real_type & xx2, real_type & yy2
    ) const {
      return m_CD.bbTriangle_ISO( m_L, offs, xx0, yy0, xx1, yy1, xx2, yy2 );
    }

    bool
    bbTriangle_SAE(
      real_type offs,
      real_type & xx0, real_type & yy0,
      real_type & xx1, real_type & yy1,
      real_type & xx2, real_type & yy2
    ) const {
      return m_CD.bbTriangle_SAE( m_L, offs, xx0, yy0, xx1, yy1, xx2, yy2 );
    }

    bool
    bbTriangle( Triangle2D & t, int_type icurve = 0 ) const {
      real_type x0, y0, x1, y1, x2, y2;
      bool ok = m_CD.bbTriangle( m_L, x0, y0, x1, y1, x2, y2 );
      if ( ok ) t.build( x0, y0, x1, y1, x2, y2, 0, 0, icurve );
      return ok;
    }

    bool
    bbTriangle_ISO( real_type offs, Triangle2D & t, int_type icurve = 0 ) const {
      real_type x0, y0, x1, y1, x2, y2;
      bool ok = m_CD.bbTriangle_ISO( m_L, offs, x0, y0, x1, y1, x2, y2 );
      if ( ok ) t.build( x0, y0, x1, y1, x2, y2, 0, 0, icurve );
      return ok;
    }

    bool
    bbTriangle_SAE( real_type offs, Triangle2D & t, int_type icurve = 0 ) const {
      real_type x0, y0, x1, y1, x2, y2;
      bool ok = m_CD.bbTriangle_SAE( m_L, offs, x0, y0, x1, y1, x2, y2 );
      if ( ok ) t.build( x0, y0, x1, y1, x2, y2, 0, 0, icurve );
      return ok;
    }

    void
    bbTriangles_ISO(
      real_type                 offs,
      std::vector<Triangle2D> & tvec,
      real_type                 max_angle = Utils::m_pi/6, // 30 degree
      real_type                 max_size  = 1e100,
      int_type                  icurve    = 0
    ) const override;

    void
    bbTriangles_SAE(
      real_type                 offs,
      std::vector<Triangle2D> & tvec,
      real_type                 max_angle = Utils::m_pi/6, // 30 degree
      real_type                 max_size  = 1e100,
      int_type                  icurve    = 0
    ) const override {
      this->bbTriangles_ISO( -offs, tvec, max_angle, max_size, icurve );
    }

    void
    bbTriangles(
      std::vector<Triangle2D> & tvec,
      real_type                 max_angle = Utils::m_pi/6, // 30 degree
      real_type                 max_size  = 1e100,
      int_type                  icurve    = 0
    ) const override {
      this->bbTriangles_ISO( 0, tvec, max_angle, max_size, icurve );
    }

    // . . . . . . . . . . . . . . . . . . . . . . . . . . . . . . . . . . . . .

    void
    bbox(
      real_type & xmin,
      real_type & ymin,
      real_type & xmax,
      real_type & ymax
    ) const override {
      bbox_ISO( 0, xmin, ymin, xmax, ymax );
    }

    void
    bbox_ISO(
      real_type   offs,
      real_type & xmin,
      real_type & ymin,
      real_type & xmax,
      real_type & ymax
    ) const override;

    // . . . . . . . . . . . . . . . . . . . . . . . . . . . . . . . . . . . . .

    real_type
    length() const override
    { return m_L; }

    real_type
    length_ISO( real_type ) const override {
      UTILS_ERROR0( "Offset length not available for Clothoids\n" );
      return 0;
    }

    real_type thetaBegin()   const override { return m_CD.theta0; }
    real_type kappaBegin()   const override { return m_CD.kappa0; }
    real_type xBegin()       const override { return m_CD.x0; }
    real_type xEnd()         const override { return m_CD.X(m_L); }
    real_type yBegin()       const override { return m_CD.y0; }
    real_type yEnd()         const override { return m_CD.Y(m_L); }
    real_type tx_Begin()     const override { return m_CD.tg0_x(); }
    real_type ty_Begin()     const override { return m_CD.tg0_y(); }
    real_type nx_Begin_ISO() const override { return m_CD.nor0_x_ISO(); }
    real_type ny_Begin_ISO() const override { return m_CD.nor0_y_ISO(); }

    // . . . . . . . . . . . . . . . . . . . . . . . . . . . . . . . . . . . . .

    /*\
     |  _____                   _   _   _
     | |_   _|   __ _ _ __   __| | | \ | |
     |   | |    / _` | '_ \ / _` | |  \| |
     |   | |   | (_| | | | | (_| | | |\  |
     |   |_|    \__,_|_| |_|\__,_| |_| \_|
    \*/

    real_type tx( real_type s ) const override { return m_CD.tg_x( s ); }
    real_type ty( real_type s ) const override { return m_CD.tg_y( s ); }
    real_type tx_D( real_type s ) const override { return m_CD.tg_x_D( s ); }
    real_type ty_D( real_type s ) const override { return m_CD.tg_y_D( s ); }
    real_type tx_DD( real_type s ) const override { return m_CD.tg_x_DD( s ); }
    real_type ty_DD( real_type s ) const override { return m_CD.tg_y_DD( s ); }
    real_type tx_DDD( real_type s ) const override { return m_CD.tg_x_DDD( s ); }
    real_type ty_DDD( real_type s ) const override { return m_CD.tg_y_DDD( s ); }

    // . . . . . . . . . . . . . . . . . . . . . . . . . . . . . . . . . . . . .

    void
    tg(
      real_type   s,
      real_type & tx,
      real_type & ty
    ) const override
    { m_CD.tg( s, tx, ty ); }

    void
    tg_D(
      real_type   s,
      real_type & tx_D,
      real_type & ty_D
    ) const override
    { m_CD.tg_D( s, tx_D, ty_D ); }

    void
    tg_DD(
      real_type   s,
      real_type & tx_DD,
      real_type & ty_DD
    ) const override
    { m_CD.tg_DD( s, tx_DD, ty_DD ); }

    void
    tg_DDD(
      real_type   s,
      real_type & tx_DDD,
      real_type & ty_DDD
    ) const override
    { m_CD.tg_DDD( s, tx_DDD, ty_DDD ); }

    // . . . . . . . . . . . . . . . . . . . . . . . . . . . . . . . . . . . . .

    real_type
    theta( real_type s ) const override
    { return m_CD.theta(s); }

    real_type
    theta_D( real_type s ) const override
    { return m_CD.kappa(s); }

    real_type
    theta_DD( real_type ) const override
    { return m_CD.dk; }

    real_type
    theta_DDD( real_type ) const override
    { return 0; }

    // . . . . . . . . . . . . . . . . . . . . . . . . . . . . . . . . . . . . .

    #ifdef G2LIB_COMPATIBILITY_MODE
    void
    evaluate(
      real_type   s,
      real_type & th,
      real_type & k,
      real_type & x,
      real_type & y
    ) const override
    { m_CD.evaluate( s, th, k, x, y ); }
    #endif

    // . . . . . . . . . . . . . . . . . . . . . . . . . . . . . . . . . . . . .

    real_type
    X( real_type s ) const override
    { return m_CD.X(s); }

    real_type
    X_D( real_type s ) const override
    { return m_CD.X_D(s); }

    real_type
    X_DD( real_type s ) const override
    { return m_CD.X_DD(s); }

    real_type
    X_DDD( real_type s ) const override
    { return m_CD.X_DDD(s); }

    real_type
    Y( real_type s ) const override
    { return m_CD.Y(s); }

    real_type
    Y_D( real_type s ) const override
    { return m_CD.Y_D(s); }

    real_type
    Y_DD ( real_type s ) const override
    { return m_CD.Y_DD(s); }

    real_type
    Y_DDD( real_type s ) const override
    { return m_CD.Y_DDD(s); }

    real_type
    X_ISO( real_type s, real_type offs ) const override
    { return m_CD.X_ISO(s,offs); }

    real_type
    X_ISO_D( real_type s, real_type offs ) const override
    { return m_CD.X_ISO_D(s,offs); }

    real_type
    X_ISO_DD( real_type s, real_type offs ) const override
    { return m_CD.X_ISO_DD(s,offs); }

    real_type
    X_ISO_DDD( real_type s, real_type offs ) const override
    { return m_CD.X_ISO_DDD(s,offs); }

    real_type
    Y_ISO( real_type s, real_type offs ) const override
    { return m_CD.Y_ISO(s,offs); }

    real_type
    Y_ISO_D( real_type s, real_type offs ) const override
    { return m_CD.Y_ISO_D(s,offs); }

    real_type
    Y_ISO_DD( real_type s, real_type offs ) const override
    { return m_CD.Y_ISO_DD(s,offs); }

    real_type
    Y_ISO_DDD( real_type s, real_type offs ) const override
    { return m_CD.Y_ISO_DDD(s,offs); }

    // . . . . . . . . . . . . . . . . . . . . . . . . . . . . . . . . . . . . .

    void
    eval(
      real_type   s,
      real_type & x,
      real_type & y
    ) const override
    { m_CD.eval( s, x, y ); }

    void
    eval_D(
      real_type   s,
      real_type & x_D,
      real_type & y_D
    ) const override
    { m_CD.eval_D( s, x_D, y_D ); }

    void
    eval_DD(
      real_type   s,
      real_type & x_DD,
      real_type & y_DD
    ) const override
    { m_CD.eval_DD( s, x_DD, y_DD ); }

    void
    eval_DDD(
      real_type   s,
      real_type & x_DDD,
      real_type & y_DDD
    ) const override
    { m_CD.eval_DDD( s, x_DDD, y_DDD ); }

    void
    eval_ISO(
      real_type   s,
      real_type   offs,
      real_type & x,
      real_type & y
    ) const override
    { m_CD.eval_ISO( s, offs, x, y ); }

    void
    eval_ISO_D(
      real_type   s,
      real_type   offs,
      real_type & x_D,
      real_type & y_D
    ) const override
    { m_CD.eval_ISO_D( s, offs, x_D, y_D ); }

    void
    eval_ISO_DD(
      real_type   s,
      real_type   offs,
      real_type & x_DD,
      real_type & y_DD
    ) const override
    { m_CD.eval_ISO_DD( s, offs, x_DD, y_DD ); }

    void
    eval_ISO_DDD(
      real_type   s,
      real_type   offs,
      real_type & x_DDD,
      real_type & y_DDD
    ) const override
    { m_CD.eval_ISO_DDD( s, offs, x_DDD, y_DDD ); }

    /*\
     |  _                        __
     | | |_ _ __ __ _ _ __  ___ / _| ___  _ __ _ __ ___
     | | __| '__/ _` | '_ \/ __| |_ / _ \| '__| '_ ` _ \
     | | |_| | | (_| | | | \__ \  _| (_) | |  | | | | | |
     |  \__|_|  \__,_|_| |_|___/_|  \___/|_|  |_| |_| |_|
    \*/

    void
    translate( real_type tx, real_type ty ) override
    { m_CD.x0 += tx; m_CD.y0 += ty; }

    void
    rotate( real_type angle, real_type cx, real_type cy ) override
    { m_CD.rotate( angle, cx, cy ); }

    void
    scale( real_type s ) override {
      m_CD.kappa0 /= s;
      m_CD.dk     /= s*s;
      m_L         *= s;
    }

    void
    reverse() override
    { m_CD.reverse(m_L); }

    void
    changeOrigin( real_type newx0, real_type newy0 ) override
    { m_CD.x0 = newx0; m_CD.y0 = newy0; }

    void
    trim( real_type s_begin, real_type s_end ) override {
      m_CD.origin_at( s_begin );
      m_L = s_end - s_begin;
    }

    void
    changeCurvilinearOrigin( real_type s0, real_type newL ) {
      m_CD.origin_at( s0 );
      m_L = newL;
    }

    /*\
     |        _                     _   ____       _       _
     |    ___| | ___  ___  ___  ___| |_|  _ \ ___ (_)_ __ | |_
     |   / __| |/ _ \/ __|/ _ \/ __| __| |_) / _ \| | '_ \| __|
     |  | (__| | (_) \__ \  __/\__ \ |_|  __/ (_) | | | | | |_
     |   \___|_|\___/|___/\___||___/\__|_|   \___/|_|_| |_|\__|
    \*/

    int_type
    closestPoint_ISO(
      real_type   qx,
      real_type   qy,
      real_type & x,
      real_type & y,
      real_type & s,
      real_type & t,
      real_type & dst
    ) const override;

    int_type
    closestPoint_ISO(
      real_type   qx,
      real_type   qy,
      real_type   offs,
      real_type & x,
      real_type & y,
      real_type & s,
      real_type & t,
      real_type & dst
    ) const override;

    // . . . . . . . . . . . . . . . . . . . . . . . . . . . . . . . . . . . . .
    // . . . . . . . . . . . . . . . . . . . . . . . . . . . . . . . . . . . . .

    /*\
     |             _ _ _     _
     |    ___ ___ | | (_)___(_) ___  _ __
     |   / __/ _ \| | | / __| |/ _ \| '_ \
     |  | (_| (_) | | | \__ \ | (_) | | | |
     |   \___\___/|_|_|_|___/_|\___/|_| |_|
    \*/

    #ifndef DOXYGEN_SHOULD_SKIP_THIS
    void
    build_AABBtree_ISO(
      real_type offs,
      real_type max_angle = Utils::m_pi/18, // 10 degree
      real_type max_size  = 1e100
    ) const;
    #endif

    // collision detection
    bool
    approximate_collision_ISO(
      real_type             offs,
      ClothoidCurve const & c,
      real_type             c_offs,
      real_type             max_angle,
      real_type             max_size
    ) const;

    bool
    collision( ClothoidCurve const & C ) const;

    bool
    collision_ISO(
      real_type             offs,
      ClothoidCurve const & C,
      real_type             offs_C
    ) const;

    /*\
     |   _       _                          _
     |  (_)_ __ | |_ ___ _ __ ___  ___  ___| |_
     |  | | '_ \| __/ _ \ '__/ __|/ _ \/ __| __|
     |  | | | | | ||  __/ |  \__ \  __/ (__| |_
     |  |_|_| |_|\__\___|_|  |___/\___|\___|\__|
    \*/

    void
    intersect(
      ClothoidCurve const & C,
      IntersectList       & ilist,
      bool                  swap_s_vals
    ) const {
      intersect_ISO( 0, C, 0, ilist, swap_s_vals );
    }

    void
    intersect_ISO(
      real_type               offs,
      ClothoidCurve const   & C,
      real_type               offs_C,
      IntersectList         & ilist,
      bool                    swap_s_vals
    ) const;

    void
    info( ostream_type & stream ) const override
    { stream << "Clothoid\n" << *this << '\n'; }

    friend
    ostream_type &
    operator << ( ostream_type & stream, ClothoidCurve const & c );

  };

}
```

### Quick search

### Table of Contents

- Matlab Interface Manual
- C++ API
- MATLAB API

«
hide menu

menu
sidebar
»

### Navigation

- index
- toc
- Clothoids »
- Program Listing for File Clothoid.hxx

© Copyright 2021, Enrico Bertolazzi and Marco Frego.
Created using Sphinx 4.2.0.
