# Supplementary material for: Smooth Interpolating Curves with Local Control and Monotone Alternating Curvature
Source: Comput Graph Forum. 2022 Oct 6;41(5):25–38. doi: 10.1111/cgf.14600 (PMC9827861; doi:10.1111/cgf.14600)
Supplement: Supplementary file 1 — Supplement Material [file CGF-41-25-s001.zip › Local-Smooth-Interpolating-MonoCurvature/extern/clothoids/docs/api-cpp/program_listing_file_Clothoids_ClothoidAsyPlot.hxx.html]

Program Listing for File ClothoidAsyPlot.hxx — Clothoids v2.0.9

### Navigation

- index
- toc
- Clothoids »
- Program Listing for File ClothoidAsyPlot.hxx

# Program Listing for File ClothoidAsyPlot.hxx¶

↰ Return to documentation for file (`Clothoids/ClothoidAsyPlot.hxx`)

```
/*--------------------------------------------------------------------------*\
 |                                                                          |
 |  Copyright (C) 2017                                                      |
 |                                                                          |
 |         , __                 , __                                        |
 |        /|/  \               /|/  \                                       |
 |         | __/ _   ,_         | __/ _   ,_                                |
 |         |   \|/  /  |  |   | |   \|/  /  |  |   |                        |
 |         |(__/|__/   |_/ \_/|/|(__/|__/   |_/ \_/|/                       |
 |                           /|                   /|                        |
 |                           \|                   \|                        |
 |                                                                          |
 |      Enrico Bertolazzi and Marco Frego                                   |
 |      Dipartimento di Ingegneria Industriale                              |
 |      Universita` degli Studi di Trento                                   |
 |      email: enrico.bertolazzi@unitn.it                                   |
 |      email: marco.frego@unitn.it                                         |
 |                                                                          |
\*--------------------------------------------------------------------------*/

namespace G2lib {

  using std::string;
  using std::ofstream;

  class AsyPlot {
  public:
    AsyPlot( string filename, bool showAxes );
    ~AsyPlot();

    void
    drawClothoid(
      ClothoidCurve const& c,
      std::string const & penna="black",
      real_type offset = 0
    ) const;

    void dot( real_type x, real_type y, string const & penna="black" ) const;
    void triangle(Triangle2D const & t, string const & penna="black" ) const;

    void
    drawRect(
      real_type x0, real_type y0,
      real_type x1, real_type y1,
      real_type x2, real_type y2,
      real_type x3, real_type y3,
      string const & penna="black"
    ) const;

    void
    drawLine(
      real_type x0, real_type y0,
      real_type x1, real_type y1,
      std::string const & penna="black"
    ) const;

    void
    label(
      string const & text,
      real_type      x,
      real_type      y,
      string const & placement = "",
      string const & penna = "black"
    ) const;

    void
    displayAxes(
      string const & labX,
      string const & labY,
      real_type      xmin,
      real_type      xmax,
      real_type      ymin,
      real_type      ymax
    ) const;

  private:
    mutable ofstream file;
    string  filename;
    bool showAxes;
    bool openFile();
    bool closeFile();
    void initFile();
    void displayAxes() const;
    void compileFile();
  };
}
```

### Quick search

### Table of Contents

- Matlab Interface Manual
- C++ API
- MATLAB API

«
hide menu

menu
sidebar
»

### Navigation

- index
- toc
- Clothoids »
- Program Listing for File ClothoidAsyPlot.hxx

© Copyright 2021, Enrico Bertolazzi and Marco Frego.
Created using Sphinx 4.2.0.
