# Supplementary material for: Smooth Interpolating Curves with Local Control and Monotone Alternating Curvature
Source: Comput Graph Forum. 2022 Oct 6;41(5):25–38. doi: 10.1111/cgf.14600 (PMC9827861; doi:10.1111/cgf.14600)
Supplement: Supplementary file 1 — Supplement Material [file CGF-41-25-s001.zip › Local-Smooth-Interpolating-MonoCurvature/extern/clothoids/docs/api-cpp/program_listing_file_Clothoids_ClothoidList.hxx.html]

Program Listing for File ClothoidList.hxx — Clothoids v2.0.9

### Navigation

- index
- toc
- Clothoids »
- Program Listing for File ClothoidList.hxx

# Program Listing for File ClothoidList.hxx¶

↰ Return to documentation for file (`Clothoids/ClothoidList.hxx`)

```
/*--------------------------------------------------------------------------*\
 |                                                                          |
 |  Copyright (C) 2017                                                      |
 |                                                                          |
 |         , __                 , __                                        |
 |        /|/  \               /|/  \                                       |
 |         | __/ _   ,_         | __/ _   ,_                                |
 |         |   \|/  /  |  |   | |   \|/  /  |  |   |                        |
 |         |(__/|__/   |_/ \_/|/|(__/|__/   |_/ \_/|/                       |
 |                           /|                   /|                        |
 |                           \|                   \|                        |
 |                                                                          |
 |      Enrico Bertolazzi                                                   |
 |      Dipartimento di Ingegneria Industriale                              |
 |      Universita` degli Studi di Trento                                   |
 |      email: enrico.bertolazzi@unitn.it                                   |
 |                                                                          |
\*--------------------------------------------------------------------------*/


namespace G2lib {

  using std::vector;

  /*\
   |    ____ ____            _           ____
   |   / ___|___ \ ___  ___ | |_   _____|___ \ __ _ _ __ ___
   |  | |  _  __) / __|/ _ \| \ \ / / _ \ __) / _` | '__/ __|
   |  | |_| |/ __/\__ \ (_) | |\ V /  __// __/ (_| | | | (__
   |   \____|_____|___/\___/|_| \_/ \___|_____\__,_|_|  \___|
  \*/


  class G2solve2arc {

    real_type tolerance;
    int_type  maxIter;

    real_type x0;
    real_type y0;
    real_type theta0;
    real_type kappa0;

    real_type x1;
    real_type y1;
    real_type theta1;
    real_type kappa1;

    // standard problem
    real_type lambda, phi, xbar, ybar;
    real_type th0, th1;
    real_type k0, k1;
    real_type DeltaK;
    real_type DeltaTheta;

    ClothoidCurve S0, S1;

    void
    evalA(
      real_type   alpha,
      real_type   L,
      real_type & A
    ) const;

    void
    evalA(
      real_type   alpha,
      real_type   L,
      real_type & A,
      real_type & A_1,
      real_type & A_2
    ) const;

    void
    evalG(
      real_type alpha,
      real_type L,
      real_type th,
      real_type k,
      real_type G[2]
    ) const;

    void
    evalG(
      real_type alpha,
      real_type L,
      real_type th,
      real_type k,
      real_type G[2],
      real_type G_1[2],
      real_type G_2[2]
    ) const;

    void
    evalF( real_type const vars[2], real_type F[2] ) const;

    void
    evalFJ(
      real_type const vars[2],
      real_type       F[2],
      real_type       J[2][2]
    ) const;

    void
    buildSolution( real_type alpha, real_type L );

  public:

    G2solve2arc()
    : tolerance(1e-10)
    , maxIter(20)
    , x0(0)
    , y0(0)
    , theta0(0)
    , kappa0(0)
    , x1(0)
    , y1(0)
    , theta1(0)
    , kappa1(0)
    , lambda(0)
    , phi(0)
    , xbar(0)
    , ybar(0)
    , th0(0)
    , th1(0)
    , k0(0)
    , k1(0)
    {}

    ~G2solve2arc() {}

    int
    build(
      real_type x0, real_type y0, real_type theta0, real_type kappa0,
      real_type x1, real_type y1, real_type theta1, real_type kappa1
    );

    void setTolerance( real_type tol );

    void setMaxIter( int tol );

    int solve();

    ClothoidCurve const & getS0() const { return S0; }

    ClothoidCurve const & getS1() const { return S1; }

  };

  /*\
   |    ____ ____            _            ____ _     ____
   |   / ___|___ \ ___  ___ | |_   _____ / ___| |   / ___|
   |  | |  _  __) / __|/ _ \| \ \ / / _ \ |   | |  | |
   |  | |_| |/ __/\__ \ (_) | |\ V /  __/ |___| |__| |___
   |   \____|_____|___/\___/|_| \_/ \___|\____|_____\____|
  \*/

  class G2solveCLC {

    real_type tolerance;
    int       maxIter;

    real_type x0;
    real_type y0;
    real_type theta0;
    real_type kappa0;
    real_type x1;
    real_type y1;
    real_type theta1;
    real_type kappa1;

    // standard problem
    real_type lambda, phi, xbar, ybar;
    real_type th0, th1;
    real_type k0, k1;

    ClothoidCurve S0, SM, S1;

    bool
    buildSolution( real_type sM, real_type thM );

  public:

    G2solveCLC()
    : tolerance(1e-10)
    , maxIter(20)
    , x0(0)
    , y0(0)
    , theta0(0)
    , kappa0(0)
    , x1(0)
    , y1(0)
    , theta1(0)
    , kappa1(0)
    , lambda(0)
    , phi(0)
    , xbar(0)
    , ybar(0)
    , th0(0)
    , th1(0)
    , k0(0)
    , k1(0)
    {}

    ~G2solveCLC() {}

    int
    build(
      real_type x0, real_type y0, real_type theta0, real_type kappa0,
      real_type x1, real_type y1, real_type theta1, real_type kappa1
    );

    void setTolerance( real_type tol );

    void setMaxIter( int tol );

    int solve();

    ClothoidCurve const & getS0() const { return S0; }

    ClothoidCurve const & getSM() const { return SM; }

    ClothoidCurve const & getS1() const { return S1; }

    void save( ostream_type & stream ) const;
  };

  /*\
   |    ____ ____            _           _____
   |   / ___|___ \ ___  ___ | |_   _____|___ /  __ _ _ __ ___
   |  | |  _  __) / __|/ _ \| \ \ / / _ \ |_ \ / _` | '__/ __|
   |  | |_| |/ __/\__ \ (_) | |\ V /  __/___) | (_| | | | (__
   |   \____|_____|___/\___/|_| \_/ \___|____/ \__,_|_|  \___|
  \*/

  class G2solve3arc {

    ClothoidCurve S0, SM, S1;

    real_type tolerance;
    int       maxIter;

    // G2 interpolation data
    real_type x0;
    real_type y0;
    real_type theta0;
    real_type kappa0;
    real_type x1;
    real_type y1;
    real_type theta1;
    real_type kappa1;

    // standard scaled problem
    real_type phi, Lscale;
    real_type th0, th1;
    real_type s0, s1;

    // precomputed values
    real_type K0, K1, c0, c1, c2, c3, c4, c5, c6, c7, c8, c9, c10, c11, c12, c13, c14;

    void
    evalFJ(
      real_type const vars[2],
      real_type       F[2],
      real_type       J[2][2]
    ) const;

    void
    evalF( real_type const vars[2], real_type F[2] ) const;

    void
    buildSolution( real_type sM, real_type thM );

    int
    solve( real_type sM_guess, real_type thM_guess );

  public:

    G2solve3arc()
    : tolerance(1e-10)
    , maxIter(100)
    {}

    ~G2solve3arc() {}

    void setTolerance( real_type tol );

    void setMaxIter( int miter );

    int
    build(
      real_type x0,
      real_type y0,
      real_type theta0,
      real_type kappa0,
      real_type x1,
      real_type y1,
      real_type theta1,
      real_type kappa1,
      real_type Dmax = 0,
      real_type dmax = 0
    );

    int
    build_fixed_length(
      real_type s0,
      real_type x0,
      real_type y0,
      real_type theta0,
      real_type kappa0,
      real_type s1,
      real_type x1,
      real_type y1,
      real_type theta1,
      real_type kappa1
    );

    ClothoidCurve const & getS0() const { return S0; }

    ClothoidCurve const & getS1() const { return S1; }

    ClothoidCurve const & getSM() const { return SM; }

    real_type
    totalLength() const {
      return S0.length() + S1.length() + SM.length();
    }

    real_type
    thetaTotalVariation() const {
      return S0.thetaTotalVariation() +
             S1.thetaTotalVariation() +
             SM.thetaTotalVariation();
    }

    real_type
    curvatureTotalVariation() const {
      return S0.curvatureTotalVariation() +
             S1.curvatureTotalVariation() +
             SM.curvatureTotalVariation();
    }

    real_type
    integralCurvature2() const {
      return S0.integralCurvature2() +
             S1.integralCurvature2() +
             SM.integralCurvature2();
    }

    real_type
    integralJerk2() const {
      return S0.integralJerk2() +
             S1.integralJerk2() +
             SM.integralJerk2();
    }

    real_type
    integralSnap2() const {
      return S0.integralSnap2() +
             S1.integralSnap2() +
             SM.integralSnap2();
    }

    real_type
    thetaMinMax( real_type & thMin, real_type & thMax ) const;

    real_type
    deltaTheta() const
    { real_type thMin, thMax; return thetaMinMax( thMin, thMax ); }

    real_type
    curvatureMinMax( real_type & kMin, real_type & kMax ) const;

    real_type theta( real_type s ) const;

    real_type theta_D( real_type s ) const;

    real_type theta_DD( real_type s ) const;

    real_type theta_DDD( real_type s ) const;

    real_type X( real_type s ) const;

    real_type Y( real_type s ) const;

    real_type xBegin() const { return S0.xBegin(); }

    real_type yBegin() const { return S0.yBegin(); }

    real_type kappaBegin() const { return S0.kappaBegin(); }

    real_type thetaBegin() const { return S0.thetaBegin(); }

    real_type xEnd()const { return S1.xEnd(); }

    real_type yEnd() const { return S1.yEnd(); }

    real_type kappaEnd() const { return S1.kappaEnd(); }

    real_type thetaEnd() const { return S1.thetaEnd(); }

    void
    eval(
      real_type   s,
      real_type & theta,
      real_type & kappa,
      real_type & x,
      real_type & y
    ) const;

    void eval( real_type s, real_type & x, real_type & y ) const;

    void eval_D( real_type s, real_type & x_D, real_type & y_D ) const;

    void eval_DD( real_type s, real_type & x_DD, real_type & y_DD ) const;

    void eval_DDD( real_type s, real_type & x_DDD, real_type & y_DDD ) const;

    void eval_ISO( real_type s, real_type offs, real_type & x, real_type & y ) const;

    void eval_ISO_D( real_type s, real_type offs, real_type & x_D, real_type & y_D ) const;

    void eval_ISO_DD( real_type s, real_type offs, real_type & x_DD, real_type & y_DD ) const;

    void eval_ISO_DDD( real_type s, real_type offs, real_type & x_DDD, real_type & y_DDD ) const;

    void
    rotate( real_type angle, real_type cx, real_type cy ) {
      S0.rotate( angle, cx, cy );
      S1.rotate( angle, cx, cy );
      SM.rotate( angle, cx, cy );
    }

    void
    translate( real_type tx, real_type ty ){
      S0.translate( tx, ty );
      S1.translate( tx, ty );
      SM.translate( tx, ty );
    }

    void
    reverse() {
      ClothoidCurve tmp(S0); S1 = S0; S0 = tmp;
      S0.reverse();
      S1.reverse();
      SM.reverse();
    }

    friend
    ostream_type &
    operator << ( ostream_type & stream, ClothoidCurve const & c );

    void save( ostream_type & stream ) const;
  };

  /*\
   |   ____ _       _   _           _     _ _     _     _
   |  / ___| | ___ | |_| |__   ___ (_) __| | |   (_)___| |_
   | | |   | |/ _ \| __| '_ \ / _ \| |/ _` | |   | / __| __|
   | | |___| | (_) | |_| | | | (_) | | (_| | |___| \__ \ |_
   |  \____|_|\___/ \__|_| |_|\___/|_|\__,_|_____|_|___/\__|
   |
  \*/
  class ClothoidList : public BaseCurve {

    bool                  m_curve_is_closed;
    vector<real_type>     m_s0;
    vector<ClothoidCurve> m_clotoidList;

    mutable Utils::BinarySearch<int_type> m_lastInterval;

    mutable bool               m_aabb_done;
    mutable AABBtree           m_aabb_tree;
    mutable real_type          m_aabb_offs;
    mutable real_type          m_aabb_max_angle;
    mutable real_type          m_aabb_max_size;
    mutable vector<Triangle2D> m_aabb_tri;

    #ifndef DOXYGEN_SHOULD_SKIP_THIS
    class T2D_collision_list_ISO {
      ClothoidList const * pList1;
      real_type    const   m_offs1;
      ClothoidList const * pList2;
      real_type    const   m_offs2;
    public:
      T2D_collision_list_ISO(
        ClothoidList const * _pList1,
        real_type    const   _offs1,
        ClothoidList const * _pList2,
        real_type    const   _offs2
      )
      : pList1(_pList1)
      , m_offs1(_offs1)
      , pList2(_pList2)
      , m_offs2(_offs2)
      {}

      bool
      operator () ( BBox::PtrBBox ptr1, BBox::PtrBBox ptr2 ) const {
        Triangle2D    const & T1 = pList1->m_aabb_tri[size_t(ptr1->Ipos())];
        Triangle2D    const & T2 = pList2->m_aabb_tri[size_t(ptr2->Ipos())];
        ClothoidCurve const & C1 = pList1->get(T1.Icurve());
        ClothoidCurve const & C2 = pList2->get(T2.Icurve());
        real_type ss1, ss2;
        return C1.aabb_intersect_ISO( T1, m_offs1, &C2, T2, m_offs2, ss1, ss2 );
      }
    };
    #endif

    void
    resetLastInterval() {
      bool ok;
      int_type & lastInterval = *m_lastInterval.search( std::this_thread::get_id(), ok );
      lastInterval = 0;
    }

    int_type
    closestPoint_internal(
      real_type   qx,
      real_type   qy,
      real_type   offs,
      real_type & x,
      real_type & y,
      real_type & s,
      real_type & DST
    ) const;

  public:

    #include "BaseCurve_using.hxx"

    ClothoidList()
    : BaseCurve(G2LIB_CLOTHOID_LIST)
    , m_curve_is_closed(false)
    , m_aabb_done(false)
    { this->resetLastInterval(); }

    ~ClothoidList() override {
      m_s0.clear();
      m_clotoidList.clear();
      m_aabb_tri.clear();
    }

    ClothoidList( ClothoidList const & s )
    : BaseCurve(G2LIB_CLOTHOID_LIST)
    , m_curve_is_closed(false)
    , m_aabb_done(false)
    { this->resetLastInterval(); copy(s); }

    void init();

    void reserve( int_type n );

    void copy( ClothoidList const & L );

    ClothoidList const & operator = ( ClothoidList const & s )
    { copy(s); return *this; }

    explicit ClothoidList( LineSegment const & LS );

    explicit ClothoidList( CircleArc const & C );

    explicit ClothoidList( Biarc const & B );

    explicit ClothoidList( BiarcList const & BL );

    explicit ClothoidList( ClothoidCurve const & CL );

    explicit ClothoidList( PolyLine const & PL );

    explicit ClothoidList( BaseCurve const & C );

    void push_back( LineSegment const & c );

    void push_back( CircleArc const & c );

    void push_back( Biarc const & c );

    void push_back( BiarcList const & c );

    void push_back( ClothoidCurve const & c );

    void push_back( ClothoidList const & c );

    void push_back( PolyLine const & c );

    void
    push_back(
      real_type kappa0,
      real_type dkappa,
      real_type L
    );

    void
    push_back(
      real_type x0,
      real_type y0,
      real_type theta0,
      real_type kappa0,
      real_type dkappa,
      real_type L
    );

    void
    push_back_G1(
      real_type x1,
      real_type y1,
      real_type theta1
    );

    void
    push_back_G1(
      real_type x0,
      real_type y0,
      real_type theta0,
      real_type x1,
      real_type y1,
      real_type theta1
    );

    bool is_closed() const { return m_curve_is_closed; }

    void make_closed() { m_curve_is_closed = true; }

    void make_open() { m_curve_is_closed = false; }

    real_type closure_gap_x()  const { return this->xEnd() - this->xBegin(); }

    real_type closure_gap_y()  const { return this->yEnd() - this->yBegin(); }

    real_type closure_gap_tx() const { return this->tx_End() - this->tx_Begin(); }

    real_type closure_gap_ty() const { return this->ty_End() - this->ty_Begin(); }

    bool
    closure_check( real_type tol_xy = 1e-6, real_type tol_tg = 1e-6 ) const {
      return std::abs(closure_gap_x())  < tol_xy &&
             std::abs(closure_gap_y())  < tol_xy &&
             std::abs(closure_gap_tx()) < tol_tg &&
             std::abs(closure_gap_ty()) < tol_tg;
    }

    bool
    build_G1(
      int_type          n,
      real_type const * x,
      real_type const * y
    );

    bool
    build_G1(
      int_type          n,
      real_type const * x,
      real_type const * y,
      real_type const * theta
    );

    bool
    build(
      real_type         x0,
      real_type         y0,
      real_type         theta0,
      int_type          n,
      real_type const * s,
      real_type const * kappa
    );

    bool
    build(
      real_type                 x0,
      real_type                 y0,
      real_type                 theta0,
      vector<real_type> const & s,
      vector<real_type> const & kappa
    ) {
      if ( s.size() != kappa.size() ) return false;
      return build(
        x0, y0, theta0,
        int_type(s.size()),
        &s.front(), &kappa.front()
      );
    }

    bool
    build_raw(
      int_type          n,
      real_type const * x,
      real_type const * y,
      real_type const * abscissa,
      real_type const * theta,
      real_type const * kappa
    );

    bool
    build_raw(
      vector<real_type> const & x,
      vector<real_type> const & y,
      vector<real_type> const & abscissa,
      vector<real_type> const & theta,
      vector<real_type> const & kappa
    ) {
      int_type n = int_type(x.size());
      if ( n != int_type(y.size())        ||
           n != int_type(abscissa.size()) ||
           n != int_type(theta.size())    ||
           n != int_type(kappa.size()) ) return false;
      return build_raw(
        n, &x.front(), &y.front(),
        &abscissa.front(), &theta.front(), &kappa.front()
      );
    }

    ClothoidCurve const & get( int_type idx ) const;

    ClothoidCurve const & getAtS( real_type s ) const;

    int_type numSegments() const { return int_type(m_clotoidList.size()); }

    void wrap_in_range( real_type & s ) const;

    int_type findAtS( real_type & s ) const;

    // . . . . . . . . . . . . . . . . . . . . . . . . . . . . . . . . . . . . .

    real_type length() const override;
    real_type length_ISO( real_type offs ) const override;

    real_type
    segment_length( int_type nseg ) const;

    real_type
    segment_length_ISO( int_type nseg, real_type offs ) const;

    real_type
    segment_length_SAE( int_type nseg, real_type offs ) const
    { return segment_length_ISO( nseg, -offs ); }

    /*\
     |  _    _   _____    _                _
     | | |__| |_|_   _| _(_)__ _ _ _  __ _| |___
     | | '_ \ '_ \| || '_| / _` | ' \/ _` | / -_)
     | |_.__/_.__/|_||_| |_\__,_|_||_\__, |_\___|
     |                               |___/
    \*/

    void
    bbTriangles(
      std::vector<Triangle2D> & tvec,
      real_type                 max_angle = Utils::m_pi/6, // 30 degree
      real_type                 max_size  = 1e100,
      int_type                  icurve    = 0
    ) const override;

    void
    bbTriangles_ISO(
      real_type                 offs,
      std::vector<Triangle2D> & tvec,
      real_type                 max_angle = Utils::m_pi/6, // 30 degree
      real_type                 max_size  = 1e100,
      int_type                  icurve    = 0
    ) const override;

    void
    bbTriangles_SAE(
      real_type                 offs,
      std::vector<Triangle2D> & tvec,
      real_type                 max_angle = Utils::m_pi/6, // 30 degree
      real_type                 max_size  = 1e100,
      int_type                  icurve    = 0
    ) const override {
      this->bbTriangles_ISO( -offs, tvec, max_angle, max_size, icurve );
    }

    #ifndef DOXYGEN_SHOULD_SKIP_THIS
    void
    build_AABBtree_ISO(
      real_type offs,
      real_type max_angle = Utils::m_pi/6, // 30 degree
      real_type max_size  = 1e100
    ) const;
    #endif

    /*\
     |   _     _
     |  | |__ | |__   _____  __
     |  | '_ \| '_ \ / _ \ \/ /
     |  | |_) | |_) | (_) >  <
     |  |_.__/|_.__/ \___/_/\_\
    \*/

    void
    bbox(
      real_type & xmin,
      real_type & ymin,
      real_type & xmax,
      real_type & ymax
    ) const override {
      bbox_ISO( 0, xmin, ymin, xmax, ymax );
    }

    void
    bbox_ISO(
      real_type   offs,
      real_type & xmin,
      real_type & ymin,
      real_type & xmax,
      real_type & ymax
    ) const override;

    /*\
     |   ____             _          _______           _
     |  | __ )  ___  __ _(_)_ __    / / ____|_ __   __| |
     |  |  _ \ / _ \/ _` | | '_ \  / /|  _| | '_ \ / _` |
     |  | |_) |  __/ (_| | | | | |/ / | |___| | | | (_| |
     |  |____/ \___|\__, |_|_| |_/_/  |_____|_| |_|\__,_|
     |              |___/
    \*/

    real_type
    thetaBegin() const override
    { return m_clotoidList.front().thetaBegin(); }

    real_type
    thetaEnd() const override
    { return m_clotoidList.back().thetaEnd(); }

    real_type
    xBegin() const override
    { return m_clotoidList.front().xBegin(); }

    real_type
    yBegin() const override
    { return m_clotoidList.front().yBegin(); }

    real_type
    xEnd() const override
    { return m_clotoidList.back().xEnd(); }

    real_type
    yEnd() const override
    { return m_clotoidList.back().yEnd(); }

    real_type
    xBegin_ISO( real_type offs ) const override
    { return m_clotoidList.front().xBegin_ISO( offs ); }

    real_type
    yBegin_ISO( real_type offs ) const override
    { return m_clotoidList.front().yBegin_ISO( offs ); }

    real_type xEnd_ISO( real_type offs ) const override
    { return m_clotoidList.back().xEnd_ISO( offs ); }

    real_type
    yEnd_ISO( real_type offs ) const override
    { return m_clotoidList.back().yEnd_ISO( offs ); }

    real_type tx_Begin() const override
    { return m_clotoidList.front().tx_Begin(); }

    real_type
    ty_Begin() const override
    { return m_clotoidList.front().ty_Begin(); }

    real_type
    tx_End() const override
    { return m_clotoidList.back().tx_End(); }

    real_type
    ty_End() const override
    { return m_clotoidList.back().ty_End(); }

    real_type
    nx_Begin_ISO() const override
    { return m_clotoidList.front().nx_Begin_ISO(); }

    real_type
    ny_Begin_ISO() const override
    { return m_clotoidList.front().ny_Begin_ISO(); }

    real_type
    nx_End_ISO() const override
    { return m_clotoidList.back().nx_End_ISO(); }

    real_type
    ny_End_ISO() const override
    { return m_clotoidList.back().ny_End_ISO(); }

    /*\
     |  _   _          _
     | | |_| |__   ___| |_ __ _
     | | __| '_ \ / _ \ __/ _` |
     | | |_| | | |  __/ || (_| |
     |  \__|_| |_|\___|\__\__,_|
    \*/

    real_type theta    ( real_type s ) const override;
    real_type theta_D  ( real_type s ) const override;
    real_type theta_DD ( real_type s ) const override;
    real_type theta_DDD( real_type s ) const override;

    /*\
     |  _____                   _   _   _
     | |_   _|   __ _ _ __   __| | | \ | |
     |   | |    / _` | '_ \ / _` | |  \| |
     |   | |   | (_| | | | | (_| | | |\  |
     |   |_|    \__,_|_| |_|\__,_| |_| \_|
    \*/

    real_type tx    ( real_type s ) const override;
    real_type ty    ( real_type s ) const override;
    real_type tx_D  ( real_type s ) const override;
    real_type ty_D  ( real_type s ) const override;
    real_type tx_DD ( real_type s ) const override;
    real_type ty_DD ( real_type s ) const override;
    real_type tx_DDD( real_type s ) const override;
    real_type ty_DDD( real_type s ) const override;

    // . . . . . . . . . . . . . . . . . . . . . . . . . . . . . . . . . . . . .

    void
    tg(
      real_type   s,
      real_type & tg_x,
      real_type & tg_y
    ) const override;

    void
    tg_D(
      real_type   s,
      real_type & tg_x_D,
      real_type & tg_y_D
    ) const override;

    void
    tg_DD(
      real_type   s,
      real_type & tg_x_DD,
      real_type & tg_y_DD
    ) const override;

    void
    tg_DDD(
      real_type   s,
      real_type & tg_x_DDD,
      real_type & tg_y_DDD
    ) const override;

    // . . . . . . . . . . . . . . . . . . . . . . . . . . . . . . . . . . . . .

    void
    evaluate(
      real_type   s,
      real_type & th,
      real_type & k,
      real_type & x,
      real_type & y
    ) const override;

    // . . . . . . . . . . . . . . . . . . . . . . . . . . . . . . . . . . . . .

    void
    evaluate_ISO(
      real_type   s,
      real_type   offs,
      real_type & th,
      real_type & k,
      real_type & x,
      real_type & y
    ) const override;

    // . . . . . . . . . . . . . . . . . . . . . . . . . . . . . . . . . . . . .

    real_type X    ( real_type s ) const override;
    real_type Y    ( real_type s ) const override;
    real_type X_D  ( real_type s ) const override;
    real_type Y_D  ( real_type s ) const override;
    real_type X_DD ( real_type s ) const override;
    real_type Y_DD ( real_type s ) const override;
    real_type X_DDD( real_type s ) const override;
    real_type Y_DDD( real_type s ) const override;

    // . . . . . . . . . . . . . . . . . . . . . . . . . . . . . . . . . . . . .

    void
    eval(
      real_type   s,
      real_type & x,
      real_type & y
    ) const override;

    void
    eval_D(
      real_type   s,
      real_type & x_D,
      real_type & y_D
    ) const override;

    void
    eval_DD(
      real_type   s,
      real_type & x_DD,
      real_type & y_DD
    ) const override;

    void
    eval_DDD(
      real_type   s,
      real_type & x_DDD,
      real_type & y_DDD
    ) const override;

    /*\
     |         __  __          _
     |   ___  / _|/ _|___  ___| |_
     |  / _ \| |_| |_/ __|/ _ \ __|
     | | (_) |  _|  _\__ \  __/ |_
     |  \___/|_| |_| |___/\___|\__|
    \*/

    real_type X_ISO    ( real_type s, real_type offs ) const override;
    real_type Y_ISO    ( real_type s, real_type offs ) const override;
    real_type X_ISO_D  ( real_type s, real_type offs ) const override;
    real_type Y_ISO_D  ( real_type s, real_type offs ) const override;
    real_type X_ISO_DD ( real_type s, real_type offs ) const override;
    real_type Y_ISO_DD ( real_type s, real_type offs ) const override;
    real_type X_ISO_DDD( real_type s, real_type offs ) const override;
    real_type Y_ISO_DDD( real_type s, real_type offs ) const override;

    // . . . . . . . . . . . . . . . . . . . . . . . . . . . . . . . . . . . . .

    void
    eval_ISO(
      real_type   s,
      real_type   offs,
      real_type & x,
      real_type & y
    ) const override;

    void
    eval_ISO_D(
      real_type   s,
      real_type   offs,
      real_type & x_D,
      real_type & y_D
    ) const override;

    void
    eval_ISO_DD(
      real_type   s,
      real_type   offs,
      real_type & x_DD,
      real_type & y_DD
    ) const override;

    void
    eval_ISO_DDD(
      real_type   s,
      real_type   offs,
      real_type & x_DDD,
      real_type & y_DDD
    ) const override;

    /*\
     |  _                        __
     | | |_ _ __ __ _ _ __  ___ / _| ___  _ __ _ __ ___
     | | __| '__/ _` | '_ \/ __| |_ / _ \| '__| '_ ` _ \
     | | |_| | | (_| | | | \__ \  _| (_) | |  | | | | | |
     |  \__|_|  \__,_|_| |_|___/_|  \___/|_|  |_| |_| |_|
    \*/

    void translate( real_type tx, real_type ty ) override;
    void rotate( real_type angle, real_type cx, real_type cy ) override;
    void scale( real_type sc ) override;
    void reverse() override;
    void changeOrigin( real_type newx0, real_type newy0 ) override;
    void trim( real_type s_begin, real_type s_end ) override;
    void trim( real_type s_begin, real_type s_end, ClothoidList & newCL ) const;

    /*\
     |      _ _     _
     |   __| (_)___| |_ __ _ _ __   ___ ___
     |  / _` | / __| __/ _` | '_ \ / __/ _ \
     | | (_| | \__ \ || (_| | | | | (_|  __/
     |  \__,_|_|___/\__\__,_|_| |_|\___\___|
    \*/

    int_type
    closestPoint_ISO(
      real_type   qx,
      real_type   qy,
      real_type & x,
      real_type & y,
      real_type & s,
      real_type & t,
      real_type & dst
    ) const override;

    int_type
    closestPoint_ISO(
      real_type   qx,
      real_type   qy,
      real_type   offs,
      real_type & x,
      real_type & y,
      real_type & s,
      real_type & t,
      real_type & dst
    ) const override;

    /*\
     |      _ _     _
     |   __| (_)___| |_ __ _ _ __   ___ ___
     |  / _` | / __| __/ _` | '_ \ / __/ _ \
     | | (_| | \__ \ || (_| | | | | (_|  __/
     |  \__,_|_|___/\__\__,_|_| |_|\___\___|
    \*/

    int_type
    closestSegment( real_type qx, real_type qy ) const;

    int_type
    closestPointInRange_ISO(
      real_type   qx,
      real_type   qy,
      int_type    icurve_begin,
      int_type    icurve_end,
      real_type & x,
      real_type & y,
      real_type & s,
      real_type & t,
      real_type & dst,
      int_type  & icurve
    ) const;

    int_type
    closestPointInRange_SAE(
      real_type   qx,
      real_type   qy,
      int_type    icurve_begin,
      int_type    icurve_end,
      real_type & x,
      real_type & y,
      real_type & s,
      real_type & t,
      real_type & dst,
      int_type  & icurve
    ) const {
      int_type res = this->closestPointInRange_ISO(
        qx, qy, icurve_begin, icurve_end, x, y, s, t, dst, icurve
      );
      t = -t;
      return res;
    }

    int_type
    closestPointInSRange_ISO(
      real_type   qx,
      real_type   qy,
      real_type   s_begin,
      real_type   s_end,
      real_type & x,
      real_type & y,
      real_type & s,
      real_type & t,
      real_type & dst,
      int_type  & icurve
    ) const;

    int_type
    closestPointInSRange_SAE(
      real_type   qx,
      real_type   qy,
      int_type    s_begin,
      int_type    s_end,
      real_type & x,
      real_type & y,
      real_type & s,
      real_type & t,
      real_type & dst,
      int_type  & icurve
    ) const {
      int_type res = this->closestPointInSRange_ISO(
        qx, qy, s_begin, s_end, x, y, s, t, dst, icurve
      );
      t = -t;
      return res;
    }

    void
    info( ostream_type & stream ) const override
    { stream << "ClothoidList\n" << *this << '\n'; }

    friend
    ostream_type &
    operator << ( ostream_type & stream, ClothoidList const & CL );

    void
    getSK( real_type * s, real_type * kappa ) const;

    void
    getSK(
      std::vector<real_type> & s,
      std::vector<real_type> & kappa
    ) const {
      s.resize( m_clotoidList.size()+1 );
      kappa.resize( m_clotoidList.size()+1 );
      getSK( &s.front(), &kappa.front() );
    }

    void
    getSTK(
      real_type * s,
      real_type * theta,
      real_type * kappa
    ) const;

    void
    getSTK(
      std::vector<real_type> & s,
      std::vector<real_type> & theta,
      std::vector<real_type> & kappa
    ) const {
      s.resize( m_clotoidList.size()+1 );
      theta.resize( m_clotoidList.size()+1 );
      kappa.resize( m_clotoidList.size()+1 );
      getSTK( &s.front(), &theta.front(), &kappa.front() );
    }

    void
    getXY( real_type * x, real_type * y ) const;

    void
    getDeltaTheta( real_type * deltaTheta ) const;

    void
    getDeltaKappa( real_type * deltaKappa ) const;

    int_type
    findST1(
      real_type   x,
      real_type   y,
      real_type & s,
      real_type & t
    ) const;

    int_type
    findST1(
      int_type    ibegin,
      int_type    iend,
      real_type   x,
      real_type   y,
      real_type & s,
      real_type & t
    ) const;

    /*\
     |             _ _ _     _
     |    ___ ___ | | (_)___(_) ___  _ __
     |   / __/ _ \| | | / __| |/ _ \| '_ \
     |  | (_| (_) | | | \__ \ | (_) | | | |
     |   \___\___/|_|_|_|___/_|\___/|_| |_|
    \*/

    bool
    collision( ClothoidList const & C ) const;

    bool
    collision_ISO(
      real_type            offs,
      ClothoidList const & CL,
      real_type            offs_C
    ) const;

    /*\
     |   _       _                          _
     |  (_)_ __ | |_ ___ _ __ ___  ___  ___| |_
     |  | | '_ \| __/ _ \ '__/ __|/ _ \/ __| __|
     |  | | | | | ||  __/ |  \__ \  __/ (__| |_
     |  |_|_| |_|\__\___|_|  |___/\___|\___|\__|
    \*/

    void
    intersect(
      ClothoidList const & CL,
      IntersectList      & ilist,
      bool                 swap_s_vals
    ) const {
      intersect_ISO( 0, CL, 0, ilist, swap_s_vals );
    }

    void
    intersect_ISO(
      real_type            offs,
      ClothoidList const & CL,
      real_type            offs_obj,
      IntersectList      & ilist,
      bool                 swap_s_vals
    ) const;

    void
    export_table( ostream_type & stream ) const;

    void
    export_ruby( ostream_type & stream ) const;

    void save( ostream_type & stream ) const;

    void load( istream_type & stream, real_type epsi = 1e-8 );

  };

  /*\
   |
   |    ___ _     _   _        _    _ ___      _ _           ___ ___
   |   / __| |___| |_| |_  ___(_)__| / __|_ __| (_)_ _  ___ / __|_  )
   |  | (__| / _ \  _| ' \/ _ \ / _` \__ \ '_ \ | | ' \/ -_) (_ |/ /
   |   \___|_\___/\__|_||_\___/_\__,_|___/ .__/_|_|_||_\___|\___/___|
   |                                     |_|
  \*/

  class ClothoidSplineG2 {
  public:
    typedef enum { P1 = 1, P2, P3, P4, P5, P6, P7, P8, P9 } TargetType;

  private:

    Utils::Malloc<real_type> realValues;

    real_type * m_x;
    real_type * m_y;
    TargetType  m_tt;
    real_type   m_theta_I;
    real_type   m_theta_F;
    int_type    m_npts;

    // work vector
    mutable real_type * m_k;
    mutable real_type * m_dk;
    mutable real_type * m_L;
    mutable real_type * m_kL;
    mutable real_type * m_L_1;
    mutable real_type * m_L_2;
    mutable real_type * m_k_1;
    mutable real_type * m_k_2;
    mutable real_type * m_dk_1;
    mutable real_type * m_dk_2;

    real_type
    diff2pi( real_type in ) const {
      return in-Utils::m_2pi*round(in/Utils::m_2pi);
    }

  public:

    ClothoidSplineG2()
    : realValues("ClothoidSplineG2"), m_tt(P1)
    {}

    ~ClothoidSplineG2() {}

    void
    setP1( real_type theta0, real_type thetaN )
    { m_tt = P1; m_theta_I = theta0; m_theta_F = thetaN; }

    void setP2() { m_tt = P2; }
    void setP3() { m_tt = P3; }
    void setP4() { m_tt = P4; }
    void setP5() { m_tt = P5; }
    void setP6() { m_tt = P6; }
    void setP7() { m_tt = P7; }
    void setP8() { m_tt = P8; }
    void setP9() { m_tt = P9; }

    void
    build(
      real_type const * xvec,
      real_type const * yvec,
      int_type          npts
    );

    int_type numPnts() const { return m_npts; }
    int_type numTheta() const;
    int_type numConstraints() const;

    void
    guess(
      real_type * theta_guess,
      real_type * theta_min,
      real_type * theta_max
    ) const;

    bool
    objective( real_type const * theta, real_type & f ) const;

    bool
    gradient( real_type const * theta, real_type * g ) const;

    bool
    constraints( real_type const * theta, real_type * c ) const;

    int_type
    jacobian_nnz() const;

    bool
    jacobian_pattern( int_type * i, int_type * j ) const;

    bool
    jacobian_pattern_matlab( real_type * i, real_type * j ) const;

    bool
    jacobian( real_type const * theta, real_type * vals ) const;

    void
    info( ostream_type & stream ) const
    { stream << "ClothoidSplineG2\n" << *this << '\n'; }

    friend
    ostream_type &
    operator << ( ostream_type & stream, ClothoidSplineG2 const & c );

  };

}
```

### Quick search

### Table of Contents

- Matlab Interface Manual
- C++ API
- MATLAB API

«
hide menu

menu
sidebar
»

### Navigation

- index
- toc
- Clothoids »
- Program Listing for File ClothoidList.hxx

© Copyright 2021, Enrico Bertolazzi and Marco Frego.
Created using Sphinx 4.2.0.
