# Supplementary material for: Smooth Interpolating Curves with Local Control and Monotone Alternating Curvature
Source: Comput Graph Forum. 2022 Oct 6;41(5):25–38. doi: 10.1111/cgf.14600 (PMC9827861; doi:10.1111/cgf.14600)
Supplement: Supplementary file 1 — Supplement Material [file CGF-41-25-s001.zip › Local-Smooth-Interpolating-MonoCurvature/extern/clothoids/docs/api-cpp/program_listing_file_Clothoids_Fresnel.hxx.html]

Program Listing for File Fresnel.hxx — Clothoids v2.0.9

### Navigation

- index
- toc
- Clothoids »
- Program Listing for File Fresnel.hxx

# Program Listing for File Fresnel.hxx¶

↰ Return to documentation for file (`Clothoids/Fresnel.hxx`)

```
/*--------------------------------------------------------------------------*\
 |                                                                          |
 |  Copyright (C) 2017                                                      |
 |                                                                          |
 |         , __                 , __                                        |
 |        /|/  \               /|/  \                                       |
 |         | __/ _   ,_         | __/ _   ,_                                |
 |         |   \|/  /  |  |   | |   \|/  /  |  |   |                        |
 |         |(__/|__/   |_/ \_/|/|(__/|__/   |_/ \_/|/                       |
 |                           /|                   /|                        |
 |                           \|                   \|                        |
 |                                                                          |
 |      Enrico Bertolazzi                                                   |
 |      Dipartimento di Ingegneria Industriale                              |
 |      Universita` degli Studi di Trento                                   |
 |      email: enrico.bertolazzi@unitn.it                                   |
 |                                                                          |
\*--------------------------------------------------------------------------*/


namespace G2lib {

  // - - - - - - - - - - - - - - - - - - - - - - - - - - - - - - - - - - - - - -

  /*\
   |   _____                         _
   |  |  ___| __ ___  ___ _ __   ___| |
   |  | |_ | '__/ _ \/ __| '_ \ / _ \ |
   |  |  _|| | |  __/\__ \ | | |  __/ |
   |  |_|  |_|  \___||___/_| |_|\___|_|
  \*/
  /*
   * Compute Fresnel integrals
   */
  void
  FresnelCS(
    real_type   x,
    real_type & C,
    real_type & S
  );

  void
  FresnelCS(
    int_type    nk,
    real_type   x,
    real_type * C,
    real_type * S
  );

  void
  GeneralizedFresnelCS(
    int_type    nk,
    real_type   a,
    real_type   b,
    real_type   c,
    real_type * intC,
    real_type * intS
  );

  void
  GeneralizedFresnelCS(
    real_type   a,
    real_type   b,
    real_type   c,
    real_type & intC,
    real_type & intS
  );

  // - - - - - - - - - - - - - - - - - - - - - - - - - - - - - - - - - - - - - -

  #ifndef DOXYGEN_SHOULD_SKIP_THIS

  class ClothoidData {
  public:

    real_type x0;
    real_type y0;
    real_type theta0;
    real_type kappa0;
    real_type dk;

    ClothoidData()
    : x0(0)
    , y0(0)
    , theta0(0)
    , kappa0(0)
    , dk(0)
    {}

    real_type
    deltaTheta( real_type s ) const
    { return s*(kappa0 + 0.5*s*dk); }

    real_type theta
    ( real_type s ) const
    { return theta0 + s*(kappa0 + 0.5*s*dk); }

    real_type theta_D  ( real_type s ) const { return kappa0 + s*dk; }
    real_type theta_DD ( real_type   ) const { return dk; }
    real_type theta_DDD( real_type   ) const { return 0; }

    real_type kappa    ( real_type s ) const { return kappa0 + s*dk; }
    real_type kappa_D  ( real_type   ) const { return dk; }
    real_type kappa_DD ( real_type   ) const { return 0; }
    real_type kappa_DDD( real_type   ) const { return 0; }

    real_type X( real_type s ) const;
    real_type Y( real_type s ) const;
    real_type X_D( real_type s ) const;
    real_type Y_D( real_type s ) const;
    real_type X_DD( real_type s ) const;
    real_type Y_DD( real_type s ) const;
    real_type X_DDD( real_type s ) const;
    real_type Y_DDD( real_type s ) const;

    // - - - - - - - - - - - - - - - - - - - - - - - - - - - - - - - -
    real_type X_ISO    ( real_type s, real_type offs ) const;
    real_type Y_ISO    ( real_type s, real_type offs ) const;
    real_type X_ISO_D  ( real_type s, real_type offs ) const;
    real_type Y_ISO_D  ( real_type s, real_type offs ) const;
    real_type X_ISO_DD ( real_type s, real_type offs ) const;
    real_type Y_ISO_DD ( real_type s, real_type offs ) const;
    real_type X_ISO_DDD( real_type s, real_type offs ) const;
    real_type Y_ISO_DDD( real_type s, real_type offs ) const;

    // - - - - - - - - - - - - - - - - - - - - - - - - - - - - - - - -

    real_type X_SAE    ( real_type s, real_type offs ) const;
    real_type Y_SAE    ( real_type s, real_type offs ) const;
    real_type X_SAE_D  ( real_type s, real_type offs ) const;
    real_type Y_SAE_D  ( real_type s, real_type offs ) const;
    real_type X_SAE_DD ( real_type s, real_type offs ) const;
    real_type Y_SAE_DD ( real_type s, real_type offs ) const;
    real_type X_SAE_DDD( real_type s, real_type offs ) const;
    real_type Y_SAE_DDD( real_type s, real_type offs ) const;

    // - - - - - - - - - - - - - - - - - - - - - - - - - - - - - - - -

    real_type tg0_x() const { return cos(this->theta0); }
    real_type tg0_y() const { return sin(this->theta0); }

    real_type tg_x( real_type s ) const { return cos(this->theta(s)); }
    real_type tg_y( real_type s ) const { return sin(this->theta(s)); }

    real_type tg_x_D( real_type s ) const;
    real_type tg_y_D( real_type s ) const;

    real_type tg_x_DD( real_type s ) const;
    real_type tg_y_DD( real_type s ) const;

    real_type tg_x_DDD( real_type s ) const;
    real_type tg_y_DDD( real_type s ) const;

    real_type nor0_x_ISO() const { return -this->tg0_y(); }
    real_type nor0_y_ISO() const { return this->tg0_x(); }

    real_type nor_x_ISO( real_type s ) const { return -this->tg_y(s); }
    real_type nor_y_ISO( real_type s ) const { return this->tg_x(s); }

    real_type nor_x_ISO_D( real_type s ) const { return -this->tg_y_D(s); }
    real_type nor_y_ISO_D( real_type s ) const { return this->tg_x_D(s);  }

    real_type nor_x_ISO_DD( real_type s ) const { return -this->tg_y_DD(s);  }
    real_type nor_y_ISO_DD( real_type s ) const { return this->tg_x_DD(s); }

    real_type nor_x_ISO_DDD( real_type s ) const { return -this->tg_y_DDD(s); }
    real_type nor_y_ISO_DDD( real_type s ) const { return this->tg_x_DDD(s); }

    real_type nor0_x_SAE() const { return this->tg0_y(); }
    real_type nor0_y_SAE() const { return -this->tg0_x(); }

    real_type nor_x_SAE( real_type s ) const { return this->tg_y(s); }
    real_type nor_y_SAE( real_type s ) const { return -this->tg_x(s); }

    real_type nor_x_SAE_D( real_type s ) const { return this->tg_y_D(s); }
    real_type nor_y_SAE_D( real_type s ) const { return -this->tg_x_D(s);  }

    real_type nor_x_SAE_DD( real_type s ) const { return this->tg_y_DD(s);  }
    real_type nor_y_SAE_DD( real_type s ) const { return -this->tg_x_DD(s); }

    real_type nor_x_SAE_DDD( real_type s ) const { return this->tg_y_DDD(s); }
    real_type nor_y_SAE_DDD( real_type s ) const { return -this->tg_x_DDD(s); }

    void tg( real_type s, real_type & tx, real_type & ty ) const;
    void tg_D( real_type s, real_type & tx, real_type & ty ) const;
    void tg_DD( real_type s, real_type & tx, real_type & ty ) const;
    void tg_DDD( real_type s, real_type & tx, real_type & ty ) const;

    void nor_ISO( real_type s, real_type & nx, real_type & ny ) const;
    void nor_ISO_D( real_type s, real_type & nx_D, real_type & ny_D ) const;
    void nor_ISO_DD( real_type s, real_type & nx_DD, real_type & ny_DD ) const;
    void nor_ISO_DDD( real_type s, real_type & nx_DDD, real_type & ny_DDD ) const;

    void nor_SAE( real_type s, real_type & nx, real_type & ny ) const;
    void nor_SAE_D( real_type s, real_type & nx_D, real_type & ny_D ) const;
    void nor_SAE_DD( real_type s, real_type & nx_DD, real_type & ny_DD ) const;
    void nor_SAE_DDD( real_type s, real_type & nx_DDD, real_type & ny_DDD ) const;

    void
    evaluate(
      real_type   s,
      real_type & theta,
      real_type & kappa,
      real_type & x,
      real_type & y
    ) const;

    void
    eval(
      real_type   s,
      real_type & x,
      real_type & y
    ) const;

    void
    eval_D(
      real_type   s,
      real_type & x_D,
      real_type & y_D
    ) const;

    void
    eval_DD(
      real_type   s,
      real_type & x_DD,
      real_type & y_DD
    ) const;

    void
    eval_DDD(
      real_type   s,
      real_type & x_DDD,
      real_type & y_DDD
    ) const;

    void
    eval_ISO(
      real_type   s,
      real_type   offs,
      real_type & x,
      real_type & y
    ) const;

    void
    eval_ISO_D(
      real_type   s,
      real_type   offs,
      real_type & x_D,
      real_type & y_D
    ) const;

    void
    eval_ISO_DD(
      real_type   s,
      real_type   offs,
      real_type & x_DD,
      real_type & y_DD
    ) const;

    void
    eval_ISO_DDD(
      real_type   s,
      real_type   offs,
      real_type & x_DDD,
      real_type & y_DDD
    ) const;

    void
    eval_SAE(
      real_type   s,
      real_type   offs,
      real_type & x,
      real_type & y
    ) const {
      this->eval_ISO( s, -offs, x, y );
    }

    void
    eval_SAE_D(
      real_type   s,
      real_type   offs,
      real_type & x_D,
      real_type & y_D
    ) const {
      this->eval_ISO_D( s, -offs, x_D, y_D );
    }

    void
    eval_DAE_DD(
      real_type   s,
      real_type   offs,
      real_type & x_DD,
      real_type & y_DD
    ) const {
      this->eval_ISO_DD( s, -offs, x_DD, y_DD );
    }

    void
    eval_SAE_DDD(
      real_type   s,
      real_type   offs,
      real_type & x_DDD,
      real_type & y_DDD
    ) const {
      this->eval_ISO_DDD( s, -offs, x_DDD, y_DDD );
    }

    void
    eval( real_type s, ClothoidData & C ) const;

    real_type c0x() const { return x0 - (sin(theta0)/kappa0); }
    real_type c0y() const { return y0 + (cos(theta0)/kappa0); }

    void
    Pinfinity( real_type & x, real_type & y, bool plus ) const;

    void
    reverse( real_type L );

    void
    reverse( real_type L, ClothoidData & out) const;

    void
    rotate( real_type angle, real_type cx, real_type cy );

    void
    origin_at( real_type s_origin );

    real_type
    split_at_flex( ClothoidData & C0, ClothoidData & C1 ) const;

    real_type
    aplus( real_type dtheta ) const;

    bool
    bbTriangle(
      real_type   L,
      real_type & xx0, real_type & yy0,
      real_type & xx1, real_type & yy1,
      real_type & xx2, real_type & yy2
    ) const;

    bool
    bbTriangle_ISO(
      real_type   L,
      real_type   offs,
      real_type & xx0, real_type & yy0,
      real_type & xx1, real_type & yy1,
      real_type & xx2, real_type & yy2
    ) const;

    bool
    bbTriangle_SAE(
      real_type   L,
      real_type   offs,
      real_type & xx0, real_type & yy0,
      real_type & xx1, real_type & yy1,
      real_type & xx2, real_type & yy2
    ) const {
      return this->bbTriangle_ISO( L, -offs, xx0, yy0, xx1, yy1, xx2, yy2 );
    }

    int
    build_G1(
      real_type   x0,
      real_type   y0,
      real_type   theta0,
      real_type   x1,
      real_type   y1,
      real_type   theta1,
      real_type   tol,
      real_type & L,
      bool        compute_deriv = false,
      real_type   L_D[2]        = nullptr,
      real_type   k_D[2]        = nullptr,
      real_type   dk_D[2]       = nullptr
    );

    bool
    build_forward(
      real_type   x0,
      real_type   y0,
      real_type   theta0,
      real_type   kappa0,
      real_type   x1,
      real_type   y1,
      real_type   tol,
      real_type & L
    );

    void
    info( ostream_type & s ) const;

  };

  #endif

}
```

### Quick search

### Table of Contents

- Matlab Interface Manual
- C++ API
- MATLAB API

«
hide menu

menu
sidebar
»

### Navigation

- index
- toc
- Clothoids »
- Program Listing for File Fresnel.hxx

© Copyright 2021, Enrico Bertolazzi and Marco Frego.
Created using Sphinx 4.2.0.
