# Supplementary material for: Smooth Interpolating Curves with Local Control and Monotone Alternating Curvature
Source: Comput Graph Forum. 2022 Oct 6;41(5):25–38. doi: 10.1111/cgf.14600 (PMC9827861; doi:10.1111/cgf.14600)
Supplement: Supplementary file 1 — Supplement Material [file CGF-41-25-s001.zip › Local-Smooth-Interpolating-MonoCurvature/extern/clothoids/docs/api-cpp/program_listing_file_Clothoids_G2lib.hxx.html]

Program Listing for File G2lib.hxx — Clothoids v2.0.9

### Navigation

- index
- toc
- Clothoids »
- Program Listing for File G2lib.hxx

# Program Listing for File G2lib.hxx¶

↰ Return to documentation for file (`Clothoids/G2lib.hxx`)

```
/*--------------------------------------------------------------------------*\
 |                                                                          |
 |  Copyright (C) 2017                                                      |
 |                                                                          |
 |         , __                 , __                                        |
 |        /|/  \               /|/  \                                       |
 |         | __/ _   ,_         | __/ _   ,_                                |
 |         |   \|/  /  |  |   | |   \|/  /  |  |   |                        |
 |         |(__/|__/   |_/ \_/|/|(__/|__/   |_/ \_/|/                       |
 |                           /|                   /|                        |
 |                           \|                   \|                        |
 |                                                                          |
 |      Enrico Bertolazzi                                                   |
 |      Dipartimento di Ingegneria Industriale                              |
 |      Universita` degli Studi di Trento                                   |
 |      email: enrico.bertolazzi@unitn.it                                   |
 |                                                                          |
\*--------------------------------------------------------------------------*/


namespace G2lib {

  extern real_type const m_1_sqrt_pi;

  extern real_type const machepsi;
  extern real_type const machepsi10;
  extern real_type const machepsi100;
  extern real_type const machepsi1000;
  extern real_type const sqrtMachepsi;
  extern bool            intersect_with_AABBtree;

  #ifdef G2LIB_COMPATIBILITY_MODE

  extern bool use_ISO;

  static
  inline
  void
  lib_use_ISO()
  { use_ISO = true; }

  static
  inline
  void
  lib_use_SAE()
  { use_ISO = false; }

  #endif

  static
  inline
  void
  noAABBtree()
  { intersect_with_AABBtree = false; }

  static
  inline
  void
  yesAABBtree()
  { intersect_with_AABBtree = true; }

  /*
   * sin(x)/x
   */
  real_type Sinc( real_type x );
  real_type Sinc_D( real_type x );
  real_type Sinc_DD( real_type x );
  real_type Sinc_DDD( real_type x );

  /*
   * (1-cos(x))/x
   */
  real_type Cosc( real_type x );
  real_type Cosc_D( real_type x );
  real_type Cosc_DD( real_type x );
  real_type Cosc_DDD( real_type x );

  /*
   * atan(x)/x
   */
  real_type Atanc( real_type x );
  real_type Atanc_D( real_type x );
  real_type Atanc_DD( real_type x );
  real_type Atanc_DDD( real_type x );

  void rangeSymm( real_type & ang );

  // - - - - - - - - - - - - - - - - - - - - - - - - - - - - - - - - - - - - - -
  inline
  void
  minmax3(
    real_type   a,
    real_type   b,
    real_type   c,
    real_type & vmin,
    real_type & vmax
  ) {
    vmin = vmax = a;
    if ( b < vmin ) vmin = b;
    else            vmax = b;
    if      ( c < vmin ) vmin = c;
    else if ( c > vmax ) vmax = c;
  }

  // - - - - - - - - - - - - - - - - - - - - - - - - - - - - - - - - - - - - - -
  real_type
  projectPointOnCircleArc(
    real_type x0,
    real_type y0,
    real_type c0,
    real_type s0,
    real_type k,
    real_type L,
    real_type qx,
    real_type qy
  );

  // - - - - - - - - - - - - - - - - - - - - - - - - - - - - - - - - - - - - - -
  real_type
  projectPointOnCircle(
    real_type x0,
    real_type y0,
    real_type theta0,
    real_type k,
    real_type qx,
    real_type qy
  );

  // - - - - - - - - - - - - - - - - - - - - - - - - - - - - - - - - - - - - - -
  inline
  bool
  pointInsideCircle(
    real_type x0,
    real_type y0,
    real_type c0,
    real_type s0,
    real_type k,
    real_type qx,
    real_type qy
  ) {
    real_type cx  = x0 - s0/k;
    real_type cy  = y0 + c0/k;
    real_type dst = hypot( qx - cx, qy - cy );
    return dst*k <= 1;
  }

  int_type
  solveLinearQuadratic(
    real_type   A,
    real_type   B,
    real_type   C,
    real_type   a,
    real_type   b,
    real_type   c,
    real_type * x,
    real_type * y
  );

  int_type
  solveLinearQuadratic2(
    real_type   A,
    real_type   B,
    real_type   C,
    real_type * x,
    real_type * y
  );

  int_type
  intersectCircleCircle(
    real_type   x1,
    real_type   y1,
    real_type   theta1,
    real_type   kappa1,
    real_type   x2,
    real_type   y2,
    real_type   theta2,
    real_type   kappa2,
    real_type * s1,
    real_type * s2
  );

  /*\
   |   ____        _           ____       ____
   |  / ___|  ___ | |_   _____|___ \__  _|___ \
   |  \___ \ / _ \| \ \ / / _ \ __) \ \/ / __) |
   |   ___) | (_) | |\ V /  __// __/ >  < / __/
   |  |____/ \___/|_| \_/ \___|_____/_/\_\_____|
  \*/
  class Solve2x2 {
    int_type  i[2], j[2];
    real_type LU[2][2];
    real_type epsi;
    bool      singular;

  public:

    Solve2x2() : epsi(1e-10) {}
    bool factorize( real_type A[2][2] );

    bool solve( real_type const b[2], real_type x[2] ) const;
  };

  int_type
  isCounterClockwise(
    real_type const * P1,
    real_type const * P2,
    real_type const * P3
  );

  int_type
  isPointInTriangle(
    real_type const * pt,
    real_type const * P1,
    real_type const * P2,
    real_type const * P3
  );

  // - - - - - - - - - - - - - - - - - - - - - - - - - - - - - - - - - - - - - -
  /*\
   |  __  ____   __  _          _____ _          _
   |  \ \/ /\ \ / / | |_ ___   |_   _| |__   ___| |_ __ _
   |   \  /  \ V /  | __/ _ \    | | | '_ \ / _ \ __/ _` |
   |   /  \   | |   | || (_) |   | | | | | |  __/ || (_| |
   |  /_/\_\  |_|    \__\___/    |_| |_| |_|\___|\__\__,_|
  \*/

  void
  xy_to_guess_angle(
    int_type          npts,
    real_type const * x,
    real_type const * y,
    real_type       * theta,
    real_type       * theta_min,
    real_type       * theta_max,
    real_type       * omega,
    real_type       * len
  );

}
```

### Quick search

### Table of Contents

- Matlab Interface Manual
- C++ API
- MATLAB API

«
hide menu

menu
sidebar
»

### Navigation

- index
- toc
- Clothoids »
- Program Listing for File G2lib.hxx

© Copyright 2021, Enrico Bertolazzi and Marco Frego.
Created using Sphinx 4.2.0.
